# Ag_3_PO_4_ enables the generation of long-lived radical cations for visible light-driven [2 + 2] and [4 + 2] pericyclic reactions

**DOI:** 10.1038/s41467-024-45217-y

**Published:** 2024-02-01

**Authors:** Lirong Guo, Rongchen Chu, Xinyu Hao, Yu Lei, Haibin Li, Dongge Ma, Guo Wang, Chen-Ho Tung, Yifeng Wang

**Affiliations:** 1 Key Lab for Colloid and Interface Science of Ministry of Education, School of Chemistry and Chemical Engineering Shandong University Jinan, 250100 Jinan, China; 2 Key Laboratory of Photochemistry, Institute of Chemistry Chinese Academy of Sciences Beijing, 100190 Beijing, China; 3 College of Chemistry and Materials Engineering Beijing Technology and Business University Beijing, 100048 Beijing, China; 4 Department of Chemistry Capital Normal University Beijing, 100048 Beijing, China

**Keywords:** Photocatalysis, Excited states

## Abstract

Photocatalytic redox reactions are important for synthesizing fine chemicals from olefins, but the limited lifetime of radical cation intermediates severely restricts semiconductor photocatalysis efficiency. Here, we report that Ag_3_PO_4_ can efficiently catalyze intramolecular and intermolecular [2 + 2] and Diels-Alder cycloadditions under visible-light irradiation. The approach is additive-free, catalyst-recyclable. Mechanistic studies indicate that visible-light irradiation on Ag_3_PO_4_ generates holes with high oxidation power, which oxidize aromatic alkene adsorbates into radical cations. In photoreduced Ag_3_PO_4_, the conduction band electron (*e*_CB_^−^) has low reduction power due to the delocalization among the Ag^+^-lattices, while the particle surfaces have a strong electrostatic interaction with the radical cations, which considerably stabilize the radical cations against recombination with *e*_CB_^−^. The radical cation on the particle’s surfaces has a lifetime of more than 2 ms, 75 times longer than homogeneous systems. Our findings highlight the effectiveness of inorganic semiconductors for challenging radical cation-mediated synthesis driven by sunlight.

## Abstract (web summary)

Photoredox catalysis is an important approach for synthesizing fine chemicals from olefins, but the limited lifetime of radical cation intermediates severely restricts the efficiency. Here, the authors report that Ag3PO4 can efficiently catalyze intramolecular and intermolecular [2 + 2] and Diels–Alder cycloadditions under visible-light irradiation.

## Introduction

**Fig. 1:**
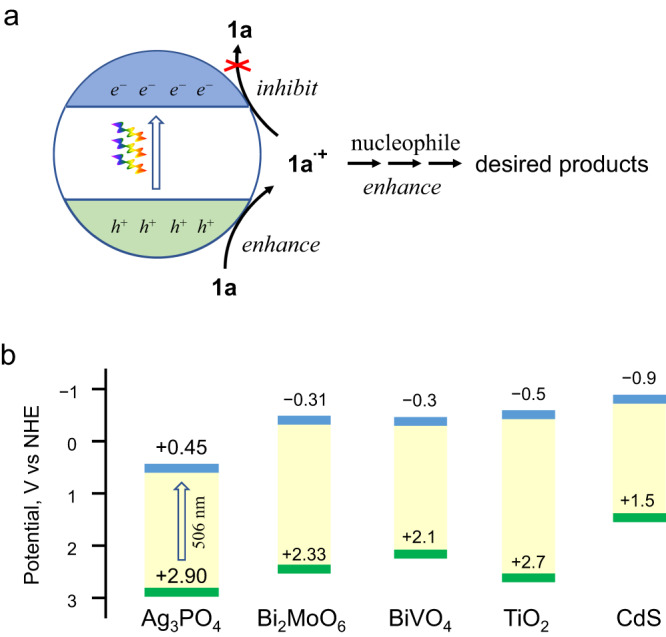
The selection of an inorganic semiconductor for photocatalytic pericyclic reactions. **a** Requirements for using anethole radical cation (**1a**^**•+**^) in photocatalytic constructing functionalized molecules from anethole (**1a**). **b** The band positions of Ag_3_PO_4_ and commonly applied photocatalysts.

Aromatic alkene radical cations, which are one electron (1e)-oxidation intermediates of aromatic alkenes, play important roles in the synthesis of complex functionalized molecules and cyclic moieties, particularly the [2 + 2] and [4 + 2] pericyclic products. To generate and make use of aromatic alkene radical cations, extensive efforts have been devoted for a long time. Compared to single electron oxidants such as Ce^4+^^1^, Fe^3+^^2^, and hypervalent iodine reagents^3,4^, photocatalysts (PCs) generate highly oxidizing holes under sunlight irradiation and operate under mild conditions, making photocatalysis a green and sustainable strategy for radical cation-mediated reactions^5–16^. However, the PCs utilized for generating radical cations are primarily homogeneous organic compounds, particularly transition metal-coordination complexes^8–11^ and π-conjugated molecules^12–15^. Meanwhile, the scope of the approaches is constrained by the short lifetime of radical cations (on a μs scale^17–23^). In contrast, inorganic semiconductor PCs (isPCs), such as TiO_2_, CdS, Bi_2_MoO_6_, and Ag_3_PO_4_, have been widely employed for solar light harvesting applications, including water splitting, organic pollutant degradation, and photoelectric conversion^24^. From a practical standpoint, they are generally considered stable, recyclable, inexpensive, and environmentally friendly, making them an ideal choice for use in photosynthesis^25^. However, the efficiency of isPCs in the 1e-oxidative activation of non-polar and non-coordinative C = C moieties on their surfaces is typically low^17,20,26–33^. One obstacle is the short lifetime of holes (fs to ns^34^), which significantly slows down the 1e-oxidation of the C = C moieties (Fig. 1a). Moreover, even if some alkene radical cations are slowly generated at the surfaces of a traditional isPC like TiO_2_, they undergo few intermolecular C-C formation reactions^17^. Instead, they are more prone to nucleophilic attack by long-lived photogenerated electrons (*e*_CB_^−^), resulting in ineffective 1e-oxidation. These hindrances lead to a diminished quantum yield of light (i.e. reduced reaction rate) and low product yield, regardless of reaction time. Therefore, it is imperative to extend the lifespan of alkene radical cations for affordable and recyclable PCs that can effectively harvest sunlight for pericyclic reactions. However, this remains an ongoing challenge.

Among all the available isPCs, Ag_3_PO_4_ stands out as one of the few that exhibits an inherent visible-light response^35^. It has been extensively used in visible-light-driven water oxidation^35–37^ and organic pollutant degradation^37,38^. Compared to other commonly used semiconductors, it produces strong oxidizing holes and weak reducing electrons under visible-light irradiation (+2.9 V and +0.45 V *vs*. NHE; Fig. 1b). Moreover, the surfaces of Ag_3_PO_4_ are rich in large PO_4_^3−^ anions with high charge density that can strongly electrostatically interact with cationic species such as radical cations. As a result, photo-excited Ag_3_PO_4_ may efficiently generate but inefficiently quench radical cations, allowing for the accumulation of radical cations to facilitate radical cation-mediated reactions.

In this work, we demonstrate that Ag_3_PO_4_ efficiently catalyzes intramolecular and intermolecular [2 + 2] and Diels-Alder cycloadditions under visible light or solar irradiation. The system exhibits remarkable efficiency with respect to substrate scope, product yield, diastereoselectivity, apparent quantum yield (AQY), and scaleup synthesis under solar irradiation. Two critical aspects of the reaction mechanism are validated: (1) the existence of long-lived **1a**^**•+**^ radical cations on the surfaces of Ag_3_PO_4_ that are photo-reduced in situ, (2) the acceleration of rate-limiting step by prolonging the lifetime of **1a**^**•+**^. Our discoveries may pave the way for employing highly active organic radical cations and even radical anions in critical pericyclic processes.

## Results

### Transformation of aromatic alkene 1a to *anti*-cyclobutane 2a

**Fig. 2:**
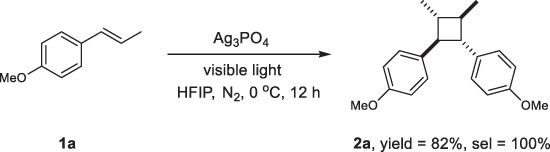
The model reaction of 1a. Conditions: **1a**, 1 mmol; Ag_3_PO_4_, (27 mg, 0.12 equiv); HFIP, 3.0 mL; LED (425 nm); 1 atm N_2_; 0 °C; 12 h.

**Fig. 3:**
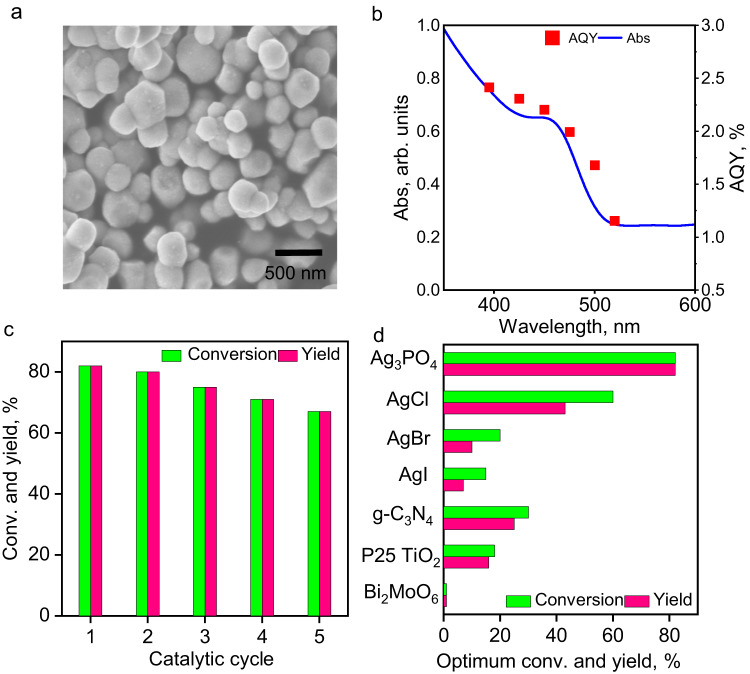
Characterization of Ag_3_PO_4_ and the yields of 2a under various conditions. **a** An SEM image of Ag_3_PO_4_. **b** The UV-vis diffuse-reflectance spectrum (smooth blue curve) and the AQY action spectrum (red diamond marks; see Supplementary Information for calculation method) of Ag_3_PO_4_. **c** The conversion of **1a** (green) and yield of **2a** (red) during reuse of Ag_3_PO_4_. **d** The conversion of **1a** (green) and yield of **2a** (red) by various photocatalysts. The unspecified conditions in panels **b**–**d**: **1a**, 0.5 mmol; catalyst, 0.12 equiv; HFIP, 1.5 mL; light source, 425 ± 10 nm LED (120 mW cm^−2^); N_2_ atmosphere; 0 °C; 12 h. In panel **d**, a 395 ± 10 nm LED lamp (119 mW cm^−2^) was used for TiO_2_. The reaction time was optimized for each photocatalyst, reaching up to 24 h for TiO_2_. The yields were determined by ^1^H NMR using 4-ethoxybenzaldehyde as the internal standard.

Anethole (**1a**) has been the most studied electron-rich *β*-substituted styrene in [2 + 2] cycloaddition reactions^3,19,39^. Therefore, it was selected as the model compound to evaluate the photocatalytic performance of Ag_3_PO_4_ (Fig. 2). A self-synthesized Ag_3_PO_4_ sample composed of nanospheres with a diameter of 230 ± 60 nm was used for the study (Fig. 3a). The cycloaddition reaction was conducted under an N_2_ atmosphere by irradiating a suspension of reactant **1a** in hexafluoroisopropanol (HFIP) solvent containing a catalytic quantity of Ag_3_PO_4_ (9.0 g L^−1^ dosage, 0.12 equiv) at 0 °C. No additive was introduced. The conversion of **1a** proceeded smoothly, affording *anti*-cyclobutane **2a** as the sole product (see Supplementary Fig. 1 for the time-resolved ^1^H NMR spectra and kinetics). After 12 h of reaction, the yield of **2a** reached 82%, with a high diastereoselectivity (*d.r*. > 19:1). Further irradiation did not affect conversion due to equilibration between **1a** and **2a** (Supplementary Fig. 2), as commonly observed in photocatalyzed radical cation processes^39,40^. Due to this equilibrium, the 82% yield was also achieved using various Ag_3_PO_4_ samples, including a commercially available Ag_3_PO_4_ and several self-synthesized faceted Ag_3_PO_4_ nanocrystal samples (see Supplementary Table 1 for screening of the experimental conditions). This implies that the catalyst is easily accessible.

The control experiments demonstrated that in the absence of Ag_3_PO_4_ or light, no conversion of **1a** occurred (Supplementary Table 1). Therefore, both light and Ag_3_PO_4_ are indispensable for the reaction, excluding the possibility of a thermocatalytic mechanism. Notably, the [2 + 2] cycloaddition reaction requires an inert atmosphere. Under air atmosphere, the conversion of **1a** could reach 100%, but 4-methoxybenzaldehyde was the main product (82% yield). The yield of the cycloaddition product **2a** was only 13%. This suggests that Ag_3_PO_4_ facilitates the selective oxidation of aromatic alkenes, resulting in the formation of the corresponding aromatic aldehydes, when O_2_ participates in the reaction process. We also performed the reaction in other solvents like tetrahydrofuran (THF), MeOH, EtOAc, and CF_3_CH_2_OH, but they were not as effective as HFIP. As shown in Fig. 3b, Ag_3_PO_4_ can effectively harvest visible light up to 500 nm to initiate the **1a** → **2a** cycloaddition, which is close to its absorption edge. The AQY values were determined according to the initial 30-min yields. The action spectrum of AQY matches the UV-vis diffuse-reflectance spectrum of Ag_3_PO_4_. The UV-vis absorption spectrum of **1a** shows it can only absorb light below 320 nm, and only Ag_3_PO_4_ can be excited by the light source (Supplementary Fig. 3). Although we were aware that depositing AgNPs on the surfaces of Ag_3_PO_4_ could enhance its photo-absorption and charge-separation efficiency^41^, our experiments using AgNPs-loaded samples with different AgNPs’ loadings afforded lower yields than pure Ag_3_PO_4_. Furthermore, as the loading of AgNPs increased, the final yield of **2a** decreased (Supplementary Figs. 4–6). This suggests that AgNPs are not the photocatalyst and can inhibit the activity of Ag_3_PO_4_. In all, it can be concluded that Ag_3_PO_4_ is the true photocatalyst, and all **2a** is the product of Ag_3_PO_4_ photocatalysis.

Silver salt-based photocatalysts often suffer from photocorrosion^42–45^. However, in the current system, the 0.12 equiv of initially added Ag_3_PO_4_ successfully worked five consecutive photocatalytic cycles with only a slight decrease in efficiency (Fig. 3c), resulting in a turnover number of 13. Photo-corrosion of Ag_3_PO_4_ was observed, as evidenced by its significant darkening after five cycles. The TEM image indicates the formation of numerous AgNPs on the surfaces of Ag_3_PO_4_ (Supplementary Fig. 7). Nevertheless, regeneration of the recycled sample was achieved through a simple immersion in 6.7 mM Na_2_HPO_4_ aqueous solution and the addition of a drop of 30% H_2_O_2_^46^. Within minutes, color, microscopic morphology, and photocatalytic performance were fully restored (Supplementary Fig. 7). As shown in Fig. 3d, when the other silver salts, such as AgCl, AgBr, and AgI, were used, the conversion of **1a** and the selectivity of **2a** were significantly lower than that of Ag_3_PO_4_. Both commercially available and self-synthesized AgCl, AgBr, and AgI decomposed rapidly upon irradiation (see Supplementary Fig. 8 for characterization). In comparison to the widely studied heterogeneous photocatalysts^47^, including graphitic carbon nitride (g-C_3_N_4_), Bi_2_MoO_6_, and TiO_2_, the performance of Ag_3_PO_4_ is superior. For example, the reaction rate was notably low over TiO_2_ even under UV. Furthermore, electron accumulation caused TiO_2_ to exhibit a blue hue and ultimately became completely inert once conversion reached 18% (Supplementary Fig. 9). When TiO_2_ was used under air atmosphere, the accumulated electrons can be efficiently quenched by O_2_. However, in this case the yield of **2a** was less than 10% due to the over-oxidization of **1a** to 4-methoxybenzaldehyde (67%). Supplementary Table 2 shows that Ag_3_PO_4_ performs comparably to state-of-the-art PCs and single-electron oxidants such as Ru(bpm)_3_(BArF)_2_^39^, PhI(OAc)_2_^3^ and Fe(ClO_4_)_3_ in the **1a** → **2a** cycloaddition^19^. Therefore, Ag_3_PO_4_ is highly applicable for [2 + 2] cyclobutanation reactions.

### Ag_3_PO_4_/visible light system for pericyclic reactions

**Fig. 4:**
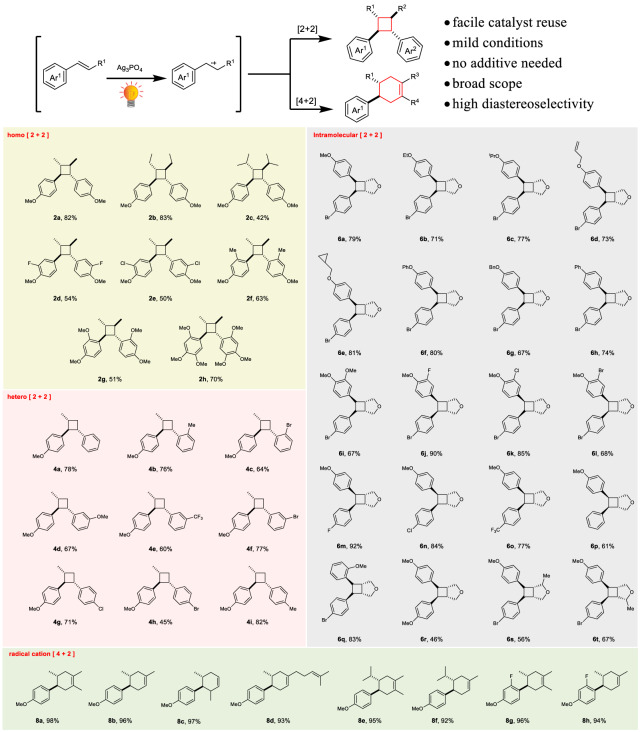
Scope of the Ag_3_PO_4_ photocatalysis in the radical cation pericyclic reactions. ^**a**^Conditions for homo [2 + 2] reactions: substrate, 1.0 mmol; Ag_3_PO_4_, 27 mg (0.12 equiv); 12–18 h. ^**b**^Conditions for hetero [2 + 2] reactions: **1a**, 0.5 mmol; the counterpart, 1.0 mmol; Ag_3_PO_4_, 44 mg (0.20 equiv); 12 h. ^**c**^Conditions for intramolecular [2 + 2] reactions: substrate, 0.3 mmol; Ag_3_PO_4_, 12 mg (0.10 equiv); 12 h. ^**d**^Conditions for [4 + 2] reactions: aromatic alkene, 1.0 mmol; diene, 2.0 mmol; Ag_3_PO_4_, 32 mg (0.08 equiv); 8 h.

Given the success of the [2 + 2] homo-cycloaddition of **1a**, we sought to explore whether the Ag_3_PO_4_/visible light system could serve as a versatile tool for achieving various pericyclic reactions. Our initial focus was on investigating the scope of the intermolecular dimerization reaction. As shown in Fig. 4, a diverse range of electron-rich aromatic alkenes with varying substituents underwent smooth reaction, affording the corresponding symmetrical cyclobutanes in moderate to good yields and excellent diastereoselectivity (**2a**-**2h**, yield ranges 42%-83%, *d.r*. > 19:1). Electronic density of the aromatic ring and steric hindrance at the *β*-site exert a noticeable influence on the reaction outcome (**2b** vs. **2c,** **2a** *vs*. **2d**-**2f**). However, this catalytic system exhibits high tolerance toward steric hindrance at the benzene ring (**2a** *vs*. **2f**-**2h**). Notably, NO_2_-, OH-, and COOH-substituted aromatic alkenes were challenging substrates for other [2 + 2] cycloaddition catalytic systems^48^, as well as the current Ag_3_PO_4_ system (Supplementary Table 3). Finally, we achieved a 70% isolated yield of magnosalin (**2h**), a valuable natural product. We then used the system to synthesize unsymmetric cyclobutanes and were pleased to discover that a variety of aromatic alkenes, including unsubstituted aromatic alkene (**4a**) and substituted aromatic alkenes with electron-donating groups (EDGs; **4b,** **4d, 4i**) and electron-withdrawing groups (EWGs; **4c, 4e, 4f, 4g, 4h**) at both ortho-, meta- and para-sites of the benzene rings, could be employed as model reaction counterparts using **1a**. The corresponding intermolecular [2 + 2] products (**2a**−**2h** and **4a**−**4i**) were obtained in high yields and high regioselectivity (head-to-head; see Supplementary Table 4 for ^1^H NMR analysis). The sole byproduct resulting from these crossed reactions is the homo-cycloaddition product, e.g., **2a**. However, because the crossed reactions are much faster than the homo-cycloaddition (see Supplementary Fig. 10 for a comparison of the rates), introducing **1a** via a syringe pump would inhibit the homo [2 + 2] reaction. Next, we explored the feasibility of intramolecular [2 + 2] cycloaddition, an efficient approach for synthesizing fused heterocycles^6^. Strikingly, all tested bis(styrene)s afforded good to excellent yields of the desired cyclobutanes (**6a**-**6t**). The intramolecular [2 + 2] cycloadditions exhibit a broad tolerance toward EDGs, EWGs, and the steric hindrance at both the benzene ring and the *β*-site. The Diels-Alder reaction is considered one of the essential C-C bond-forming reactions in synthetic organic chemistry. Recent studies have shown that photocatalytic methods are effective in the radical-cation-mediated cycloadditions of electron-rich olefins and dienes^32,49–52^, which are challenging substrates for conventional thermal processes. In this study, we found that even with a reduced Ag_3_PO_4_ loading of 0.08 equiv, the Ag_3_PO_4_/visible light system was still capable of facilitating Diels-Alder cycloadditions involving the radical cations. Moreover, the 0.08 equiv of Ag_3_PO_4_ was successfully reused for five consecutive runs without any noticeable decrease in performance (Supplementary Figure 11). Based on the substituted aromatic alkenes, the yields achieved in all tested reactions are near unity (**8a**-**8h**). The products **8b, 8c, 8d, 8f, and 8h** were isolated in high regioselectivity without isomers. This can be attributed to the large steric hindrance of the butadiene side chain (see Supplementary Table 4 for ^1^H NMR analysis).

The remarkable diastereoselectivity of the reactions is noteworthy. Specifically, nearly all intermolecular [2 + 2] products are anti; intramolecular [2 + 2] products are predominately syn, and nearly all Diels-Alder reactions yield anti-products.

### Scale synthesis of [2 + 2] and [4 + 2] reactions

**Fig. 5:**
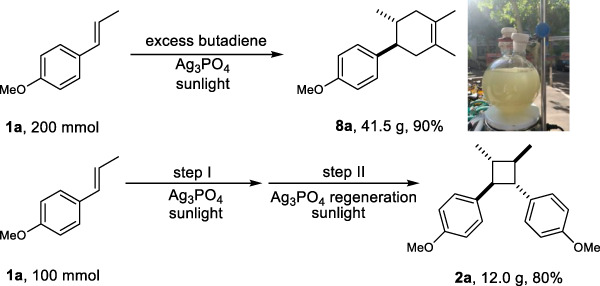
Scaleup synthesis using sunlight. Detailed reaction conditions were described in the Supplementary Information.

To investigate the synthetic potential of the Ag_3_PO_4_/visible light system, we performed large-scale [2 + 2] and [4 + 2] reactions under natural sunlight irradiation by placing the reaction flasks on a windowsill at outdoor temperatures (1–10 °C). The sunlight intensity ranged from 15−23 mW cm^−2^. Interestingly, 41.5 g of the [4 + 2] cycloaddition product **8a** was obtained almost quantitatively in a one-pot reaction after only six hours of irradiation (Fig. 5). After being filtered through diatomite, the Ag_3_PO_4_ solid was removed, resulting in nearly pure **8a** (41.5 g), which confirms the method’s flexibility and ease of use. The intermolecular [2 + 2] homo-cycloaddition of **1a** yielded a 60% yield of **2a** (100% selectivity) before Ag_3_PO_4_ (0.05 equiv) became deactivated. However, upon reintroduction of regenerated Ag_3_PO_4_ into the catalytic system, a final yield of 80% of **2a** was achieved (Fig. 5). The use of a 425 nm LED lamp achieved yields of **8a** that are comparable to that achieved by sunlight (Supplementary Table 1). Hence, the Ag_3_PO_4_/visible light system could facilitate actual industrial production considering the advances in LED technology.

Supplementary Tables 5–7 demonstrate the superior performance of the Ag_3_PO_4_/visible light system compared to the state-of-art reports in [2 + 2] and Diels-Alder cycloadditions. The current system boasts one of the highest efficiencies in heterogeneous systems, with broad light absorption. It is capable of realizing intramolecular and intermolecular [2 + 2] cycloadditions and Diels-Alder reactions.

### Mechanism study and DFT simulations

**Fig. 6:**
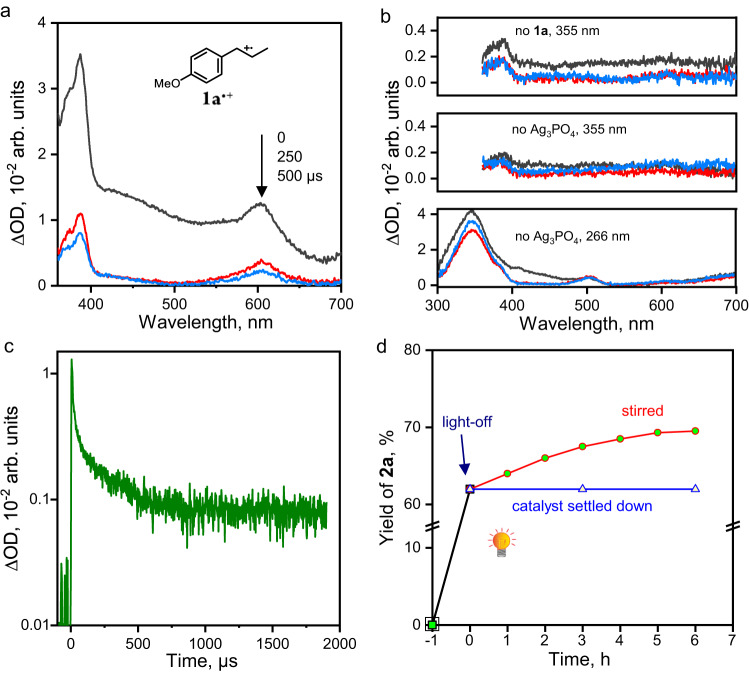
Detection of the transient species. **a** Transient absorption spectra of **1a**^**•+**^ obtained at various times after a 10-ns 355-nm pulse irradiation of the **1a**/Ag_3_PO_4_/HFIP system at room temperature under an air atmosphere. Before LFP, the suspension was ultrasonicated for 30 min to enhance the dispersion of Ag_3_PO_4_ in HFIP. **b** The transient absorption spectra obtained without **1a** or Ag_3_PO_4_: black, *t* = 0; red, 250 μs; blue, 500 μs. **c** The decay kinetics of **1a**^**•+**^ after 355-nm pulse irradiation. **d** The kinetics of **2a** formation in the light on-off experiment.

Laser flash photolysis (LFP) was performed to detect the transient species involved in the photocatalytic **1a** → **2a** cycloaddition. The LFP transient absorption spectra of the **1a**/Ag_3_PO_4_/HFIP suspension were obtained in transmission mode. The representative spectra are depicted in Fig. 6a. The two distinct, intense peaks centered at ca. 387 and 605 nm precisely match the spectra of **1a**^**•+**^ generated by photolysis of homogeneous solutions of **1a**/H_2_O-MeCN at 266 nm^53^ and **1a**/MeCN at 308 nm^54^, respectively, as reported in previous studies. Figure 6b demonstrates that the absence of **1a** or Ag_3_PO_4_ resulted in no signal from a 355-nm laser pulse, indicating the indispensability of both **1a** and Ag_3_PO_4_ for the formation of **1a**^**•+**^. Hence, the LFP spectra confirm that photogenerated *h*^+^ of Ag_3_PO_4_ facilitates the 1e-oxidation of **1a** to afford **1a**^**•+**^.

The concentration of a transient species, which is determined by both formation rate and lifetime, is essential for detecting it via LFP. Hence, the observation of **1a**^**•+**^ in the **1a**/Ag_3_PO_4_/HFIP system suggests that **1a**^**•+**^ forms rapidly and has a long life, allowing for its accumulation due to its formation far exceeding decay. Recall Fig. 6a, the sample initially exhibited strong background absorption, which significantly decreased after 250 μs due to the sedimentation of Ag_3_PO_4_ particles. The spectra at 250 and 500 μs showed similar level of background absorption, indicating that sedimentation was minor during this period. Based on this understanding, we measured the decay kinetics of **1a**^**•+**^ in the **1a**/Ag_3_PO_4_/HFIP suspension at *λ* = 600 nm. Figure 6c demonstrates that the signal intensity decayed significantly before 500 μs, which could be mostly attributable to the sedimentation mentioned above of Ag_3_PO_4_. Afterward, the signal intensity remained nearly constant until 1900 μs. This must not be attributed to the continuous generation of **1a**^•+^ by the photogenerated holes of Ag_3_PO_4_ after LFP, because it is well-known that the photogenerated holes in semiconductors are transient species. Instead, this indicates that the lifetime of **1a**^•+^ in the **1a**/Ag_3_PO_4_/HFIP system is more than 1900 μs.

We conducted light-on-off experiments to investigate the lifetime of **1a**^**•+**^ in the **1a**/Ag_3_PO_4_/HFIP system. The reaction vial was either left stirring in the dark after turning off the light or centrifuged to settle down the Ag_3_PO_4_ before being left in the dark. In the former situation, over the next six hours, there was a gradual increase of ca. 8% in yield of **2a** (Fig. 6d). This is distinct from the homogeneous systems, where the **1a** → **2a** cycloaddition practically ceased upon light-off^55^. In the dark reaction without agitation, the yield of **2a** remained unchanged after Ag_3_PO_4_ settled down (Fig. 6d). It indicates that the long-lived **1a**^•+^ radical cations must remain adsorbed on the surfaces of the reduced Ag_3_PO_4_ (denoted as (Ag_3_PO_4_)^*n***−**^, where *n* is the number of *e*_CB_^−^ per NP). When all the Ag_3_PO_4_ particles settled down, the **1a** molecules in solution could not reach **1a**^•+^, and thus the cycloaddition ceased. This verifies that the signals originate from **1a**^**•+**^ adsorbed on the Ag_3_PO_4_ surfaces rather than in the solution bulk. Further, it confirms that the radical cation-mediated [2 + 2] pericyclic reaction occurs on the surfaces of Ag_3_PO_4_ rather than in the solution.

It was reported that the decay rate constant^53,56^ of **1a**^**•+**^ is 4 × 10^4 ^s^−1^ in aerated MeCN, corresponding to a very short lifetime of 25 μs^54^. This is likely why the **1a** → **2a** cycloaddition stopped nearly instantly upon light-off in homogeneous systems^22,55^. To probe the lifetime of **1a**^**•+**^ in neat HFIP, we used a 266-nm laser pulse to excite a **1a**/HFIP solution. However, the system produced signals from unknown species and the absorption peaks of **1a**^**•+**^ at ca. 387 and 605 nm were not detected. This may suggest that the lifetime of **1a**^**•+**^ in HFIP is too short, resulting in a **1a**^**•+**^ concentration below the detection limit. We also used a 355-nm laser pulse to excite the **1a**/TiO_2_/HFIP system, which produced a 16% yield of **2a**, indicating that photo-excited TiO_2_ NPs generated **1a**^**•+**^. However, the transient spectrum of **1a**^**•+**^ was not detected by LFP (Supplementary Fig. 12), implying that its lifetime in the system is also very short.

**Fig. 7:**
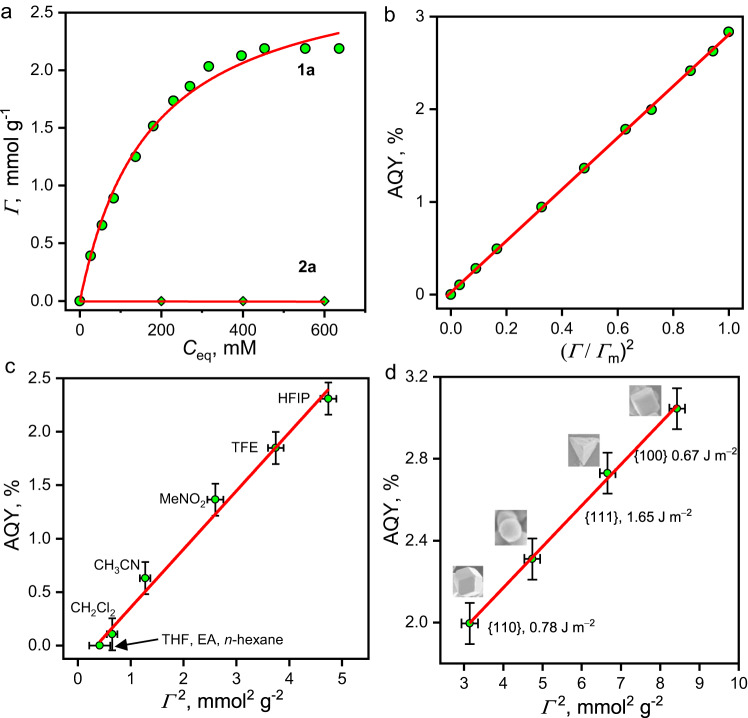
Dependence of AQY on adsorption. **a** The adsorption isotherm of **1a** and **2a** on spherical Ag_3_PO_4_. **b**–**d** The AQY values of the **1a** → **2a** cycloaddition reaction as functions of (*Г*/*Г*_m_)^2^ or *Г*^2^. The scattered points represent the experimental values, and the red lines are the curve fit or linear fits. Abbreviations for panel **c**: THF tetrahydrofuran, EA ethyl acetate, TFE trifluoroethanol. The SEM images, facets, and surface energies of Ag_3_PO_4_ NPs are indicated in panel d. The error bars in panels (**c**) and(**d**) associated with AQY represent the standard error of three sets of unique measurements.

We found that the adsorption of reactant **1a** and desorption of product **2a** are crucial in the reaction. Figure 7a shows that the adsorption of **1a** on the (100)-facet-rich spherical Ag_3_PO_4_ (as the representative example) can fit well to the Langmuir type-I isotherm (Eq. 1),

1 $$\varGamma={\varGamma }_{{{{{{\rm{m}}}}}}}\cdot {K}\cdot {{C}}_{eq}/(1+{K}\cdot {{C}}_{{eq}})$$

where *Г* is the adsorbed amount at the adsorption/desorption equilibrium concentration *C*_eq_. The Langmuir coefficient *K* and the capacity of adsorption *Г*_m_ are calculated to be 5.8 ± 0.6 M^−1^ and 2.9 ± 0.1 mmol g^−1^, respectively. In contrast, the adsorption of **2a** on Ag_3_PO_4_ surfaces is barely detectable, indicating very weak adsorption of **2a**.

At low **1a** concentrations, the AQY values increase dramatically with the initially added concentration of **1a** but then approach a plateau (Supplementary Fig. 13). This differs from a typical homogeneous bimolecular reaction, which is second order depending on the substrate concentration. Instead, the AQY values are well linearly correlated with the square of the fractional coverage of **1a,** *Ɵ* (Eq. 2),

2 $${{{{{\rm{AQY}}}}}}=k{(\varGamma {/}{\varGamma }_{{{{{{\rm{m}}}}}}})}^{2}=k\,{\theta }^{2}$$

where *k* denotes the slope of the plot (Fig. 7b). This kinetic equation corroborates that the conversion of **1a** through photocatalysis occurs on the Ag_3_PO_4_ surface. The rate-limiting step (RLS) of the formation of **2a** involves two molecular species of **1a** adsorbed on the surface. We also observe a significant solvent effect in the performance of Ag_3_PO_4_ in the **1a** → **2a** cycloaddition reaction (Fig. 7c). Ag_3_PO_4_ did not perform well in EtOAc, *n*-hexane or CH_2_Cl_2_, but performed better in CH_3_CN, MeNO_2_, and TFE, and performed best in HFIP. Surprisingly, Eq. 2 applies to the results from different solvents. Thus, solvents influence the AQY by modulating the **1a** distribution between the bulk solution and Ag_3_PO_4_ surfaces. It has been reported that the facets of photocatalysts substantially influence their activities, especially in the case of Ag_3_PO_4_^36,37,57,58^. For the **1a** → **2a** cycloaddition reaction, we used the spherical, rhombic dodecahedral, cubic, and tetrahedral NPs of Ag_3_PO_4_, which are rich in {100}, {110}, {100}, and {111} facets, respectively^36^. Figure 7d displays distinct AQYs on different facets and Eq. 2 is applicable to the results from various Ag_3_PO_4_ facets. Overall, Fig. 7 indicates that the photocatalytic cycloaddition reaction on Ag_3_PO_4_ surfaces follows the Langmuir-Hinshelwood mechanism for a bimolecular reaction. It also implies that the interaction between **1a** and Ag_3_PO_4_ surfaces is vital in the success of Ag_3_PO_4_ in the photocatalytic [2 + 2] cycloadditions.

**Fig. 8:**
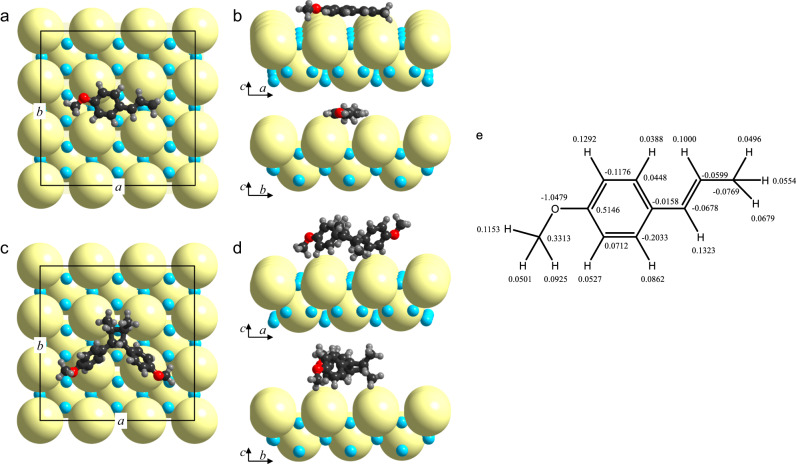
Configurations of adsorption and Bader charge. **a**–**d** The lowest-energy configurations of **1a** and **2a** molecules on the 2 × 2 region of the PO_4_^3−^-terminated (100) facet of Ag_3_PO_4_. **a** and **c** are the top views. **b** and **d** are the side views. The labels *a* and *b* denote the cell axes. The pale-yellow spheres represent PO_4_^3−^ (*r* = 2.38 Å), and the cyan spheres represent Ag^+^ (*r* = 0.67 Å). **1a** and **2a** molecules are drawn to scale. **e** The atomic Bader charges of adsorbed **1a**.

We conducted DFT simulations to determine the mechanism of interfacial interactions between **1a** (and **2a**) and the Ag_3_PO_4_ surfaces. Given the substantial dimensions of the Ag_3_PO_4_ particles and the comparable sizes of **1a** or **2a** molecules to only a few phosphate anions, it is reasonable to regard the surface of Ag_3_PO_4_ particles as an infinitely expansive plane for adsorbing **1a** and **2a**. As the (100) facet is the lowest-energy facet of Ag_3_PO_4_ crystals^36,37,58^ and the primary surface of spherical Ag_3_PO_4_, we selected the PO_4_^3−^-terminated and the Ag^+^-terminated (100) facets to calculate the optimal adsorption configurations (see Supplementary Figs. 14–15 for details of results). Figure 8 displays the lowest-energy configurations of **1a** and **2a** molecules on the PO_4_^3−^-terminated (100) facet. It should be noted that the size of PO_4_^3−^ is much larger than that of Ag^+^ (2.38 Å vs. 0.67 Å), but comparable to molecule **1a** in size. Molecule **1a** lies parallel to the facet, with its long axis aligned with the *a*-axis of the crystal lattice, and the CH = CH moiety positioned close to the O^2−^ ions of PO_4_^3−^. In this configuration, each **1a** molecule intimately contacts four PO_4_^3−^ anions, maximizing the interfacial interaction between **1a** and the Ag_3_PO_4_ surface. The strong interaction is consistent with the previously discussed adsorption of **1a** to the Ag_3_PO_4_ surfaces. This adsorption mode should be beneficial for the 1e-oxidation of the CH = CH moiety upon photo-excitation of Ag_3_PO_4_ since photogenerated holes are localized at O of PO_4_^3−^^59^. The energy required for adsorption of each **1a** molecule from the vacuum is −2.10 eV (Fig. 8a, b). In contrast, the calculated energies for **1a** adsorption onto PO_4_^3−^-terminated (100) facet along the *b*-axis and that onto the Ag^+^-terminated (100) facet along both the *a*- and *b*-axis, are substantially smaller at −1.67, −1.54, and −1.54 eV, respectively (Supplementary Fig. 14). These lower adsorption energies can be attributed to the greater spacing between the adjacent PO_4_^3−^ anions on these facets, which weakens the contact between **1a** and PO_4_^3−^. However, due to the large steric effects of the **2a** molecule, each **2a** has an optimum adsorption energy of −1.56 eV when adsorbed on the PO_4_^3−^-terminated (100) facet (Fig. 8c, d). The above calculations reveal a large adsorption energy difference between two **1a** and one **2a**, i.e. +2.64 eV on the PO_4_^3−^-terminated Ag_3_PO_4_ (100) facet in a vacuum. The much weaker adsorption of **2a** suggests that the **1a**→**2a** cycloaddition on the Ag_3_PO_4_ surfaces benefits from the easier removal of product **2a**.

The atomic Bader charges shown in Fig. 8e indicate that the electron density of **1a** changes upon adsorption. The overall Bader charge of adsorbed **1a** is 0.34e, indicating a transfer of 0.34e from **1a** to the Ag_3_PO_4_ surfaces during adsorption. The two H-atoms in the CH = CH moiety have enormous Bader charges, namely, 0.1323e and 0.1e, respectively. This, combined with the very negative Bader charge of the CH_3_O moiety ( − 0.4587e), strongly suggests that polarization occurs in molecule **1a** and that its CH = CH group is activated upon adsorption. The high Bader charge also means that the CH = CH group is the most readily oxidized site in **1a**.

**Fig. 9:**
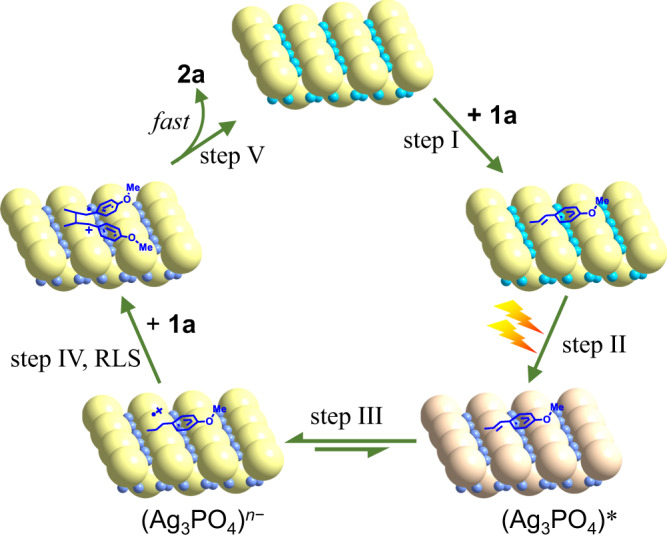
A plausible mechanism. (Ag_3_PO_4_)* denotes a photo-excited Ag_3_PO_4_ NP with many *e*_CB_^−^ and *h*^+^. The purple-blue spheres are used to show that in (Ag_3_PO_4_)* and (Ag_3_PO_4_)^*n***−**^, the *e*_CB_^−^ is shared by silver cations, resulting in a fraction charge of *δ* + (0 < *δ* < 1) on each cation.

Figure 9 illustrates a plausible mechanism for Ag_3_PO_4_ triggering the [2 + 2] cycloaddition of **1a** under visible-light irradiation. Initially, Ag_3_PO_4_ adsorbs **1a** molecules on its surface via PO_4_^3−^ anions (step I). Then, upon excitation, Ag_3_PO_4_ generates many *e*_CB_^−^ in the conduction band and *h*^+^ in the valance band (step II). Due to the fact that the conduction band bottom is mainly composed of hybridized Ag 5s5p orbitals^35^, the reduction power of the electrons in the conduction band of Ag_3_PO_4_ is low (*E*_CB_ = +0.45 V vs. NHE^35,41,60,61^). The unreactive *e*_CB_^−^ remains in the form of (Ag^δ+^)_m_^−^, which means it is shared by many Ag^+^ ions. The observed AgNP formation substantiates that *e*_CB_^−^ transfers to Ag^+^ ions. The valance band top of Ag_3_PO_4_ mainly comprises hybridized O 2p and Ag 4d orbitals. The *h*^+^ in the valence band has strong oxidation power (*E*_VB_ = +2.90 V vs. NHE^35,41,60,61^) and is able to gain an electron from the CH = CH group of **1a** to yield **1a**^**•+**^ (step III). The 1e-oxidation of **1a** results in the photocatalytically reduced NP with numerous *e*_CB_^**−**^. The **1a**^**•+**^ species strongly adsorbs on the surfaces of (Ag_3_PO_4_)^*n***−**^. In this case, the electrostatic interaction between **1a**^**•+**^ and (Ag_3_PO_4_)^*n***−**^ NP surfaces is analogous to the interactions in homogeneous solutions described by Yoon et al. for **1a**^**•+**^ with a tetraarylborate anion^18^, Ishihara et al. for **1a**^**•+**^ with FeCl_4_^−^^19–21^, and List et al. for **1a**^**•+**^ with an imidodiphosphorimidate counteranion^22^.

The adsorbed **1a**^**•+**^ is stabilized by electrostatic interaction with the (Ag_3_PO_4_)^*n***−**^ surfaces. The low possibility recombination between **1a**^**•+**^ and *e*_CB_^−^ shared by multiple Ag^+^ cations, (Ag^δ+^)_m_^−^, is evidenced by the observation of AgNP formation. As a result, the lifetime of **1a**^**•+**^ is dramatically prolonged to > 2 ms, which is over 75 times longer than in a homogeneous solution (μs level^54^). The long lifetime of **1a**^**•+**^ benefits the nucleophilic attack by another adsorbed **1a** molecule, allowing the reaction of **1a** + **1a**^**•+**^ **→ 2a**^**•+**^ in step IV to proceed (the RLS; see Supplementary Information for analysis).

The final step (step V) is the ring closure reaction of **2a**^•+^. The potential of **2a**^•+^/**2a** is approximately +1.5 V *vs*. NHE, much higher than the *E*_CB_ of Ag_3_PO_4_. Thermodynamically, the transfer of an electron from the reduced Ag_3_PO_4_ to **2a**^**•+**^ is an exothermic reaction. Step V may also occur through a radical chain mechanism^22^. Considering the potential of **1a**^•+^/**1a**, which is approximately +1.3 V *vs*. NHE, the ring closure of **2a**^•+^ by *e*_CB_^−^ exhibits a much larger driving force than that by the radical chain process.

The ability to generate the long-lived radical cation **1a**^**•+**^ distinguishes (Ag_3_PO_4_)^*n***−**^ from a traditional photocatalytically reduced TiO_2_ NP. On the illuminated TiO_2_ NP, the lifetime of **1a**^**•+**^ was too short to detect, and the conversion was only 18% (Fig. 3d). Okada et al. used a substantial excess of TiO_2_ (e.g., > 6 equiv) to minimize the accumulated electron per NP and extra LiClO_4_ (1.0 M) to stabilize the radical cations^18,22,23,25–27^. In contrast, Ag_3_PO_4_ photocatalysis can harvest the visible spectrum of sunlight and achieve a high yield of the desired product without any additives.

## Discussion

Ag_3_PO_4_ is a powerful photocatalyst for homo, crossed, intramolecular [2 + 2], and Diels-Alder [4 + 2] pericyclic reactions under visible-light irradiation. The catalytic process is mild, straightforward, affordable, additive-free, scalable under sunlight irradiation, and allows for easy product separation and catalyst reuse. It has a broad substrate scope and produces a wide range of desired products in modest to excellent yields. Our study reveals the potential of this photocatalytic process for fine chemical production. We have demonstrated that the rate-limiting step is the reaction between the reactant and its 1e-oxidation intermediate, a radical cation. The lifetime of anethole radical cation (**1a**^**•+**^) on the Ag_3_PO_4_ surfaces exceeds 2 ms, which is over 75 times longer than that in the homogeneous solutions, thus effectively promoting the rate-limiting step. The long lifetime of **1a**^**•+**^ is attributed to the appropriate band structure of Ag_3_PO_4_ and the strong electrostatic interaction between **1a**^**•+**^ and the (Ag_3_PO_4_)^*n***−**^ NP surfaces, which should be a general and essential mechanism for promoting chemical processes mediated by radical cations on heterogeneous surfaces. This may inspire ideas for more challenging radical cation/anion-mediated solar synthesis using inorganic semiconductor photocatalysts.

## Methods

### General procedure for the photocatalytic reactions

The reactions were carried out in 10-mL Pyrex vials. Olefin and Ag_3_PO_4_ were dispersed in a solvent in the vial, which was then purged for 10 min with high-purity N_2_ (99.999%). The vial was immersed in a mixture of ice and water to maintain the reaction temperature. The reaction suspension was stirred in the dark for 30 min to achieve adsorption-desorption equilibrium. The mixture was then exposed to the lamp from the side. To monitor the reaction progress, a syringe was used to withdraw 10 μL of the solution for analysis by thin-layer chromatography (TLC). After the reaction, the suspension was centrifuged to separate the solid catalyst from the solution. The residue is purified with column chromatography to afford the desired pure products.

### Details for the homo-dimerization of aromatic alkenes

To a solution of aromatic alkene **1** (1.0 mmol) in 3.0 mL of HFIP, Ag_3_PO_4_ (27 mg, 0.12 equiv) was added in one portion. The remaining steps are the same as the general procedure.

### Details for the cross-dimerization of aromatic alkenes

To a solution of aromatic alkene **3** (1.0 mmol, 2.0 equiv) in 2.0 mL of HFIP was added Ag_3_PO_4_ (44 mg, 0.20 equiv). Then, the reaction mixture was degassed by purging with high-purity nitrogen for 10 min. An ice bath was used to maintain the reaction temperature. The mixture was stirred in the dark at 0 °C for half an hour to achieve adsorption-desorption equilibrium. The photocatalytic reaction was then initiated by irradiating the dispersion from the side with the LED lamps. During the reaction, a solution of **1** (0.5 mmol) in 2.0 mL HFIP was added using a syringe pump (at a rate of 4.0 mL/h). The remaining steps are the same as the general procedure.

### Details for the intramolecular [2 + 2] reactions

To a solution of aromatic alkene **5** (0.3 mmol) in 2.0 mL of HFIP, Ag_3_PO_4_ (12 mg, 0.10 equiv) was added in one portion. The remaining steps are the same as the general procedure.

### Details for the Diels–Alder cycloadditions

To a solution of the diene **7** (2.0 mmol, 2.0 equiv) in 2.0 mL of HFIP was added Ag_3_PO_4_ (32 mg, 0.08 equiv). Then, the reaction mixture was degassed by purging with high-purity nitrogen for 10 min. An ice bath was used to maintain the reaction temperature. The mixture was then stirred in the dark at 0 °C for half an hour to achieve adsorption-desorption equilibrium. The photocatalytic reaction was initiated by irradiating the dispersion from the side with an LED lamp. During the reaction, a solution of **1** (1.0 mmol) in 2.0 mL of HFIP was added using a syringe pump (at a rate of 4.0 mL/h). The remaining steps are the same as the general procedure.

### Supplementary information

Supplementary Information

Peer Review File

### Source data

Source Data

## Data Availability

The authors declare that all relevant data supporting the findings of this study are available either within the manuscript itself and/or in the Supplementary Information. Experimental details and characterization of products are provided in the Supplementary Information. All other data are available from the corresponding author upon request. Source data are provided in this paper.

## References

[1] Nair V, Rajan R, Mohanan K, Sheeba V (2003). Cerium(iv) ammonium nitrate-mediated oxidative rearrangement of cyclobutanes and oxetanes. Tetrahedron Lett..

[2] Yu Y, Fu Y, Zhong F (2018). Benign catalysis with iron: facile assembly of cyclobutanes and cyclohexenes via intermolecular radical cation cycloadditions. Green Chem..

[3] Colomer I, Coura Barcelos R, Donohoe TJ (2016). Catalytic hypervalent iodine promoters lead to styrene dimerization and the formation of tri- and tetrasubstituted cyclobutanes. Angew. Chem. Int. Ed..

[4] Colomer I, Batchelor-McAuley C, Odell B, Donohoe TJ, Compton RG (2016). Hydrogen bonding to hexafluoroisopropanol controls the oxidative strength of hypervalent iodine reagents. J. Am. Chem. Soc..

[5] Liu X (2023). Unraveling the structure and reactivity patterns of the indole radical cation in regioselective electrochemical oxidative annulations. J. Am. Chem. Soc..

[6] Ischay MA, Lu Z, Yoon TP (2010). [2+2] cycloadditions by oxidative visible light photocatalysis. J. Am. Chem. Soc..

[7] Yoon TP, Ischay MA, Du J (2010). Visible light photocatalysis as a greener approach to photochemical synthesis. Nat. Chem..

[8] Jiang M, Yang H, Fu H (2016). Visible-light photoredox borylation of aryl halides and subsequent aerobic oxidative hydroxylation. Org. Lett..

[9] Tian YM (2020). Visible-light-induced Ni-catalyzed radical borylation of chloroarenes. J. Am. Chem. Soc..

[10] Tian YM (2018). Selective photocatalytic C-F borylation of polyfluoroarenes by Rh/Ni dual catalysis providing valuable fluorinated arylboronate esters. J. Am. Chem. Soc..

[11] Zhang L, Si X, Rominger F, Hashmi ASK (2020). Visible-light-induced radical carbo-cyclization/gem-diborylation through triplet energy transfer between a gold catalyst and aryl iodides. J. Am. Chem. Soc..

[12] Mazzarella D, Magagnano G, Schweitzer-Chaput B, Melchiorre P (2019). Photochemical organocatalytic borylation of alkyl chlorides, bromides, and sulfonates. ACS Catal..

[13] Zhang L, Jiao L (2019). Visible-light-induced organocatalytic borylation of aryl chlorides. J. Am. Chem. Soc..

[14] Chen J, Cen J, Xu X, Li X (2016). The application of heterogeneous visible light photocatalysts in organic synthesis. Catal. Sci. Technol..

[15] Jin S (2020). Visible light-induced borylation of C-O, C-N, and C-X bonds. J. Am. Chem. Soc..

[16] Al-Ekabi H, De Mayo P (1986). Surface photochemistry: the CdS photoinduced dimerization of n-vinylcarbazole. Tetrahedron.

[17] Liu Y, Zhang M, Tung C-H, Wang Y (2016). TiO_2_ photocatalytic cyclization reactions for the syntheses of aryltetralones. ACS Catal..

[18] Farney EP (2019). Discovery and elucidation of counteranion dependence in photoredox catalysis. J. Am. Chem. Soc..

[19] Horibe T, Ohmura S, Ishihara K (2019). Structure and reactivity of aromatic radical cations generated by FeCl_3_. J. Am. Chem. Soc..

[20] Okada Y (2020). Redox-neutral radical-cation reactions: Multiple carbon–carbon bond formations enabled by single-electron transfer. Electrochemistry.

[21] Horiguchi G, Kamiya H, Okada Y (2020). Mechanistic studies on TiO_2_ photoelectrochemical radical cation [2 + 2] cycloadditions. J. Electrochem. Soc..

[22] Das S (2023). Asymmetric counteranion-directed photoredox catalysis. Science.

[23] Horibe T, Katagiri K, Ishihara K (2020). Radical-cation-induced crossed [2+2] cycloaddition of electron-deficient anetholes initiated by iron(iii) salt. Adv. Synth. Catal..

[24] Wang Y, Wei Y, Song W, Chen C, Zhao J (2019). Photocatalytic hydrodehalogenation for the removal of halogenated aromatic contaminants. ChemCatChem.

[25] Ghosh I (2019). Organic semiconductor photocatalyst can bifunctionalize arenes and heteroarenes. Science.

[26] Nakayama K, Kamiya H, Okada Y (2022). Radical cation Diels-Alder reactions of arylidene cycloalkanes. Beilstein J. Org. Chem..

[27] Adachi S, Horiguchi G, Kamiya H, Okada Y (2022). Photochemical radical cation cycloadditions of aryl vinyl ethers. Eur. J. Org. Chem..

[28] Okada Y (2019). “Snapshots” of intramolecular electron transfer in redox tag-guided [2 + 2] cycloadditions. J. Org. Chem..

[29] Okada Y, Maeta N, Nakayama K, Kamiya H (2018). TiO_2_ photocatalysis in aromatic “redox tag”-guided intermolecular formal [2 + 2] cycloadditions. J. Org. Chem..

[30] Horiguchi G, Okada Y (2022). Mechanistic understanding of electrocatalytic vinylcyclopropane rearrangement. Eur. J. Org. Chem..

[31] Maeta N, Kamiya H, Okada Y (2019). Probing intramolecular electron transfer in redox tag processes. Org. Lett..

[32] Nakayama K, Maeta N, Horiguchi G, Kamiya H, Okada Y (2019). Radical cation Diels–Alder reactions by TiO_2_ photocatalysis. Org. Lett..

[33] Maeta N, Kamiya H, Okada Y (2020). Radical-cation vinylcyclopropane rearrangements by TiO_2_ photocatalysis. J. Org. Chem..

[34] Linsebigler AL, Lu G, Yates JT (1995). Photocatalysis on TiO_2_ surfaces: Principles, mechanisms, and selected results. Chem. Rev..

[35] Yi Z (2010). An orthophosphate semiconductor with photooxidation properties under visible-light irradiation. Nat. Mater..

[36] Martin DJ, Umezawa N, Chen X, Ye J, Tang J (2013). Facet engineered Ag_3_PO_4_ for efficient water photooxidation. Energy Environ. Sci..

[37] Bi Y, Ouyang S, Umezawa N, Cao J, Ye J (2011). Facet effect of single-crystalline Ag_3_PO_4_ sub-microcrystals on photocatalytic properties. J. Am. Chem. Soc..

[38] Martin DJ (2015). Efficient visible driven photocatalyst, silver phosphate: performance, understanding and perspective. Chem. Soc. Rev..

[39] Ischay MA, Ament MS, Yoon TP (2012). Crossed intermolecular [2 + 2] cycloaddition of styrenes by visible light photocatalysis. Chem. Sci..

[40] Riener M, Nicewicz DA (2013). Synthesis of cyclobutane lignans via an organic single electron oxidant-electron relay system. Chem. Sci..

[41] Guo L, Cui E, Li H, Tung C-H, Wang Y (2021). Singlet oxygen- and hole-mediated selective oxidation of arylethylenes to aryltetralones by Ag/Ag_3_PO_4_ under visible light irradiation. ACS Sustainable Chem. Eng..

[42] Cui E (2021). Engaging Ag(0) single atoms in silver(i) salts-mediated C-B and C-S coupling under visible light irradiation. J. Catal..

[43] An C (2016). Plasmonic silver incorporated silver halides for efficient photocatalysis. J. Mater. Chem. A.

[44] Cortie MB, McDonagh AM (2011). Synthesis and optical properties of hybrid and alloy plasmonic nanoparticles. Chem. Rev..

[45] An C, Peng S, Sun Y (2010). Facile synthesis of sunlight-driven AgCl:Ag plasmonic nanophotocatalyst. Adv. Mater..

[46] Garg R, Mondal S, Sahoo L, Vinod CP, Gautam UK (2020). Nanocrystalline Ag_3_PO_4_ for sunlight- and ambient air-driven oxidation of amines: high photocatalytic efficiency and a facile catalyst regeneration strategy. ACS Appl. Mater. Interfaces.

[47] Yang C (2020). Heterogeneous photoredox flow chemistry for the scalable organosynthesis of fine chemicals. Nat. Commun..

[48] Jiang Y, Wang C, Rogers CR, Kodaimati MS, Weiss EA (2019). Regio- and diastereoselective intermolecular [2+2] cycloadditions photocatalysed by quantum dots. Nat. Chem..

[49] Zhao Y, Antonietti M (2017). Visible-light-irradiated graphitic carbon nitride photocatalyzed Diels–Alder reactions with dioxygen as sustainable mediator for photoinduced electrons. Angew. Chem. Int. Ed..

[50] Lin S, Ischay MA, Fry CG, Yoon TP (2011). Radical cation Diels-Alder cycloadditions by visible light photocatalysis. J. Am. Chem. Soc..

[51] Pitre SP, Scaiano JC, Yoon TP (2017). Photocatalytic indole Diels-Alder cycloadditions mediated by heterogeneous platinum-modified titanium dioxide. ACS Catal..

[52] Pitre SP, Yoon TP, Scaiano JC (2017). Titanium dioxide visible light photocatalysis: Surface association enables photocatalysis with visible light irradiation. Chem. Commun..

[53] Johnston LJ, Schepp NP (1993). Reactivities of radical cations: characterization of styrene radical cations and measurements of their reactivity toward nucleophiles. J. Am. Chem. Soc..

[54] Schepp NP, Johnston LJ (1994). Reactivity of radical cations. Absolute kinetic data for cycloaddition reactions of styrene radical cations to alkenes. J. Am. Chem. Soc..

[55] Cismesia MA, Yoon TP (2015). Characterizing chain processes in visible light photoredox catalysis. Chem. Sci..

[56] Cozens FL (1997). Photochemical and thermal behavior of styrenes within acidic and nonacidic zeolites. Radical cation versus carbocation formation. J. Phys. Chem. B.

[57] Hsieh MS, Su HJ, Hsieh PL, Chiang YW, Huang MH (2017). Synthesis of Ag_3_PO_4_ crystals with tunable shapes for facet-dependent optical property, photocatalytic activity, and electrical conductivity examinations. ACS Appl. Mater. Interfaces.

[58] Ke J (2022). Facet-dependent electrooxidation of propylene into propylene oxide over Ag_3_PO_4_ crystals. Nat. Commun..

[59] Xiong Y (2020). Single-atom Rh/N-doped carbon electrocatalyst for formic acid oxidation. Nat. Nanotechnol..

[60] Hou Y (2012). Ag_3_PO_4_ oxygen evolution photocatalyst employing synergistic action of Ag/AgBr nanoparticles and graphene sheets. J. Phys. Chem. C.

[61] Bi Y, Ouyang S, Cao J, Ye J (2011). Facile synthesis of rhombic dodecahedral AgX/Ag_3_PO_4_ (X = Cl, Br, I) heterocrystals with enhanced photocatalytic properties and stabilities. Phys. Chem. Chem. Phys..

