# Supplementary material for: Ag3PO4 enables the generation of long-lived radical cations for visible light-driven [2 + 2] and [4 + 2] pericyclic reactions
Source: Nat Commun. 2024 Feb 1;15:979. doi: 10.1038/s41467-024-45217-y (PMC10834519; doi:10.1038/s41467-024-45217-y)
Supplement: Supplementary file 1 — Supplementary Information [file 41467_2024_45217_MOESM1_ESM.pdf]

Supplementary Information

Ag_3PO_4 Enables the Generation of Long-lived Radical Cations for Visible Light-Driven [2+2] and [4+2] Pericyclic Reactions

*Lirong Guo,[1] Rongchen Chu,[1] Xinyu Hao,[1] Yu Lei,[2] Haibin Li,[1] Dongge Ma,[3] Guo Wang,[4] Chen-Ho Tung[1] and Yifeng Wang[1]**

[1] Key Lab for Colloid and Interface Science of Ministry of Education, School of Chemistry and Chemical Engineering, Shandong University, Jinan 250100, China

[2] Key Laboratory of Photochemistry, Institute of Chemistry Chinese Academy of Sciences, Beijing National Laboratory for Molecular Sciences, Beijing 100190, China

[3] College of Chemistry and Materials Engineering, Beijing Technology and Business University, Beijing 100048, China

[4] Department of Chemistry, Capital Normal University, Beijing 100048, China

*Corresponding author email: yifeng@sdu.edu.cn

Contents:

1. Experimental details S2-S7

2. Additional results and discussion S8-S25

3. Analysis data of the substrates and products S26-S42

4. The spectra of the compounds S43-S174

5. References S175-S176

1. Experimental details

Materials. All reagents were of analytical grade and used without further purification unless otherwise stated. $AgNO_3$, Na_2HPO_4, Ag_3PO_4, AgCl, AgBr, and AgI were purchased from Energy Organics Co. Ltd. The commercially available Ag_3PO_4 was immersed in a 6.7 mM Na_2HPO_4 aqueous solution containing a drop of 30% H_2O_2, then washed with water and dried before use. TiO_2 (P25) and polyvinyl pyrrolidone (PVP, MW = 58,000) were purchased from Sigma-Aldrich Pharmaceutical Group Co. Ltd. If the solid raw chemicals agglomerated (such as AgCl), they were carefully ground into powders before use. Ammonium hydroxide, ascorbic acid, and hydrogen peroxide were purchased from Macklin Organics Co. Ltd. The commercially available aromatic alkenes were purchased from Sigma-Aldrich Pharmaceutical Group Co. Ltd. and used without purification. The anethole derivatives not commercially available were synthesized using Wittig reaction. All solvents, such as HFIP, nitromethane, dichloromethane, 2,2,2-trifluoroethanol, acetonitrile, tetrahydrofuran, *n*-hexane, and ethyl acetate, were dried by distillation with CaH as the desiccant.

Instruments. ^1H and ^{13}C NMR spectra were obtained on a Bruker AV-500 spectrometer with $CDCl_3$ as the solvent. High-resolution mass spectra were recorded on an Agilent Q-TOF 6510 instrument. A JEOL JSM-7000F SEM and a JEOL JEM–1011 TEM were used to investigate the morphology of the materials. The UV–vis diffusive reflectance spectra were recorded using the HITACHI U-3900 UV–vis spectrophotometer.

The emission spectra of all the LED lamps were reported previously. The light intensity irradiated on the reaction vial was measured using a light intensity meter (model CEL-NP2000-10; Beijing China Education Au-light Co., Ltd). The distance between the light intensity meter and the LED lamp was 8 cm. The light intensity values for the 395, 425, 450, 475, and 510 nm LED, and Xenon lamp were 119, 120, 112, 127, 122, and 300 mW cm^{-2}, respectively. The distance between the vials and the LED lamp was also 8 cm.[1]

Preparation of Ag_3PO_4. A home-prepared Ag_3PO_4 was chosen for most studies for the following reasons: (1) The synthesis is simple. (2) The recycled and regenerated samples can be easily compared with the as-prepared sample. It was prepared following the literature method. Briefly, a 100 mL aqueous

solution containing 717 mg of NaH_2PO_4 was added dropwise to a 100 mL solution containing PVP (800 mg) and $AgNO_3$ (1.7 g). The yellow suspension was heated at 60 °C for 30 mins under vigorous stirring. It was then filtered, rinsed with absolute alcohol and distilled water (Ag_3PO_4 is purified in this step), and dried under vacuum overnight to yield the Ag_3PO_4 spheres as a powder.[2, 3] According to our experience, spherical Ag_3PO_4 NPs of similar size could also be synthesized without using PVP. However, PVP was routinely used in the synthesis.

The tetrahedral, cubic, and rhomboid dodecahedral Ag_3PO_4 NPs were prepared following the literature procedures and characterized by SEM (shown later). The details are described as follows.

Ag_3PO_4 cubes. 89 mL of DI water was added to a beaker. Under magnetic stirring, 1 mL of NH_4NO_3 solution (0.40 M), 1.8 mL of NaOH solution (0.20 M), and 4.0 mL of $AgNO_3$ solution (0.05 M) were added to the beaker sequentially. Finally, 4.0 mL of K_2HPO_4 solution (0.10 M) was added to the beaker and stirred for 5 min. The as-obtained Ag_3PO_4 cubes were separated by centrifugation and washed three times with water.[4]

Ag_3PO_4 rhombic dodecahedra. Except for the feeding ratio of the reactants, the procedure for rhombic dodecahedra is similar to that of cubes. Specifically, 84 mL of DI water and 6.0 mL of NH_4NO_3 solution (0.40 M) were used. The other steps are the same as those of cubes.

Ag_3PO_4 tetrahedra. 3.0 mmol of $AgNO_3$ was dissolved in 30 mL of ethanol. 5.0 mL of H_3PO_4 was mixed with 30 mL of ethanol. Then, the $AgNO_3$-ethanol solution was added dropwise to the H_3PO_4-ethanol solution, until the mixture turned slightly cloudy. Finally, the mixture was added back into the $AgNO_3$-ethanol solution. The tetrahedral Ag_3PO_4 was separated by centrifugation and washed with ethanol three times.

Preparation of the other catalysts. Ag/Ag_3PO_4 was prepared by the photo-deposition method. Briefly, 1.0 g of Ag_3PO_4 powder was dispersed in a mixture of 10 mL of water, 2.0 mL of isopropanol, and 1.0 mL of $AgNO_3$ aqueous solution containing a certain amount of $AgNO_3$. The reaction vial was purged with high-purity N_2 for 10 min. The suspension was exposed to visible-light irradiation by a 300-W Xenon lamp with a 400 nm longpass filter under continuous stirring. After 2 hours of irradiation, the green precipitate was separated by centrifugation and washed twice with deionized water.

AgCl, AgBr, and AgI were prepared by the precipitation method. In a typical procedure, 5.0 mmol of sodium salt (NaCl, NaBr, and NaI) was dissolved in 100 mL of distilled water. This solution was added dropwise to 100 mL of $AgNO_3$ solution (0.10 M). The as-obtained precipitate was separated by centrifugation and washed with water three times.

g-C_3N_4 was prepared by pyrolysis of urea following the literature method.[5] Briefly, 13 g of urea was placed in an alumina crucible with a cover and heated at 600 °C for 4 h under air atmosphere. The product was washed with water five times until the supernatant became near neutral.

Bi_2MoO_6 was prepared by the precipitation method.[6] 1.6 g $Bi(NO_3)_3 \cdot 5H_2O$ and 0.42 g $Na_2MoO_4 \cdot 2H_2O$ were dissolved in 5.0 mL of ethylene glycol under magnetic stirring, respectively. The two solutions were mixed. Then, 20 mL of ethanol was slowly added into the above solution, followed by stirring for 10 min. The resulting clear solution was transferred into a 50 mL teflon-lined stainless steel autoclave, which was heated to 160 °C and maintained for 5 hours. Subsequently, the autoclave was cooled to room temperature naturally. The obtained samples were filtered, washed with ethanol, and dried at 80 °C in air.

Preparation of cyclization substrates. *E*-styrenes **1a-1h** were synthesized using Wittig reaction.[7] A solution of triphenylphosphonium halide (1.2 equiv.) in dry THF was placed in a flame-dried flask. The solution was cooled to -30 °C, and the base (1.2 equiv. of *n*-BuLi) was added dropwise. After stirring at 0 °C for 30 min, corresponding aldehyde (1.0 equiv.) was added. The reaction was gradually warmed to room temperature. After 12 h, the mixture was quenched by slow addition of saturated NH_4Cl. The aqueous phase was extracted twice with Et_2O. The combined organic layers were washed with brine, dried over Na_2SO_4. The solvent was evaporated under reduced pressure to give the corresponding styrene **2a-2h**, that was purified by chromatography on silica gel using 15:1 to 6:1 hexanes/Et_2O.

A 25 mL round-bottomed flask was added with NaH oil (9.8 mmol) and 2 mL THF under notrogen. Substrate **5a-5t** (6.1 mmol) was added dropwise in 6 mL of THF, and the reaction mixture was stirred vigorously for 15 min. The flask was cooled to 0 ℃, and (*E*)-phenyl-propenyl bromide (8.5 mmol) was added dropwise in 5 mL THF. The resulting mixture was warmed to room temperature. After 6 h, the reaction mixture was quenched by 10 mL saturated NH_4Cl. The aqueous phase was extracted two times

with ethyl acetate. The organic layers were washed with brine, dried over Na_2SO_4, and concentrated by rotary evaporation. Flash column chromatography (15:1 to 6:1 hexanes/Et_2O) afforded the title compound **6** as a white solid.[8]

Laser flash photolysis (LFP) experiments. A laser flash photolysis system (LP980, Edinburgh Instruments, U.K.) equipped with an Nd: YAG laser (Quanta Ray, Spectra-Physics, U.S.) was used for LFP experiments. The third harmonic mode (355 nm, laser duration of 10 ns, ~30 mJ/pulse) and the fourth harmonic mode (266 nm, laser duration of 10 ns, ~10 mJ/pulse) of the laser were employed as the pump source. A Xenon lamp (150 W) was used as the probe light. The pulsed laser output was directed onto a quartz cuvette at a right angle to the probe light.

Transmission mode was used for FLP transient absorption spectroscopy. All experiments were conducted at room temperature under an air atmosphere, as described in the literature.[9, 10] *E*-anethole (0.05 mmol) was added to a suspension of Ag_3PO_4 (10 mg) and HFIP (5.0 mL). The suspension was ultrasonicated for 30 min before the experiment. Because Ag_3PO_4 settled over time, all operations were completed quickly.

DFT calculations. The PBE density functional[11] and PAW basis[12, 13] in the Vienna ab initio Simulation Package (VASP)[14] were used for the DFT calculations. The DFT-D3 method with Becke-Jonson damping was used for weak interactions between molecules and substrates.[15, 16] A 3×3×3 supercell of Ag_3PO_4 was constructed following the literature method, and the lattice parameter of one direction was set to 35 Å for the adsorption of molecules.[4] The distance between image molecules in such a two-dimensional structure was set in the 10-15 Å to avoid possible interactions between them. Only Γ point was used for such a large supercell. Both PO_4^{3-}-terminated and Ag^+-terminated (100) facets, with various initial configurations of the adsorbed molecules on the facets, were used in the calculations. During geometric optimization, the bottom 2/3 of the two-dimensional supercell was kept fixed while the other atoms were relaxed. Several optimized structures are shown in Figures S14-S15.

Details for scale-up synthesis: the D-A cycloaddition. To a solution of the diene **7** (400 mmol, 2.0 equiv) in 350 mL of HFIP was added Ag_3PO_4 (4.2 g, 0.05 equiv). Then, the reaction mixture was degassed by purging with high-purity nitrogen for 10 min. The mixture was then stirred in the dark at 0

°C for 30 min to achieve adsorption-desorption equilibrium. During the reaction, a solution of styrene **1a** (200 mmol) in 150 mL of HFIP was added using a syringe pump (at a rate of 10 mL h^{-1}). The remaining steps are the same as the general procedure.

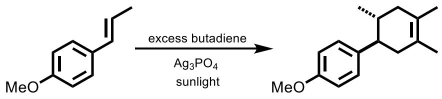

1a, 200 mmol **8a**, 41.5 g, 90%

Details for scale-up synthesis: the [2+2] cycloaddition of 1a. To a solution of styrene **1a** (100 mmol) in 300 mL of HFIP, Ag_3PO_4 (1.0 g, 0.05 equiv) was added in one portion. Then, the reaction mixture was degassed by purging with high-purity nitrogen for 10 min. An ice bath was used to maintain the reaction temperature. After completely deactivating the catalyst, the solid is filtered and washed with ethanol. Then, it was dispersed in 20 mL of Na_2HPO_4 aqueous solution (6.7 mM), followed by the dropwise addition of 1.0 mL of 30% H_2O_2 under magnetic stirring. The resulting catalyst was collected by centrifugation, washed several times with distilled water, and dried in an oven overnight. Finally, the regenerated Ag_3PO_4 was used again in the above mixture. The remaining steps are the same as the general procedure.

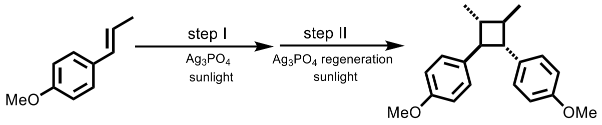

1a, 100 mmol **2a**, 12.0 g, 80%

Supplementary equation:

The apparent quantum efficiency (AQY) can be defined using the following equation **1**:

$$AQY = \frac{N_e}{N_p} \times 100\%$$
$$= \frac{10^9 \times (rate \times N_A \times K) \times (h \times c)}{I \times A \times \lambda} \qquad (1)$$

Where *Ne* is the number of electrons calculated according to the quantity of **2a**; *Np* is the total number of the incident photon. To get reliable data about AQY, we assume that all UV photons reaching the solution were absorbed. *Rate* is the reaction rate (mol s^{-1}); N_A is the Avogadro constant (6.022×10^{23} mol^{-1}); *K* is the electron number of the reaction; *h* is the Planck constant (6.626×10^{-34} J·s); *c* is the speed of light (3×10^{8} m s^{-1}); *I* is the intensity of irradiation light; *A* is the irradiation area (6.28 cm^2); λ is the wavelength of the monochromatic light (425 nm).

The AQY is then calculated using the following equation **2**.

$$AQY = \frac{1.2 \times 10^8 \times rate}{0.12 \times 6.28 \times 425} \qquad (2)$$

2. Additional results and discussion

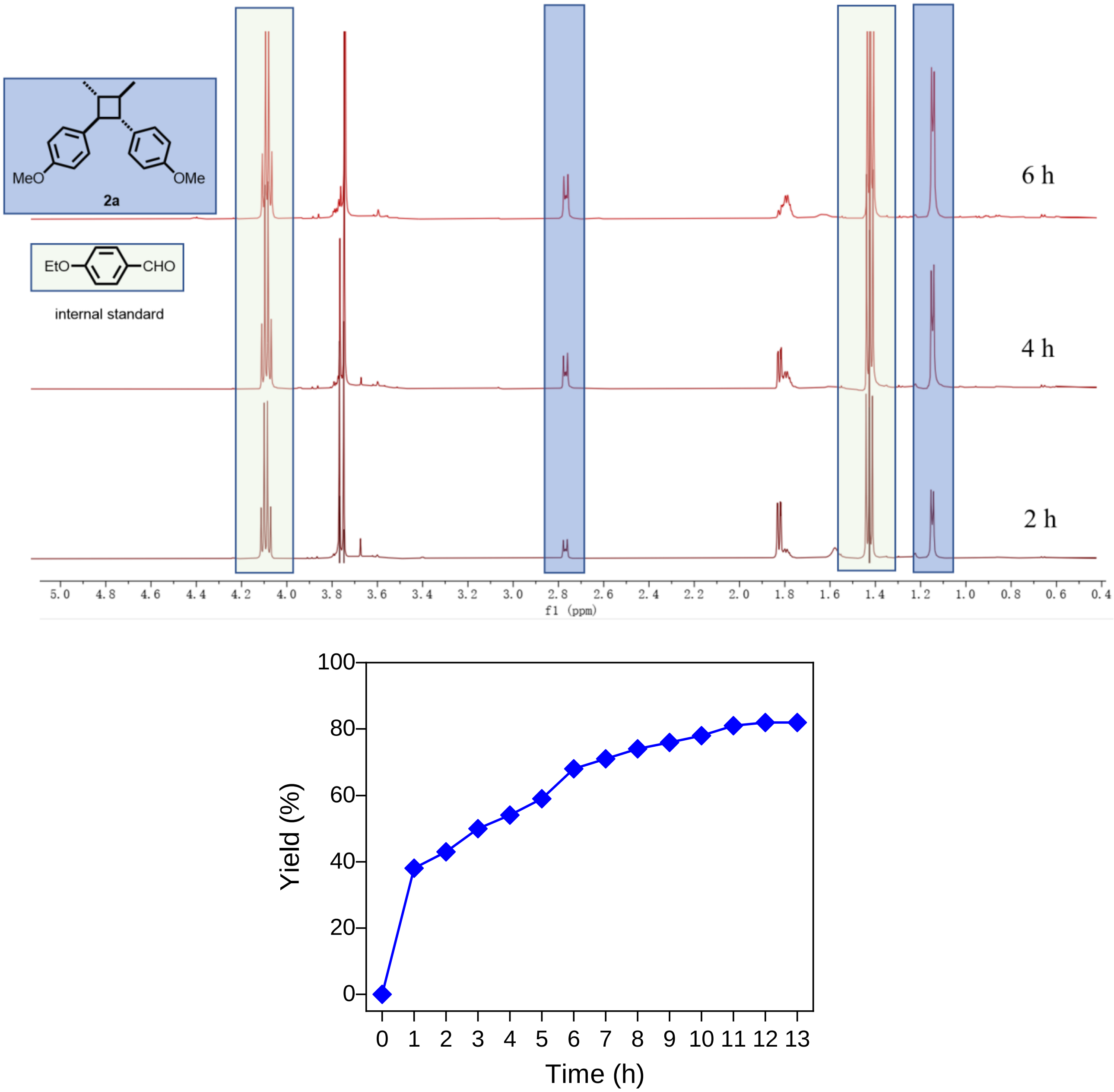

Supplementary Figure 1. Time-resolved ^{1}H NMR spectra and yield of **2a**.

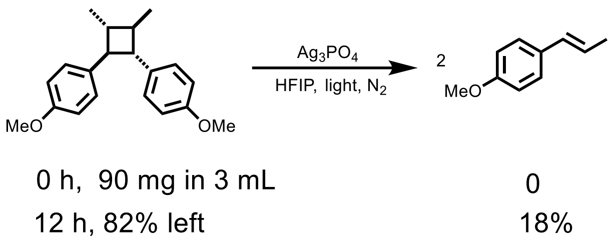

Supplementary Figure 2. The decomposition of **2a** by Ag_3PO_4 photocatalysis.

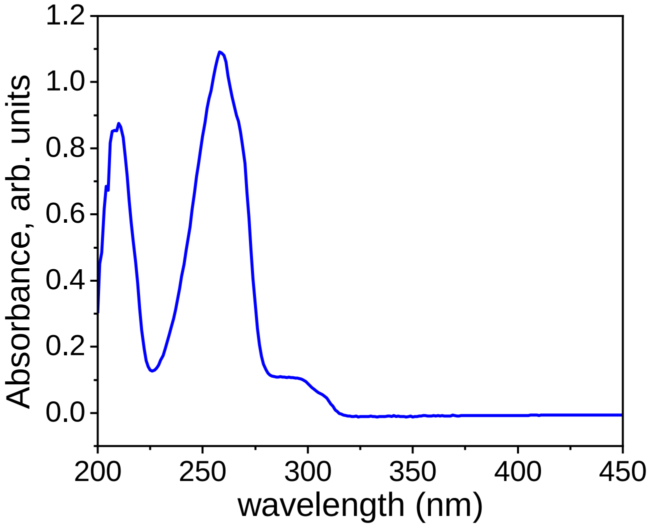

Supplementary Figure 3. The UV-Vis spectra of **1a** in MeCN.

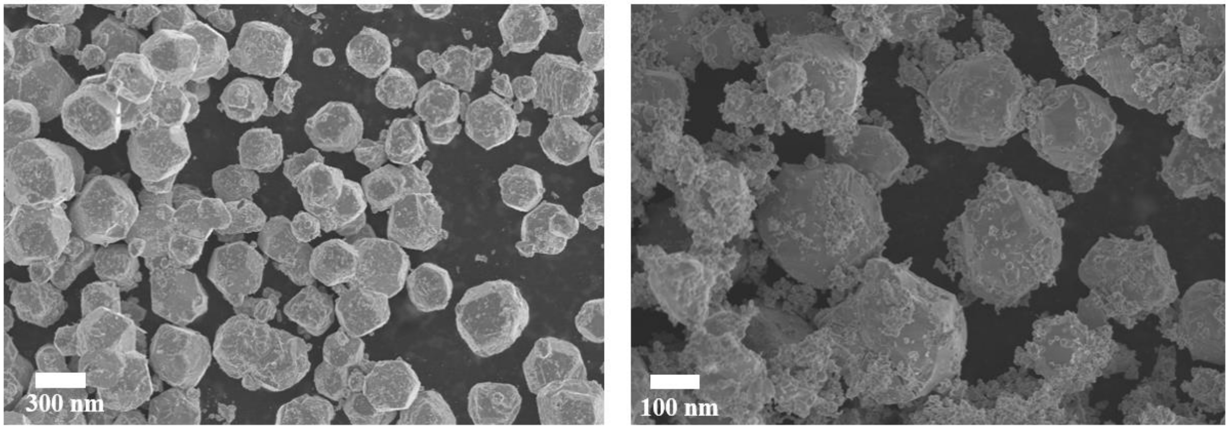

Supplementary Figure 4. SEM images of Ag/Ag_3PO_4-0.1 (left) and Ag/Ag_3PO_4-0.3 (right). The ratio of Ag and P in Ag/Ag_3PO_4-0.1 is 31.73 : 10.10, smaller than that in Ag/Ag_3PO_4-0.3 (33.51 : 10.18). This indicates Ag/Ag_3PO_4-0.3 contains more Ag NPs.

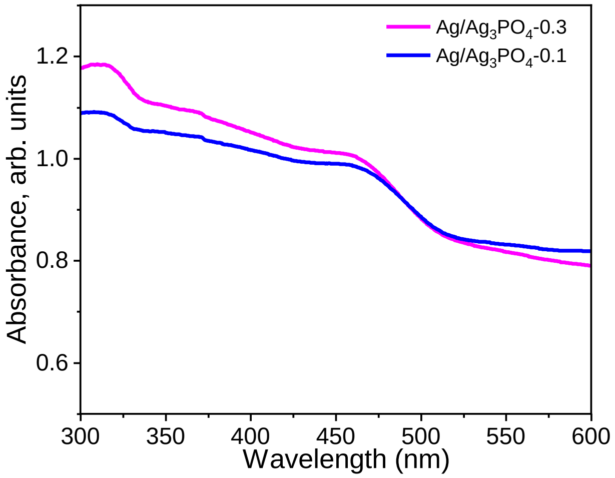

Supplementary Figure 5. The UV-Vis spectra of Ag/Ag_3PO_4-0.1 and Ag/Ag_3PO_4-0.3.

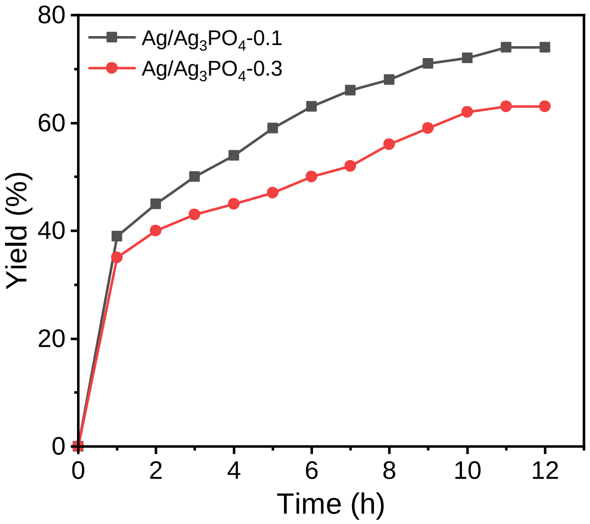

Supplementary Figure 6. Kinetics of reactions using Ag/Ag_3PO_4-0.1 and Ag/Ag_3PO_4-0.3.

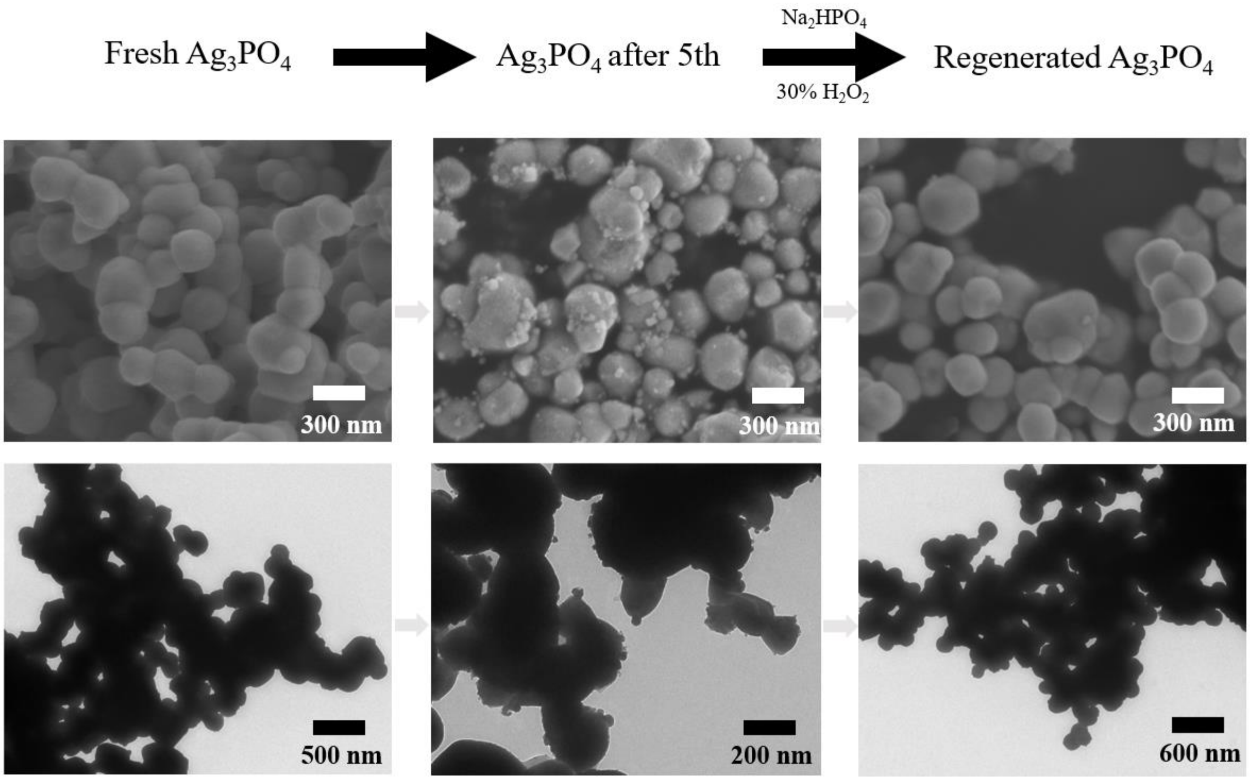

Supplementary Figure 7. The TEM and SEM images of fresh, recycled, and regenerated Ag_3PO_4.

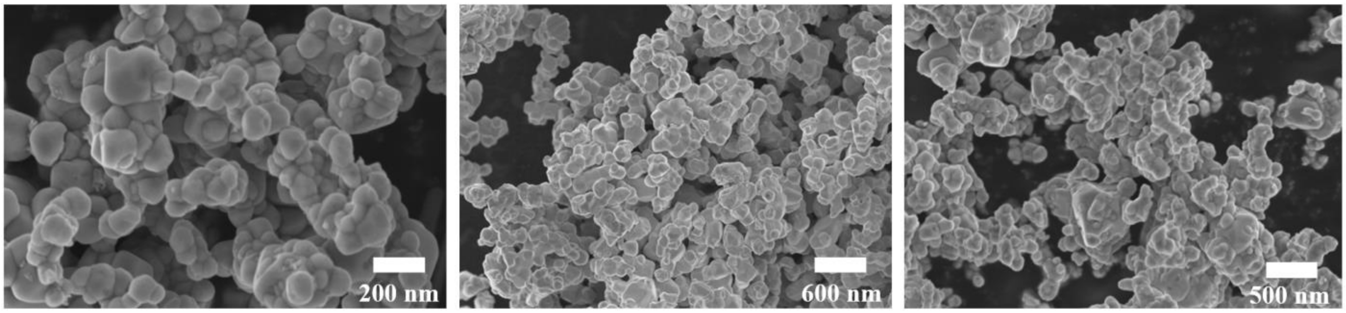

Supplementary Figure 8. The SEM images of self-synthesized AgCl (left), AgBr (middle), and AgI (right).

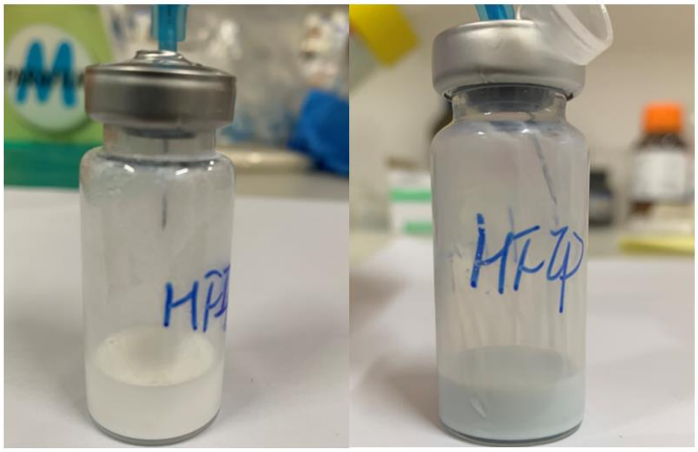

Supplementary Figure 9. The color change of TiO_2 before (left) and after irradiation (right).

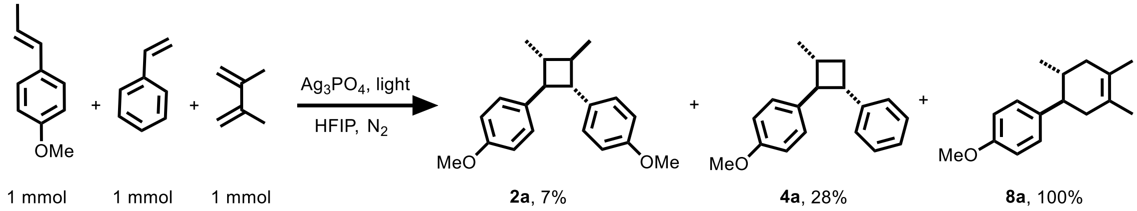

Supplementary Figure 10. Comparison of the reaction rates. Because the yields follow the order: **2a** << **4a** << **8a**, the rate of cycloaddition follows the order: homo [2+2] of **1** << crossed [2+2] of **1** and **3** << D-A cycloaddition of **1** and **7**.

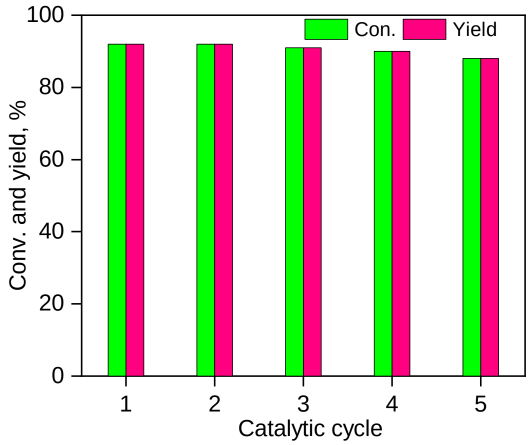

Supplementary Figure 11. Reuse of Ag_3PO_4 in the Diels-Alder cycloaddition.

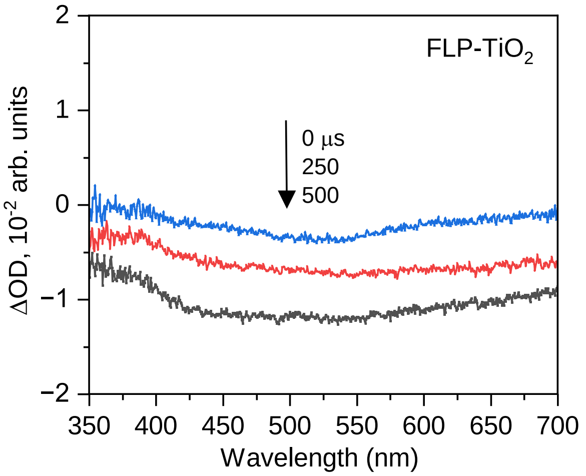

Supplementary Figure 12. Transient spectra (transmission mode) of the **1a**/TiO_2/HFIP system after 355 nm excitation. In this system, TiO_2 was stimulated by 355 nm light, producing 12% of **2a**. However, no **1a**$^{•+}$ was detected. This results from the low concentration of **1a**$^{•+}$, which is attributed to their limited lifespan.

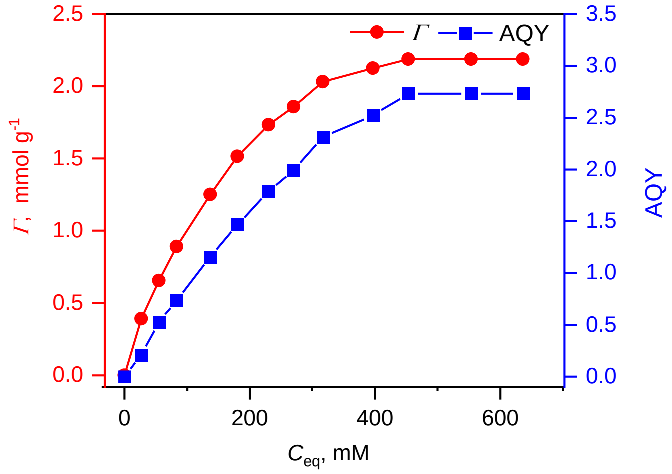

Supplementary Figure 13. The AQY as a function of substrate concentration.

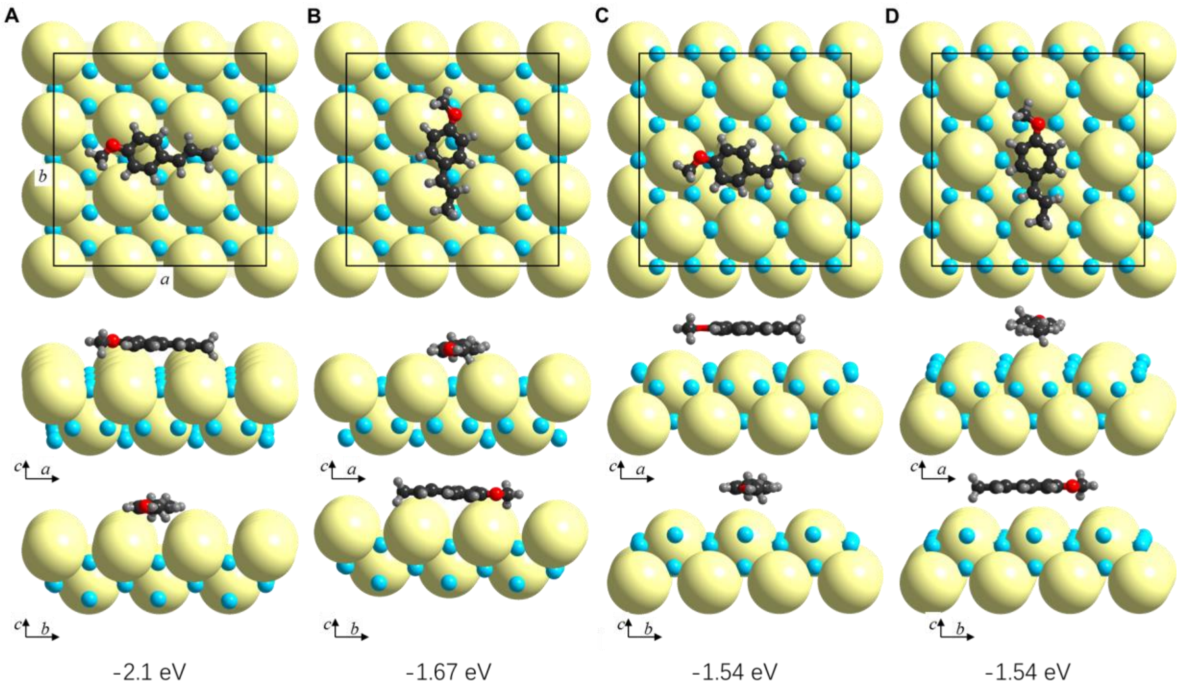

Supplementary Figure 14. The adsorption energy of **1a** from vacuum to Ag_3PO_4: (**A**) and (**B**), PO_4^{3-}-terminated surfaces; (**C**) and (**D**), Ag^+-terminated surfaces. The optimum configuration of **1a** is **A**, which is reported as Figure 5 in the main text.

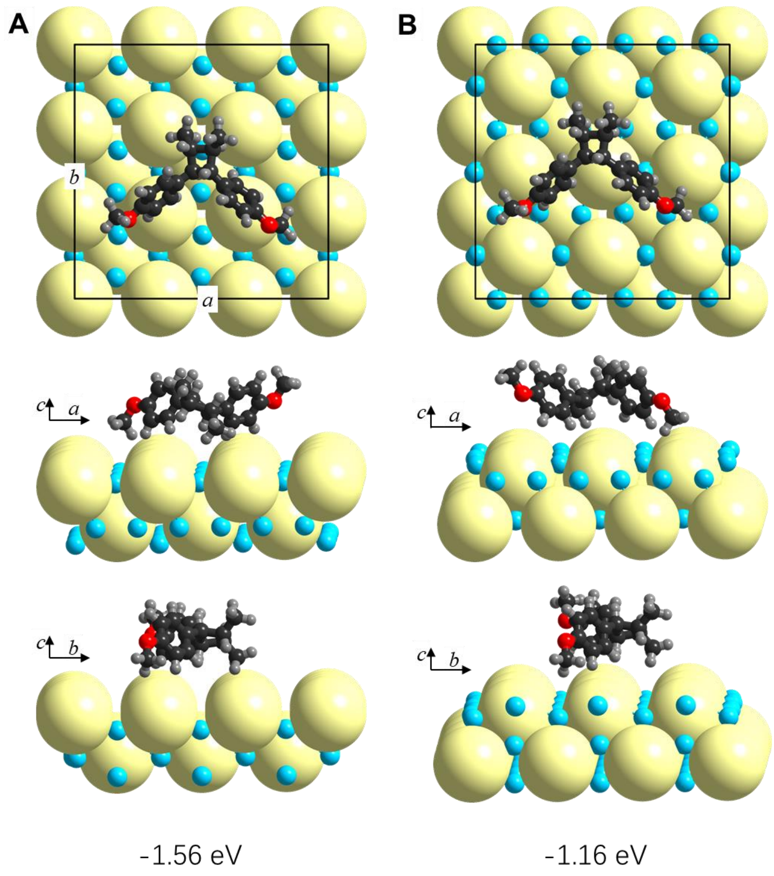

Supplementary Figure 15. The adsorption energy of **2a** from vacuum to Ag_3PO_4: (**A**) PO_4^{3-}-terminated surface and (**B**) Ag^+-terminated surface. The optimum configuration of **2a** is **A**, as shown in Figure 5 in the main text.

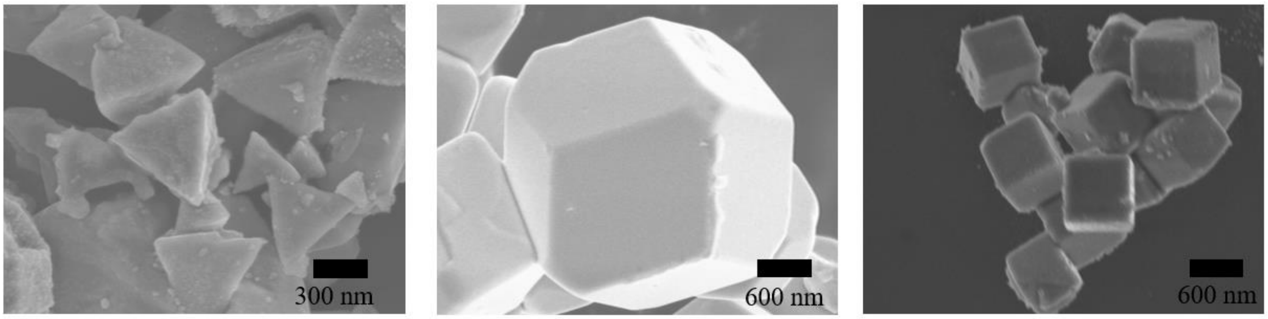

Supplementary Figure 16. The SEM images of the various faceted Ag_3PO_4 samples. The BET area values of all the samples are ranging 1-2 m^2 g^{-1} with uncertainties of ca. 0.5 m^2 g^{-1}.

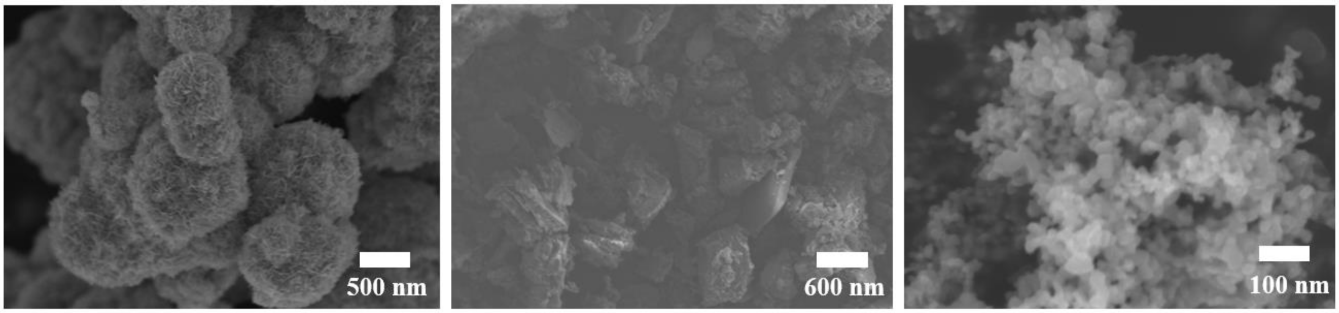

Supplementary Figure 17. The SEM images of self-synthesized Bi_2MoO_6 (left), C_3N_4 (middle), and commercial TiO_2 (right).

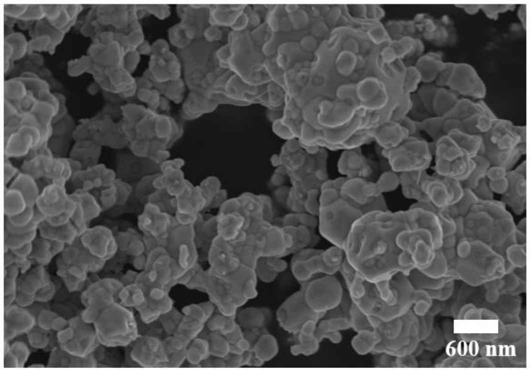

Supplementary Figure 18. SEM images of commercial Ag_3PO_4.

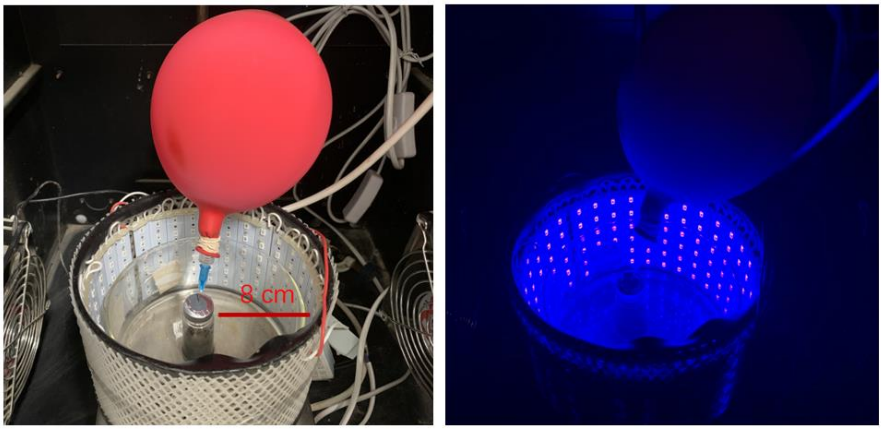

Supplementary Figure 19. The photos of the experimental setup.

Supplementary Table 1. Screening of reaction conditions.

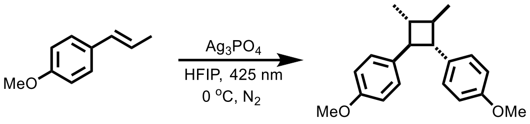

1a **2a**, 82(100)

Entry	Change from standard conditions	Conversion (%)	Yield (%)	Notes
1	no change	82	82	
2	no light, 0 ℃	0	N.R.	This indicates that light is essential for this reaction
3	no light, 80 ℃	0	N.R.	
4	no catalyst	0	N.R.	This indicates that a catalyst is essential for this reaction
5	commercial Ag_3PO_4	82	82	All Ag_3PO_4 samples can afford an 82% yield
6	self-synthesized rhombic dodecahedral Ag_3PO_4	82	82	
7	self-synthesized cubic Ag_3PO_4	82	82	
8	self-synthesized tetrahedral Ag_3PO_4	82	82	
9	air	100	13	In air atmosphere, Ag_3PO_4 leads to over-oxidation of **1a**
10	sunlight	81	81	
11	Xenon lamp	82	82	Both sunlight and Xenon lamps can effectively trigger the reaction
12	MeOH	0	N.R.	
13	CF_3CH_2OH	54	53	
14	THF	0	N.R.	
15	EtOAc	0	N.R.	HFIP is the best solvent
16	18 mg	73	72	
17	36 mg	82	82	27 mg is the optimal catalyst dosage

Standard condition: substrate, 1 mmol; photocatalyst, (27 mg, 0.12 equiv); solvent, 3.0 mL; LED (425 nm); 1 atm N_2; 0 °C; 12 h. The yield was determined by ^1H NMR analysis using 4-ethoxybenzaldehyde as an internal standard. N.R. = No Reaction.

Supplementary Table 2. Comparison of the performance of Ag_3PO_4 with various catalysts in the cycloadditions.

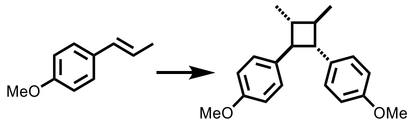

1a **2a**

catalyst	yield	conditions	catalyst reuse	gram-scale reaction	ref
Ag_3PO_4	1.0 mmol, 82%	12 mol%, 425 nm LED, HFIP, 0 °C	5 runs, > 64%	12.0 g, 80%	this study
triarylpyrylium-based polymer	0.1 mmol, 66%	2 mol% PC, 427 nm Kessil lamp, MeCN, r.t.	no	no	*ACS Catal.* **2020**, *10*, 13251.
cross-linked poly(benzothiadiazole) network	0.24 mmol, 80%	1 mg/mL PC, 460 nm LED, $MeNO_2$, r.t.	no	no	*ACS Catal.* **2017**, *7*, 3097.
2,4,6-tris(4-methoxyphenyl)pyrylium tetrafluoroborate	0.7 mmol, 54%	3 mol% PC, 450 nm LED. 50 mol% naphthalene as electron relays, MeCN, r.t.	no	no	*Chem. Sci.* **2013**, *4*, 2625.
$Ru(bpm)_3(BAr^F)_2$	1.38 mmol, 81%	0.5 mol% PC, fluorescent lightbulb, DCM, 0 °C	no	no	*Chem. Sci.* **2012**, *3*, 2807.
Cage-quinone	0.006 mmol, 34% in the NMR tube	5 mol% catalyst,CD_2Cl_2, r.t.	no	no	*J. Am. Chem. Soc.* **2020**, *142*, 2134.
phenyliodine diacetate	0.2 mmol, 76%	5 mol% catalyst, HFIPA, r.t.	no	no	*Angew. Chem., Int. Ed.* **2016**, *55*, 4748.
$Fe(ClO_4)_3$	1.0 mmol, 68%	10 mol% catalyst, MeCN 30 °C	no	no	*Green Chem.* **2018**, *20*, 1743.
fluorenone	0.5 mmol, 76%	3 mol% PC, blue LED, $MeNO_2$, r.t.	no	no	*Green Chem.* **2019,** *21*, 1916.

Supplementary Table 3. Challenging substrates in the [2+2] cycloaddition.

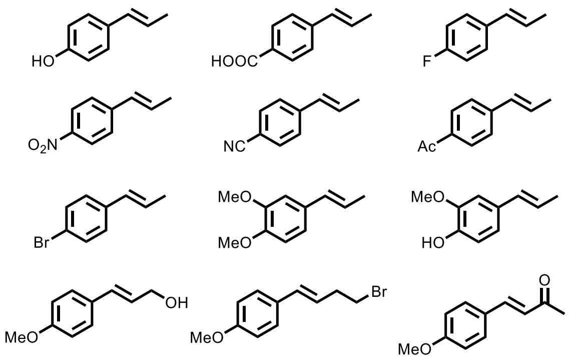

Supplementary Table 4. Comparison of the ^{1}H NMR spectra of **2a, 4a, 8b** with the literature report.[17, 18]

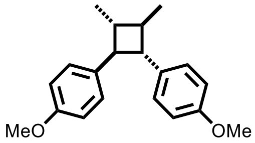

2a

group of chemical shift	^{1}H NMR of the synthetic sample (500 MHz, $CDCl_3$) δ	^{1}H NMR of reference (500 MHz, $CDCl_3$) δ
1	7.12 (d, *J* = 8.7 Hz, 4H),	7.12 (d, J = 8.6 Hz, 4H)
2	6.82 (d, *J* = 8.7 Hz, 4H)	6.82 (d, J = 8.6 Hz, 4H)
3	3.76 (s, 6H)	3.76 (s, 6H)
4	2.80 (d, 2H)	2.80 (d, 2H)
5	1.82 (m, 2H)	1.82 (m, 2H)
6	1.18 (d, *J* = 6.1 Hz, 6H)	1.18 (d, J = 6.1 Hz, 6H)

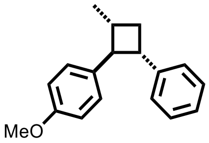

4a

group of chemical shift	^{1}H NMR of the synthetic sample (500 MHz, $CDCl_3$) δ	^{1}H NMR of reference (400 MHz, $CDCl_3$) δ
1	7.37 (t, *J* = 7.5 Hz, 2H)	7.40-7.33 (m, 2H)
2	7.27 (q, *J* = 7.9, 6.8 Hz, 5H)	7.31-7.25 (m, 5H)
3	6.95 (d, *J* = 8.6 Hz, 2H)	6.95 (d, *J* = 6.7Hz, 2H)
4	3.88 (s, 3H)	3.87 (s, 3H)
5	3.49 (q, *J* = 9.9 Hz, 1H)	3.54-3.43 (m, 1H)
6	3.05 (t, *J* = 9.5 Hz, 1H)	3.05 (t, *J* = 9.5 Hz, 1H)
7	2.68-2.56 (m, 1H)	2.66-2.55 (m, 1H)
8	2.43 (m , 1H)	2.44 (d, *J*= 7.3 Hz, 1H)
9	1.80 (q, *J* = 10.1 Hz, 1H)	1.80 (q, *J* = 10.1 Hz, 1H)
10	1.29 (d, *J* = 6.5 Hz, 3H)	1.29 (d, *J* = 6.5 Hz, 3H)

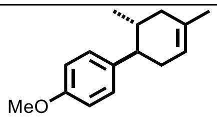

8b

group of chemical shift	^{1}H NMR of the synthetic sample (500 MHz, $CDCl_3$) δ	^{1}H NMR of reference (400 MHz, $CDCl_3$) δ
1	7.13 (d, *J* = 8.5 Hz, 2H)	7.18-7.05 (m, 2H)
2	6.89 (d, *J* = 8.5 Hz, 2H)	6.97-6.81 (m, 2H)
3	5.50 (s, 1H)	5.48 (s, 1H)
4	3.82 (s, 3H)	3.83 (s, 3H),
5	2.35 (td, *J* = 10.7, 5.3 Hz, 1H)	2.33 (dt, *J* = 10.4, 5.2 Hz, 1H)
6	2.30-2.09 (m, 3H)	2.26-2.08 (m, 3H)
7	2.00-1.91 (m, 1H)	1.93 (dt, *J* = 10.4, 5.1 Hz, 1H),
8	1.89-1.81 (m, 1H)	1.89-1.79 (m, 1H),
9	1.74 (s, 3H)	1.73 (s, 3H)
10	0.76 (d, *J* = 6.4 Hz, 3H)	0.75 (d, *J* = 5.9 Hz, 3H)

Supplementary Table 5. Comparison of the performance of Ag_3PO_4 with various catalysts in the cycloadditions.

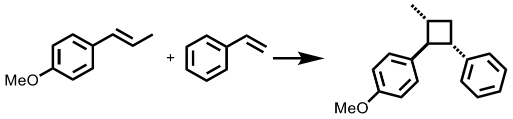

1a **3a** **4a**

**catalyst**	**yield**	**conditions**	**catalyst reuse**	**gram-scale reaction**	**ref**
Ag_3PO_4	1.0 mmol, 78%	20 mol%, 425 nm LED, HFIP, 0 °C	yes	no	this study
triarylpyrylium-based polymer	0.1 mmol, 44%	2 mol% PC, 427 nm Kessil lamp, MeCN, r.t.	no	no	*ACS Catal.* **2020**, *10*, 13251.
cross-linked poly(benzothiadiazole) network	0.24 mmol, 85%	1 mg/mL PC, 460 nm LED, $MeNO_2$, r.t.	no	no	*ACS Catal.* **2017,** *7*, 3097.
UCN-coating glass beads	10 mmol, flow chemistry equipment, 82%	1 mg/mL PC, white LED, $MeNO_2$,r.t.	5th, 80%	1.5 g, 82%	*Nat. Commun.* **2020,** *11*, 1239.

$Ru(bpm)_3(BArF)_2$	0.67 mmol, 79%	0.25 mol% PC, fluorescent lightbulb, DCM, -15 ^{o}C	no	1.45 g, 81%	*Chem. Sci.* **2012,** *3*, 2807.
Thioxanthylium-based catalysts	0.5 mmol, 99%	1 mol% PC, green LED,$MeNO_2$, r.t.	no	no	*Org. Lett.* **2020,** *22*, 5207.
$Fe(Me_4phen)_3(PF_6)_3$	0.1 mmol, 86%	3 mol% catalyst, DCE/TFE 4:1, r.t.	no	no	*Org. Lett.* **2018,** *20*, 5872.
Cage-Quinone	0.006 mmol, 56% in the NMR tube	5 mol% catalyst, CD_2Cl_2, r.t.	no	no	*J. Am. Chem. Soc.* **2020,** *142*, 2134.
Dess–Martin periodinane	1 mmol, 70%	10 mol% catalyst, HFIPA, r.t.	no	no	*Angew. Chem., Int. Ed.* **2016,** *55*, 4748.
$Fe(ClO_4)_3$	1.0 mmol, 85%	10 mol% catalyst, EtOAc, 40 ^{o}C	no	2.16 g, 85%	*Green Chem.* **2018,** *20*, 1743.

Supplementary Table 6. Comparison of the performance of Ag_3PO_4 with various catalysts in the cycloadditions.

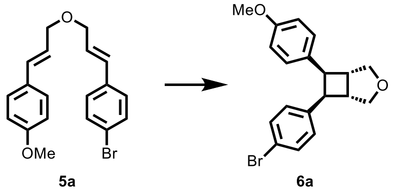

catalyst	yield	conditions	catalyst reuse	gram-scale reaction	ref
Ag_3PO_4	0.3 mmol, 79%	10 mol%， 425 nm LED, HFIP, 0 ^{o}C	yes	no	this study
$Ru(bpy)_3(PF_6)_2$	0.32 mmol, 92%	1 mol% PC, 15 mol % $MV(PF_6)_2$, visible light, $MeNO_2$, r.t.	no	1.0 g, 69%	*J. Am. Chem. Soc.* **2010,** *132*, 8572.
phenyliodine diacetate	0.0489 mmol, 75%	10 mol% catalyst, HFIP, r.t.	no	no	*Chem. Commun.* **2019,** *55*, 10316.

Supplementary Table 7. Comparison of the performance of Ag_3PO_4 with various catalysts in the cycloadditions.

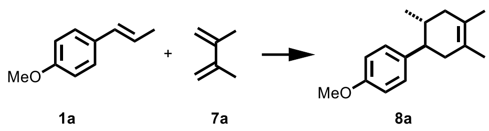

catalyst	yield	conditions	catalyst reuse	gram-scale reaction	ref
Ag_3PO_4	1.0 mmol, 98%	8 mol%, 425 nm LED, HFIP, 0 °C	5 runs, 90%	41.5 g, 90%	this study
g-C_3N_4	0.5 mmol, 97%	25 mg PC, 30 mg $MgSO_4$, >420 nm LED, $MeNO_2$, r.t.	5 th, 94%	1.0 g, 95%	*Angew. Chem., Int. Ed.* **2017,** *56*, 9336.
TiO_2	0.2 mmol, 97%	100 mg PC, 1 M $LiClO_4$,365 nm LED, $MeNO_2$, r.t.	no	no	*Org. Lett.* **2019,** *21*, 2246-2250.
$Ru(bpz)_3(BArF)_2$	0.68 mmol, 96%	0.5 mol% PC, CFL bulb, DCM, r.t.	no	no	*J. Am. Chem. Soc.* **2011,** *133*, 19350.
$[Cr(Ph_2phen)_3](BF_4)_3$	0.12 mmol, 86%	1 mol% PC, 300-419 nm, $MeNO_2$, r.t.	no	no	*Angew. Chem., Int. Ed.* **2015,** *54*, 6506.
Thioxanthylium-based catalysts	0.5 mmol, 95%	1 mol% PC, green light, $MeNO_2$, r.t.	no	1.5 g, 84%	*Tetrahedron Lett.* **2018,** 59, 3361.
$Fe(Me_4phen)_3(PF_6)_3$	0.1 mmol, 95%	1 mol% catalyst, DCE/TFE 9:1, r.t.	no	no	*Org. Lett.* **2018,** *20*, 5872.
Thioxanthylium-based catalysts	0.5 mmol, 88%	1 mol% PC, green light, $MeNO_2$, r.t.	no	1.5 g, 84%	*J. Org. Chem.* **2022,** *87*, 3319.
$[Cr(Ph_2phen)_3]_3BF_4$	0.12 mmol, 88%	1 mol% PC, 300-419 nm, $MeNO_2$, r.t.	no	no	*J. Am. Chem. Soc.* **2016,** *138*, 5451.
fluorenone	0.5 mmol, 74%	3 mol% PC, blue LED, $MeNO_2$, r.t.	no	no	*Green Chem.* **2019,** *21*, 1916.
$FeCl_3$	0.18 mmol, 98%	3 mol% catalyst, MeCN, r.t.	0	103.5 g, 95%	*J. Am. Chem. Soc.* **2019,** *141*, 1877.
$Fe(ClO_4)_3$	1.0 mmol, 68%	10 mol% catalyst, MeCN, r.t.	0	1.26 g, 97%	*Green Chem.* **2018,** *20*, 1743.
electrochemistry	1.6 mmol, 96%	$MeNO_2$, $LiClO_4$ electrolyte solution	0	0	*Chem. Sci.* **2016,** *7*, 6387.

Supplementary Table 8. Typical conversion and regioselectivity.

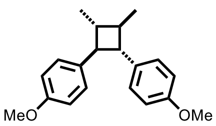

2a, conv. 82%, yield 82%

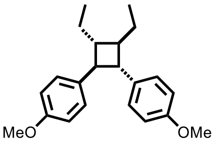

2b, conv. 83%, yield 83%

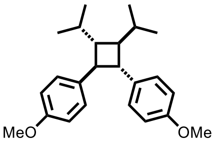

2c, conv. 42%, yield 42%

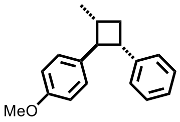

4a, conv. 86%, yield 78%

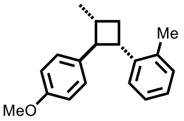

4b, conv. 85%, yield 76%

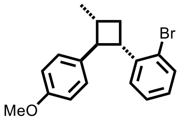

4c, conv. 74%, yield 64%

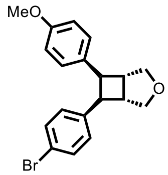

6a, conv. 79%, yield 79%

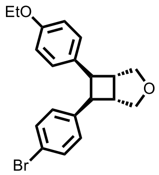

6b, conv. 71%, yield 71%

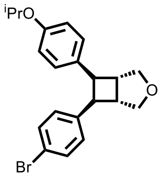

6c, conv. 77%, yield 77%

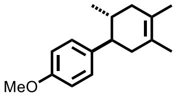

8a, conv. 100%, yield 98%

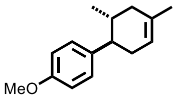

8b, conv. 100%, yield 96%

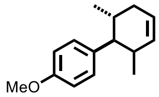

8c, conv. 100%, yield 97%

Discussion about the conversion and regioselectivity

For homo [2+2] cycloaddition and intramolecular [2+2] reactions, prolonged irradiation did not affect conversion due to equilibration between the subatrate and the product. As shown in the above Table 8, with the increase in steric hindrance of the substrate, the conversion of the reaction decreases obviously (**2a-2c**). For hetero [2+2] cycloaddition, a syringe pump was used to minimize the self-coupling of highly active **1.** In the crude mixture of **4a-4c**, we detected the self-coupling product of **1a.** For the [4+2] cycloaddition, little self-coupling product of **1a** was detected after 100% conversion.

There are two intermolecular [2+2] cycloaddition products: head-to-head and head-to-tail. They can be distinguished by ^{1}H NMR analysis. No head-to-tail products were generated in our study. There is only one product for intramolecular [2+2] cycloadditions, and there is no regioselectivity issue.

Theoretically, there are two products for the [4+2] cycloaddition between 2a and asymmetric butylenes 7. Namely, products **8b**, **8c**, **8d**, **8f**, and **8h** should have their own isomers. However, by ^{1}H NMR analysis, we found that these products were very pure, and their spectra were consistent with literature reports without any isomers, indicating that the regioselectivity should be 100%. This is because the groups possessing large steric hindrance on the butadiene side chain exhibit a tendency to position themselves

distantly from the p-methoxyphenyl group in the resultant [4+2] product, in a way of reducing steric hindrance.

More discussion about the LFP transient absorption spectra

The signals of **1a**$^{\bullet+}$ can be detected after 1900 μs by LFP spectra. This can be explained in two ways: (a) the long-lived signal is attributed to **1a**$^{\bullet+}$ located on the surface of Ag_3PO_4 NPs, or (b) the persistent signal of **1a**$^{\bullet+}$ is attributed to the **1a**$^{\bullet+}$ in the solution bulk which is slowly and continuously released from the surface of Ag_3PO_4.

However, explanation (b) can be ruled out for the following reasons. According to this assumption, the signal intensity should be equivalent to the desorption rate of **1a**$^{\bullet+}$. Because the signal of **1a**$^{\bullet+}$ almost no longer decays after 500 μs, the unchanged signal intensity indicates that the desorption rate is constant. This contradicts the widely accepted Langmuir desorption kinetics, which states that the desorption rate is proportional to the coverage. In addition, ultrasonic treatment can make Ag_3PO_4 highly dispersed in HFIP solvent, making the suspension appear quite homogeneous and transparent. This may be the main reason that the signal of **1a**$^{\bullet+}$ adsorbed on the surface of Ag_3PO_4 can be measured in transmission mode.

Unambiguously, neither explanation will affect one of the main conclusions of this article, namely, that **1a**$^{\bullet+}$ has an ultra-long lifetime on the Ag_3PO_4 surfaces.

Discussion on the rate-limiting step of the [2+2] cycloaddition by Ag_3PO_4 photocatalysis

First of all, assume substrate incoming (step I in Figure 9 of the text) is the rate-limiting step, then the overall rate should be equal to the rate of adsorption, namely,

$$\text{overall rate} = k_{ads} \bullet [\mathbf{1a}] \bullet (1-\theta) = k_{ads} \bullet [\mathbf{1a}] \bullet (1-\Gamma / \Gamma_m) \tag{3}$$

Next, assume the 1e-oxidation of **1a** (step III) is the rate-limiting step, then the overall rate should be determined by the amounts of both h^+ and adsorbed **1a**, namely,

$$\text{overall rate} = k_{\text{III}} \bullet [h^+] \bullet \Gamma \tag{4}$$

The above assumptions could be ruled out by the linear correlation of AQY with the square of the adsorbed amount of **1a** (recall eq 2 in the text)**.**

Finally, if the 1e-reduction of **2a**$^{\bullet+}$ (step V) is the rate-limiting step, the overall rate should be determined by the amounts of both e_{CB}^- and **2a**$^{\bullet+}$, namely,

$$\text{overall rate} = k_{\text{V}} \bullet [e_{\text{CB}}^-] \bullet [\mathbf{2a}^{\bullet+}] \tag{5}$$

Both the amounts of e_{CB}^- and **2a**$^{\bullet+}$ should not have direct connections with Γ. Therefore, step V should not be the rate-limiting step.

3. Analysis data of the substrates and products

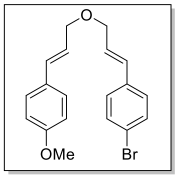

5a

1-bromo-4-((E)-3-(((E)-3-(4-methoxyphenyl)allyl)oxy)prop-1-en-1-yl)benzene (5a)

^{1}H NMR (500 MHz, $CDCl_3$) δ 7.43 (d, *J* = 8.4 Hz, 2H), 7.37 – 7.30 (m, 2H), 7.29 – 7.21 (m, 2H), 6.86 (t, *J* = 5.8 Hz, 2H), 6.58 (d, *J* = 15.9 Hz, 2H), 6.31 (dt, *J* = 15.9, 5.9 Hz, 1H), 6.19 (dt, *J* = 15.9, 6.3 Hz, 1H), 4.22 – 4.13 (m, 4H), 3.81 (s, 3H).

^{13}C NMR (126 MHz, $CDCl_3$) δ 159.35, 135.71, 132.46, 131.65, 131.09, 129.41, 127.99, 127.70, 127.00, 123.56, 121.40, 113.98, 71.14, 70.38, 55.28.

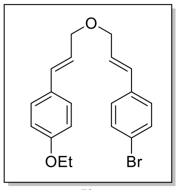

5b

1-bromo-4-((E)-3-(((E)-3-(4-ethoxyphenyl)allyl)oxy)prop-1-en-1-yl)benzene (5b)

^{1}H NMR (500 MHz, $CDCl_3$) δ 7.43 (d, *J* = 8.4 Hz, 2H), 7.32 (d, *J* = 8.6 Hz, 2H), 7.25 (d, *J* = 8.3 Hz, 2H), 6.84 (d, *J* = 8.6 Hz, 2H), 6.57 (d, *J* = 15.9 Hz, 2H), 6.31 (dt, *J* = 15.9, 5.9 Hz, 1H), 6.18 (dt, *J* = 15.8, 6.3 Hz, 1H), 4.26 – 4.13 (m, 4H), 4.03 (q, *J* = 7.0 Hz, 2H), 1.41 (t, *J* = 7.0 Hz, 3H).

^{13}C NMR (126 MHz, $CDCl_3$) δ 158.72, 135.72, 132.52, 131.64, 131.05, 129.25, 127.98, 127.68, 127.02, 123.43, 121.38, 114.53, 71.14, 70.34, 63.44, 14.80.

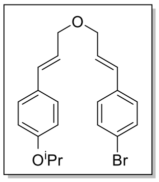

5c

1-bromo-4-((E)-3-(((E)-3-(4-isopropoxyphenyl)allyl)oxy)prop-1-en-1-yl)benzene (5c)

^{1}H NMR (500 MHz, $CDCl_3$) δ 7.48 – 7.38 (m, 2H), 7.37 – 7.29 (m, 2H), 7.25 (d, *J* = 8.1 Hz, 2H), 6.89 – 6.77 (m, 2H), 6.64 – 6.49 (m, 2H), 6.31 (dt, *J* = 15.9, 5.9 Hz, 1H), 6.17 (dt, *J* = 15.9, 6.3 Hz, 1H), 4.54 (dt, *J* = 12.1, 6.1 Hz, 1H), 4.24 – 4.10 (m, 4H), 1.56 (s, 1H), 1.33 (d, *J* = 6.1 Hz, 6H).

^{13}C NMR (126 MHz, $CDCl_3$) δ 157.70, 135.73, 132.56, 131.65, 131.06, 129.19, 127.99, 127.71, 127.04, 123.42, 121.39, 115.89, 71.16, 70.33, 69.92, 22.03.

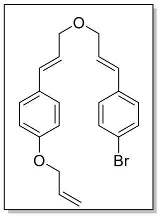

5d

1-(allyloxy)-4-((E)-3-(((E)-3-(4-bromophenyl)allyl)oxy)prop-1-en-1-yl)benzene (5d)

^{1}H NMR (500 MHz, $CDCl_3$) δ 7.48 – 7.40 (m, 2H), 7.37 – 7.29 (m, 2H), 7.28 – 7.22 (m, 3H), 6.94 – 6.77 (m, 2H), 6.57 (d, *J* = 15.9 Hz, 2H), 6.31 (dt, *J* = 15.9, 5.9 Hz, 1H), 6.19 (dt, *J* = 15.9, 6.3 Hz, 1H), 6.05 (ddd, *J* = 11.9, 10.5, 5.3 Hz, 1H), 5.41 (dq, *J* = 17.3, 1.6 Hz, 1H), 5.29 (dq, *J* = 10.5, 1.4 Hz, 1H), 4.54 (dt, *J* = 5.3, 1.5 Hz, 2H), 4.24 – 4.11 (m, 4H).

^{13}C NMR (126 MHz, $CDCl_3$) δ 158.33, 135.69, 133.15, 132.43, 131.64, 131.09, 129.54, 127.98, 127.67, 126.97, 123.62, 121.40, 117.73, 114.78, 71.12, 70.37, 68.80.

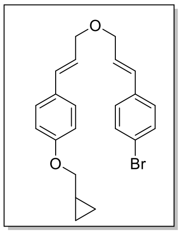

5e

1-bromo-4-((E)-3-(((E)-3-(4-(cyclopropylmethoxy)phenyl)allyl)oxy)prop-1-en-1-yl)benzene (5e)

^{1}H NMR (500 MHz, $CDCl_3$) δ 7.43 (d, *J* = 8.4 Hz, 2H), 7.32 (d, *J* = 8.7 Hz, 2H), 7.25 (d, *J* = 8.0 Hz, 3H), 6.85 (d, *J* = 8.7 Hz, 2H), 6.57 (d, *J* = 15.7 Hz, 2H), 6.32 (dd, *J* = 13.9, 8.0 Hz, 1H), 6.25 – 6.12 (m, 1H), 4.18 (d, *J* = 6.1 Hz, 4H), 3.80 (d, *J* = 6.9 Hz, 2H), 1.27 (ddd, *J* = 11.9, 7.5, 5.2 Hz, 1H), 0.73 – 0.58 (m, 2H), 0.35 (q, *J* = 4.8 Hz, 2H).

^{13}C NMR (126 MHz, $CDCl_3$) δ 158.79, 135.70, 132.52, 131.65, 131.09,

129.30, 127.99, 127.68, 126.98, 123.44, 121.40, 114.63, 72.79, 71.15, 70.37, 10.24, 3.17.

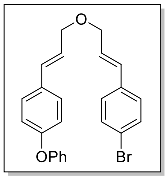

5f

1-bromo-4-((E)-3-(((E)-3-(4-phenoxyphenyl)allyl)oxy)prop-1-en-1-yl)benzene (5f)

^{1}H NMR (500 MHz, $CDCl_3$) δ 7.50 – 7.41 (m, 2H), 7.39 – 7.31 (m, 4H), 7.29 – 7.22 (m, 3H), 7.11 (t, *J* = 7.4 Hz, 1H), 7.04 – 6.99 (m, 2H), 6.98 – 6.92 (m, 2H), 6.65 – 6.53 (m, 2H), 6.36 – 6.29 (m, 1H), 6.24 (dt, *J* = 15.9, 6.1 Hz, 1H), 4.25 – 4.15 (m, 4H).

^{13}C NMR (126 MHz, $CDCl_3$) δ 157.02, 156.99, 135.68, 131.99, 131.84, 131.67, 131.15, 129.75, 127.99, 127.85, 126.92, 124.90, 123.39, 121.44, 118.98, 118.82, 70.97, 70.48.

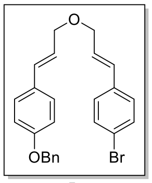

5g

1-(benzyloxy)-4-((E)-3-(((E)-3-(4-bromophenyl)allyl)oxy)prop-1-en-1-yl)benzene (5g)

^{1}H NMR (500 MHz, $CDCl_3$) δ 7.43 (d, *J* = 8.3 Hz, 4H), 7.38 (dd, *J* = 8.0, 6.8 Hz, 2H), 7.32 (dd, *J* = 7.4, 5.9 Hz, 3H), 7.29 – 7.21 (m, 2H), 6.93 (dd, *J* = 8.7, 1.7 Hz, 2H), 6.57 (d, *J* = 15.9 Hz, 2H), 6.31 (dtd, *J* = 15.9, 5.9, 1.4 Hz, 1H), 6.25 – 6.13 (m, 1H), 5.06 (s, 2H), 4.24 – 4.12 (m, 4H).

^{13}C NMR (126 MHz, $CDCl_3$) δ 158.55, 136.89, 135.71, 132.39, 131.65, 131.07, 129.67, 128.57, 127.98, 127.97, 127.71, 127.43, 127.00, 123.72, 121.40, 114.95, 71.11, 70.37, 70.03.

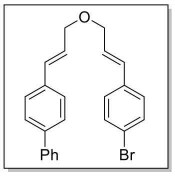

5h

4-((E)-3-(((E)-3-(4-bromophenyl)allyl)oxy)prop-1-en-1-yl)-1,1'-biphenyl (5h)

^{1}H NMR (500 MHz, $CDCl_3$) δ 7.58 (dd, *J* = 16.6, 7.8 Hz, 4H), 7.50 – 7.39 (m, 6H), 7.34 (t, *J* = 7.4 Hz, 1H), 7.26 (d, *J* = 8.2 Hz, 2H), 6.68 (d, *J* = 15.9 Hz, 1H), 6.59 (d, *J* = 16.0 Hz, 1H), 6.34 (ddt, *J* = 19.8, 15.9, 6.0 Hz, 2H), 4.28 – 4.11 (m, 4H).

^{13}C NMR (126 MHz, $CDCl_3$) δ 140.64, 140.50, 135.68, 132.21, 131.67, 131.19, 128.77, 128.00, 127.32, 127.25, 126.92, 125.99, 121.45, 70.94, 70.54.

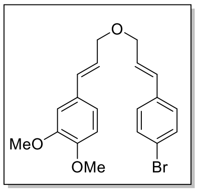

5i

4-((E)-3-(((E)-3-(4-bromophenyl)allyl)oxy)prop-1-en-1-yl)-1,2-dimethoxybenzene (5i)

^{1}H NMR (500 MHz, $CDCl_3$) δ 7.47 – 7.37 (m, 2H), 7.29 – 7.17 (m, 2H), 6.94 (dd, *J* = 17.9, 4.7 Hz, 2H), 6.85 – 6.76 (m, 1H), 6.57 (d, *J* = 15.9 Hz, 2H), 6.40 – 6.26 (m, 1H), 6.19 (dt, *J* = 15.8, 6.2 Hz, 1H), 4.18 (dd, *J* = 5.8, 4.6 Hz, 4H), 3.95 – 3.81 (m, 6H).

^{13}C NMR (126 MHz, $CDCl_3$) δ 149.08, 149.01, 135.72, 132.64, 131.68, 131.09, 129.77, 128.02, 127.02, 123.92, 121.44, 119.81, 111.17, 108.93, 71.09, 70.49, 55.93, 55.83.

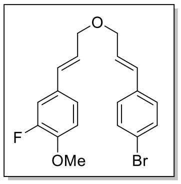

5j

4-((E)-3-(((E)-3-(4-bromophenyl)allyl)oxy)prop-1-en-1-yl)-2-fluoro-1-methoxybenzene (5j)

^{1}H NMR (500 MHz, $CDCl_3$) δ 7.48 – 7.40 (m, 2H), 7.26 (t, *J* = 4.1 Hz, 3H), 7.15 (dd, *J* = 12.5, 2.0 Hz, 1H), 7.07 (d, *J* = 8.5 Hz, 1H), 6.90 (t, *J* = 8.6 Hz, 1H), 6.56 (dd, *J* = 21.3, 16.0 Hz, 2H), 6.31 (dt, *J* = 15.9, 5.9 Hz, 1H), 6.18 (dt, *J* = 15.9, 6.1 Hz, 1H), 4.18 (d, *J* = 6.0 Hz, 4H), 3.89 (s, 3H).

^{13}C NMR (126 MHz, $CDCl_3$) δ 147.34, 135.66, 131.68, 131.29, 131.21, 128.00, 126.86, 125.09, 122.78, 121.47, 113.66, 113.51, 113.29, 70.79,

70.58, 56.30.

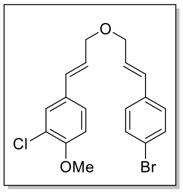

5k

4-((E)-3-(((E)-3-(4-bromophenyl)allyl)oxy)prop-1-en-1-yl)-2-chloro-1-methoxybenzene (5k)

^{1}H NMR (500 MHz, $CDCl_3$) δ 7.47 – 7.39 (m, 3H), 7.23 (dd, J = 13.7, 5.2 Hz, 3H), 6.87 (d, J = 8.5 Hz, 1H), 6.54 (dd, J = 26.7, 15.9 Hz, 2H), 6.30 (dt, J = 15.9, 5.9 Hz, 1H), 6.19 (dt, J = 15.9, 6.0 Hz, 1H), 4.22 – 4.14 (m, 4H), 3.89 (s, 3H).

^{13}C NMR (126 MHz, $CDCl_3$) δ 154.57, 135.69, 131.69, 131.19, 130.96, 130.45, 128.08, 128.02, 126.89, 126.03, 125.16, 122.70, 121.48, 112.03, 70.82, 70.58, 56.21.

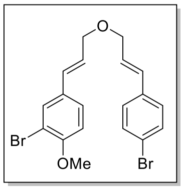

5l

2-bromo-4-((E)-3-(((E)-3-(4-bromophenyl)allyl)oxy)prop-1-en-1-yl)-1-methoxybenzene (5l)

^{1}H NMR (500 MHz, $CDCl_3$) δ 7.60 (d, J = 2.1 Hz, 1H), 7.48 – 7.37 (m, 2H), 7.33 – 7.21 (m, 4H), 6.85 (d, J = 8.5 Hz, 1H), 6.55 (dd, J = 29.2, 15.9 Hz, 2H), 6.31 (dt, J = 15.9, 5.9 Hz, 1H), 6.19 (dt, J = 15.9, 6.1 Hz, 1H), 4.18 (dd, J = 6.0, 1.4 Hz, 4H), 3.89 (s, 3H).

^{13}C NMR (126 MHz, $CDCl_3$) δ 155.45, 135.68, 131.69, 131.20, 130.93, 130.83, 128.02, 126.88, 126.74, 125.18, 121.48, 111.94, 111.85, 70.81, 70.57, 56.31.

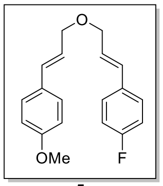

5m

1-fluoro-4-((E)-3-(((E)-3-(4-methoxyphenyl)allyl)oxy)prop-1-en-1-yl)benzene (5m)

^{1}H NMR (500 MHz, $CDCl_3$) δ 7.33 (ddt, J = 9.5, 7.1, 2.5 Hz, 4H), 7.03 – 6.95 (m, 2H), 6.89 – 6.81 (m, 2H), 6.58 (dd, J = 15.9, 6.7 Hz, 2H), 6.24 (dd, J = 14.1, 8.1 Hz, 1H), 6.20 – 6.13 (m, 1H), 4.20 – 4.14 (m, 4H), 3.78 (s, 3H).

^{13}C NMR (126 MHz, $CDCl_3$) δ 163.37, 161.41, 159.37, 132.97, 132.95, 132.44, 131.32, 129.48, 128.07, 128.00, 127.75, 125.90, 125.89, 123.67, 115.58, 115.41, 114.03, 71.08, 70.55, 55.28.

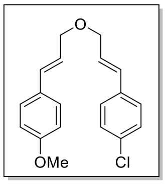

5n

1-chloro-4-((E)-3-(((E)-3-(4-methoxyphenyl)allyl)oxy)prop-1-en-1-yl)benzene (5n)

^{1}H NMR (500 MHz, $CDCl_3$) δ 7.39 – 7.23 (m, 6H), 6.92 – 6.82 (m, 2H), 6.58 (dd, J = 15.9, 5.6 Hz, 2H), 6.30 (dt, J = 15.9, 5.9 Hz, 1H), 6.19 (dt, J = 15.9, 6.3 Hz, 1H), 4.18 (d, J = 5.9 Hz, 4H), 3.81 (s, 3H).

^{13}C NMR (126 MHz, $CDCl_3$) δ 159.36, 135.28, 133.28, 132.49, 131.11, 129.43, 128.73, 127.73, 127.70, 126.85, 123.58, 114.00, 71.16, 70.43, 55.31.

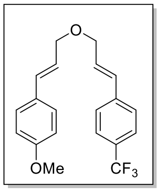

5o

1-methoxy-4-((E)-3-(((E)-3-(4-(trifluoromethyl)phenyl)allyl)oxy)prop-1-en-1-yl)benzene (5o)

^{1}H NMR (500 MHz, $CDCl_3$) δ 7.56 (d, J = 8.2 Hz, 2H), 7.48 (d, J = 8.2 Hz, 2H), 7.38 – 7.29 (m, 2H), 6.90 – 6.81 (m, 2H), 6.63 (dd, J = 44.0, 15.9 Hz, 2H), 6.42 (dt, J = 16.0, 5.7 Hz, 1H), 6.20 (dt, J = 15.9, 6.3 Hz, 1H), 4.21 (ddd, J = 9.5, 6.0, 1.3 Hz, 4H), 3.81 (s, 3H).

^{13}C NMR (126 MHz, $CDCl_3$) δ 159.39, 140.27, 132.60, 130.65, 129.55, 129.37, 129.29, 128.98, 127.74, 126.61, 125.55, 125.52, 125.28, 123.45, 114.01, 71.31, 70.21, 55.30.

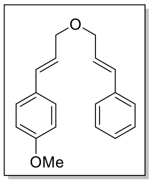

5p

1-((E)-3-(cinnamyloxy)prop-1-en-1-yl)-4-methoxybenzene(5p)

^{1}H NMR (500 MHz, $CDCl_3$) δ 7.39 (dd, *J* = 12.2, 4.8 Hz, 2H), 7.32 (dt, *J* = 13.7, 5.3 Hz, 4H), 7.23 (t, *J* = 7.3 Hz, 1H), 6.91 – 6.78 (m, 2H), 6.60 (dd, *J* = 27.7, 15.9 Hz, 2H), 6.32 (dt, *J* = 15.9, 6.1 Hz, 1H), 6.19 (dt, *J* = 15.9, 6.2 Hz, 1H), 4.23 – 4.16 (m, 4H), 3.79 (s, 3H).

^{13}C NMR (126 MHz, $CDCl_3$) δ 159.35, 136.78, 132.56, 132.41, 129.52, 128.63, 128.60, 127.75, 127.71, 126.54, 126.50, 126.14, 123.74, 114.02, 71.00, 70.66, 55.31.

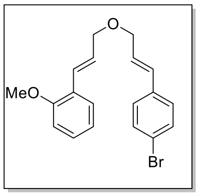

5q

1-((E)-3-(((E)-3-(4-bromophenyl)allyl)oxy)prop-1-en-1-yl)-2-methoxybenzene (5q)

^{1}H NMR (500 MHz, $CDCl_3$) δ 7.50 – 7.39 (m, 3H), 7.30 – 7.20 (m, 4H), 6.94 (dd, *J* = 18.1, 11.2 Hz, 2H), 6.87 (d, *J* = 8.2 Hz, 1H), 6.58 (d, *J* = 16.0 Hz, 1H), 6.33 (ddt, *J* = 15.9, 7.2, 6.1 Hz, 2H), 4.20 (ddd, *J* = 15.5, 6.1, 1.3 Hz, 4H), 3.85 (s, 3H).

^{13}C NMR (126 MHz, $CDCl_3$) δ 156.78, 135.77, 131.67, 131.10, 128.82, 128.02, 127.86, 127.09, 127.05, 126.49, 125.66, 121.39, 120.66, 110.84, 71.51, 70.36, 55.45.

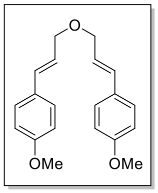

5r

4,4'-((1E,1'E)-oxybis(prop-1-ene-3,1-diyl))bis(methoxybenzene) (5r)

^{1}H NMR (500 MHz, $CDCl_3$) δ 7.45 – 7.26 (m, 4H), 6.95 – 6.76 (m, 4H), 6.57 (d, *J* = 15.9 Hz, 2H), 6.19 (dt, *J* = 15.9, 6.2 Hz, 2H), 4.17 (dd, *J* = 6.2, 1.3 Hz, 4H), 3.79 (s, 6H).

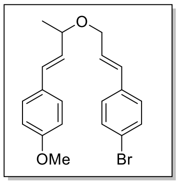

5s

1-bromo-4-((E)-3-(((E)-4-(4-methoxyphenyl)but-3-en-2-yl)oxy)prop-1-en-1-yl)benzene (5s)

^{1}H NMR (500 MHz, $CDCl_3$) δ 7.47 – 7.39 (m, 2H), 7.36 – 7.30 (m, 2H), 7.27 – 7.22 (m, 2H), 6.90 – 6.82 (m, 2H), 6.58 – 6.45 (m, 2H), 6.35 – 6.24 (m, 1H), 5.99 (dd, *J* = 15.9, 7.9 Hz, 1H), 4.20 – 4.16 (m, 1H), 4.09 – 4.02 (m, 2H), 3.79 (d, *J* = 7.9 Hz, 3H), 1.37 (d, *J* = 6.3 Hz, 3H).

^{13}C NMR (126 MHz, $CDCl_3$) δ 159.37, 135.87, 131.72, 131.69, 131.64, 131.29, 131.07, 130.70, 129.34, 129.30, 128.04, 128.01, 127.70, 127.51, 126.78, 121.31, 114.05, 76.45, 70.73, 68.50, 55.34, 21.86.

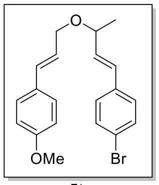

5t

1-bromo-4-((E)-3-(((E)-3-(4-methoxyphenyl)allyl)oxy)but-1-en-1-yl)benzene (5t)

^{1}H NMR (500 MHz, $CDCl_3$) δ 7.43 (d, *J* = 8.4 Hz, 2H), 7.28 (dd, *J* = 31.8, 8.6 Hz, 4H), 6.84 (d, *J* = 8.7 Hz, 2H), 6.50 (dd, *J* = 29.9, 15.9 Hz, 2H), 6.22 – 6.08 (m, 2H), 4.24 – 4.16 (m, 1H), 4.13 – 4.00 (m, 2H), 3.79 (d, *J* = 3.1 Hz, 3H), 1.36 (d, *J* = 6.4 Hz, 3H).

^{13}C NMR (126 MHz, $CDCl_3$) δ 159.27, 135.64, 132.64, 132.01, 131.70, 129.97, 129.56, 128.27, 128.02, 127.69, 124.04, 121.41, 113.96, 113.90, 75.65, 69.12, 55.29, 21.60.

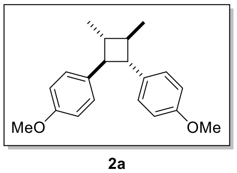

2a

(±)-4,4'-((*1S,2S,3R,4R*)-3,4-dimethylcyclobutane-1,2-diyl)bis(methoxybenzene) (2a)

Following the general procedure A; 82% yield; dr:>19:1; White Oil; R_f = 0.4 (*n*-hexane : EA = 20:1);

^{1}H NMR (500 MHz, $CDCl_3$) 7.12 (d, *J* = 8.7 Hz, 4H), 6.82 (d, *J* = 8.7 Hz, 4H), 3.76 (s, 6H), 2.84 – 2.76 (m, 2H), 1.82 (m, 2H), 1.18 (d, *J* = 6.1 Hz, 6H).^{13}C NMR (126 MHz, $CDCl_3$) δ 157.93, 135.90, 127.70, 113.68, 55.20, 52.47, 43.19, 18.85.

HRMS (ESI) m/z calcd for $[C_{20}H_{25}O_2]^+$ $[M+H]^+$: 297.1849, found 297.1852.

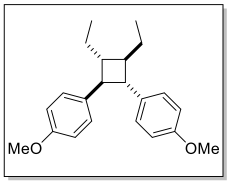

2b

(±)-4,4'-((*1S,2S,3R,4R*)-3,4-diethylcyclobutane-1,2-diyl)bis(methoxybenzene) (2b)

Following the general procedure A; 83% yield; dr:>19:1; White Oil; R_f = 0.3 (*n*-hexane : EA = 20:1);

^{1}H NMR (500 MHz, $CDCl_3$) δ 7.14 (d, *J* = 8.6 Hz, 4H), 6.82 (d, *J* = 8.6 Hz, 4H), 3.77 (s, 6H), 2.80 (dd, *J* = 5.7, 3.2 Hz, 2H), 1.98 – 1.87 (m, 2H), 1.72 – 1.53 (m, 4H), 0.87 (t, *J* = 7.5 Hz, 6H).

^{13}C NMR (126 MHz, $CDCl_3$) δ 157.75, 136.47, 127.87, 113.52, 55.13, 51.24, 47.26, 28.53, 11.87.

HRMS (ESI) m/z calcd for $[C_{22}H_{29}O_2]^+$ $[M+H]^+$: 325.2162, found 325.2163.

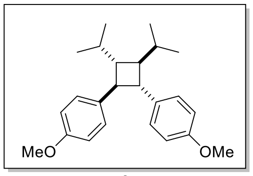

2c

(±)-4,4'-((*1S,2S,3R,4R*)-3,4-diisopropylcyclobutane-1,2-diyl)bis(methoxybenzene) (2c)

Following the general procedure A; 42% yield; dr:>19:1; White Oil; R_f = 0.2 (*n*-hexane : EA = 10:1);

^{1}H NMR (500 MHz, $CDCl_3$) δ 7.10 (d, *J* = 8.7 Hz, 4H), 6.80 (d, *J* = 8.7 Hz, 4H), 3.77 (s, 6H), 2.78 (d, *J* = 9.0 Hz, 2H), 2.05 – 1.98 (m, 2H), 1.82 (dd, *J* = 13.3, 6.7 Hz, 2H), 0.87 (dd, *J* = 8.7, 6.8 Hz, 12H).

^{13}C NMR (126 MHz, $CDCl_3$) δ 157.71, 137.12, 128.13, 113.52, 55.16, 49.35, 49.18, 32.98, 21.19, 19.70.

HRMS (ESI) m/z calcd for $[C_{24}H_{33}O_2]^+$ $[M+H]^+$: 353.2475, found 353.2481.

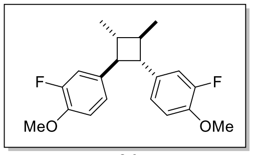

2d

(±)-4,4'-((*1S,2S,3R,4R*)-3,4-dimethylcyclobutane-1,2-diyl)bis(2-fluoro-1-methoxybenzene) (2d)

Following the general procedure A; 54% yield; dr:>19:1; White Oil; R_f = 0.4 (*n*-hexane : EA = 20:1);

^{1}H NMR (500 MHz, $CDCl_3$) δ 6.93 (d, *J* = 12.8 Hz, 2H), 6.87 (d, *J* = 4.5 Hz, 4H), 3.85 (s, 6H), 2.81 – 2.67 (m, 2H), 1.81 (d, *J* = 4.9 Hz, 2H), 1.17 (d, *J* = 5.5 Hz, 6H).

^{13}C NMR (126 MHz, $CDCl_3$) δ 153.35, 151.39, 145.90 (d, $J_{C\text{-}F}$ = 11.3 Hz), 136.55 (d, $J_{C\text{-}F}$ = 6.3 Hz), 122.25 (d, $J_{C\text{-}F}$ = 3.8 Hz), 114.34 (d, $J_{C\text{-}F}$ = 17.6 Hz), 113.37, 56.33, 52.33, 43.10, 18.69.

^{19}F NMR (471 MHz, $CDCl_3$): δ -135.31.

HRMS (ESI) m/z calcd for $[C_{20}H_{23}F_2O_2]^+$ $[M+H]^+$: 333.1661, found 333.1661.

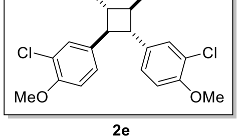

2e

(±)-4,4'-((*1S,2S,3R,4R*)-3,4-dimethylcyclobutane-1,2-diyl)bis(2-chloro-1-methoxybenzene) (2e)

Following the general procedure A; 50% yield; dr:>19:1; White Oil; R_f = 0.3 (*n*-hexane : EA = 20:1);

^{1}H NMR (500 MHz, $CDCl_3$) δ 7.21 (d, *J* = 1.9 Hz, 2H), 7.02 (dd, *J* = 8.4,

1.9 Hz, 2H), 6.84 (d, *J* = 8.4 Hz, 2H), 3.86 (s, 6H), 2.74 (dd, *J* = 5.6, 3.3 Hz, 2H), 1.81 (dd, *J* = 9.8, 4.9 Hz, 2H), 1.17 (d, *J* = 5.7 Hz, 6H).

^{13}C NMR (126 MHz, $CDCl_3$) δ 153.37, 136.48, 128.40, 126.03, 122.24, 112.01, 56.15, 52.20, 43.16, 18.68.

HRMS (ESI) m/z calcd for $[C_{20}H_{23}Cl_2O_2]^+$ $[M+H]^+$: 365.1070, found 365.1058.

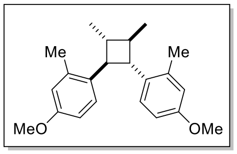

2f

(±)-4,4'-((*1S,2S,3R,4R*)-3,4-dimethylcyclobutane-1,2-diyl)bis(1-methoxy-3-methylbenzene) (2f)

Following the general procedure A; 63% yield; dr:>19:1; White Oil; R_f = 0.2 (*n*-hexane : EA = 10:1);

^{1}H NMR (500 MHz, $CDCl_3$) δ 7.32 (d, *J* = 8.5 Hz, 2H), 6.74 (dd, *J* = 8.5, 2.6 Hz, 2H), 6.60 (d, *J* = 2.6 Hz, 2H), 3.75 (s, 6H), 3.05 – 2.87 (m, 2H), 2.01 (s, 6H), 1.99 – 1.90 (m, 2H), 1.15 (d, *J* = 5.8 Hz, 6H).

^{13}C NMR (126 MHz, $CDCl_3$) δ 157.46, 137.61, 133.97, 127.06, 115.51, 111.18, 55.15, 50.42, 42.33, 20.40, 18.95.

HRMS (ESI) m/z calcd for $[C_{22}H_{29}O_2]^+$ $[M+H]^+$: 325.2162, found 325.2167.

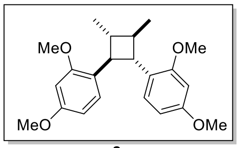

2g

(±)-4,4'-((*1S,2S,3R,4R*)-3,4-dimethylcyclobutane-1,2-diyl)bis(1,3-dimethoxybenzene) (2g)

Following the general procedure A; 51% yield; dr:>19:1; White Oil; R_f = 0.4 (*n*-hexane : EA = 10:1);

^{1}H NMR (500 MHz, Chloroform-*d*) δ 7.20 (d, *J* = 8.4 Hz, 2H), 6.43 (dd, *J* = 8.3, 2.4 Hz, 2H), 6.38 (d, *J* = 2.3 Hz, 2H), 3.76 (s, 6H), 3.68 (s, 6H), 3.30 – 3.17 (m, 2H), 1.74 (q, *J* = 5.0 Hz, 2H), 1.16 (d, *J* = 5.9 Hz, 6H).

^{13}C NMR (126 MHz, $CDCl_3$) δ 158.73, 158.43, 127.71, 124.96, 103.69, 98.16, 55.25, 55.06, 44.84, 43.41, 19.19.

HRMS (ESI) m/z calcd for $[C_{22}H_{29}O_4]^+$ $[M+H]^+$: 357.2060, found 357.2060.

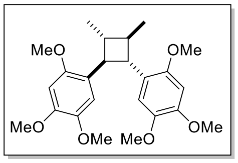

2h

(±)-5,5'-((*1S,2S,3R,4R*)-3,4-dimethylcyclobutane-1,2-diyl)bis(1,2,4-trimethoxybenzene) (2h)

Following the general procedure A; 70% yield; dr:>19:1; White Oil; R_f = 0.3 (*n*-hexane : EA = 10:1);

^{1}H NMR (500 MHz, Chloroform-*d*) δ 6.95 (s, 2H), 6.47 (s, 2H), 3.86 (d, *J* = 12.0 Hz, 12H), 3.69 (s, 6H), 3.27 (d, *J* = 9.0 Hz, 2H), 1.77 (q, *J* = 5.1 Hz, 2H), 1.19 (d, *J* = 5.9 Hz, 6H).

^{13}C NMR (126 MHz, $CDCl_3$) δ 151.57, 147.44, 143.01, 123.79, 112.17, 97.77, 56.63, 56.44, 56.08, 45.26, 43.38, 18.98.

HRMS (ESI) m/z calcd for $[C_{24}H_{33}O_6]^+$ $[M+H]^+$: 417.2272, found 417.2275.

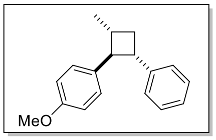

4a

(±)-1-methoxy-4-((*1S,2R,4S*)-2-methyl-4-phenylcyclobutyl)benzene (4a)

Following the general procedure B; 78% yield; dr:>10:1; White Oil; R_f = 0.3 (*n*-hexane : EA = 10:1);

^{1}H NMR (500 MHz, Chloroform-*d*) δ 7.37 (t, *J* = 7.5 Hz, 2H), 7.27 (q, *J* = 7.9, 6.8 Hz, 5H), 6.95 (d, *J* = 8.6 Hz, 2H), 3.88 (s, 3H), 3.49 (q, *J* = 9.9 Hz, 1H), 3.05 (t, *J* = 9.5 Hz, 1H), 2.68 – 2.56 (m, 1H), 2.43 (m, 1H), 1.80 (q, *J* = 10.1 Hz, 1H), 1.29 (d, *J* = 6.5 Hz, 3H).

HRMS (ESI) m/z calcd for $[C_{18}H_{21}O]^+$ $[M+H]^+$: 253.1587, found 253.1590.

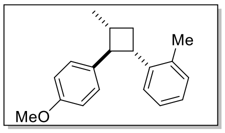

4b

(±)-1-((*1S,2S,3R*)-2-(4-methoxyphenyl)-3-methylcyclobutyl)-2-methylbenzene (4b)

Following the general procedure B; 76% yield; dr:>10:1; White Oil; R_f = 0.3 (*n*-hexane : EA = 10:1);

^{1}H NMR (500 MHz, Chloroform-*d*) δ 7.29 (d, *J* = 7.7 Hz, 1H), 7.20 – 7.11 (m, 3H), 7.09 – 7.04 (m, 2H), 6.81 (d, *J* = 8.6 Hz, 2H), 3.73 (s, 3H), 3.51 (q, *J* = 9.9 Hz, 1H), 3.11 (t, *J* = 9.5 Hz, 1H), 2.64 – 2.51 (m, 1H), 2.38 – 2.26 (m, 1H), 2.17 (s, 3H), 1.53 (q, *J* = 10.1 Hz, 1H), 1.18 (d, *J* = 6.5 Hz, 3H).

^{13}C NMR (126 MHz, $CDCl_3$) δ 158.01, 142.24, 135.97, 135.86, 129.92, 127.70, 125.84, 125.77, 125.67, 113.68, 55.13, 53.31, 41.67, 35.61, 34.64, 20.56, 19.77.

HRMS (ESI) m/z calcd for $[C_{19}H_{23}O]^+$ $[M+H]^+$: 267.1743, found 267.1748.

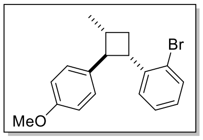

4c

(±)-1-bromo-2-((*1S,2S,3R*)-2-(4-methoxyphenyl)-3-methylcyclobutyl)benzene (4c)

Following the general procedure B; 64% yield; dr:>10:1; White Oil; R_f = 0.3 (*n*-hexane : EA = 15:1);

^{1}H NMR (500 MHz, Chloroform-*d*) δ 7.55 – 7.43 (m, 1H), 7.35 (d, *J* = 7.1 Hz, 1H), 7.21 (dd, *J* = 31.1, 8.1 Hz, 3H), 7.11 – 6.90 (m, 1H), 6.83 (d, *J* = 8.6 Hz, 2H), 3.75 (s, 3H), 3.70 (q, *J* = 10.0 Hz, 1H), 3.13 (t, *J* = 9.6 Hz, 1H), 2.84 – 2.68 (m, 1H), 2.42 – 2.23 (m, 1H), 1.45 (q, *J* = 10.1 Hz, 1H), 1.20 (d, *J* = 6.5 Hz, 3H).

^{13}C NMR (126 MHz, $CDCl_3$) δ 158.11, 143.10, 135.20, 132.64, 127.74, 127.72, 127.43, 127.34, 124.12, 113.73, 55.19, 52.72, 43.47, 35.72, 35.32, 20.56.

HRMS (ESI) m/z calcd for $[C_{18}H_{20}BrO]^+$ $[M+H]^+$: 331.0692, found 331.0529.

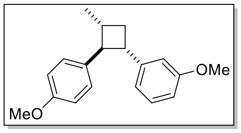

4d

(±)-1-methoxy-3-((*1S,2S,3R*)-2-(4-methoxyphenyl)-3-methylcyclobutyl)benzene (4d)

Following the general procedure B; 67% yield; dr:>10:1; White Oil; R_f = 0.3 (*n*-hexane : EA = 15:1);

^{1}H NMR (500 MHz, Chloroform-*d*) δ 7.19 – 7.13 (m, 3H), 6.83 (d, *J* = 8.7 Hz, 2H), 6.78 (d, *J* = 7.6 Hz, 1H), 6.75 – 6.67 (m, 2H), 3.76 (s, 3H), 3.75 (s, 3H), 3.35 (q, *J* = 9.8 Hz, 1H), 2.93 (t, *J* = 9.5 Hz, 1H), 2.54 – 2.42 (m, 1H), 2.31 (ddd, *J* = 14.1, 9.5, 7.4 Hz, 1H), 1.68 (q, *J* = 10.1 Hz, 1H), 1.17 (d, *J* = 6.5 Hz, 4H).

^{13}C NMR (126 MHz, $CDCl_3$) δ 159.54, 158.04, 146.36, 135.73, 129.17, 127.73, 127.68, 119.03, 113.72, 113.67, 112.55, 110.94, 55.40, 55.18, 55.05, 44.13, 35.32, 33.88, 20.42.

HRMS (ESI) m/z calcd for $[C_{19}H_{23}O_2]^+$ $[M+H]^+$: 283.1693, found 283.1698.

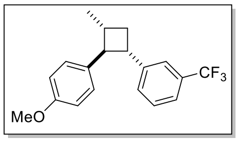

4e

(±)-1-((*1S,2S,3R*)-2-(4-methoxyphenyl)-3-methylcyclobutyl)-3-(trifluoromethyl)benzene (4e)

Following the general procedure B; 60% yield; dr:>10:1; White Oil; R_f = 0.3 (*n*-hexane : EA = 10:1);

^{1}H NMR (500 MHz, Chloroform-*d*) δ 7.38 – 7.29 (m, 2H), 7.28 – 7.20 (m, 2H), 7.07 (d, *J* = 8.6 Hz, 2H), 6.80 – 6.74 (m, 2H), 3.68 (s, 3H), 3.40 – 3.26 (m, 1H), 2.84 (t, *J* = 9.6 Hz, 1H), 2.44 (dt, *J* = 10.1, 7.8 Hz, 1H), 2.27 (ddd, *J* = 9.2, 6.7, 2.2 Hz, 1H), 1.60 (q, *J* = 10.1 Hz, 1H), 1.09 (d, *J* = 6.5 Hz, 3H).

^{13}C NMR (126 MHz, $CDCl_3$) δ 158.24, 145.47, 135.10, 130.62, 130.37, 130.09, 128.62, 127.74, 125.37, 124.3 (q, J_{C-F} = 271.9 Hz), 123.23 (q, J_{C-F} = 3.8 Hz), 122.77 (q, J_{C-F} = 3.8 Hz), 113.85, 55.55, 55.18, 43.93, 35.54, 33.78, 20.32.

^{19}F NMR (471 MHz, $CDCl_3$): δ -62.47.

HRMS (ESI) m/z calcd for $[C_{19}H_{20}F_3O]^+$ $[M+H]^+$: 321.1461, found 321.1461.

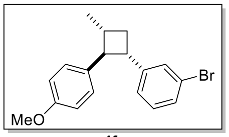

4f

(±)-1-bromo-3-((*1S,2S,3R*)-2-(4-methoxyphenyl)-3-methylcyclobutyl)benzene (4f)

Following the general procedure B; 77% yield; dr:>10:1; White Oil; R_f = 0.2 (*n*-hexane : EA = 15:1);

^{1}H NMR (500 MHz, Chloroform-*d*) δ 7.24 (s, 1H), 7.19 (dt, *J* = 7.2, 1.8 Hz, 1H), 7.05 (d, *J* = 8.6 Hz, 2H), 7.03 – 6.96 (m, 2H), 6.79 – 6.73 (m, 2H), 3.68 (s, 3H), 3.24 (q, *J* = 9.9 Hz, 1H), 2.81 (t, *J* = 9.5 Hz, 1H), 2.40 (dt, *J* = 10.1, 7.8 Hz, 1H), 2.29 – 2.17 (m, 1H), 1.56 (q, *J* = 10.1 Hz, 1H), 1.08 (d, *J* = 6.5 Hz, 3H).

^{13}C NMR (126 MHz, $CDCl_3$) δ 158.15, 146.96, 135.18, 129.76, 129.64, 128.97, 127.69, 125.33, 122.42, 113.80, 55.46, 55.18, 43.75, 35.45, 33.81, 20.35.

HRMS (ESI) m/z calcd for $[C_{18}H_{20}BrO]^+$ $[M+H]^+$: 331.0692, found 331.0679.

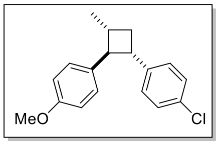

4g

(±)-1-chloro-4-((*1S,2S,3R*)-2-(4-methoxyphenyl)-3-methylcyclobutyl)benzene (4g)

Following the general procedure B; 71% yield; dr:>10:1; White Oil; R_f = 0.3 (*n*-hexane : EA = 10:1);

^{1}H NMR (500 MHz, Chloroform-*d*) δ 7.20 (d, *J* = 8.4 Hz, 2H), 7.12 (d, *J* = 8.6 Hz, 2H), 7.08 (d, *J* = 8.4 Hz, 2H), 6.83 (d, *J* = 8.7 Hz, 2H), 3.75 (s, 3H), 3.31 (td, *J* = 10.0, 7.9 Hz, 1H), 2.86 (t, *J* = 9.5 Hz, 1H), 2.47 (dt, *J* = 10.2, 7.7 Hz, 1H), 2.37 – 2.25 (m, 1H), 1.63 (q, *J* = 10.1 Hz, 1H), 1.16 (d, *J* = 6.5 Hz, 3H).

^{13}C NMR (126 MHz, $CDCl_3$) δ 158.13, 143.00, 135.28, 131.49, 128.25, 127.92, 127.68, 113.78, 55.72, 55.13, 43.61, 35.36, 33.78, 20.35.

HRMS (ESI) m/z calcd for $[C_{18}H_{20}ClO]^+$ $[M+H]^+$: 287.1197, found 287.1200.

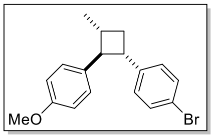

4h

(±)-1-bromo-4-((*1S,2S,3R*)-2-(4-methoxyphenyl)-3-methylcyclobutyl)benzene (4h)

Following the general procedure B; 45% yield; dr:>10:1; White Oil; R_f = 0.3 (*n*-hexane : EA = 10:1);

^{1}H NMR (500 MHz, Chloroform-*d*) δ 7.45 – 7.40 (m, 2H), 7.21 (d, *J* = 8.6 Hz, 2H), 7.13 – 7.08 (m, 2H), 6.95 – 6.89 (m, 2H), 3.84 (s, 3H), 3.43 – 3.31 (m, 1H), 2.94 (t, *J* = 9.5 Hz, 1H), 2.56 (dt, *J* = 10.0, 7.8 Hz, 1H), 2.45 – 2.32 (m, 1H), 1.71 (q, *J* = 10.1 Hz, 1H), 1.24 (d, *J* = 6.5 Hz, 3H).

HRMS (ESI) m/z calcd for $[C_{18}H_{20}BrO]^+$ $[M+H]^+$: 331.0692, found 331.0538.

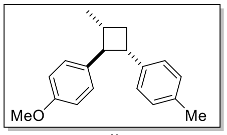

4i

(±)-1-methoxy-4-((*1S,2R,4S*)-2-methyl-4-(p-tolyl)cyclobutyl)benzene (4i)

Following the general procedure B; 82% yield; dr:>10:1; White Oil; R_f = 0.5 (*n*-hexane : EA = 10:1);

^{1}H NMR (500 MHz, Chloroform-*d*) δ 7.20 (d, *J* = 8.6 Hz, 2H), 7.16 – 7.08 (m, 4H), 6.88 (d, *J* = 8.6 Hz, 2H), 3.81 (s, 3H), 3.38 (q, *J* = 9.8 Hz, 1H), 2.96 (t, *J* = 9.5 Hz, 1H), 2.58 – 2.47 (m, 1H), 2.34 (s, 4H), 1.71 (q, *J* = 10.1 Hz, 1H), 1.22 (d, *J* = 6.5 Hz, 3H).

^{13}C NMR (126 MHz, $CDCl_3$) δ 157.98, 141.61, 135.90, 135.34, 128.88, 127.72, 126.50, 113.68, 55.56, 55.18, 43.81, 35.35, 34.06, 20.98, 20.47.

HRMS (ESI) m/z calcd for $[C_{19}H_{23}O]^+$ $[M+H]^+$: 267.1743, found 267.1747.

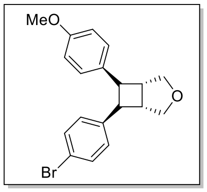

6a

(*1S,5R,6R,7S*)-6-(4-bromophenyl)-7-(4-methoxyphenyl)-3-oxabicyclo[3.2.0]heptane (6a)

Following the general procedure C; 79% yield; dr:>10:1; White Oil; R_f = 0.2 (*n*-hexane : EA = 10:1);

^{1}H NMR (500 MHz, Chloroform-*d*) δ 7.23 – 7.16 (m, 2H), 6.93 – 6.77 (m, 4H), 6.68 – 6.64 (m, 2H), 4.08 (dd, *J* = 9.4, 4.0 Hz, 2H), 3.76 – 3.63 (m, 7H), 3.22 (dq, *J* = 4.1, 1.9 Hz, 2H).

HRMS (ESI) m/z calcd for $[C_{19}H_{20}BrO_2]^+$ $[M+H]^+$: 359.0641, found 359.0641.

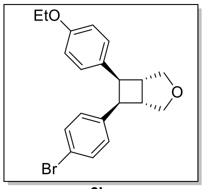

6b

(*1S,5R,6S,7R*)-6-(4-bromophenyl)-7-(4-ethoxyphenyl)-3-oxabicyclo[3.2.0]heptane (6b)

Following the general procedure C; 71% yield; dr:>10:1; White Oil; R_f = 0.2 (*n*-hexane : EA = 10:1);

^{1}H NMR (500 MHz, Chloroform-*d*) δ 7.20 (d, *J* = 8.3 Hz, 2H), 6.81 (dd, *J* = 14.2, 8.5 Hz, 4H), 6.64 (d, *J* = 8.5 Hz, 2H), 4.08 (dd, *J* = 9.4, 4.4 Hz, 2H), 3.92 (q, *J* = 7.0 Hz, 2H), 3.67 (dq, *J* = 11.4, 4.1 Hz, 4H), 3.22 (s, 2H), 1.35 (t, *J* = 7.0 Hz, 3H).

^{13}C NMR (126 MHz, $CDCl_3$) δ 156.98, 140.02, 132.40, 130.73, 129.76, 128.97, 119.36, 113.96, 73.92, 73.83, 63.28, 46.61, 46.38, 42.46, 42.14, 14.76.

HRMS (ESI) m/z calcd for $[C_{20}H_{22}BrO_2]^+$ $[M+H]^+$: 373.0798, found 373.0793.

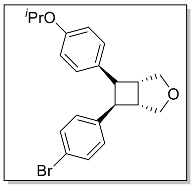

6c

(*1S,5R,6S,7S*)-6-(4-bromophenyl)-7-(4-isopropoxyphenyl)-3-oxabicyclo[3.2.0]heptane (6c)

Following the general procedure C; 77% yield; dr:>10:1; White Oil; R_f = 0.2 (*n*-hexane : EA = 10:1);

^{1}H NMR (500 MHz, Chloroform-*d*) δ 7.19 (d, *J* = 8.4 Hz, 2H), 6.80 (dd, *J* = 11.3, 8.5 Hz, 4H), 6.63 (d, *J* = 8.6 Hz, 2H), 4.41 (p, *J* = 6.1 Hz, 1H), 4.08 (dd, *J* = 9.4, 4.2 Hz, 2H), 3.67 (dq, *J* = 13.4, 4.4 Hz, 4H), 3.22 (t, *J* = 4.2 Hz, 2H), 1.25 (d, *J* = 6.1 Hz, 7H).

^{13}C NMR (126 MHz, $CDCl_3$) δ 155.87, 139.99, 132.46, 130.68, 129.78, 128.98, 119.33, 115.69, 73.93, 73.83, 69.96, 46.64, 46.42, 42.33, 42.04, 29.67, 21.92.

HRMS (ESI) m/z calcd for $[C_{21}H_{24}BrO_2]^+$ $[M+H]^+$: 387.0954, found 387.0947.

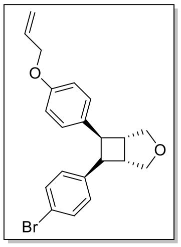

6d

(*1R,5S,6R,7R*)-6-(4-(allyloxy)phenyl)-7-(4-bromophenyl)-3-oxabicyclo[3.2.0]heptane (6d)

Following the general procedure C; 73% yield; dr:>10:1; White Oil; R_f = 0.3 (*n*-hexane : EA = 10:1);

^{1}H NMR (500 MHz, Chloroform-*d*) δ 7.23 – 7.16 (m, 2H), 6.84 – 6.76 (m, 4H), 6.69 – 6.63 (m, 2H), 6.00 (ddt, *J* = 17.2, 10.5, 5.3 Hz, 1H), 5.35 (dq, *J* = 17.3, 1.6 Hz, 1H), 5.25 (dq, *J* = 10.5, 1.4 Hz, 1H), 4.43 (dt, *J* = 5.3, 1.5 Hz, 2H), 3.72 – 3.60 (m, 4H), 3.22 (td, *J* = 4.0, 1.3 Hz, 2H).

^{13}C NMR (126 MHz, $CDCl_3$) δ 156.61, 139.97, 133.27, 132.72, 130.73, 129.76, 128.96, 119.37, 117.54, 114.22, 73.92, 73.83, 68.69, 46.61, 46.36, 42.38, 42.09.

HRMS (ESI) m/z calcd for $[C_{21}H_{22}BrO_2]^+$ $[M+H]^+$: 385.0798, found 385.0789.

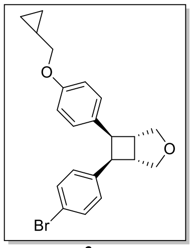

6e

(*1S,5R,6S,7R*)-6-(4-bromophenyl)-7-(4-(cyclopropylmethoxy)phenyl)-3-oxabicyclo[3.2.0]heptane (6e)

Following the general procedure C; 81% yield; dr:>10:1; White Oil; R_f = 0.2 (*n*-hexane : EA = 15:1);

^{1}H NMR (500 MHz, Chloroform-*d*) δ 7.20 (d, *J* = 8.4 Hz, 2H), 6.81 (dd, *J* = 11.8, 8.6 Hz, 4H), 6.64 (d, *J* = 8.7 Hz, 2H), 4.07 (dd, *J* = 9.5, 3.2 Hz, 2H), 3.72 – 3.59 (m, 6H), 3.22 (dq, *J* = 3.9, 1.8 Hz, 2H), 1.25 – 1.16 (m, 1H), 0.65 – 0.55 (m, 2H), 0.30 (dt, *J* = 6.0, 4.6 Hz, 2H).

^{13}C NMR (126 MHz, $CDCl_3$) δ 157.06, 140.02, 132.51, 130.74, 129.77, 128.96, 119.36, 114.15, 73.92, 73.83, 72.70, 46.60, 46.36, 42.46, 42.11, 10.23, 3.08.

HRMS (ESI) m/z calcd for $[C_{22}H_{24}BrO_2]^+$ $[M+H]^+$: 399.0954, found 399.0946.

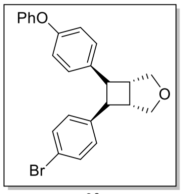

6f

(*1S,5R,6R,7S*)-6-(4-bromophenyl)-7-(4-phenoxyphenyl)-3-oxabicyclo[3.2.0]heptane (6f)

Following the general procedure C; 80% yield; dr:>10:1; White Oil; R_f = 0.2 (*n*-hexane : EA = 10:1);

^{1}H NMR (500 MHz, Chloroform-*d*) δ 7.34 – 7.27 (m, 2H), 7.24 – 7.18 (m, 2H), 7.07 – 7.00 (m, 1H), 6.92 – 6.86 (m, 2H), 6.83 – 6.75 (m, 5H), 4.10 (dd, *J* = 10.8, 9.4 Hz, 2H), 3.76 – 3.64 (m, 4H), 3.31 – 3.21 (m, 2H).

^{13}C NMR (126 MHz, $CDCl_3$) δ 157.89, 154.68, 139.76, 135.69, 130.72, 129.84, 129.64, 129.29, 122.65, 119.47, 119.15, 117.91, 73.92, 73.84, 46.77, 46.63, 41.86, 41.80.

HRMS (ESI) m/z calcd for $[C_{24}H_{22}BrO_2]^+$ $[M+H]^+$: 421.0798, found 421.0795.

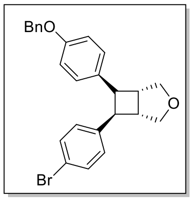

6g

(*1R,5S,6S,7S*)-6-(4-(benzyloxy)phenyl)-7-(4-bromophenyl)-3-oxabicyclo[3.2.0]heptane (6g)

Following the general procedure C; 67% yield; dr:>10:1; White Oil; R_f = 0.2 (*n*-hexane : EA = 15:1);

^1H NMR (500 MHz, Chloroform-*d*) δ 7.39 – 7.33 (m, 4H), 7.33 – 7.27 (m, 1H), 7.19 (d, *J* = 8.4 Hz, 2H), 6.83 (d, *J* = 8.6 Hz, 2H), 6.79 (d, *J* = 8.4 Hz, 2H), 6.71 (d, *J* = 8.7 Hz, 2H), 4.96 (s, 2H), 4.07 (dd, *J* = 9.4, 3.4 Hz, 2H), 3.67 (td, *J* = 9.1, 4.5 Hz, 4H), 3.21 (s, 2H).

^{13}C NMR (126 MHz, $CDCl_3$) δ 156.82, 139.98, 137.04, 132.85, 130.74, 129.77, 129.00, 128.48, 127.83, 127.41, 119.37, 114.41, 73.91, 73.82, 69.91, 46.61, 46.38, 42.41, 42.13.

HRMS (ESI) m/z calcd for $[C_{25}H_{24}BrO_2]^+$ $[M+H]^+$: 435.0954, found 435.0950.

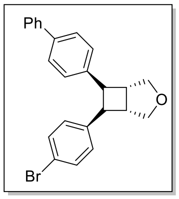

6h

(*1R,5S,6S,7R*)-6-([1,1'-biphenyl]-4-yl)-7-(4-bromophenyl)-3-oxabicyclo[3.2.0]heptane (6h)

Following the general procedure C; 74% yield; dr:>10:1; White Oil; R_f = 0.2 (*n*-hexane : EA = 10:1);

^1H NMR (500 MHz, Chloroform-*d*) δ 7.53 – 7.47 (m, 2H), 7.42 – 7.32 (m, 4H), 7.31 – 7.26 (m, 1H), 7.23 – 7.16 (m, 2H), 6.99 (d, *J* = 8.2 Hz, 2H), 6.83 (d, *J* = 8.4 Hz, 2H), 4.10 (dd, *J* = 9.3, 7.2 Hz, 2H), 3.77 (dd, *J* = 10.1, 5.0 Hz, 1H), 3.70 (dt, *J* = 9.3, 4.6 Hz, 3H), 3.37 – 3.20 (m, 2H).

^{13}C NMR (126 MHz, $CDCl_3$) δ 140.68, 139.86, 139.56, 138.57, 130.81, 129.75, 128.64, 128.43, 127.04, 126.84, 126.51, 119.49, 73.89, 73.82, 46.72, 46.61, 42.35, 42.27.

HRMS (ESI) m/z calcd for $[C_{24}H_{22}BrO]^+$ $[M+H]^+$: 405.0849, found 405.0842.

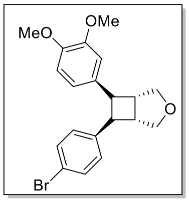

6i

(*1S,5R,6R,7S*)-6-(4-bromophenyl)-7-(3,4-dimethoxyphenyl)-3-oxabicyclo[3.2.0]heptane (6i)

Following the general procedure C; 67% yield; dr:>10:1; White Oil; R_f = 0.3 (*n*-hexane : EA = 10:1);

^1H NMR (500 MHz, Chloroform-*d*) δ 7.21 (d, *J* = 8.4 Hz, 2H), 6.82 (d, *J* = 8.4 Hz, 2H), 6.65 (d, *J* = 8.2 Hz, 1H), 6.56 (dd, *J* = 8.2, 1.9 Hz, 1H), 6.27 (d, *J* = 1.9 Hz, 1H), 4.08 (d, *J* = 10.0 Hz, 2H), 3.79 (s, 3H), 3.68 (ddd, *J* = 12.2, 6.6, 2.7 Hz, 4H), 3.62 (s, 3H), 3.22 (q, *J* = 4.3 Hz, 2H).

^{13}C NMR (126 MHz, $CDCl_3$) δ 148.39, 147.21, 140.14, 133.08, 131.71, 130.83, 129.79, 127.98, 119.94, 119.53, 111.99, 110.61, 73.90, 73.86, 55.78, 55.73, 46.72, 46.62, 42.61, 42.12.

HRMS (ESI) m/z calcd for $[C_{20}H_{22}BrO_3]^+$ $[M+H]^+$: 389.0747, found 389.0745.

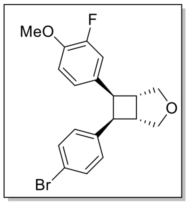

6j

(*1S,5R,6S,7R*)-6-(4-bromophenyl)-7-(3-fluoro-4-methoxyphenyl)-3-oxabicyclo[3.2.0]heptane (6j)

Following the general procedure C; 90% yield; dr:>10:1; White Oil; R_f = 0.3 (*n*-hexane : EA = 10:1);

^{1}H NMR (500 MHz, Chloroform-*d*) δ 7.22 (d, *J* = 8.4 Hz, 2H), 6.80 (d, *J* = 8.4 Hz, 2H), 6.74 – 6.64 (m, 2H), 6.61 (d, *J* = 8.5 Hz, 1H), 4.07 (dd, *J* = 9.4, 3.2 Hz, 2H), 3.78 (s, 3H), 3.72 – 3.56 (m, 4H), 3.28 – 3.12 (m, 2H).

^{13}C NMR (126 MHz, $CDCl_3$) δ 152.92, 150.97, 145.56 (d, J_{C-F} = 11.3 Hz), 139.63, 133.70 (d, J_{C-F} = 5.0 Hz), 130.92, 129.70, 123.65 (d, J_{C-F} = 3.8 Hz), 119.63, 115.66 (d, J_{C-F} = 18.9 Hz), 112.90, 73.80, 73.79, 56.20, 46.60, 46.21, 42.30, 42.07, 29.70.

^{19}F NMR (471 MHz, $CDCl_3$): δ -135.69.

HRMS (ESI) m/z calcd for $[C_{19}H_{19}BrFO_2]^+$ $[M+H]^+$: 377.0547, found 377.0546.

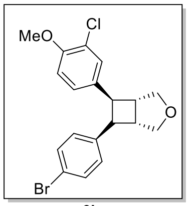

6k

(*1S,5R,6R,7R*)-6-(4-bromophenyl)-7-(3-chloro-4-methoxyphenyl)-3-oxabicyclo[3.2.0]heptane (6k)

Following the general procedure C; 85% yield; dr:>10:1; White Oil; R_f = 0.4 (*n*-hexane : EA = 10:1);

^{1}H NMR (500 MHz, Chloroform-*d*) δ 7.26 – 7.19 (m, 2H), 6.97 (d, *J* = 2.2 Hz, 1H), 6.84 – 6.77 (m, 2H), 6.71 (dd, *J* = 8.5, 2.2 Hz, 1H), 6.64 (d, *J* = 8.5 Hz, 1H), 4.07 (d, *J* = 9.4 Hz, 2H), 3.79 (s, 3H), 3.72 – 3.57 (m, 4H), 3.21 (q, *J* = 3.7 Hz, 2H).

^{13}C NMR (126 MHz, $CDCl_3$) δ 152.97, 139.54, 133.69, 130.88, 129.70, 129.60, 127.28, 121.74, 119.61, 111.55, 73.75, 56.00, 46.56, 45.97, 42.31, 42.01.

HRMS (ESI) m/z calcd for $[C_{19}H_{19}BrClO_2]^+$ $[M+H]^+$: 393.0251, found 393.0247.

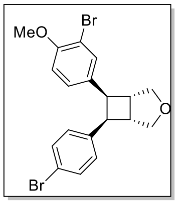

6l

(*1R,5S,6R,7S*)-6-(3-bromo-4-methoxyphenyl)-7-(4-bromophenyl)-3-oxabicyclo[3.2.0]heptane (6l)

Following the general procedure C; 68% yield; dr:>10:1; White Oil; R_f = 0.4 (*n*-hexane : EA = 10:1);

^{1}H NMR (500 MHz, Chloroform-*d*) δ 7.23 (d, *J* = 8.3 Hz, 2H), 7.14 (d, *J* = 2.0 Hz, 1H), 6.81 (d, *J* = 8.3 Hz, 2H), 6.74 (dd, *J* = 8.4, 2.0 Hz, 1H), 6.61 (d, *J* = 8.5 Hz, 1H), 4.07 (d, *J* = 9.5 Hz, 2H), 3.78 (s, 3H), 3.71 – 3.60 (m, 4H), 3.21 (q, *J* = 4.0 Hz, 2H).

^{13}C NMR (126 MHz, $CDCl_3$) δ 153.87, 139.53, 134.17, 132.66, 130.89, 129.72, 128.06, 119.63, 111.39, 111.03, 73.76, 56.11, 46.61, 45.91, 42.38, 42.00.

HRMS (ESI) m/z calcd for $[C_{19}H_{19}Br_2O_2]^+$ $[M+H]^+$: 436.9746, found 436.9738.

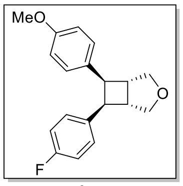

6m

(*1S,5R,6S,7R*)-6-(4-fluorophenyl)-7-(4-methoxyphenyl)-3-oxabicyclo[3.2.0]heptane (6m)

Following the general procedure C; 92% yield; dr:>10:1; White Oil; R_f = 0.3 (*n*-hexane : EA = 10:1);

^{1}H NMR (500 MHz, Chloroform-*d*) δ 6.90 – 6.85 (m, 2H), 6.85 – 6.80 (m, 2H), 6.80 – 6.72 (m, 2H), 6.67 – 6.60 (m, 2H), 4.08 (d, *J* = 9.5 Hz, 2H),

3.68 (d, J = 10.1 Hz, 7H), 3.28 – 3.17 (m, 2H).

^{13}C NMR (126 MHz, $CDCl_3$) δ 161.84, 159.90, 157.51, 136.56 (d, $J_{C\text{-}F}$ = 3.8 Hz), 132.63, 129.35 (d, $J_{C\text{-}F}$ = 7.6 Hz), 128.98, 128.76, 114.44 (d, $J_{C\text{-}F}$ = 20.2 Hz), 113.17, 73.90, 73.84, 55.05, 46.42, 42.28, 42.22.

^{19}F NMR (471 MHz, $CDCl_3$): δ -117.65.

HRMS (ESI) m/z calcd for $[C_{19}H_{20}FO_2]^+$ $[M+H]^+$: 299.1442, found 299.1439.

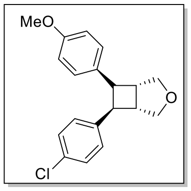

6n

(*1S,5R,6S,7S*)-6-(4-chlorophenyl)-7-(4-methoxyphenyl)-3-oxabicyclo[3.2.0]heptane (6n)

Following the general procedure C; 84% yield; dr:>10:1; White Oil; R_f = 0.3 (*n*-hexane : EA = 10:1);

^{1}H NMR (500 MHz, Chloroform-*d*) δ 7.08 – 7.02 (m, 2H), 6.90 – 6.79 (m, 4H), 6.68 – 6.61 (m, 2H), 4.08 (dd, J = 9.5, 3.1 Hz, 2H), 3.69 (d, J = 16.5 Hz, 7H), 3.23 (dd, J = 2.5, 1.4 Hz, 2H).

^{13}C NMR (126 MHz, $CDCl_3$) δ 157.62, 139.47, 132.56, 131.25, 129.36, 128.99, 127.82, 113.31, 73.93, 73.84, 55.11, 46.56, 46.42, 42.46, 42.17.

HRMS (ESI) m/z calcd for $[C_{19}H_{20}ClO_2]^+$ $[M+H]^+$: 315.1146, found 315.1146.

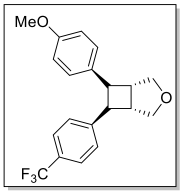

6o

(*1R,5S,6S,7R*)-6-(4-methoxyphenyl)-7-(4-(trifluoromethyl)phenyl)-3-oxabicyclo[3.2.0]heptane (6o)

Following the general procedure C; 77% yield; dr:>10:1; White Oil; R_f = 0.4 (*n*-hexane : EA = 15:1);

^{1}H NMR (500 MHz, Chloroform-*d*) δ 7.33 (d, J = 8.2 Hz, 2H), 7.03 (d, J = 8.1 Hz, 2H), 6.83 (d, J = 8.7 Hz, 2H), 6.63 (d, J = 8.7 Hz, 2H), 4.09 (dd, J = 9.4, 4.7 Hz, 2H), 3.79 – 3.65 (m, 7H), 3.27 (dtd, J = 18.2, 8.4, 5.0 Hz, 2H).

^{13}C NMR (126 MHz, $CDCl_3$) δ 157.68, 145.13, 132.31, 128.93, 128.26, 124.57 (q, $J_{C\text{-}F}$ = 11.3 Hz), 113.33, 73.88, 73.76, 55.08, 46.89, 46.45, 42.55, 41.96.

^{19}F NMR (471 MHz, $CDCl_3$): δ -62.31.

HRMS (ESI) m/z calcd for $[C_{20}H_{20}F_3O_2]^+$ $[M+H]^+$: 349.1410, found 349.1406.

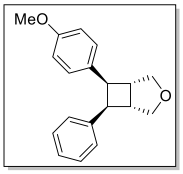

6p

(*1R,5S,6R,7S*)-6-(4-methoxyphenyl)-7-phenyl-3-oxabicyclo[3.2.0]heptane (6p)

Following the general procedure C; 61% yield; dr:>10:1; White Oil; R_f = 0.4 (*n*-hexane : EA = 10:1);

^{1}H NMR (500 MHz, Chloroform-*d*) δ 7.09 (t, J = 7.5 Hz, 2H), 7.01 (d, J = 7.3 Hz, 1H), 6.94 (d, J = 7.2 Hz, 2H), 6.88 – 6.81 (m, 2H), 6.68 – 6.58 (m, 2H), 4.09 (d, J = 9.4 Hz, 2H), 3.69 (d, J = 7.7 Hz, 7H), 3.30 (dt, J = 9.7, 4.9 Hz, 1H), 3.24 (dt, J = 8.3, 4.8 Hz, 1H).

^{13}C NMR (126 MHz, $CDCl_3$) δ 157.45, 140.86, 132.97, 129.02, 128.08, 127.70, 125.54, 113.10, 73.97, 55.08, 47.15, 46.48, 42.60, 41.97.

HRMS (ESI) m/z calcd for $[C_{19}H_{21}O_2]^+$ $[M+H]^+$: 281.1536, found 281.1535.

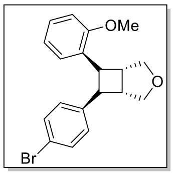

6q

(*1S,5R,6R,7S*)-6-(4-bromophenyl)-7-(2-methoxyphenyl)-3-oxabicyclo[3.2.0]heptane (6q)

Following the general procedure C; 83% yield; dr:>10:1; White Oil; R_f = 0.3 (*n*-hexane : EA = 10:1);

^{1}H NMR (500 MHz, Chloroform-*d*) δ 7.22 – 7.08 (m, 3H), 7.07 – 6.98 (m, 1H), 6.80 (dd, *J* = 7.2, 5.1 Hz, 3H), 6.49 (d, *J* = 8.1 Hz, 1H), 4.10 (d, *J* = 9.4 Hz, 1H), 4.03 (d, *J* = 9.2 Hz, 1H), 3.93 (dd, *J* = 10.0, 6.1 Hz, 1H), 3.68 (dd, *J* = 9.3, 5.5 Hz, 2H), 3.62 (dd, *J* = 10.1, 4.8 Hz, 1H), 3.50 (s, 4H), 3.03 (dt, *J* = 9.3, 5.4 Hz, 1H).

^{13}C NMR (126 MHz, $CDCl_3$) δ 156.52, 141.02, 130.06, 129.65, 128.17, 127.22, 127.01, 119.67, 119.08, 109.55, 73.97, 73.94, 54.54, 47.43, 42.94, 41.97, 39.64.

HRMS (ESI) m/z calcd for $[C_{19}H_{20}BrO_2]^+$ $[M+H]^+$: 359.0641, found 359.0642.

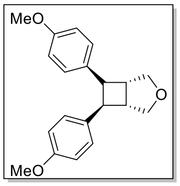

6r

(*1R,5S,6S,7S*)-6,7-bis(4-methoxyphenyl)-3-oxabicyclo[3.2.0]heptane (6r)

Following the general procedure C; 46% yield; dr:>10:1; White Oil; R_f = 0.3 (*n*-hexane : EA = 20:1);

^{1}H NMR (500 MHz, Chloroform-*d*) δ 6.85 (d, *J* = 8.7 Hz, 4H), 6.64 (d, *J* = 8.7 Hz, 4H), 4.08 (d, *J* = 9.4 Hz, 2H), 3.70 (s, 7H), 3.68 (dd, *J* = 3.4, 1.7 Hz, 1H), 3.65 (d, *J* = 4.1 Hz, 2H), 3.25 – 3.19 (m, 2H).

HRMS (ESI) m/z calcd for $[C_{20}H_{23}O_3]^+$ $[M+H]^+$: 311.1642 found 311.1640.

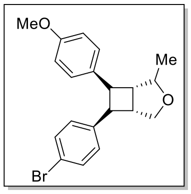

6s

(*1S,2R,5R,6R,7S*)-6-(4-bromophenyl)-7-(4-methoxyphenyl)-2-methyl-3-oxabicyclo[3.2.0]heptane (6s)

Following the general procedure C; 56% yield; dr:>10:1; White Oil; R_f = 0.3 (*n*-hexane : EA = 10:1);

^{1}H NMR (500 MHz, Chloroform-*d*) δ 7.20 (d, *J* = 8.4 Hz, 2H), 6.81 (dd, *J* = 13.4, 8.5 Hz, 4H), 6.65 (d, *J* = 8.7 Hz, 2H), 4.35 (q, *J* = 6.5 Hz, 1H), 4.01 (qd, *J* = 9.6, 3.7 Hz, 2H), 3.71 (s, 4H), 3.66 (dd, *J* = 9.9, 5.1 Hz, 1H), 3.30 – 3.20 (m, 1H), 2.92 (dd, *J* = 8.0, 5.5 Hz, 1H), 1.16 (d, *J* = 6.5 Hz, 3H).

^{13}C NMR (126 MHz, $CDCl_3$) δ 157.62, 140.06, 132.55, 130.76, 129.74, 128.95, 119.37, 113.31, 81.01, 71.54, 55.11, 48.75, 46.67, 46.10, 42.06, 19.36.

HRMS (ESI) m/z calcd for $[C_{20}H_{22}BrO_2]^+$ $[M+H]^+$: 373.0798, found 373.0796.

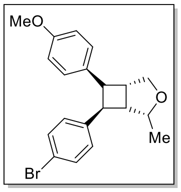

6t

(*1S,2R,5S,6S,7S*)-7-(4-bromophenyl)-6-(4-methoxyphenyl)-2-methyl-3-oxabicyclo[3.2.0]heptane (6t)

Following the general procedure C; 67% yield; dr:>10:1; White Oil; R_f = 0.3 (*n*-hexane : EA = 10:1);

^{1}H NMR (500 MHz, Chloroform-*d*) δ 7.20 (d, *J* = 8.4 Hz, 2H), 6.84 (d, *J* = 8.6 Hz, 2H), 6.78 (d, *J* = 8.4 Hz, 2H), 6.65 (d, *J* = 8.7 Hz, 2H), 4.34 (q, *J* = 6.5 Hz, 1H), 4.07 – 3.97 (m, 2H), 3.70 (d, *J* = 7.4 Hz, 5H), 3.24 (dtd, *J* = 7.6, 4.9, 3.7, 1.8 Hz, 1H), 2.92 (dd, *J* = 8.1, 4.6 Hz, 1H), 1.17 (d, *J* = 6.5 Hz, 3H).

^{13}C NMR (126 MHz, $CDCl_3$) δ 157.60, 140.02, 132.56, 130.73, 129.70, 128.95, 119.36, 113.31, 80.87, 71.61, 55.08, 48.37, 46.41, 46.34, 42.37,

19.30.

HRMS (ESI) m/z calcd for $[C_{20}H_{22}BrO_2]^+$ $[M+H]^+$: 373.0798, found 373.0795.

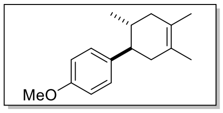

8a

(±)-(*1R,2R*)-4'-methoxy-2,4,5-trimethyl-1,2,3,6-tetrahydro-1,1'-biphenyl (8a)

Following the general procedure D; 98% yield; dr:>10:1; White Oil; R_f = 0.3 (*n*-hexane : EA = 30:1);

^{1}H NMR (500 MHz, Chloroform-*d*) δ 7.09 (d, *J* = 8.6 Hz, 2H), 6.85 (d, *J* = 8.7 Hz, 2H), 3.79 (s, 3H), 2.35 (td, *J* = 10.6, 5.7 Hz, 1H), 2.24 – 2.02 (m, 3H), 1.94 – 1.78 (m, 2H), 1.64 (d, *J* = 13.8 Hz, 6H), 0.71 (d, *J* = 6.2 Hz, 3H).

^{13}C NMR (126 MHz, $CDCl_3$) δ 157.70, 138.11, 128.39, 125.40, 125.24, 113.63, 55.11, 47.78, 41.80, 41.60, 34.21, 19.97, 18.69, 18.61.

HRMS (ESI) m/z calcd for $[C_{16}H_{23}O]^+$ $[M+H]^+$: 231.1743, found 231.1747.

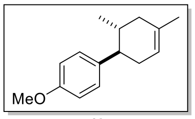

8b

(±)-(*1R,2R*)-4'-methoxy-2,4-dimethyl-1,2,3,6-tetrahydro-1,1'-biphenyl (8b)

Following the general procedure D; 96% yield; dr:>10:1; White Oil; R_f = 0.4 (*n*-hexane : EA = 20:1);

^{1}H NMR (500 MHz, Chloroform-*d*) δ 7.13 (d, *J* = 8.5 Hz, 2H), 6.89 (d, *J* = 8.5 Hz, 2H), 5.50 (s, 1H), 3.82 (s, 3H), 2.35 (td, *J* = 10.7, 5.3 Hz, 1H), 2.30 – 2.09 (m, 3H), 2.00 – 1.91 (m, 1H), 1.89 – 1.81 (m, 1H), 1.74 (s, 3H), 0.76 (d, *J* = 6.4 Hz, 3H).

^{13}C NMR (126 MHz, $CDCl_3$) δ 157.82, 138.19, 133.81, 128.54, 121.01, 113.74, 55.19, 47.02, 39.93, 35.37, 34.02, 23.49, 20.33.

HRMS (ESI) m/z calcd for $[C_{15}H_{21}O]^+$ $[M+H]^+$: 217.1587, found 217.1591.

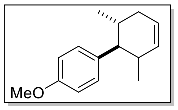

8c

(±)-(*1S,2R*)-4'-methoxy-2,6-dimethyl-1,2,3,6-tetrahydro-1,1'-biphenyl (8c)

Following the general procedure D; 97% yield; dr:>10:1; White Oil; R_f = 0.3 (*n*-hexane : EA = 20:1);

^{1}H NMR (500 MHz, Chloroform-*d*) δ 7.06 (d, *J* = 8.0 Hz, 2H), 6.83 (d, *J* = 8.0 Hz, 2H), 5.68 (d, *J* = 39.8 Hz, 2H), 3.78 (s, 3H), 2.66 (dd, *J* = 10.4, 5.0 Hz, 1H), 2.36 – 2.21 (m, 2H), 2.13 (s, 1H), 1.80 (dd, *J* = 16.7, 9.3 Hz, 1H), 0.79 (dd, *J* = 45.9, 6.6 Hz, 6H).

^{13}C NMR (126 MHz, $CDCl_3$) δ 157.55, 135.32, 133.41, 130.04, 125.05, 113.15, 55.06, 50.42, 35.50, 34.73, 26.40, 20.51, 16.75.

HRMS (ESI) m/z calcd for $[C_{15}H_{21}O]^+$ $[M+H]^+$: 217.1587, found 217.1591.

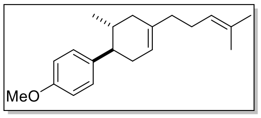

8d

(±)-(*1R,2R*)-4'-methoxy-2-methyl-4-(4-methylpent-3-en-1-yl)-1,2,3,6-tetrahydro-1,1'-biphenyl (8d)

Following the general procedure D; 93% yield; dr:>10:1; White Oil; R_f = 0.3 (*n*-hexane : EA = 30:1);

^{1}H NMR (500 MHz, Chloroform-*d*) δ 7.08 (d, *J* = 8.6 Hz, 2H), 6.83 (d, *J* = 8.6 Hz, 2H), 5.45 (s, 1H), 5.14 (t, *J* = 6.9 Hz, 1H), 3.77 (s, 3H), 2.41 – 2.06 (m, 6H), 1.99 (t, *J* = 7.7 Hz, 2H), 1.93 – 1.76 (m, 2H), 1.70 (s, 3H), 1.61 (d, *J* = 14.4 Hz, 3H), 0.71 (d, *J* = 6.3 Hz, 3H).

^{13}C NMR (126 MHz, $CDCl_3$) δ 157.69, 138.14, 137.37, 131.30, 128.42, 124.35, 120.40, 113.61, 55.08, 47.02, 38.07, 37.51, 35.14, 33.92, 26.43,

25.70, 20.25, 17.68.

HRMS (ESI) m/z calcd for $[C_{20}H_{29}O]^+$ $[M+H]^+$: 285.2213, found 285.2219.

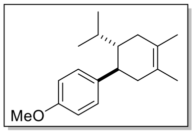

8e

(±)-(*1R,2S*)-2-isopropyl-4'-methoxy-4,5-dimethyl-1,2,3,6-tetrahydro-1,1'-biphenyl (8e)

Following the general procedure D; 95% yield; dr:>10:1; White Oil; R_f = 0.4 (*n*-hexane : EA = 20:1);

^{1}H NMR (500 MHz, Chloroform-*d*) δ 7.00 (d, *J* = 8.6 Hz, 2H), 6.75 (d, *J* = 8.6 Hz, 2H), 3.68 (s, 3H), 2.49 (td, *J* = 10.7, 6.0 Hz, 1H), 2.08 – 1.94 (m, 2H), 1.83 (d, *J* = 10.2 Hz, 2H), 1.73 (ddt, *J* = 10.5, 8.4, 4.0 Hz, 1H), 1.58 (s, 4H), 1.52 (s, 3H), 1.46 (td, *J* = 6.9, 3.4 Hz, 1H), 0.75 (d, *J* = 7.0 Hz, 3H), 0.57 (d, *J* = 6.8 Hz, 3H).

^{13}C NMR (126 MHz, $CDCl_3$) δ 157.62, 137.81, 128.56, 125.25, 125.05, 113.65, 55.07, 43.91, 43.78, 42.74, 30.70, 27.03, 21.30, 18.99, 18.57, 14.84.

HRMS (ESI) m/z calcd for $[C_{18}H_{27}O]^+$ $[M+H]^+$: 259.2056, found 259.2054.

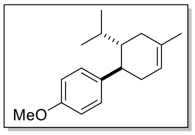

8f

(±)-(*1R,2S*)-2-isopropyl-4'-methoxy-4-methyl-1,2,3,6-tetrahydro-1,1'-biphenyl (8f)

Following the general procedure D; 92% yield; dr:>10:1; White Oil; R_f = 0.3 (*n*-hexane : EA = 30:1);

^{1}H NMR (500 MHz, Chloroform-*d*) δ 7.09 (d, *J* = 8.6 Hz, 2H), 6.83 (d, *J* = 8.6 Hz, 2H), 3.77 (s, 3H), 2.53 (td, *J* = 10.7, 5.3 Hz, 1H), 2.20 (d, *J* = 17.3 Hz, 1H), 2.16 – 2.06 (m, 1H), 1.95 – 1.78 (m, 3H), 1.71 (s, 3H), 1.55 (td, *J* = 6.9, 3.2 Hz, 1H), 0.84 (d, *J* = 7.0 Hz, 3H), 0.66 (d, *J* = 6.8 Hz, 3H).

^{13}C NMR (126 MHz, $CDCl_3$) δ 157.62, 137.83, 133.84, 128.59, 120.51, 113.63, 55.08, 43.46, 43.12, 36.21, 29.09, 27.07, 23.65, 21.23, 14.80.

HRMS (ESI) m/z calcd for $[C_{17}H_{25}O]^+$ $[M+H]^+$: 245.1900, found 245.1903.

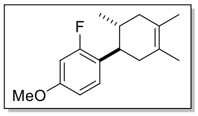

8g

(±)-(*1R,2R*)-2'-fluoro-4'-methoxy-2,4,5-trimethyl-1,2,3,6-tetrahydro-1,1'-biphenyl (8g)

Following the general procedure D; 96% yield; dr:>10:1; White Oil; R_f = 0.3 (*n*-hexane : EA = 20:1);

^{1}H NMR (500 MHz, Chloroform-*d*) δ 6.97 (t, *J* = 8.5 Hz, 1H), 6.64 – 6.35 (m, 2H), 3.67 (s, 3H), 2.63 (td, *J* = 10.9, 5.3 Hz, 1H), 2.10 (d, *J* = 12.0 Hz, 1H), 1.97 (d, *J* = 17.3 Hz, 2H), 1.89 – 1.70 (m, 2H), 1.54 (d, *J* = 11.6 Hz, 6H), 0.64 (d, *J* = 6.3 Hz, 3H).

^{13}C NMR (126 MHz, $CDCl_3$) δ 162.42, 160.48, 158.64 (d, J_{C-F} = 10.1 Hz), 128.96 (d, J_{C-F} = 7.6 Hz), 125.24 (d, J_{C-F} = 12.6 Hz), 124.16 (d, J_{C-F} = 16.4 Hz), 109.90 (d, J_{C-F} = 2.5 Hz), 101.27 (d, J_{C-F} = 26.5 Hz), 55.33, 41.46, 40.48, 39.91, 33.32, 19.64, 18.70, 18.60.

^{19}F NMR (471 MHz, $CDCl_3$): δ -116.94.

HRMS (ESI) m/z calcd for $[C_{16}H_{22}FO]^+$ $[M+H]^+$: 249.1649, found 249.1644.

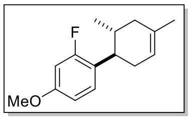

8h

(±)-(*1R,2R*)-2'-fluoro-4'-methoxy-2,4-dimethyl-1,2,3,6-tetrahydro-1,1'-biphenyl (8h)

Following the general procedure D; 94% yield; dr:>10:1; White Oil; R_f = 0.5 (*n*-hexane : EA = 10:1);

^{1}H NMR (500 MHz, Chloroform-*d*) δ 6.98 (t, *J* = 8.5 Hz, 1H), 6.63 – 6.43

(m, 2H), 5.34 (s, 1H), 3.67 (s, 3H), 2.59 (q, J = 9.9 Hz, 1H), 2.09 (s, 2H), 2.04 – 1.93 (m, 1H), 1.87 (dd, J = 10.5, 5.3 Hz, 1H), 1.81 – 1.66 (m, 1H), 1.60 (s, 3H), 0.66 (d, J = 6.4 Hz, 3H).

^{13}C NMR (126 MHz, $CDCl_3$) δ 162.49, 160.55, 158.73 (d, $J_{C\text{-}F}$ = 11.3 Hz), 133.82, 129.05 (d, $J_{C\text{-}F}$ = 7.6 Hz), 124.22 (d, $J_{C\text{-}F}$ = 15.1 Hz), 120.72, 109.96 (d, $J_{C\text{-}F}$ = 2.5 Hz), 101.33 (d, $J_{C\text{-}F}$ = 27.7 Hz), 55.41, 39.74, 33.50, 33.06, 23.43, 19.90.

^{19}F NMR (471 MHz, $CDCl_3$): δ -116.90.

HRMS (ESI) m/z calcd for $[C_{15}H_{20}FO]^+$ $[M+H]^+$: 235.1493, found 235.1495.

4. The spectra of the compounds

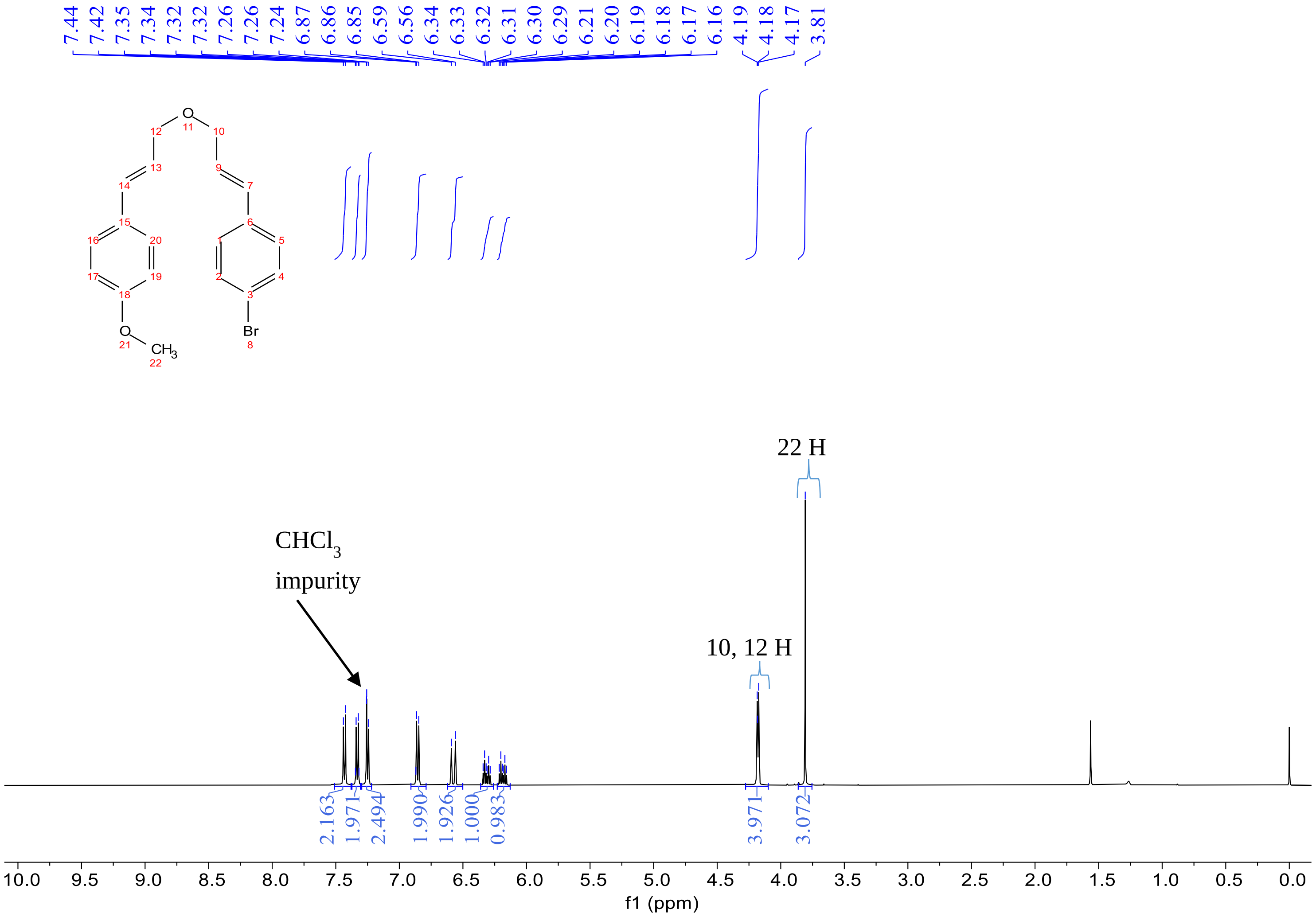

Supplementary Figure 20. ^{1}H NMR of **5a** (500 MHz, Chloroform-*d*)

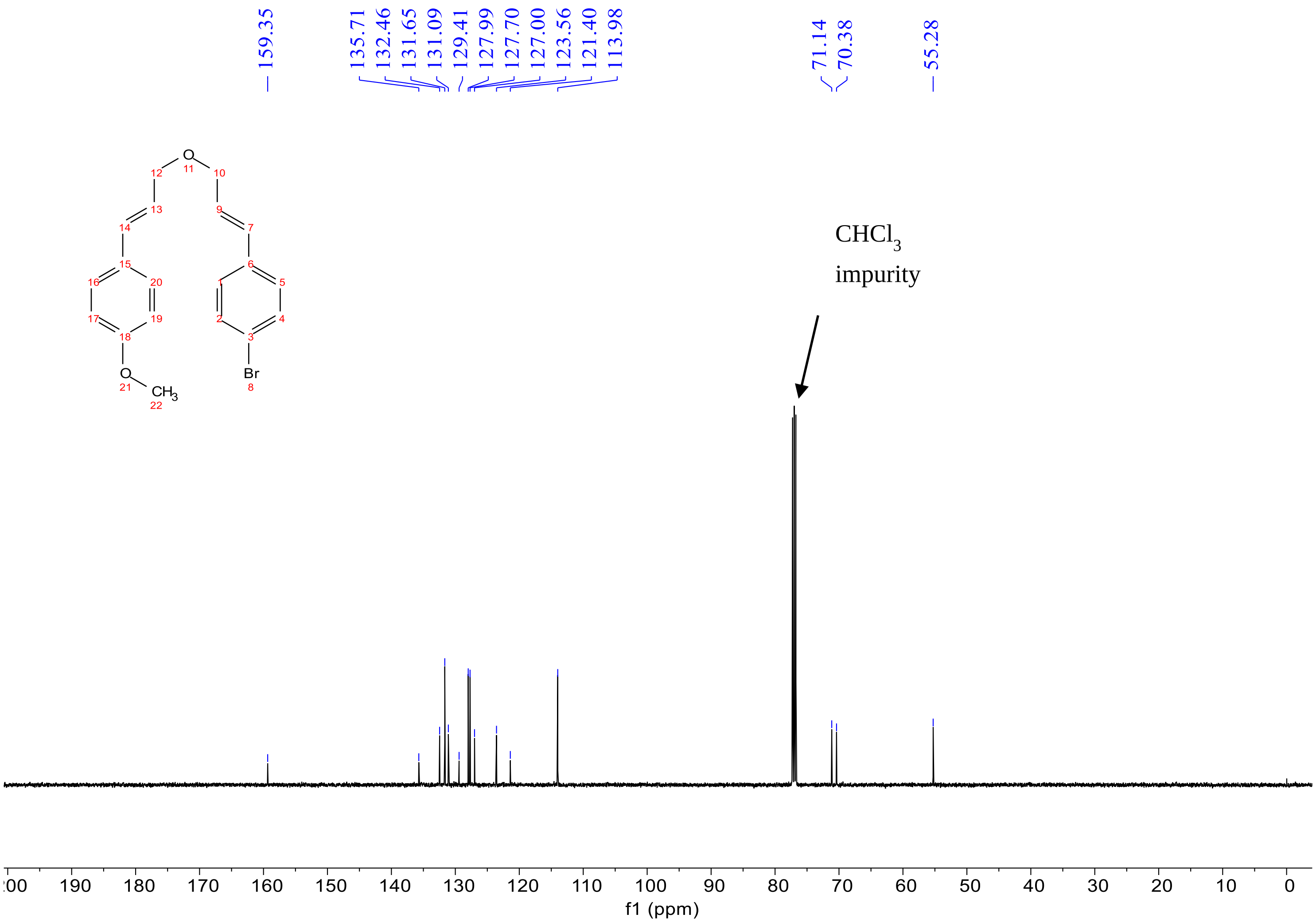

Supplementary Figure 21. ^{13}C NMR of **5a** (126 MHz, Chloroform-*d*)

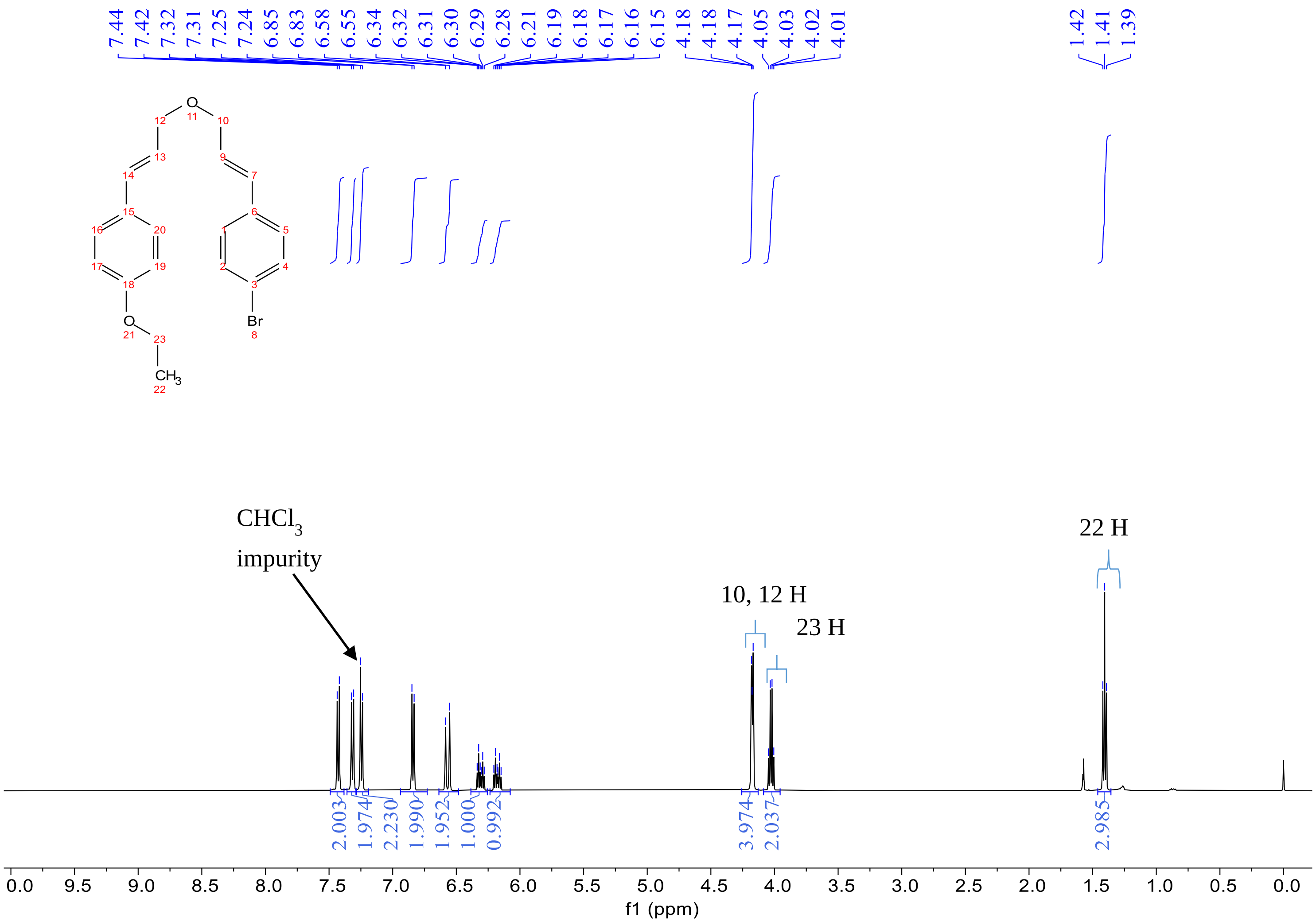

Supplementary Figure 22. ^{1}H NMR of **5b** (500 MHz, Chloroform-*d*)

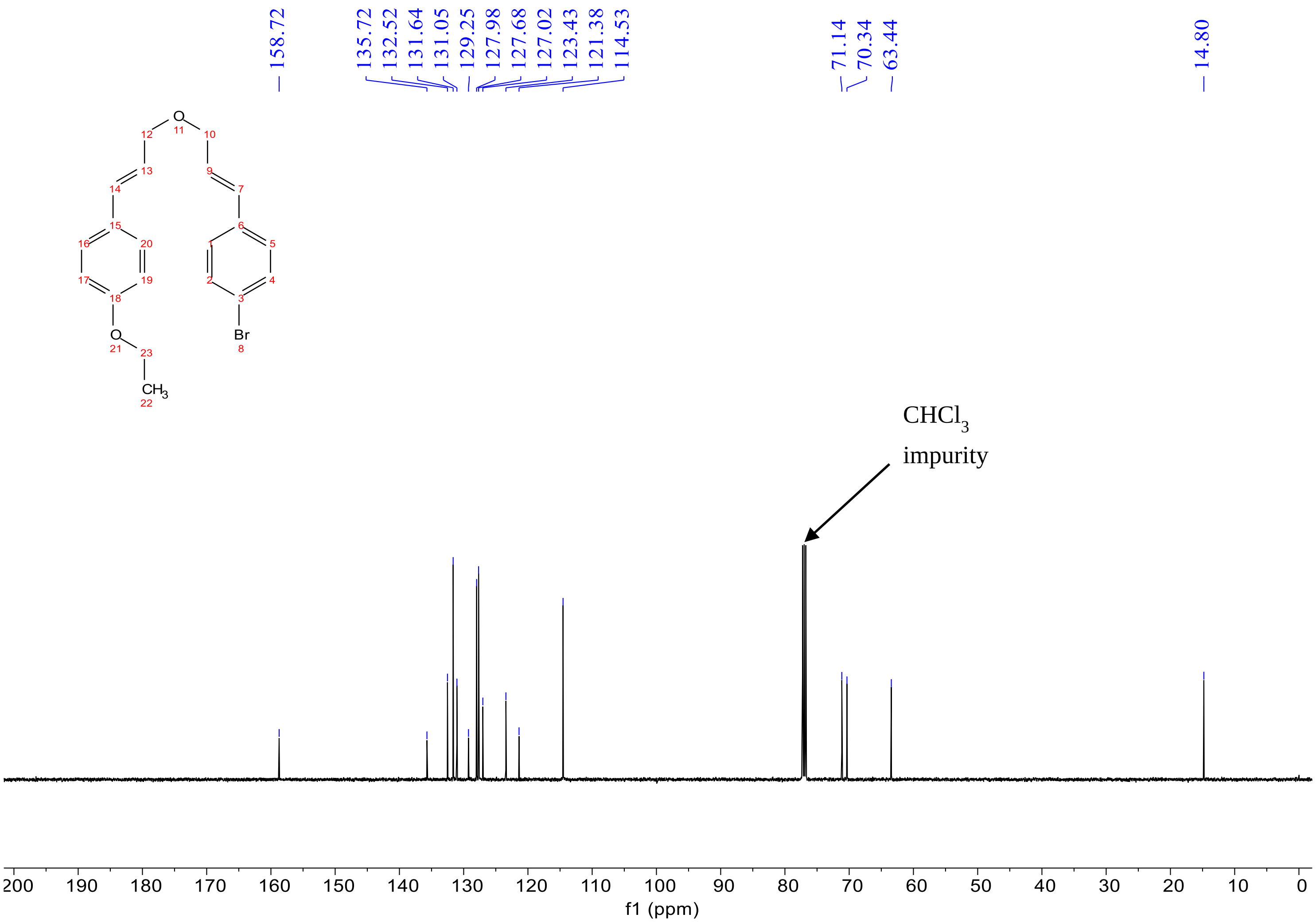

Supplementary Figure 23. ^{13}C NMR of **5b** (126 MHz, Chloroform-*d*)

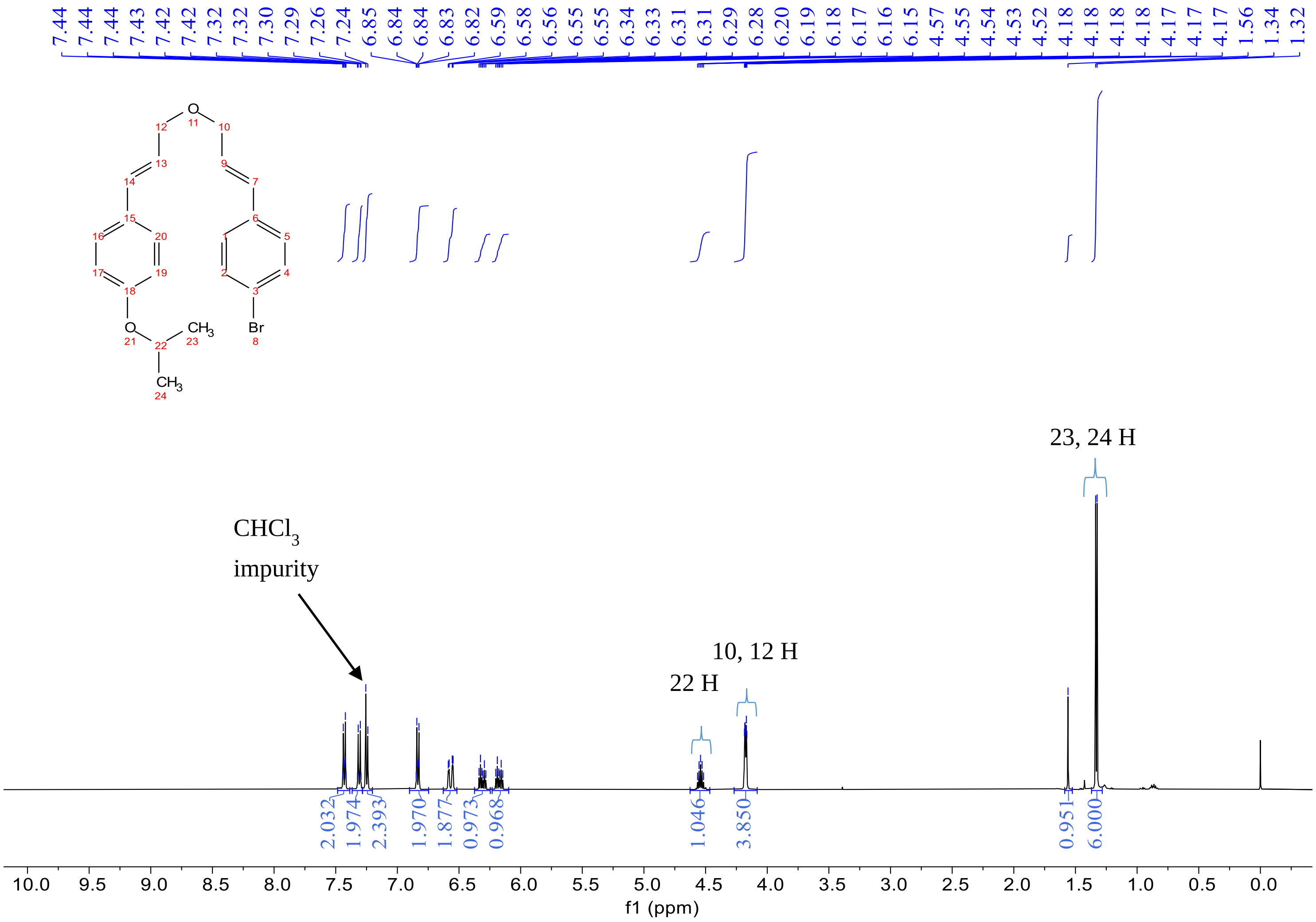

Supplementary Figure 24. ^{1}H NMR of **5c** (500 MHz, Chloroform-*d*)

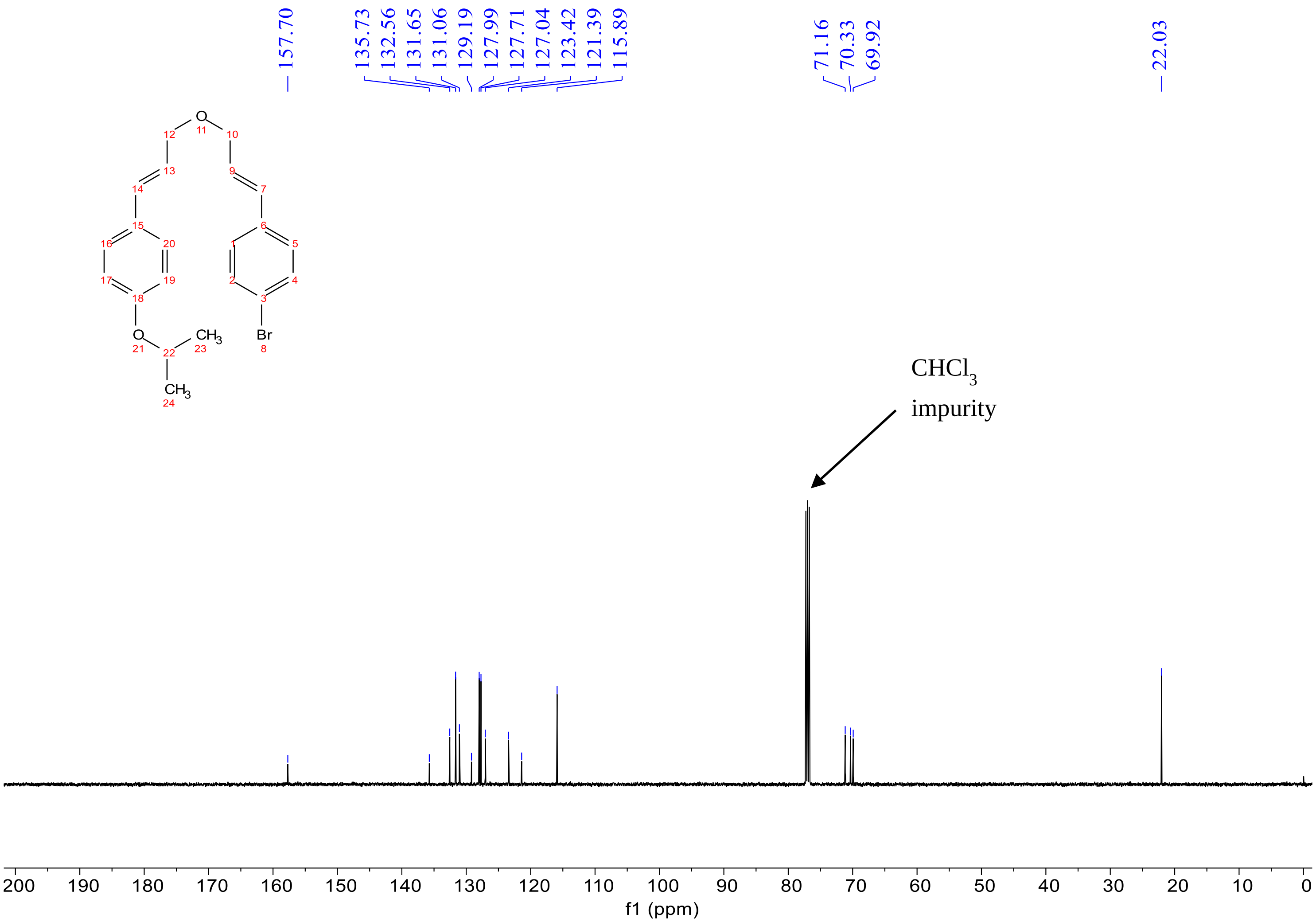

Supplementary Figure 25. ^{13}C NMR of **5c** (126 MHz, Chloroform-*d*)

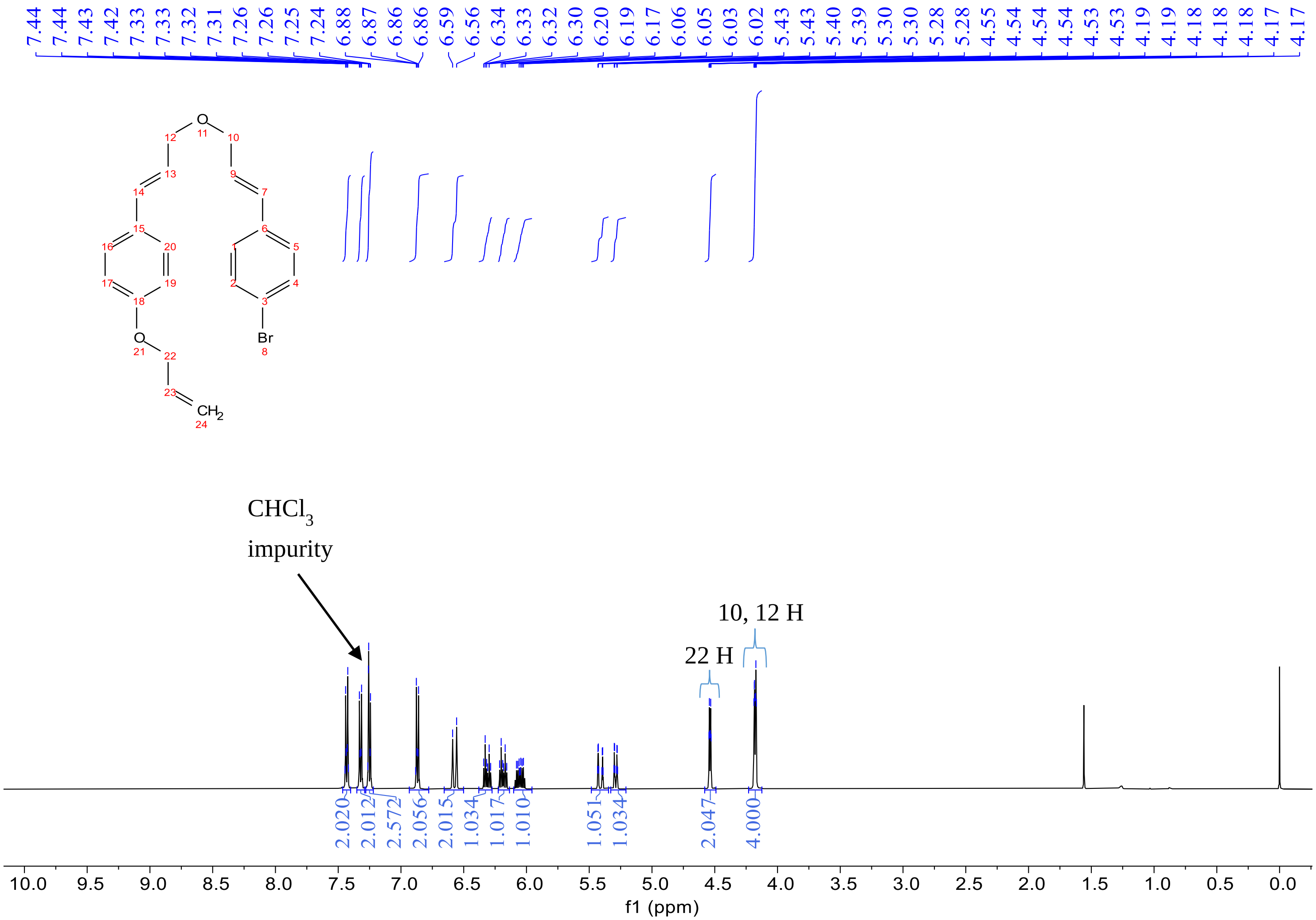

Supplementary Figure 26. ^{1}H NMR of **5d** (500 MHz, Chloroform-*d*)

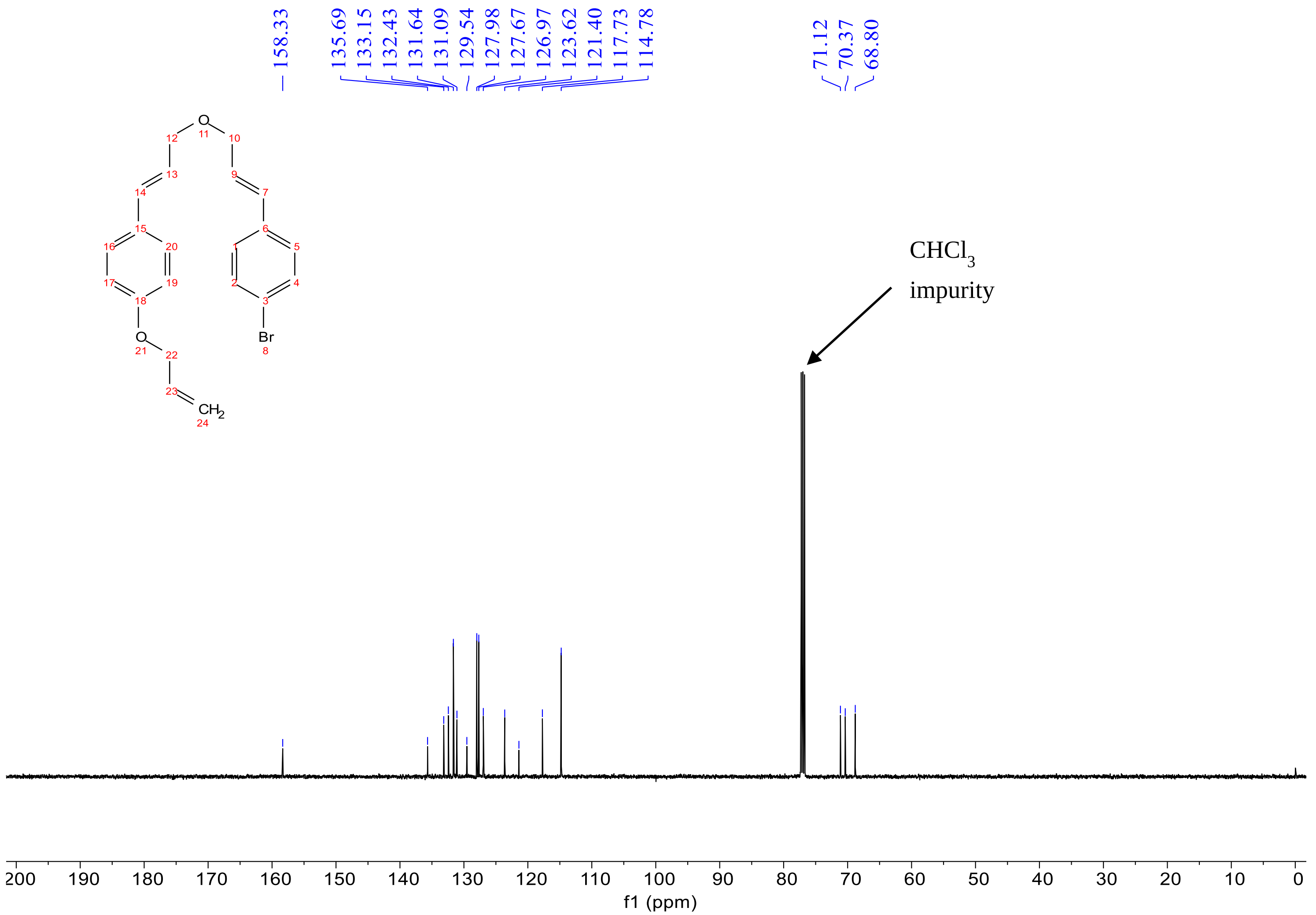

Supplementary Figure 27. ^{13}C NMR of **5d** (126 MHz, Chloroform-*d*)

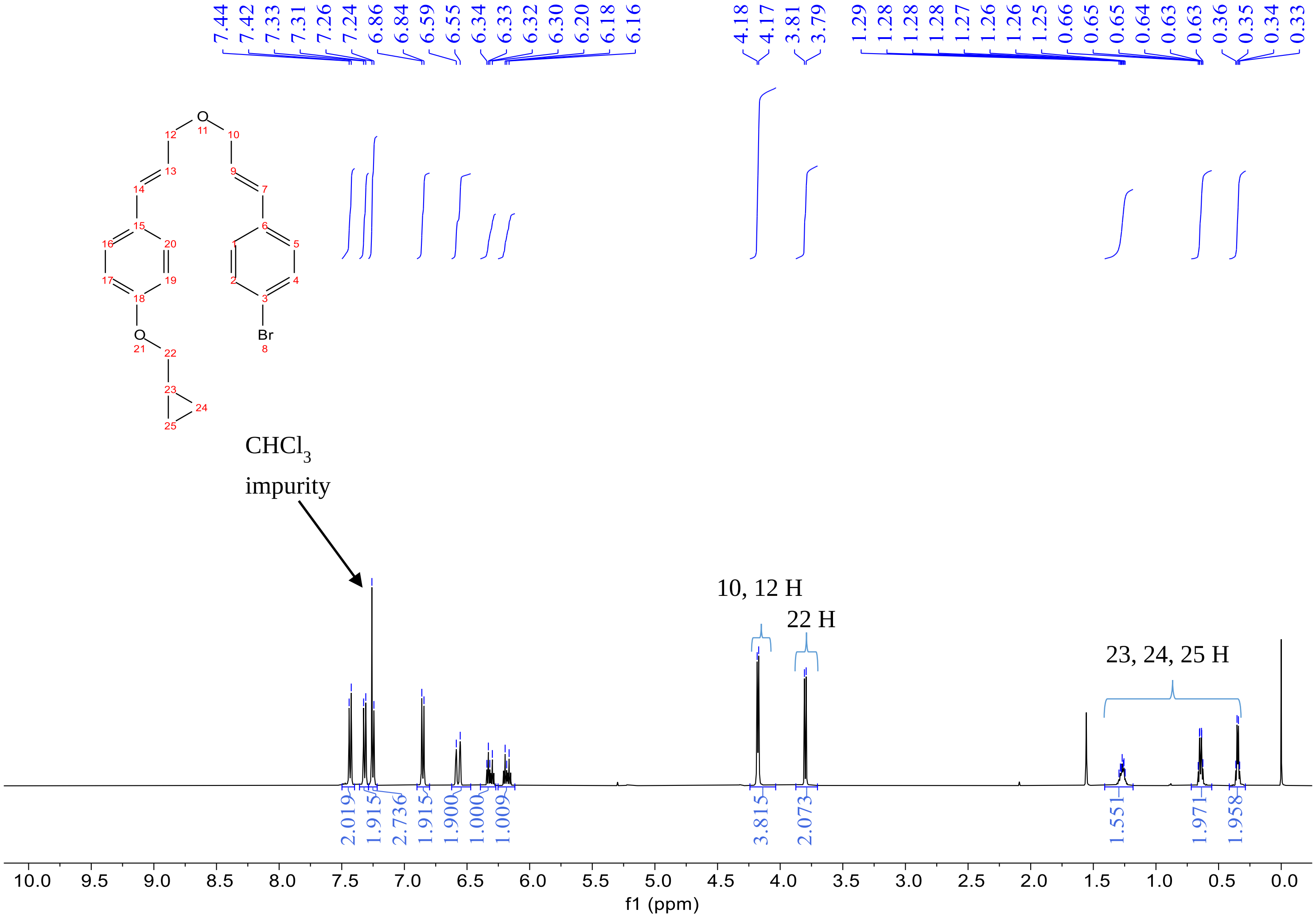

Supplementary Figure 28. ^{1}H NMR of **5e** (500 MHz, Chloroform-*d*)

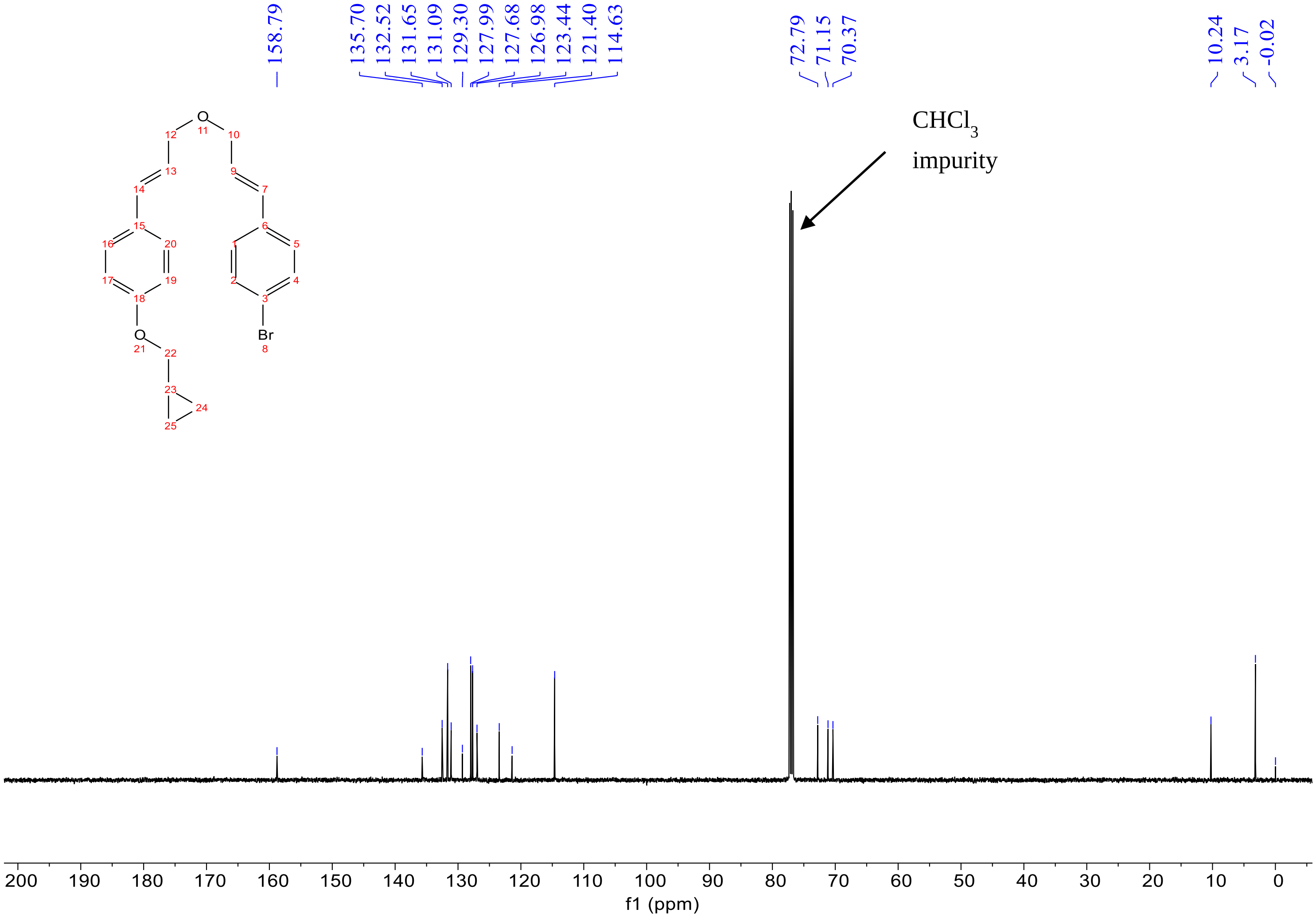

Supplementary Figure 29. ^{13}C NMR of **5e** (126 MHz, Chloroform-*d*)

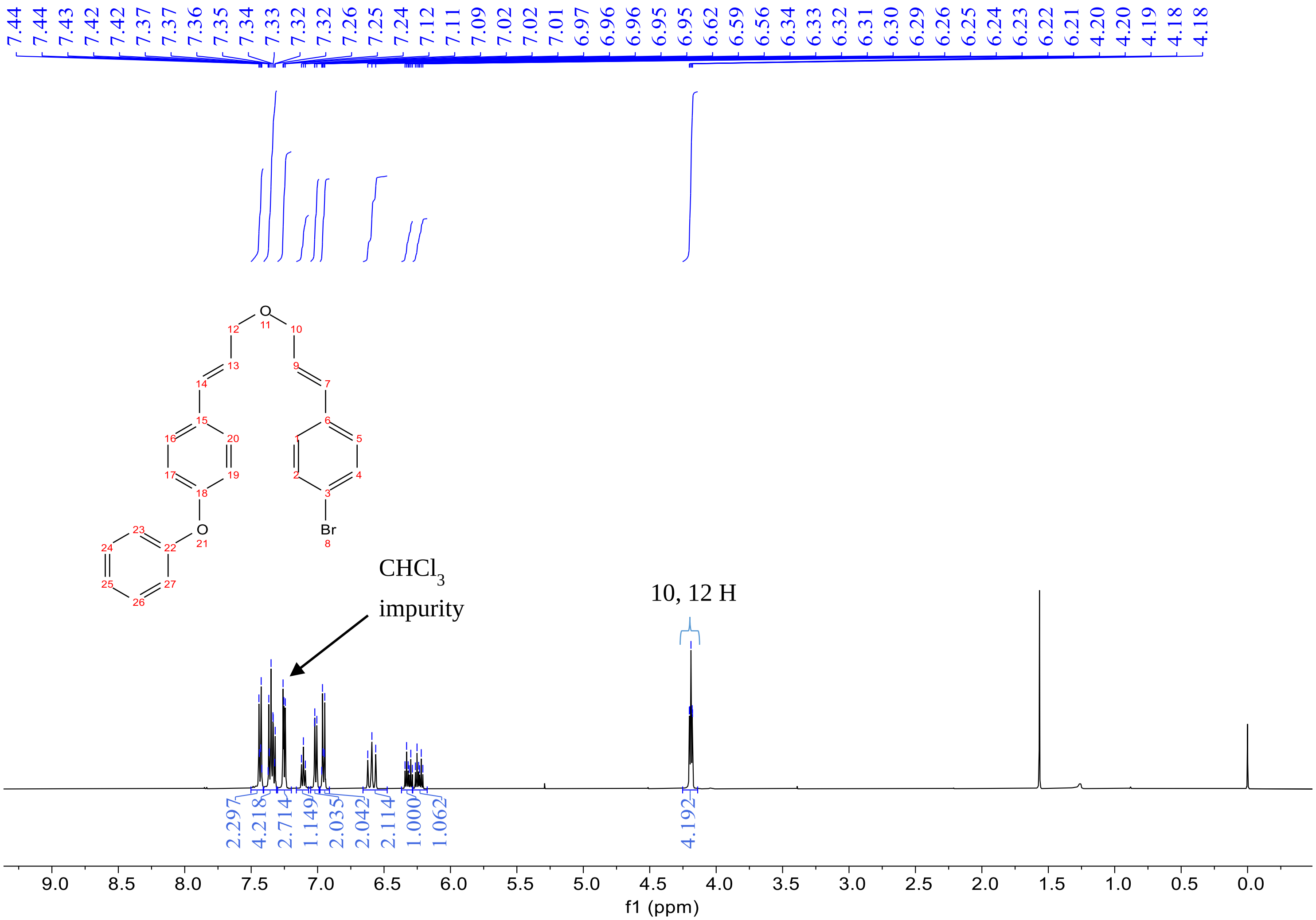

Supplementary Figure 30. ^{1}H NMR of **5f** (500 MHz, Chloroform-*d*)

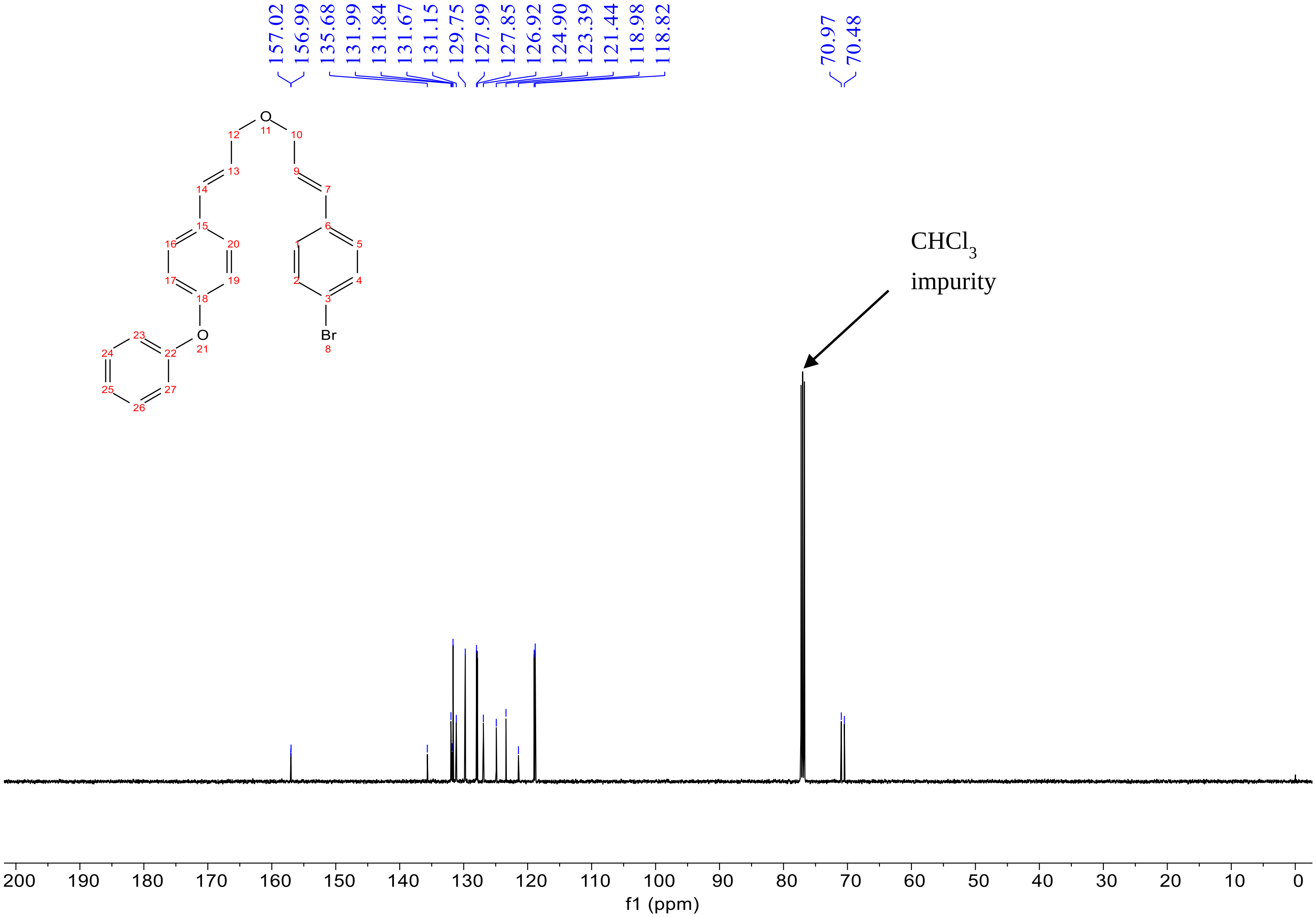

Supplementary Figure 31. ^{13}C NMR of **5f** (126 MHz, Chloroform-*d*)

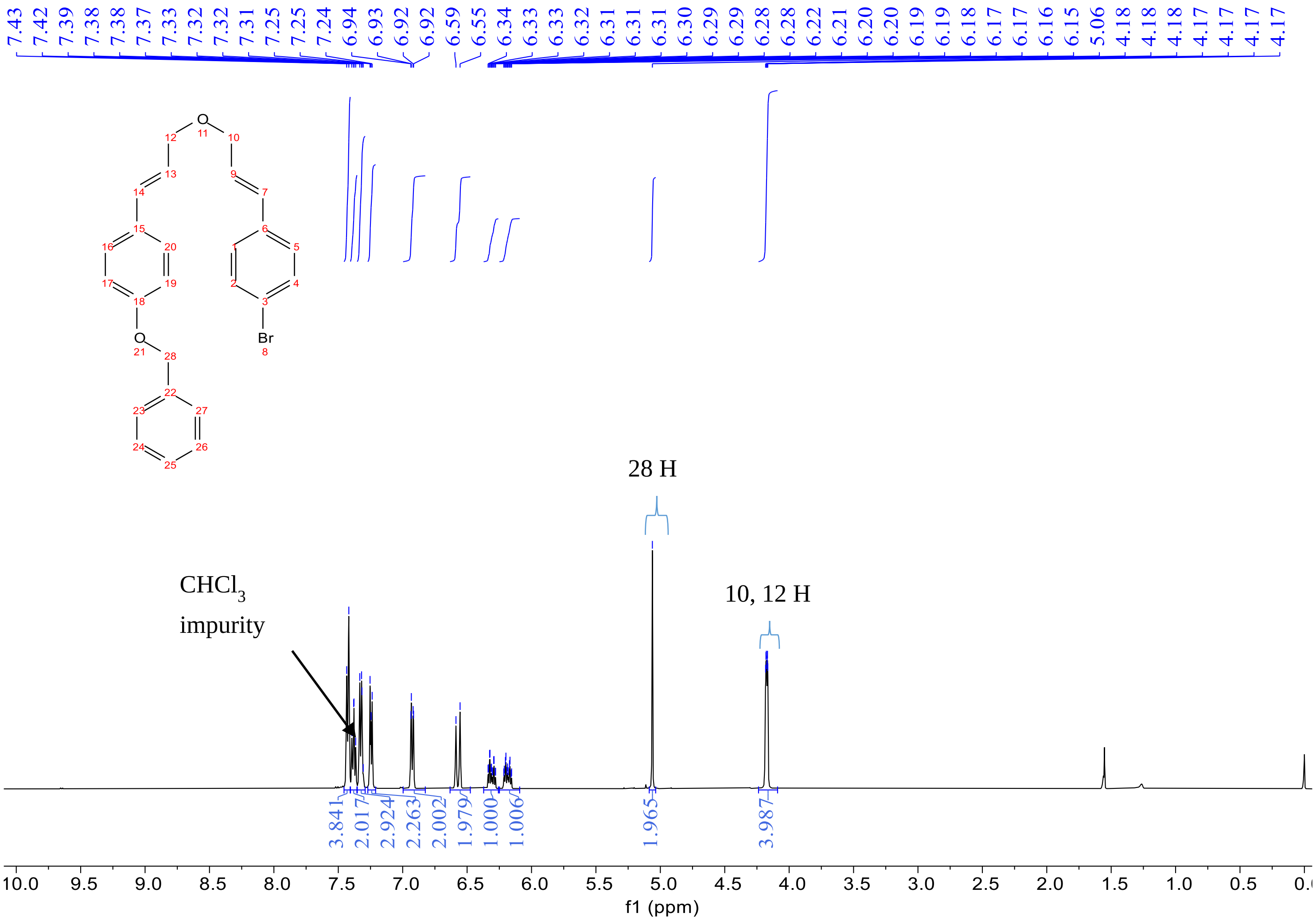

Supplementary Figure 32. ^{1}H NMR of **5g** (500 MHz, Chloroform-*d*)

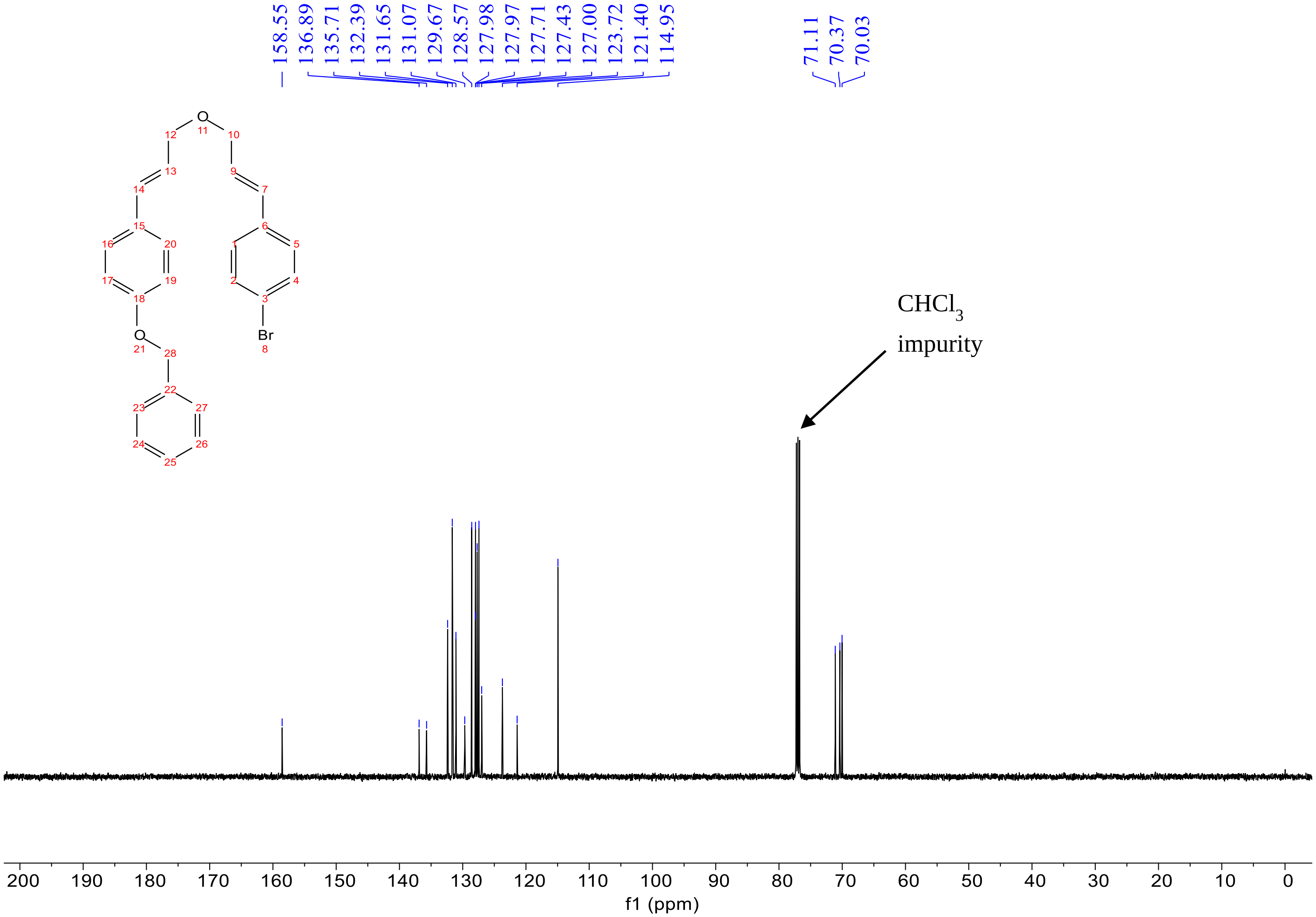

Supplementary Figure 33. ^{13}C NMR of **5g** (126 MHz, Chloroform-*d*)

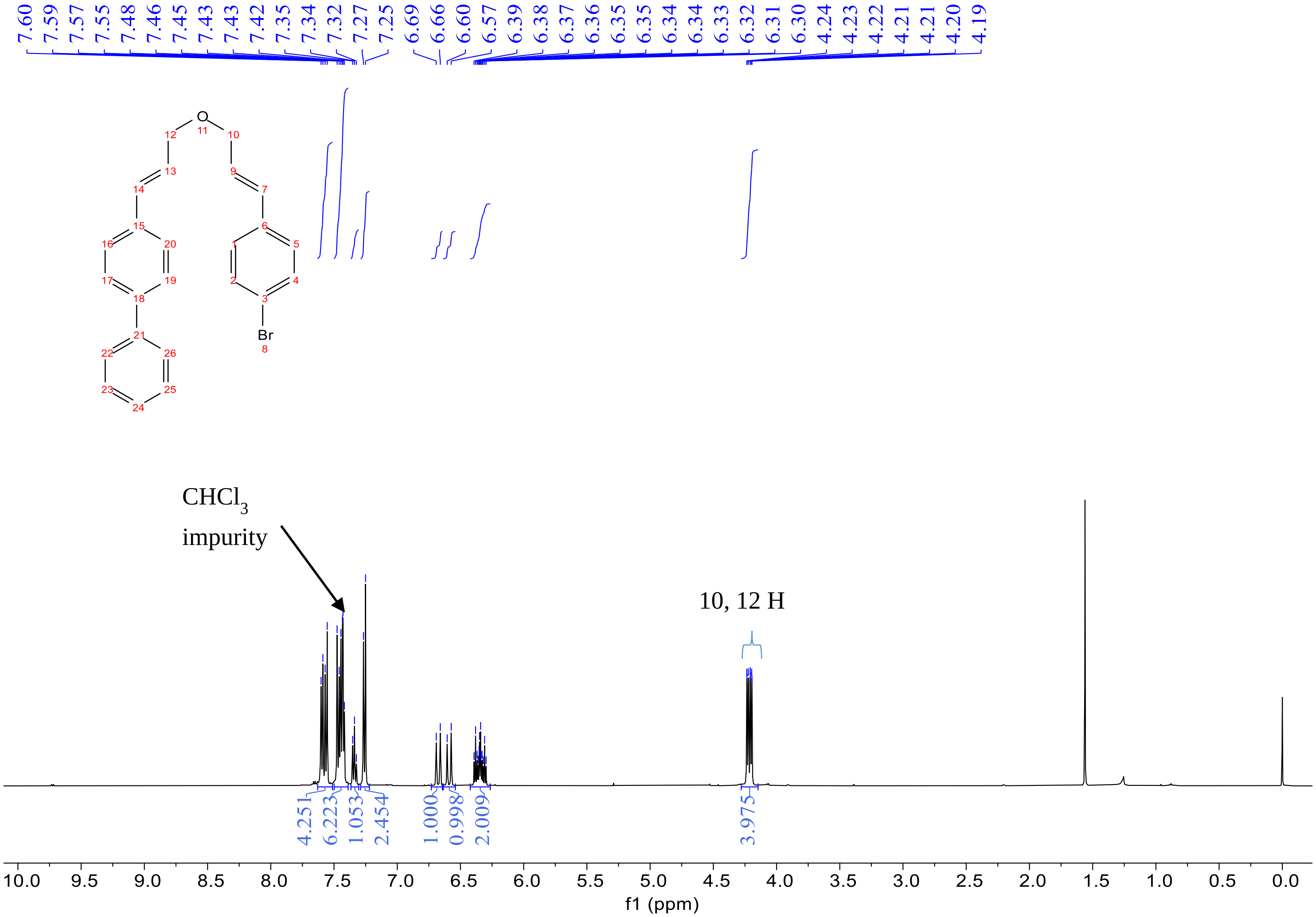

Supplementary Figure 34. ^{1}H NMR of **5h** (500 MHz, Chloroform-*d*)

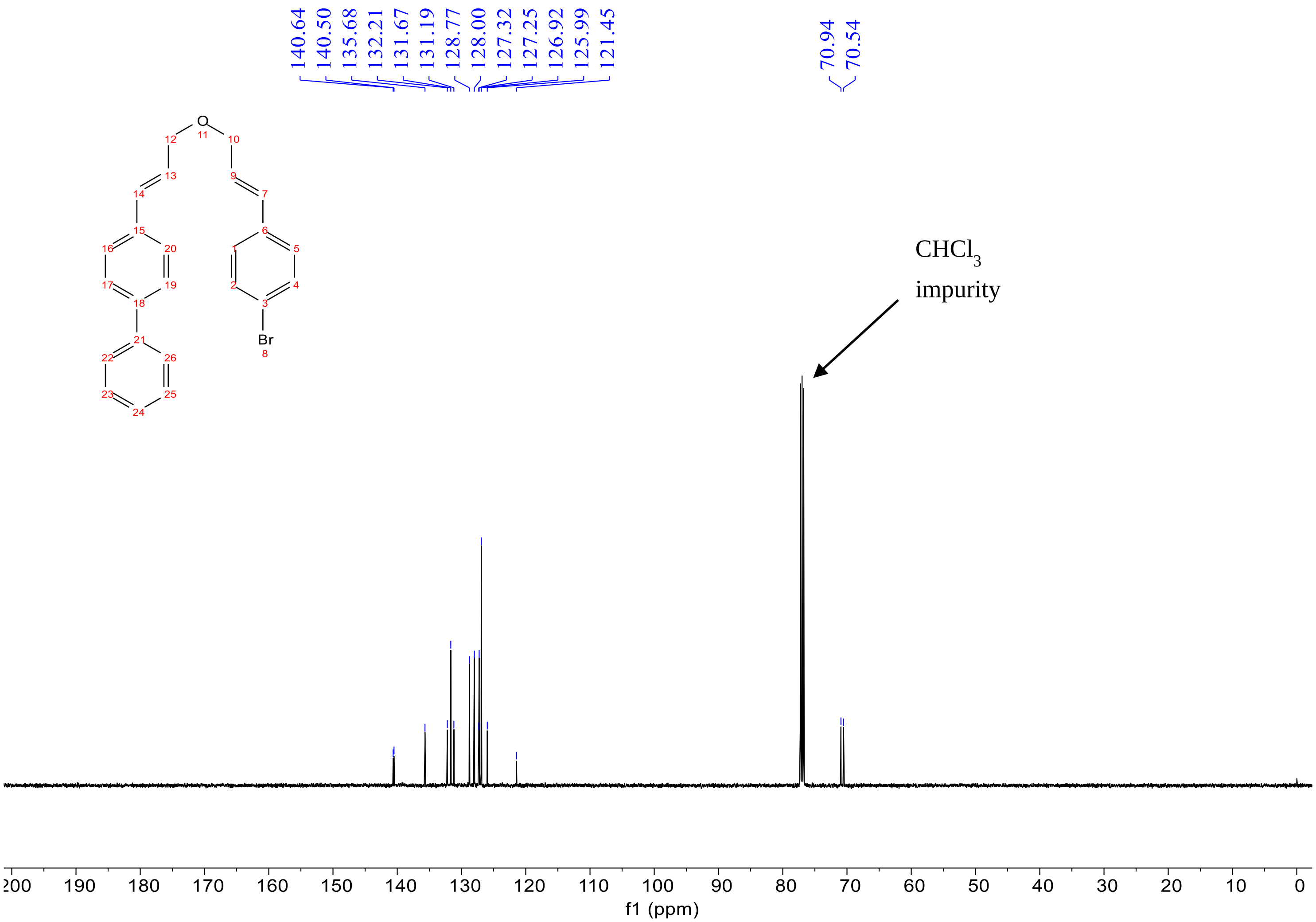

Supplementary Figure 35. ^{13}C NMR of **5h** (126 MHz, Chloroform-*d*)

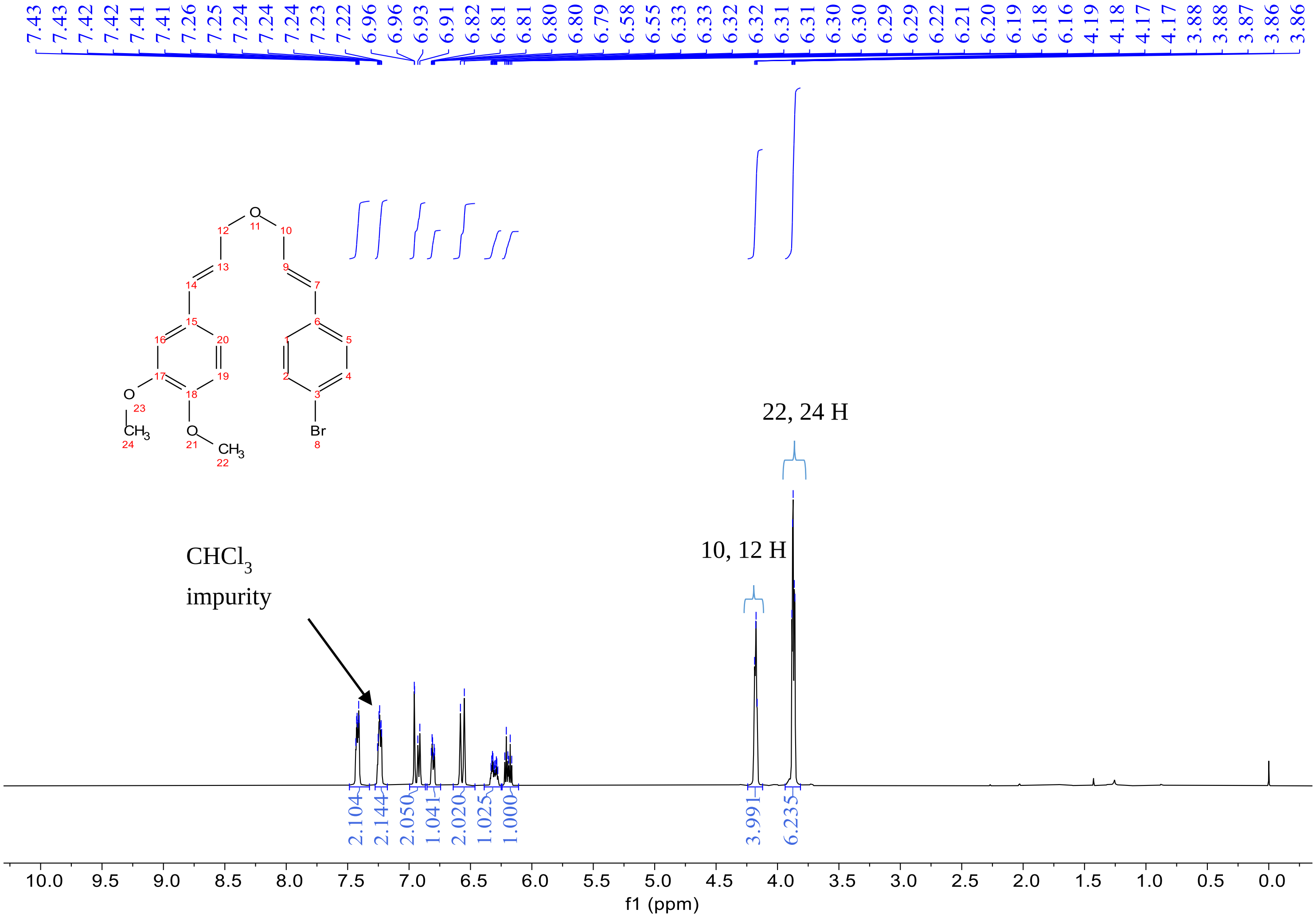

Supplementary Figure 36. ^{1}H NMR of **5i** (500 MHz, Chloroform-*d*)

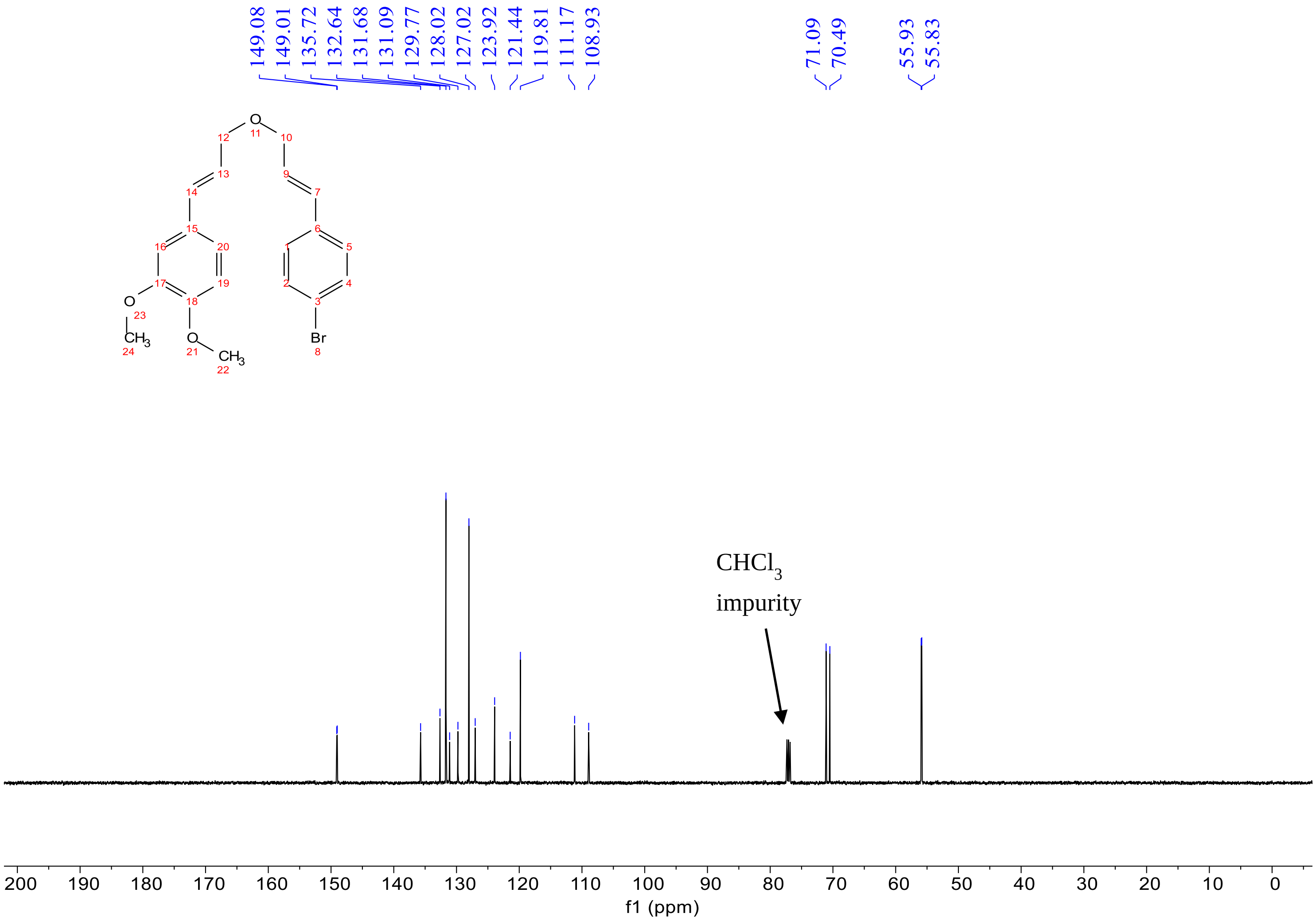

Supplementary Figure 37. ^{13}C NMR of **5i** (126 MHz, Chloroform-*d*)

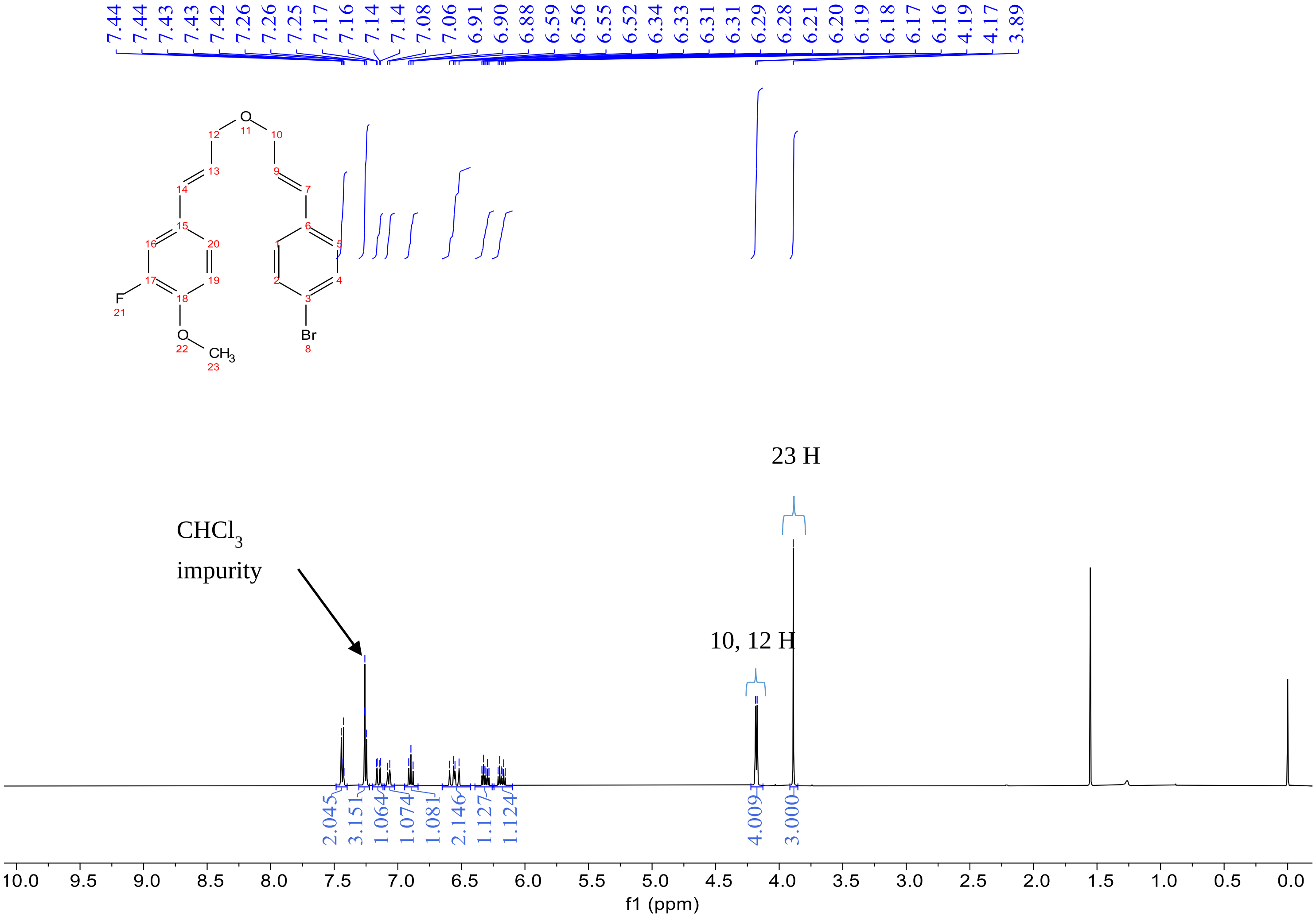

Supplementary Figure 38. ^{1}H NMR of **5j** (500 MHz, Chloroform-*d*)

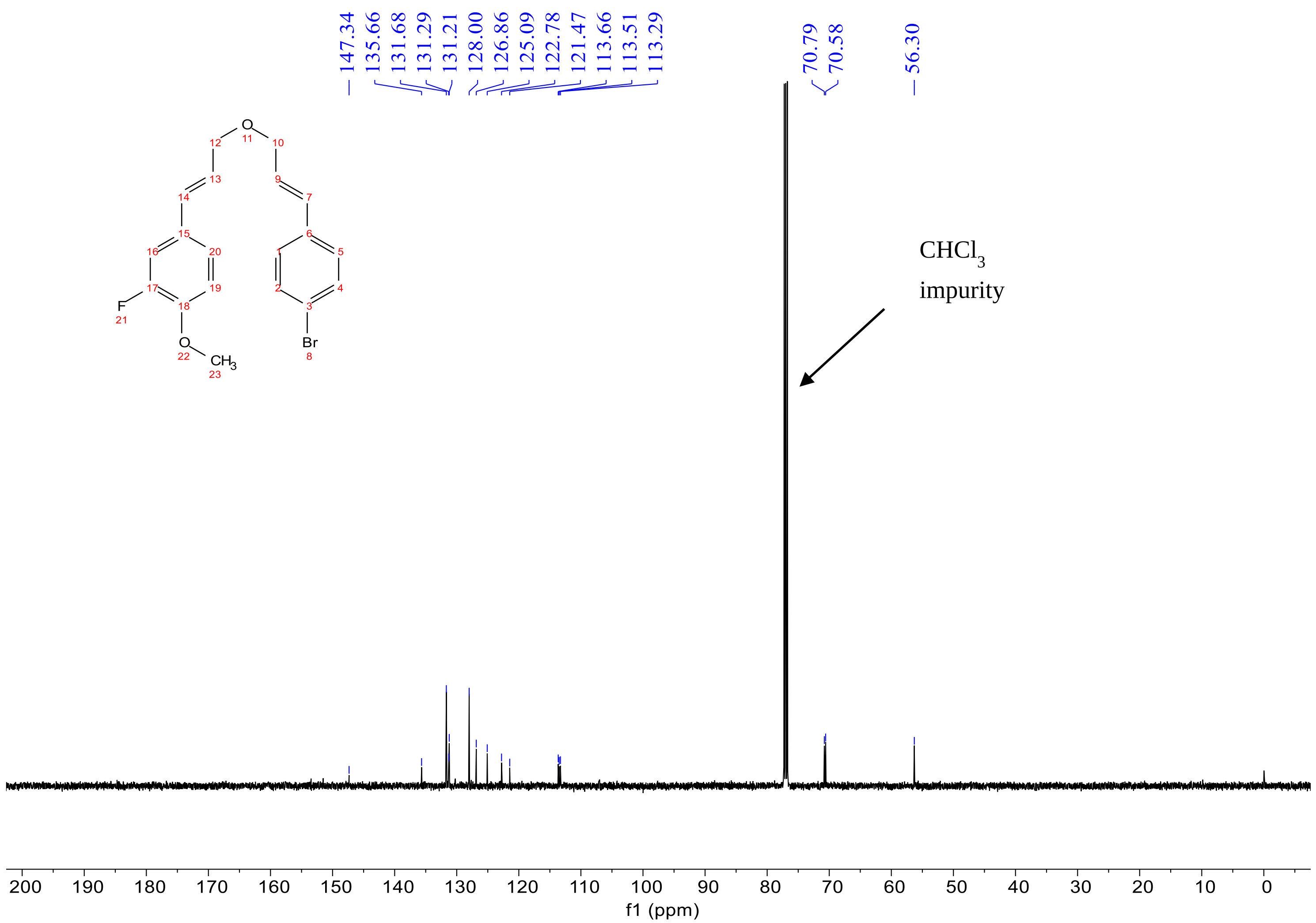

Supplementary Figure 39. ^{13}C NMR of **5j** (126 MHz, Chloroform-*d*)

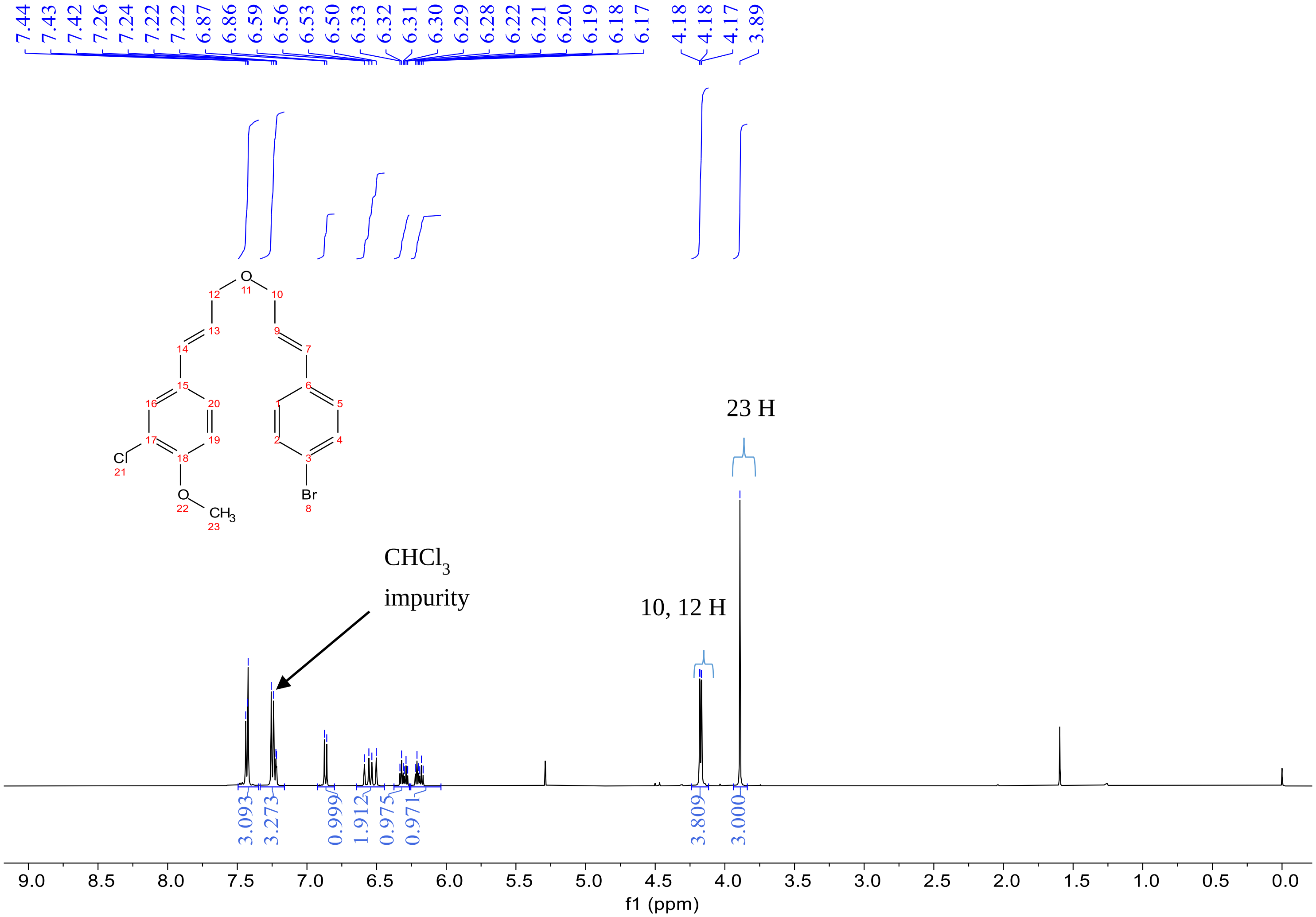

Supplementary Figure 40. ^{1}H NMR of **5k** (500 MHz, Chloroform-*d*)

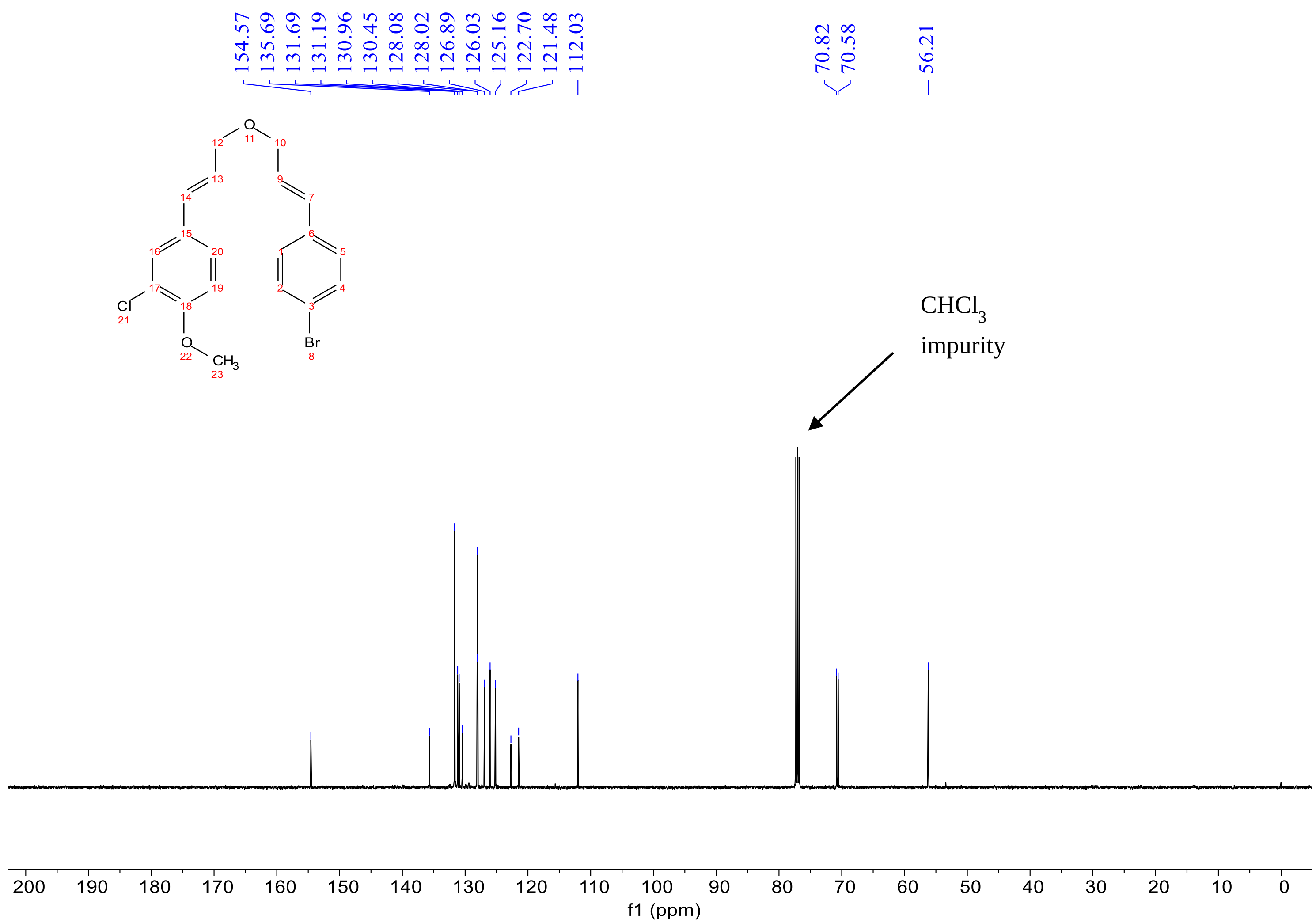

Supplementary Figure 41. ^{13}C NMR of **5k** (126 MHz, Chloroform-*d*)

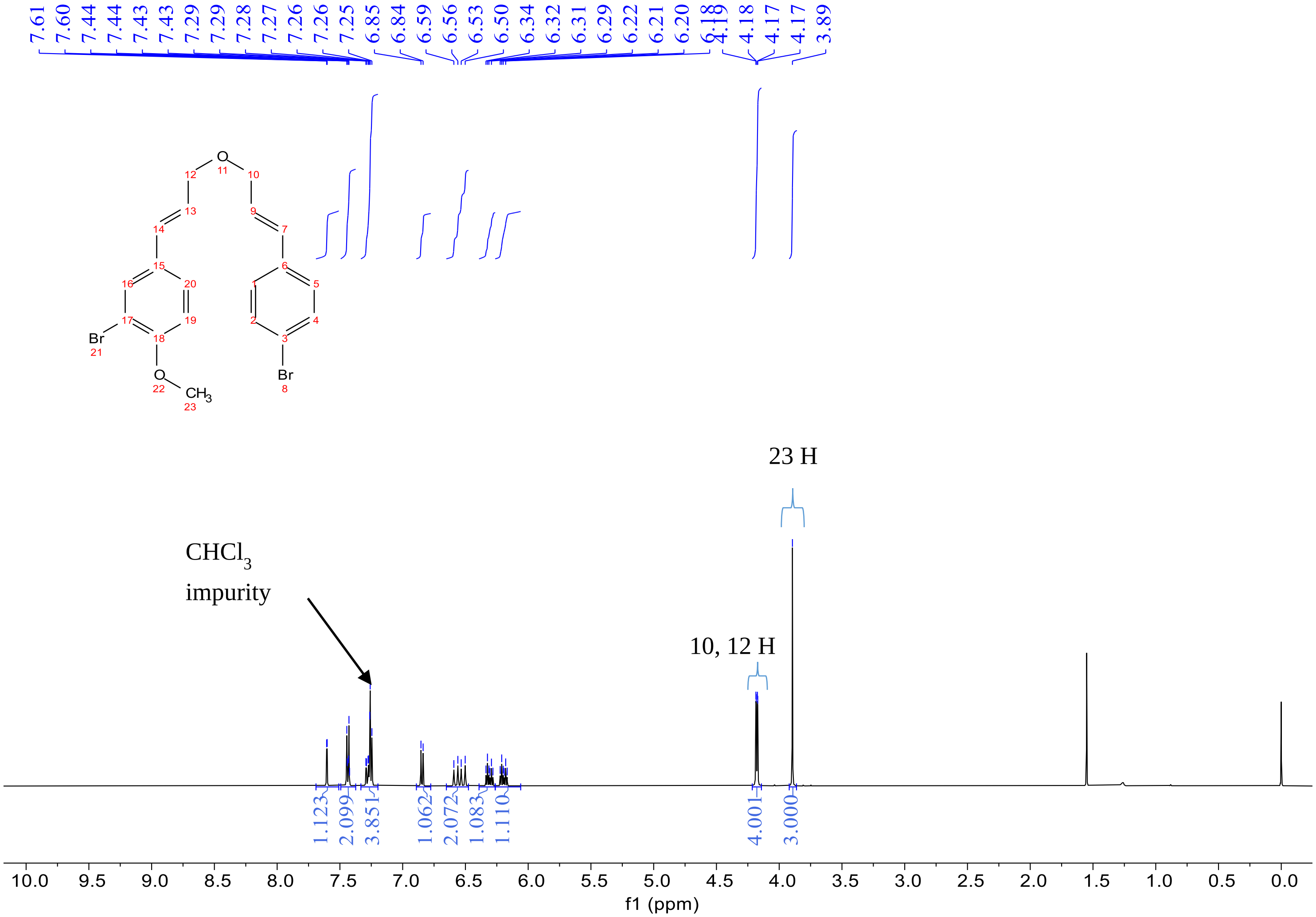

Supplementary Figure 42. ^{1}H NMR of **5l** (500 MHz, Chloroform-*d*)

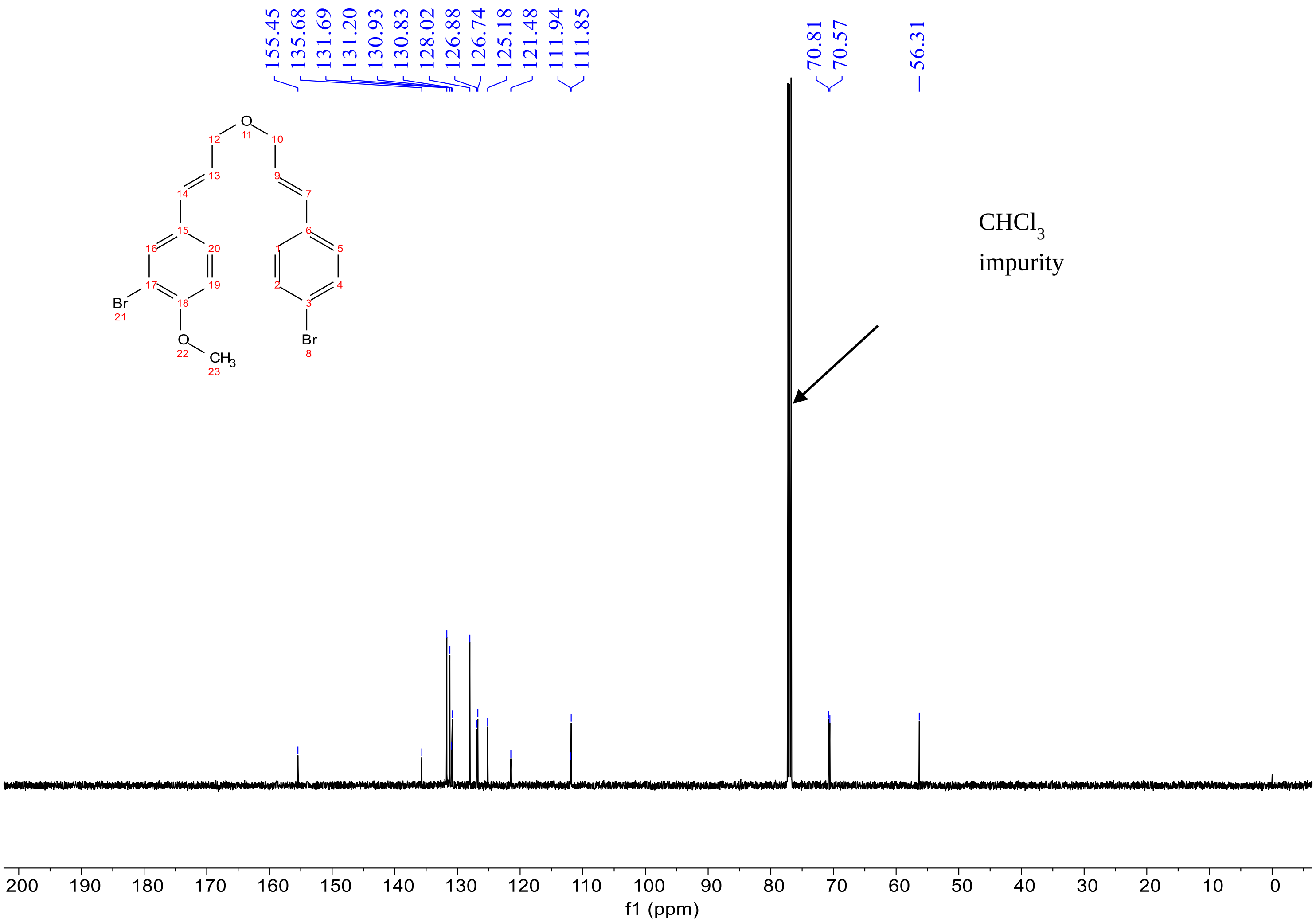

Supplementary Figure 43. ^{13}C NMR of **5l** (126 MHz, Chloroform-*d*)

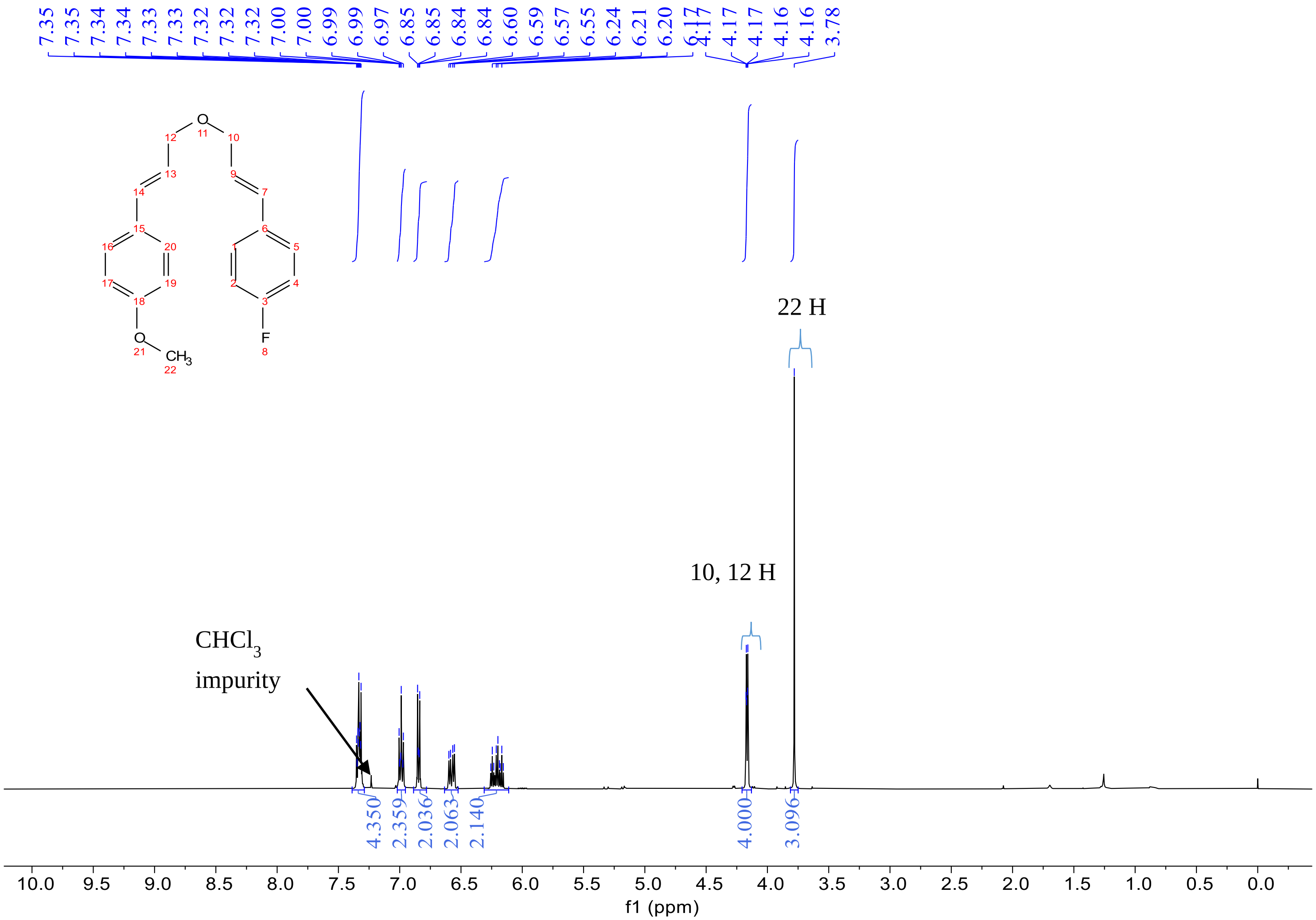

Supplementary Figure 44. ^{1}H NMR of **5m** (500 MHz, Chloroform-*d*)

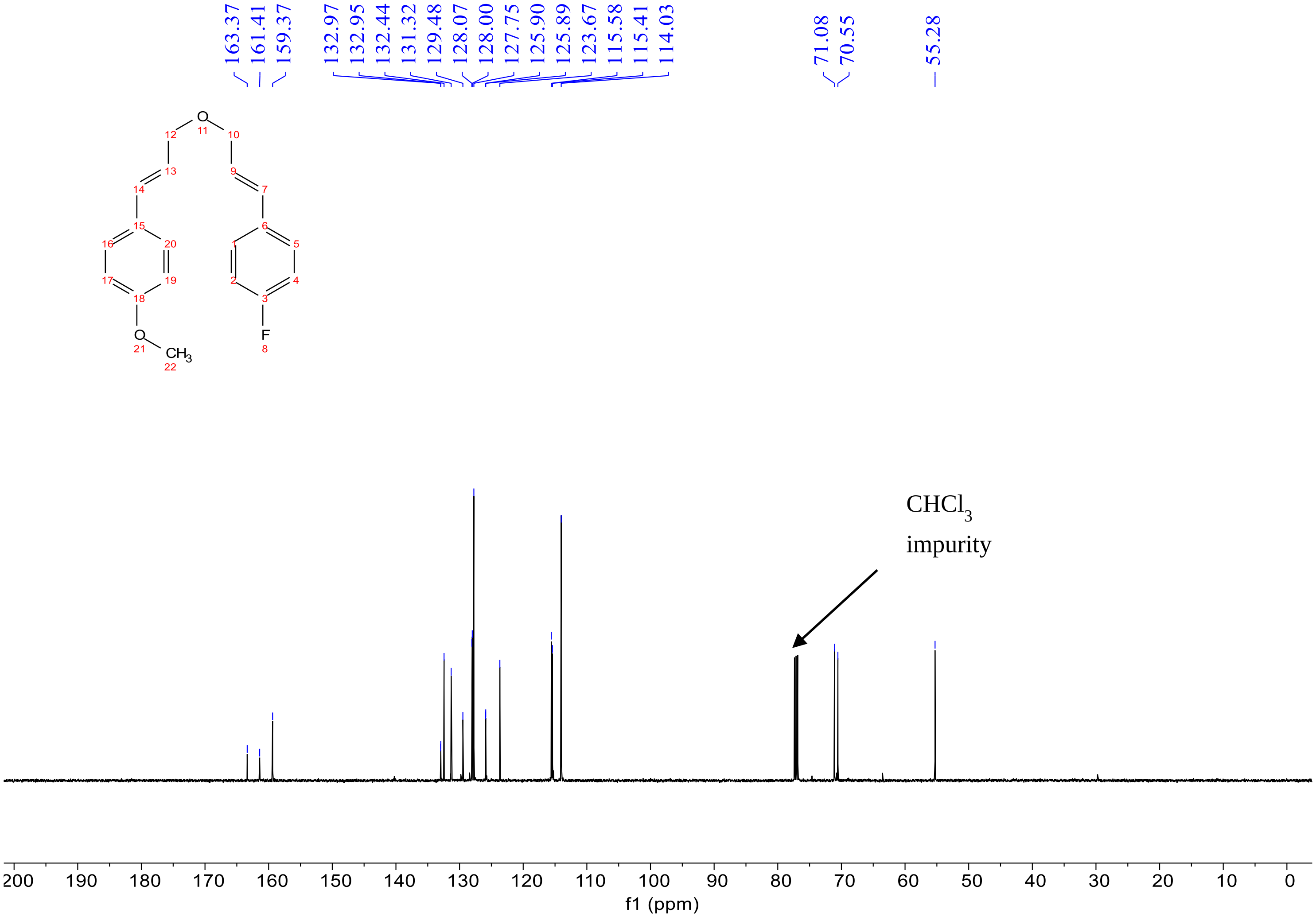

Supplementary Figure 45. ^{13}C NMR of **5m** (126 MHz, Chloroform-*d*)

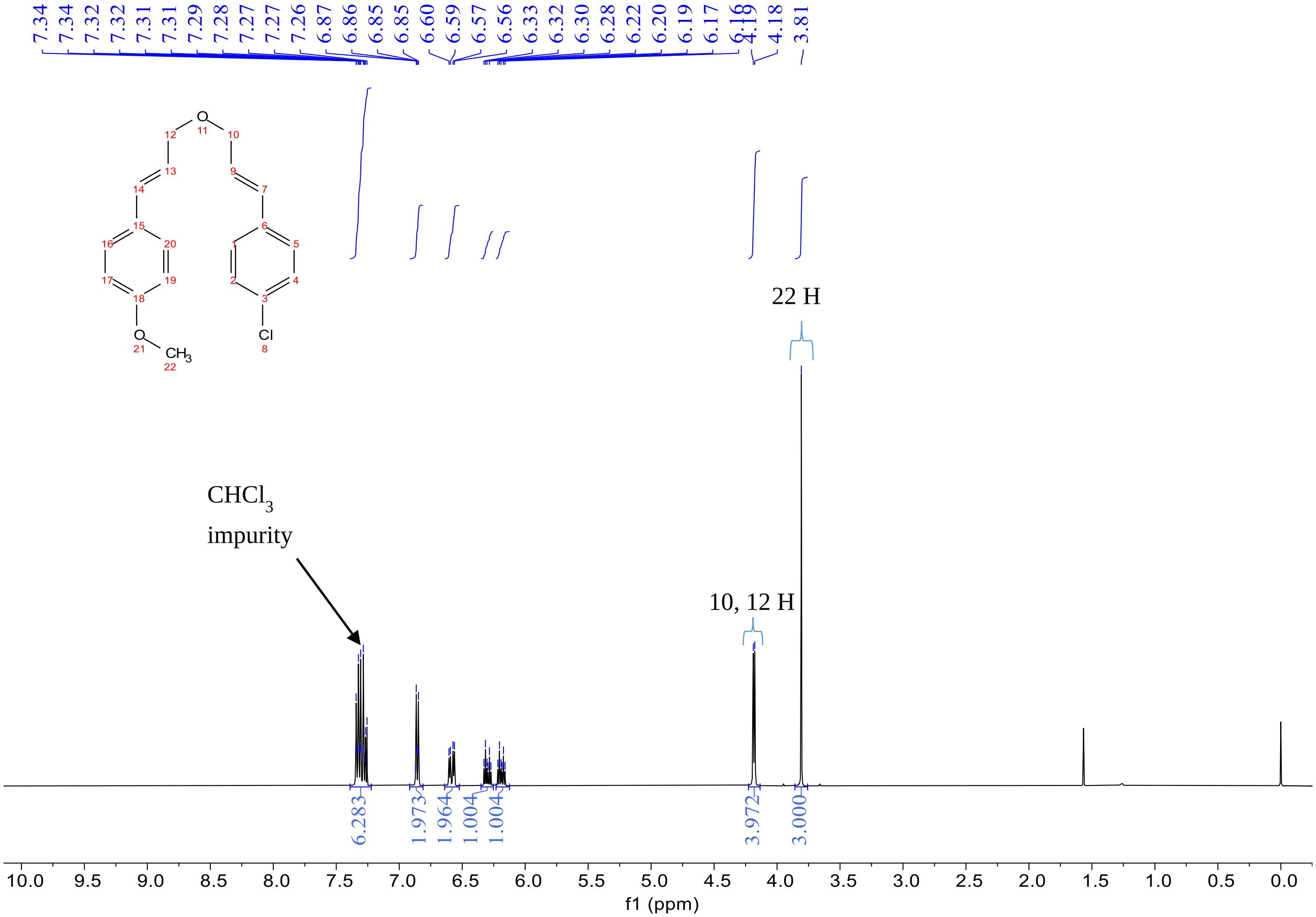

Supplementary Figure 46. ^{1}H NMR of **5n** (500 MHz, Chloroform-*d*)

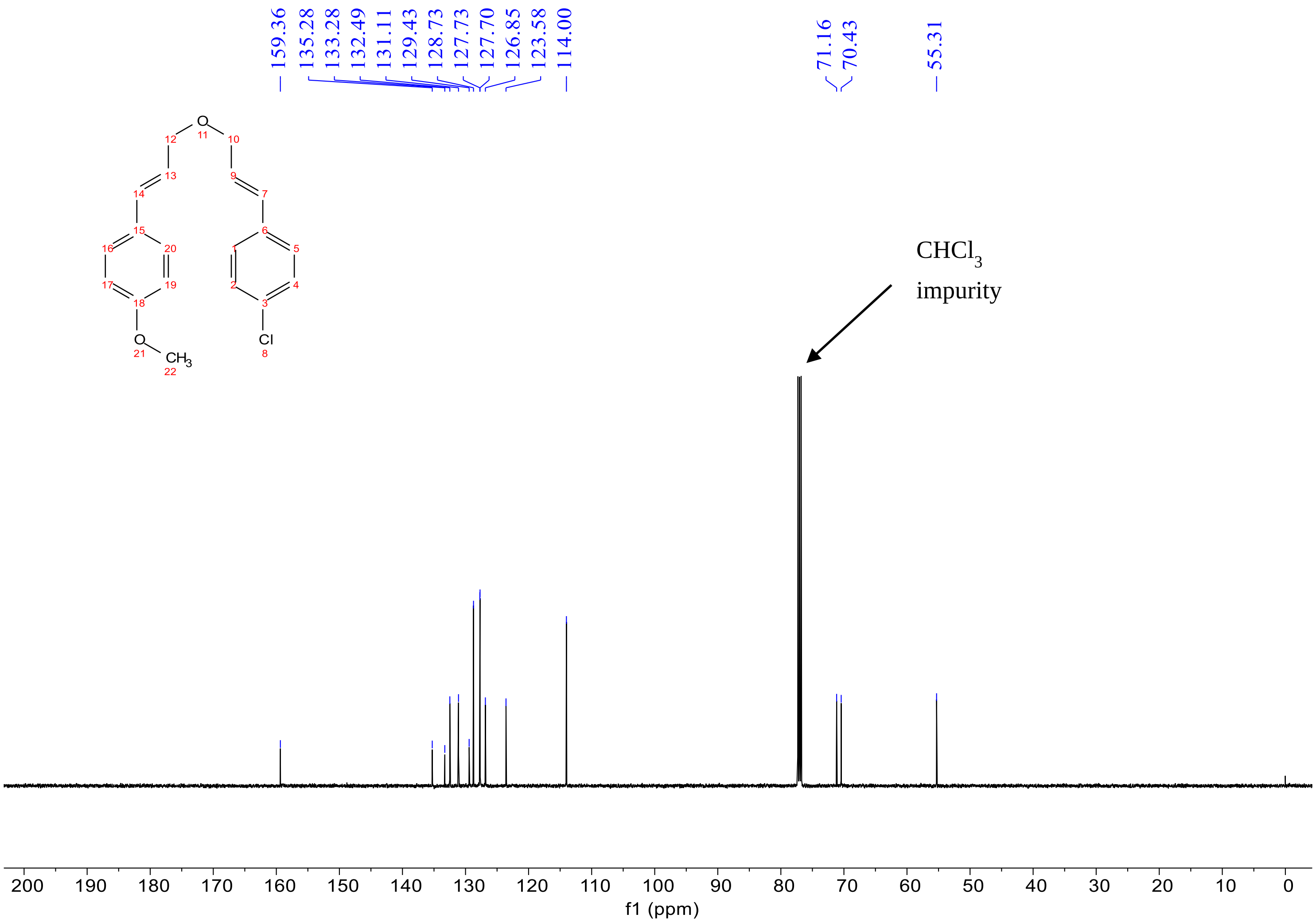

Supplementary Figure 47. ^{13}C NMR of **5n** (126 MHz, Chloroform-*d*)

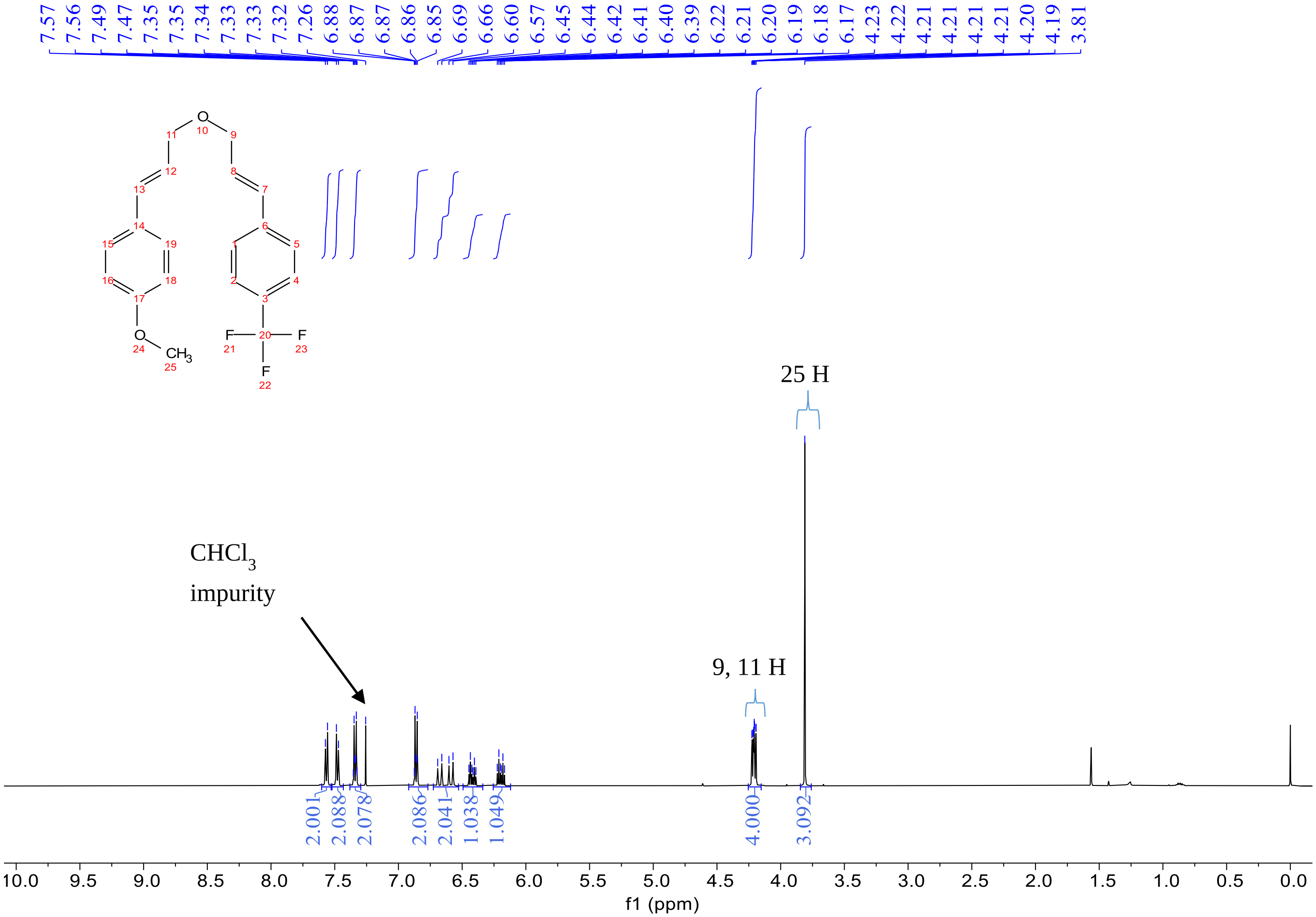

Supplementary Figure 48. ^{1}H NMR of **5o** (500 MHz, Chloroform-*d*)

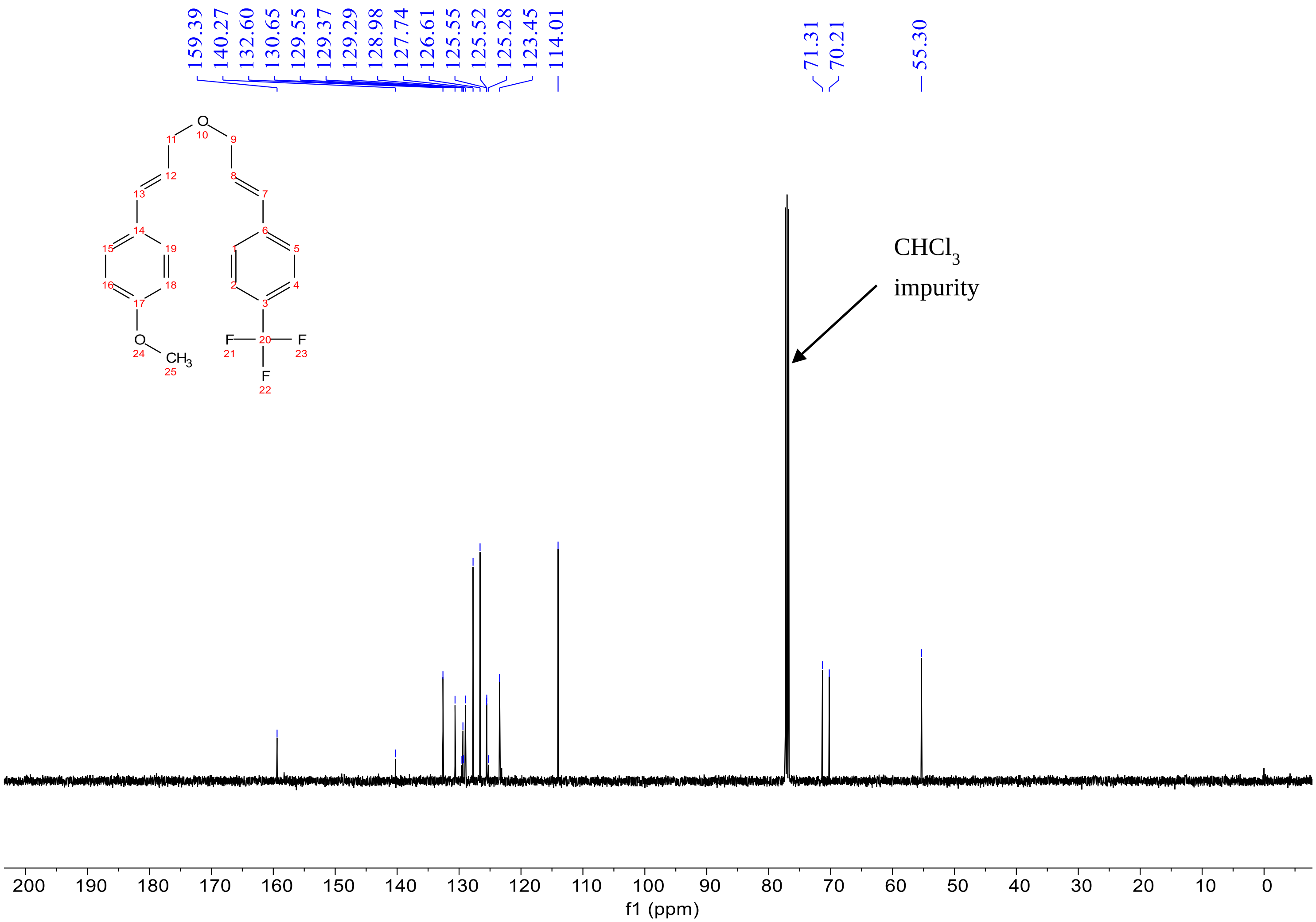

Supplementary Figure 49. ^{13}C NMR of **5o** (126 MHz, Chloroform-*d*)

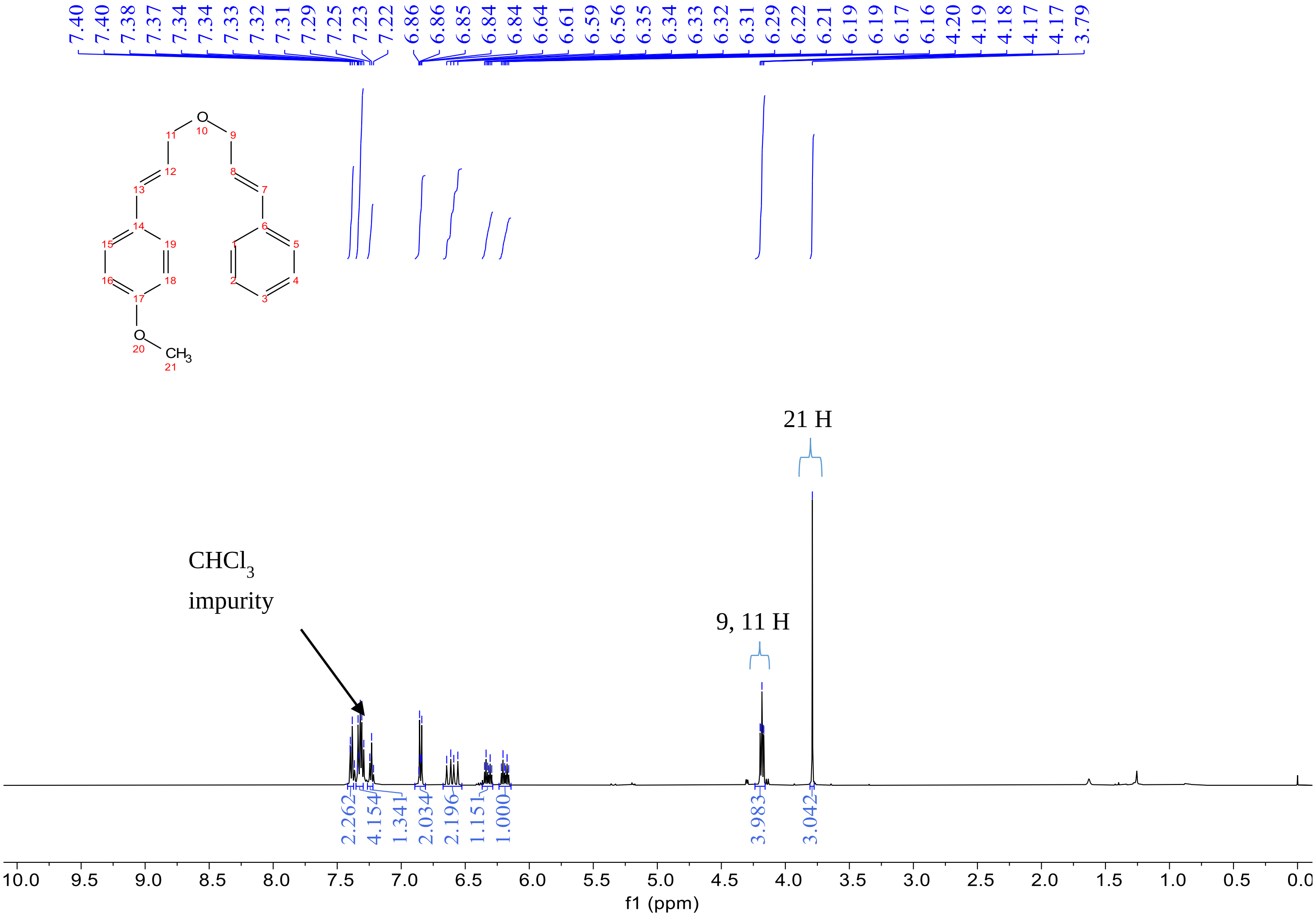

Supplementary Figure 50. ^{1}H NMR of **5p** (500 MHz, Chloroform-*d*)

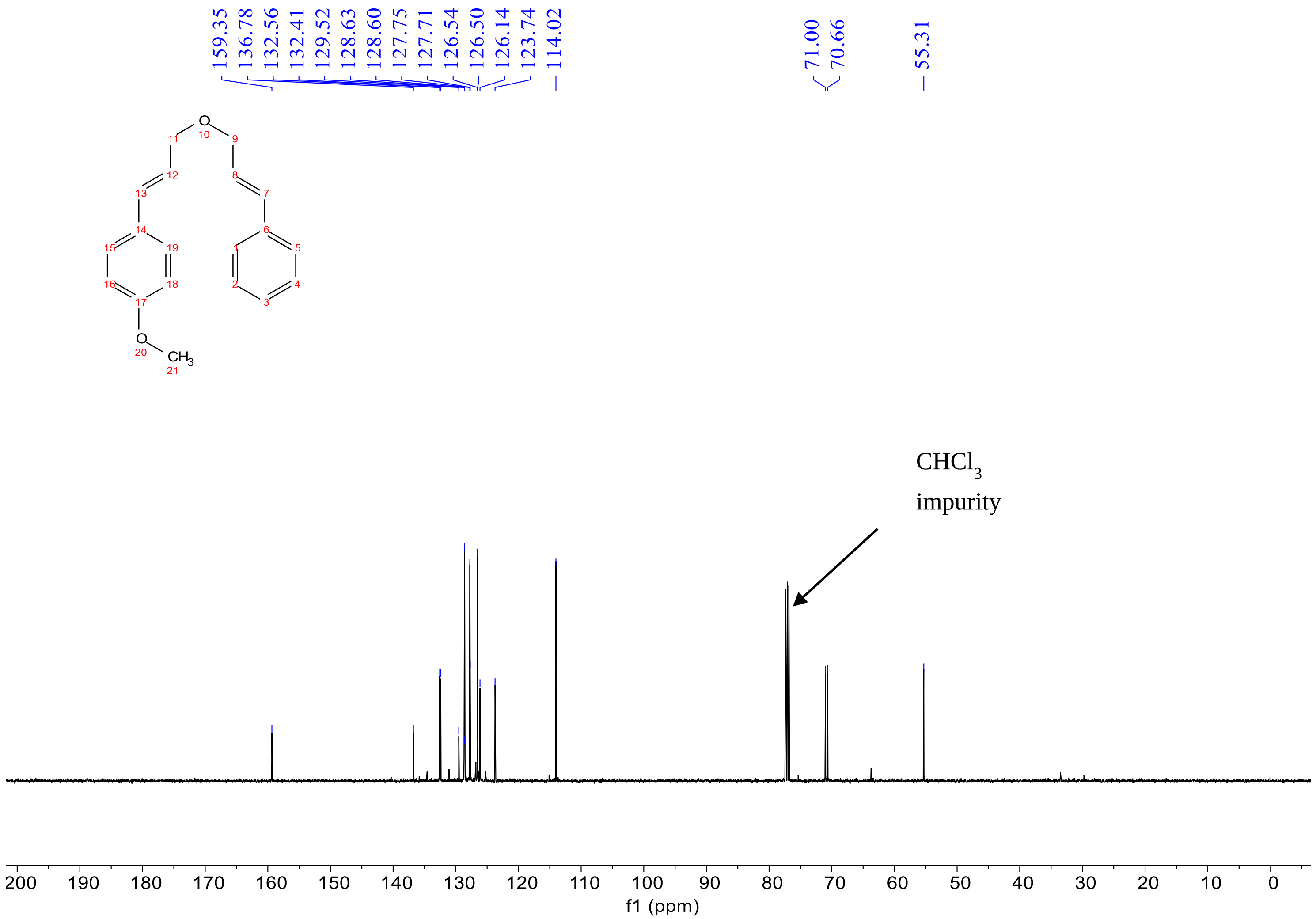

Supplementary Figure 51. ^{13}C NMR of **5p** (126 MHz, Chloroform-*d*)

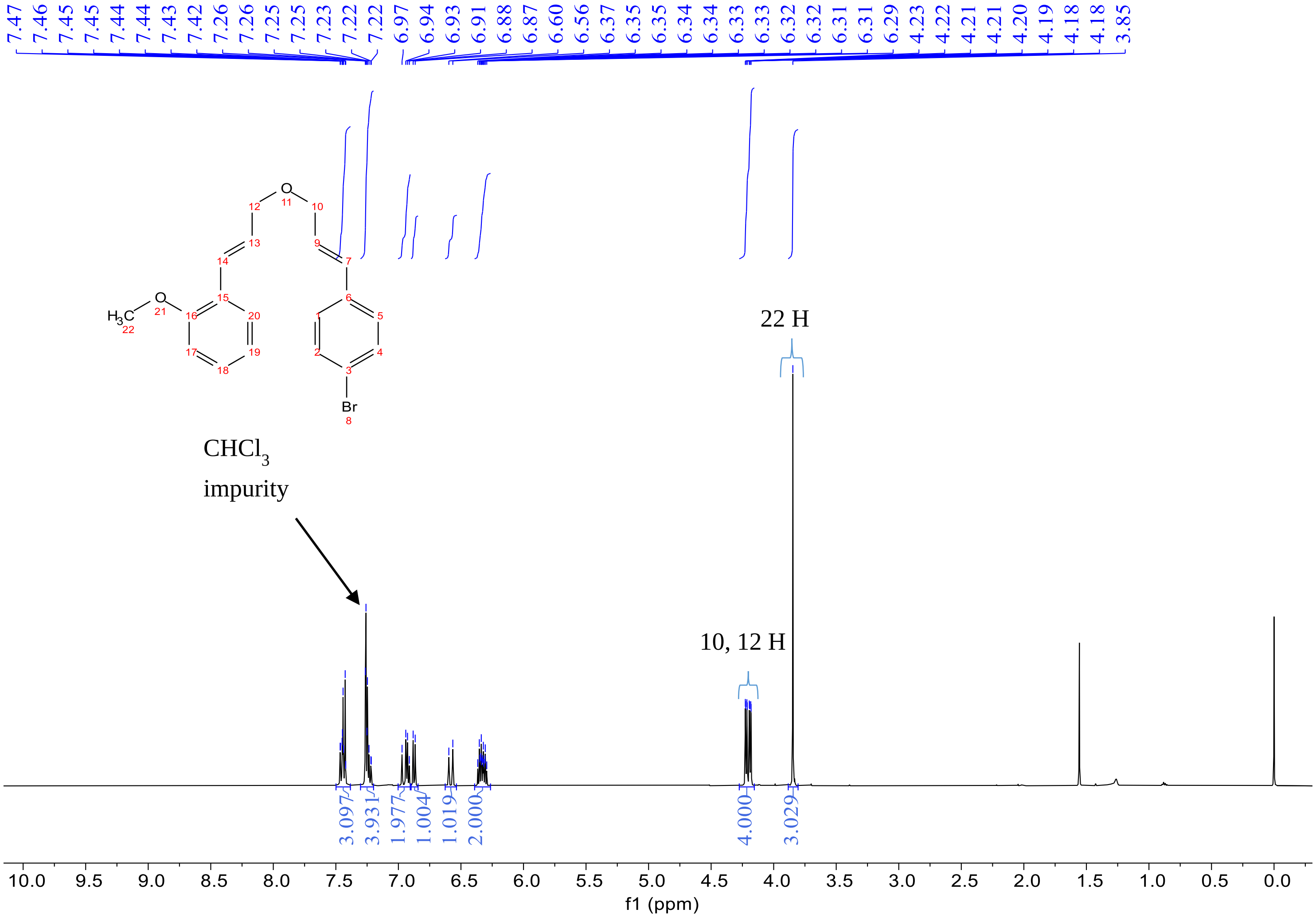

Supplementary Figure 52. ^{1}H NMR of **5q** (500 MHz, Chloroform-*d*)

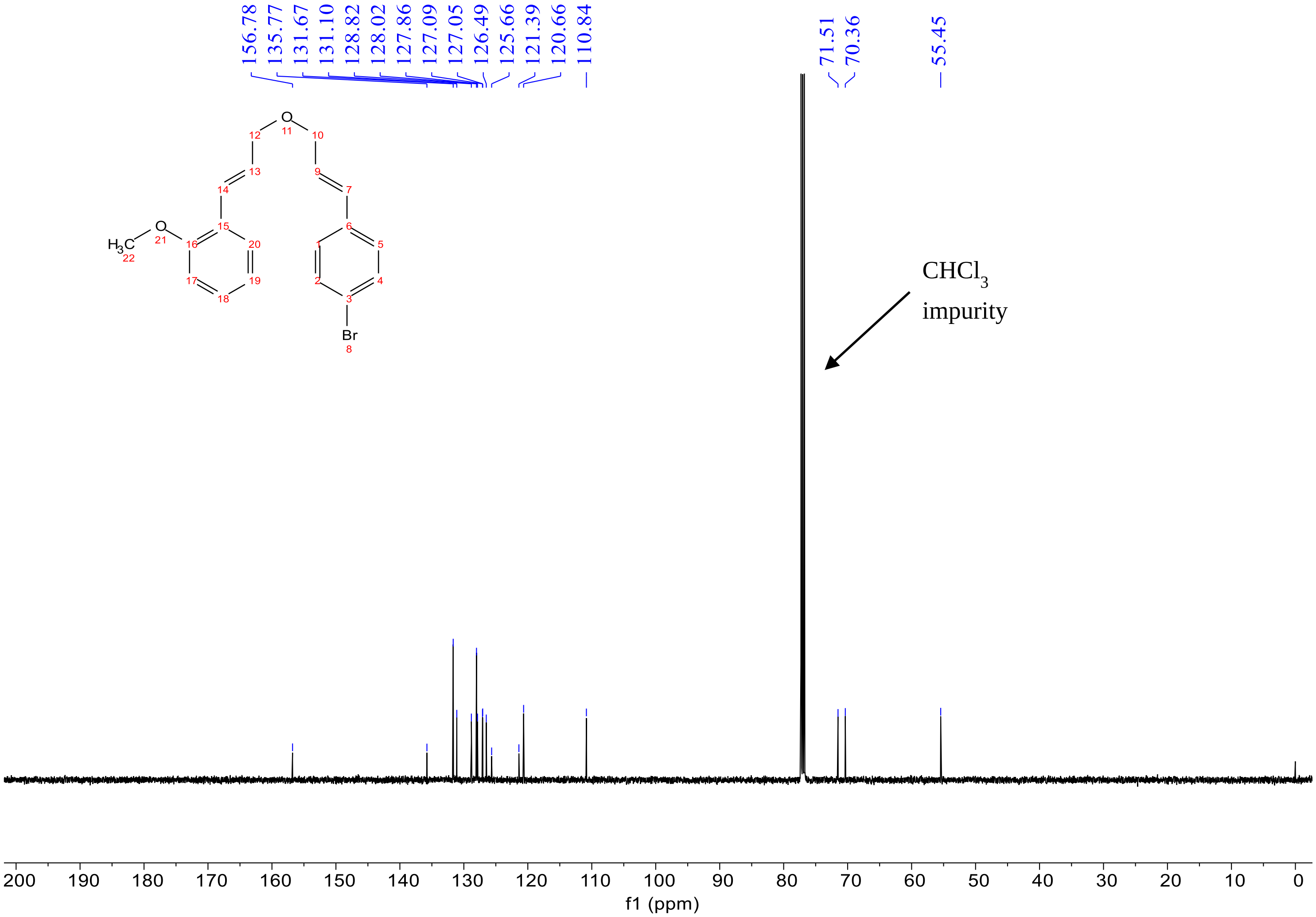

Supplementary Figure 53. ^{13}C NMR of **5q** (126 MHz, Chloroform-*d*)

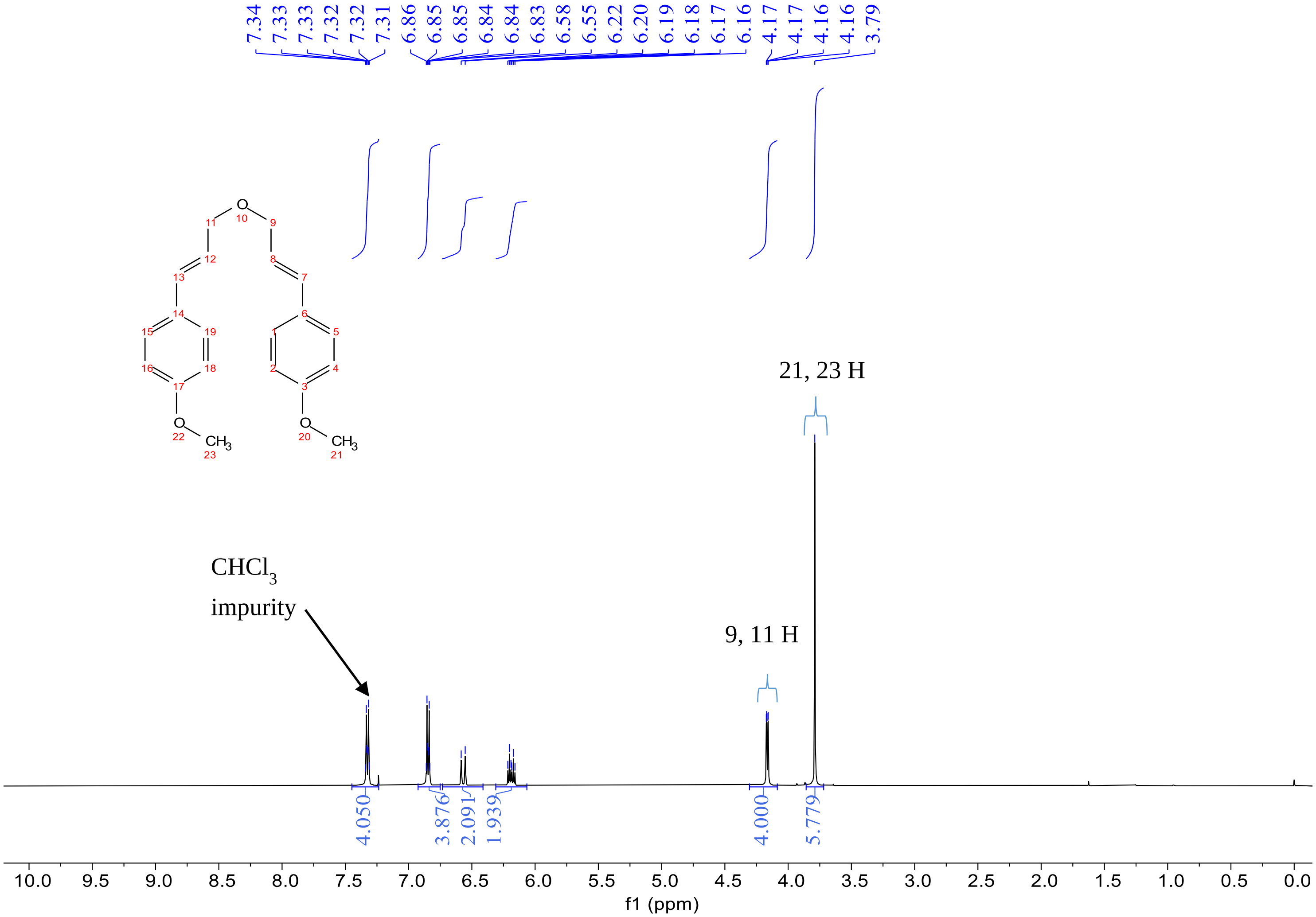

Supplementary Figure 54. ^{1}H NMR of **5r** (500 MHz, Chloroform-*d*)

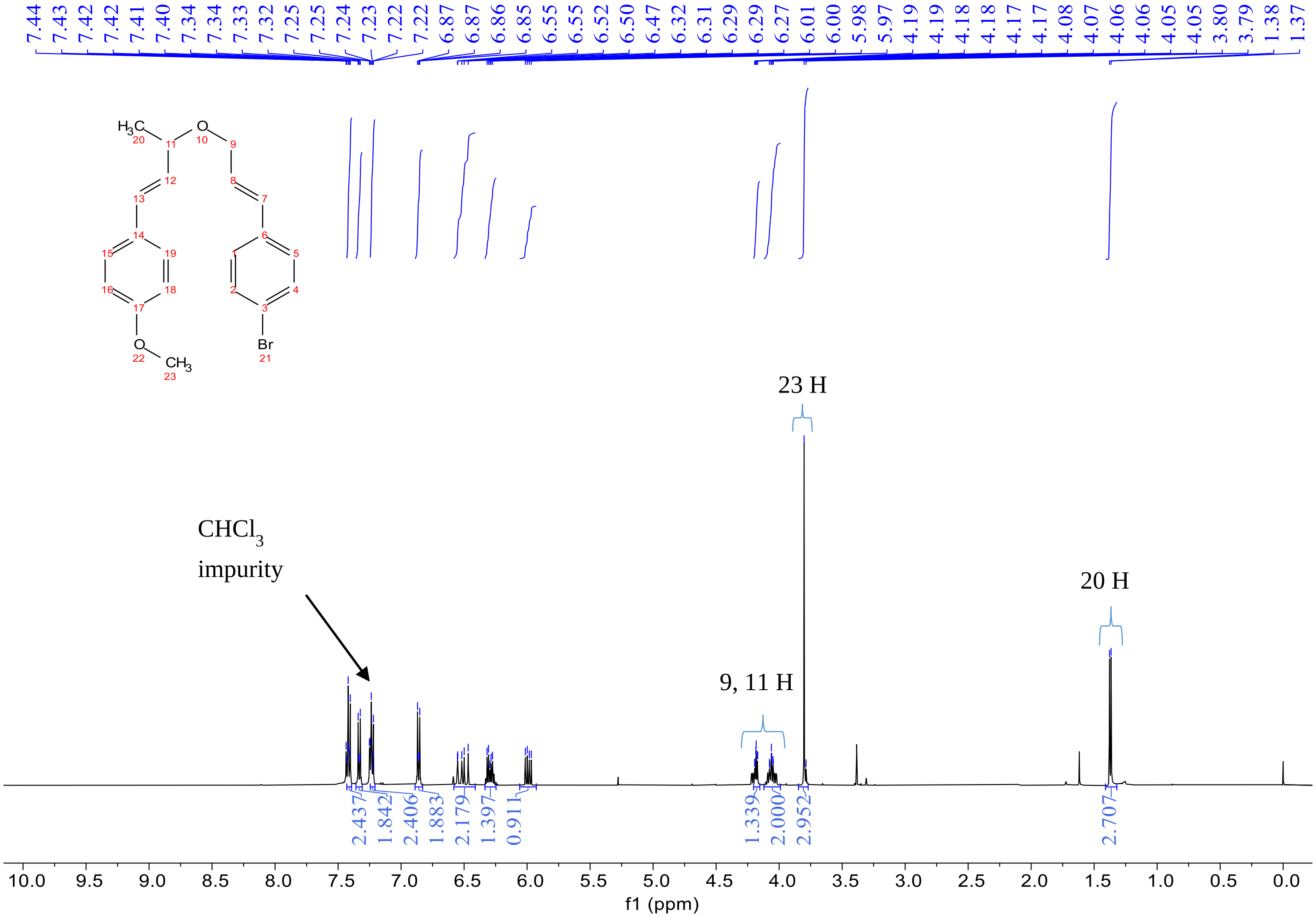

Supplementary Figure 55. ^{1}H NMR of **5s** (500 MHz, Chloroform-*d*)

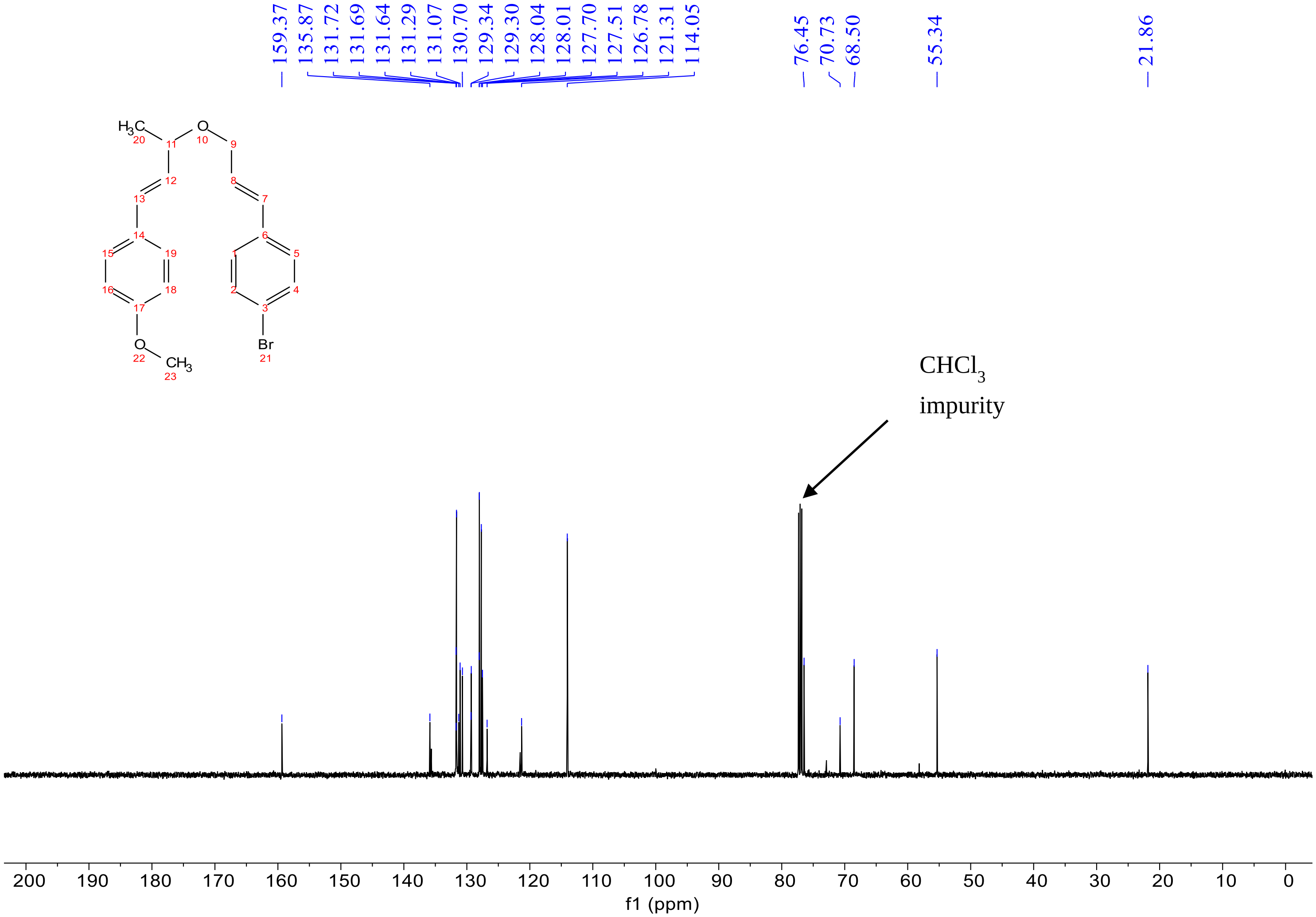

Supplementary Figure 56. ^{13}C NMR of **5s** (126 MHz, Chloroform-*d*)

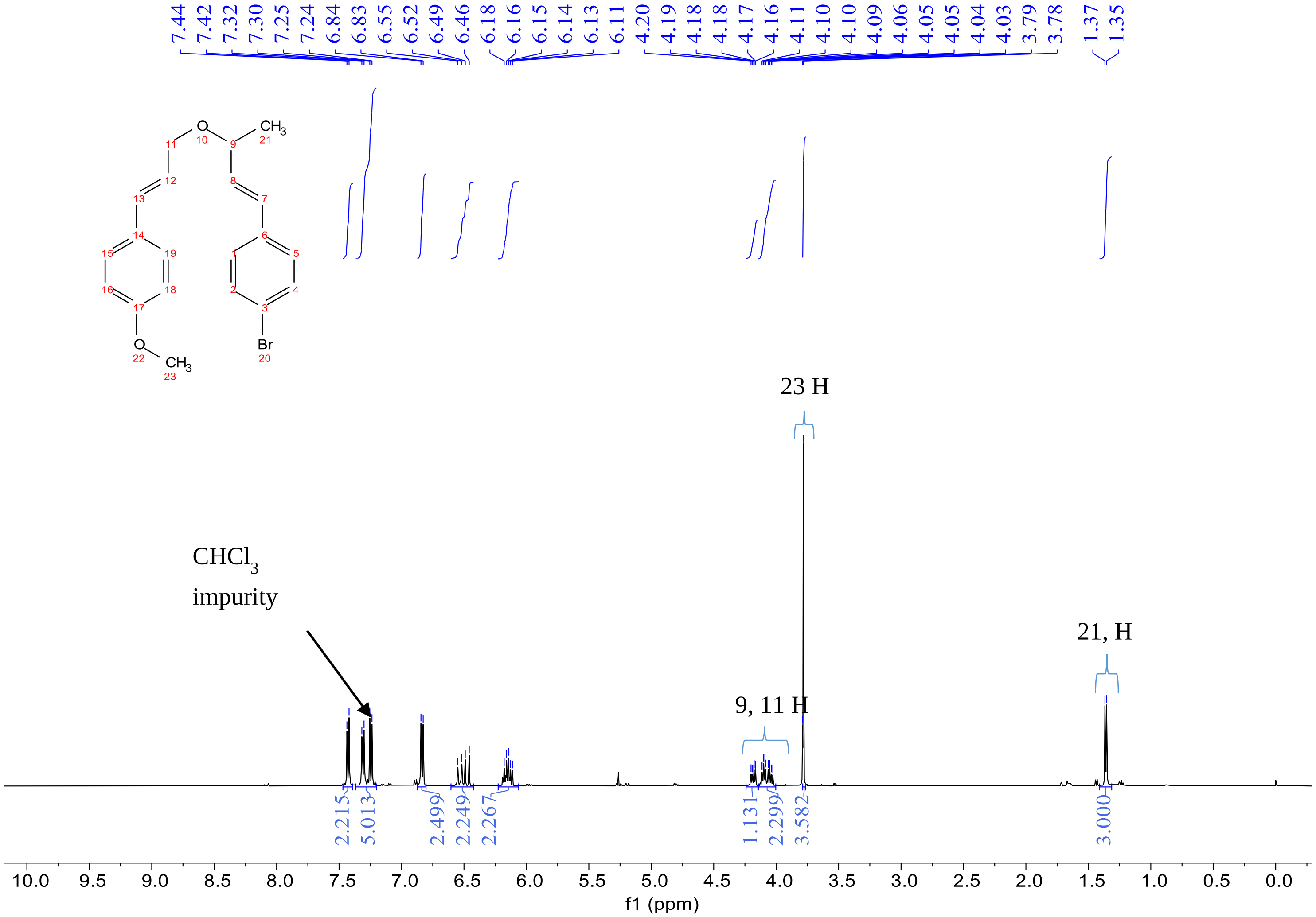

Supplementary Figure 57. ^{1}H NMR of **5t** (500 MHz, Chloroform-*d*)

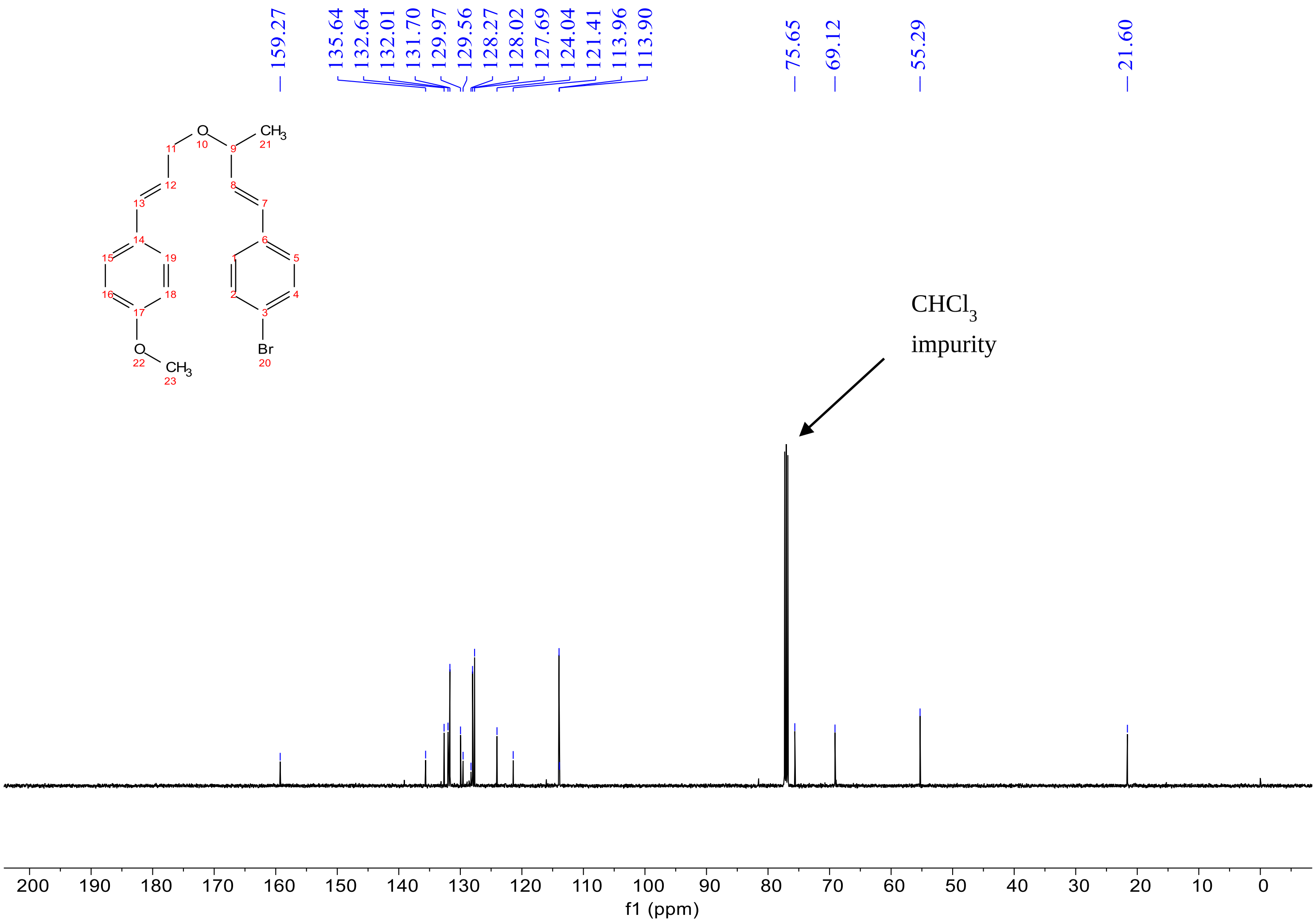

Supplementary Figure 58. ^{13}C NMR of **5t** (126 MHz, Chloroform-*d*)

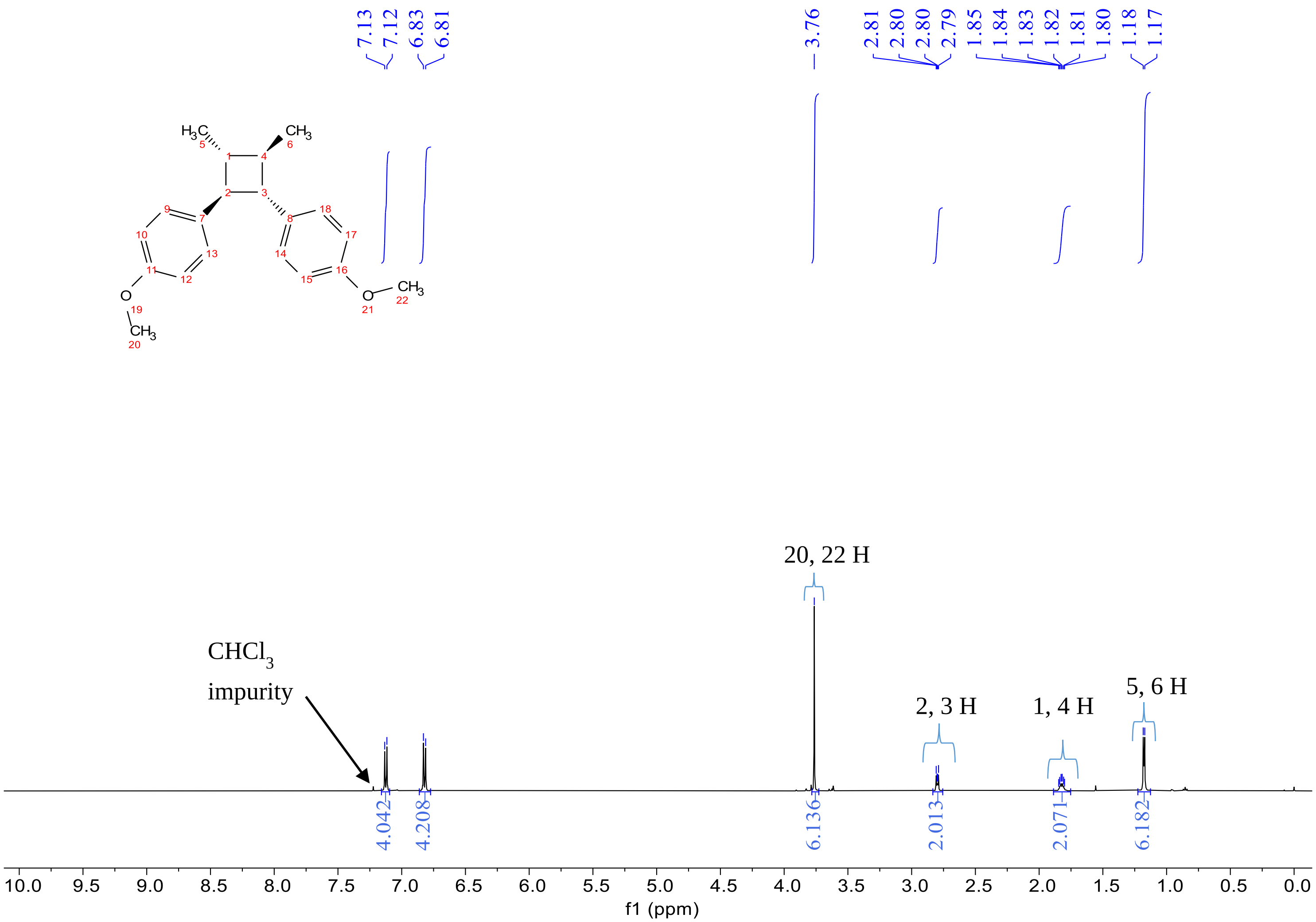

Supplementary Figure 59. ^{1}H NMR of **2a** (500 MHz, Chloroform-*d*)

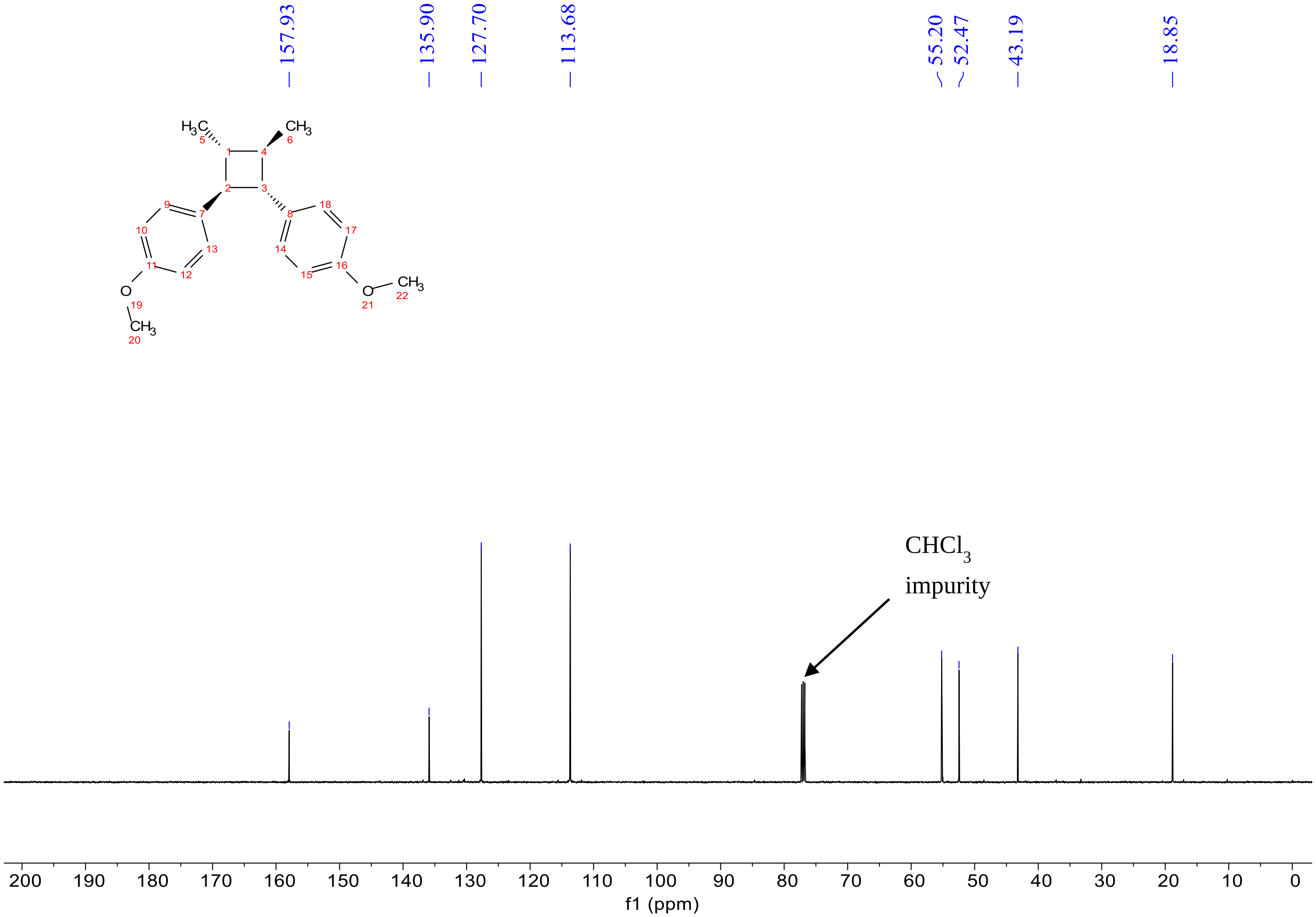

Supplementary Figure 60. ^{13}C NMR of **2a** (126 MHz, Chloroform-*d*)

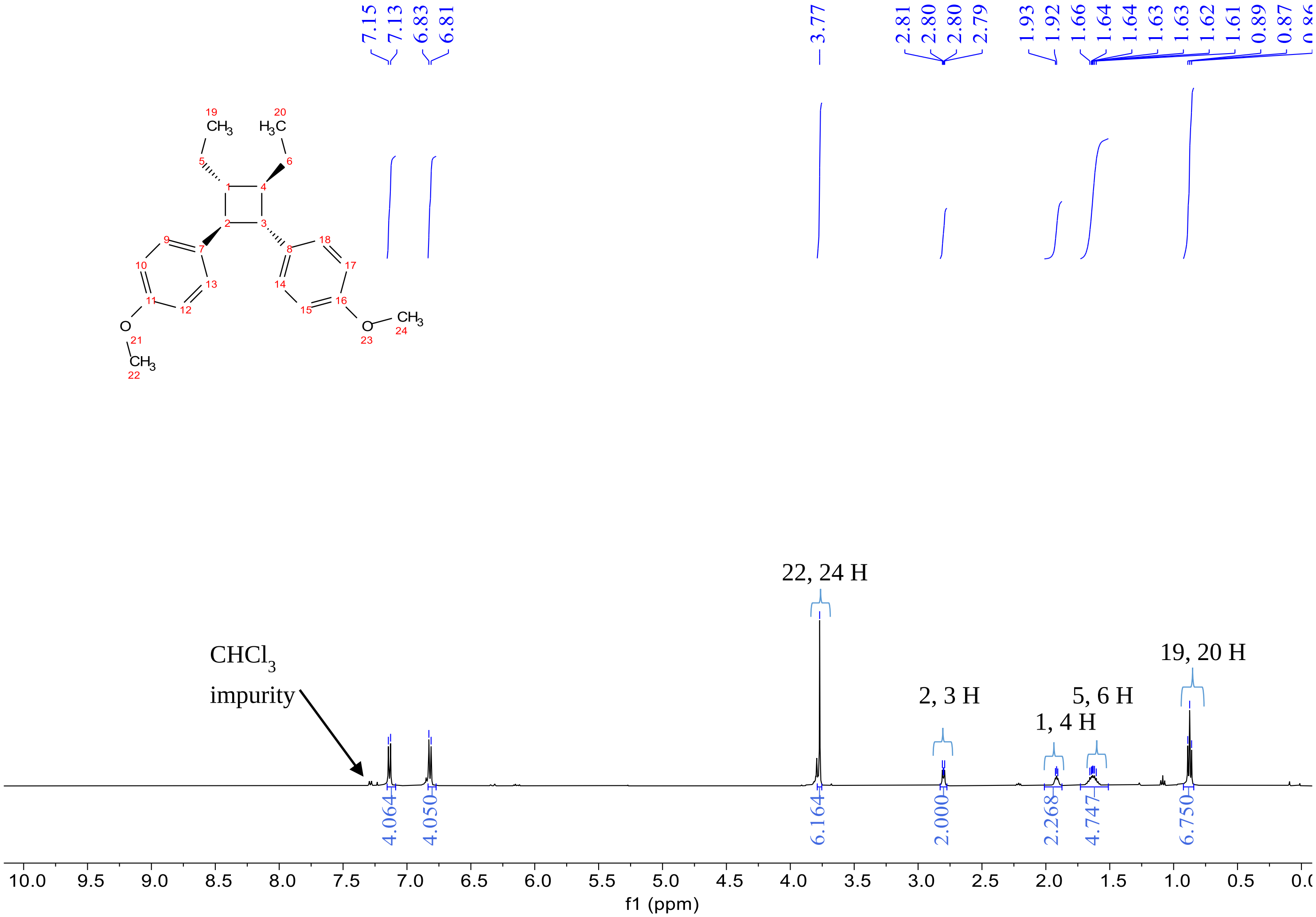

Supplementary Figure 61. ^{1}H NMR of **2b** (500 MHz, Chloroform-*d*)

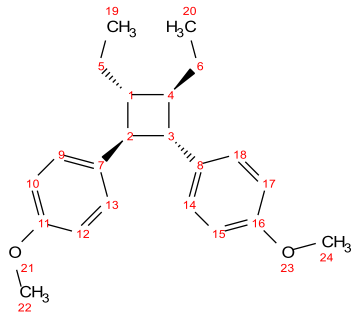

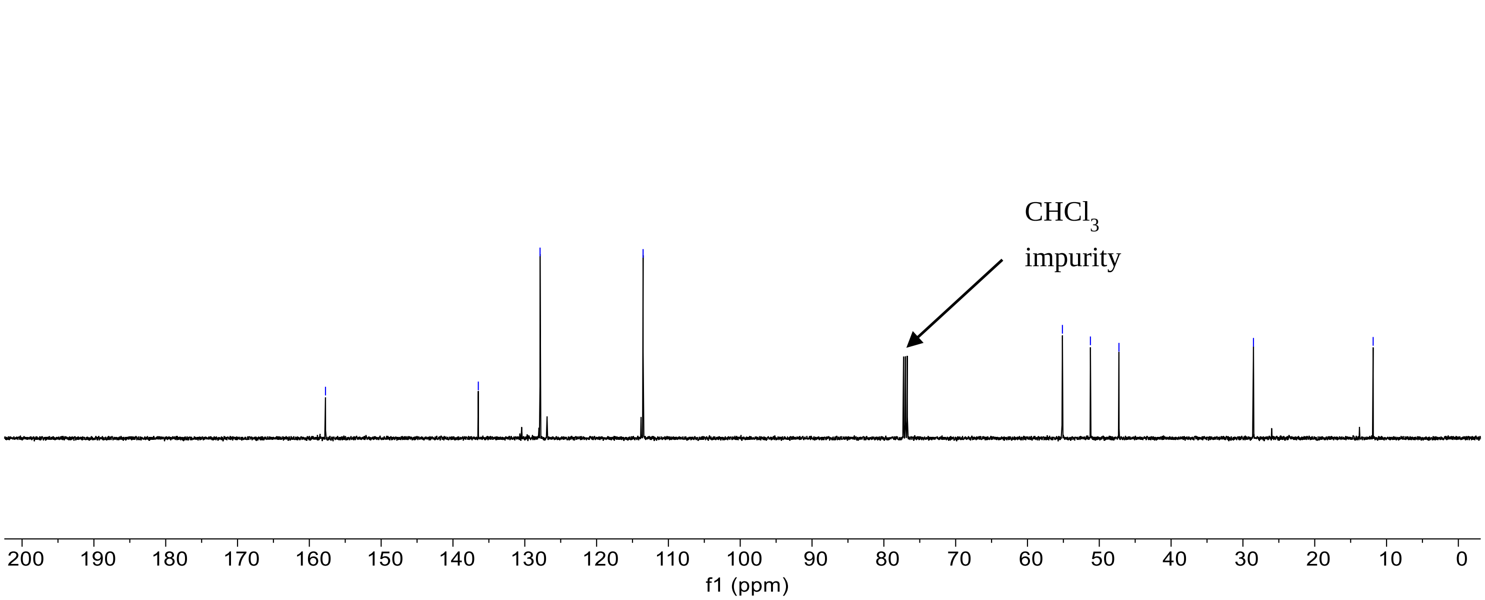

Supplementary Figure 62. ^{13}C NMR of **2b** (126 MHz, Chloroform-*d*)

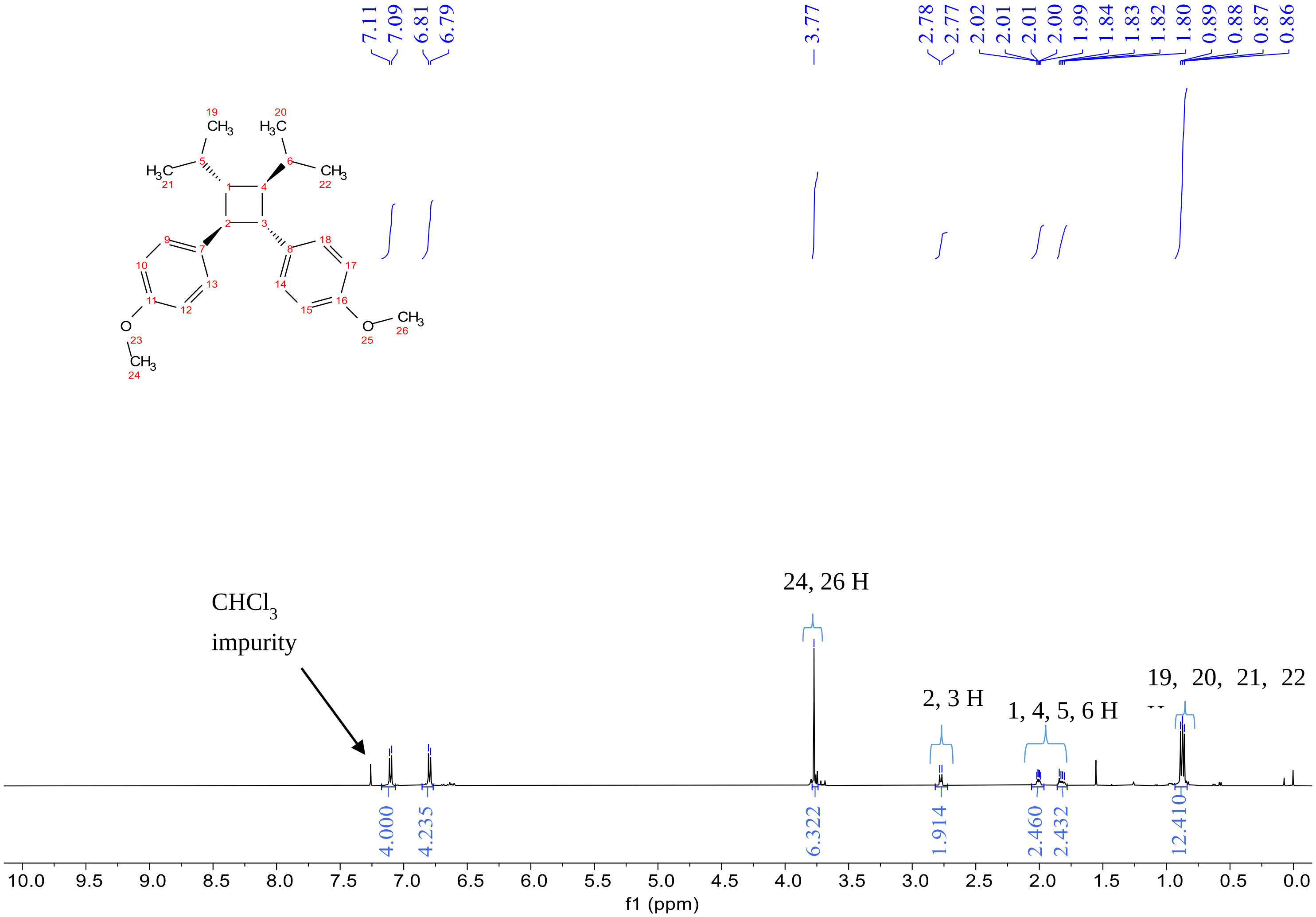

Supplementary Figure 63. ^{1}H NMR of **2c** (500 MHz, Chloroform-*d*)

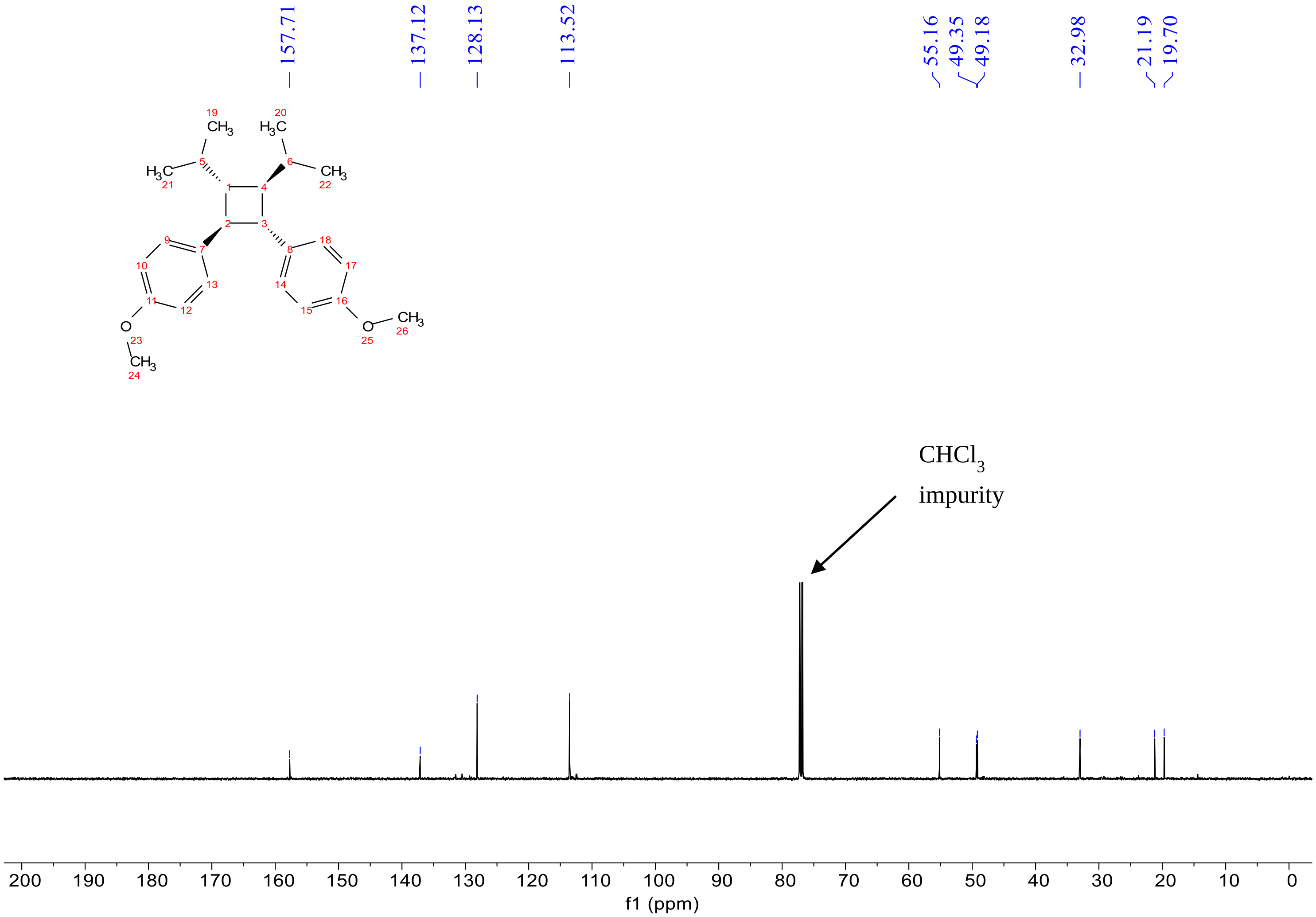

Supplementary Figure 64. ^{13}C NMR of **2c** (126 MHz, Chloroform-*d*)

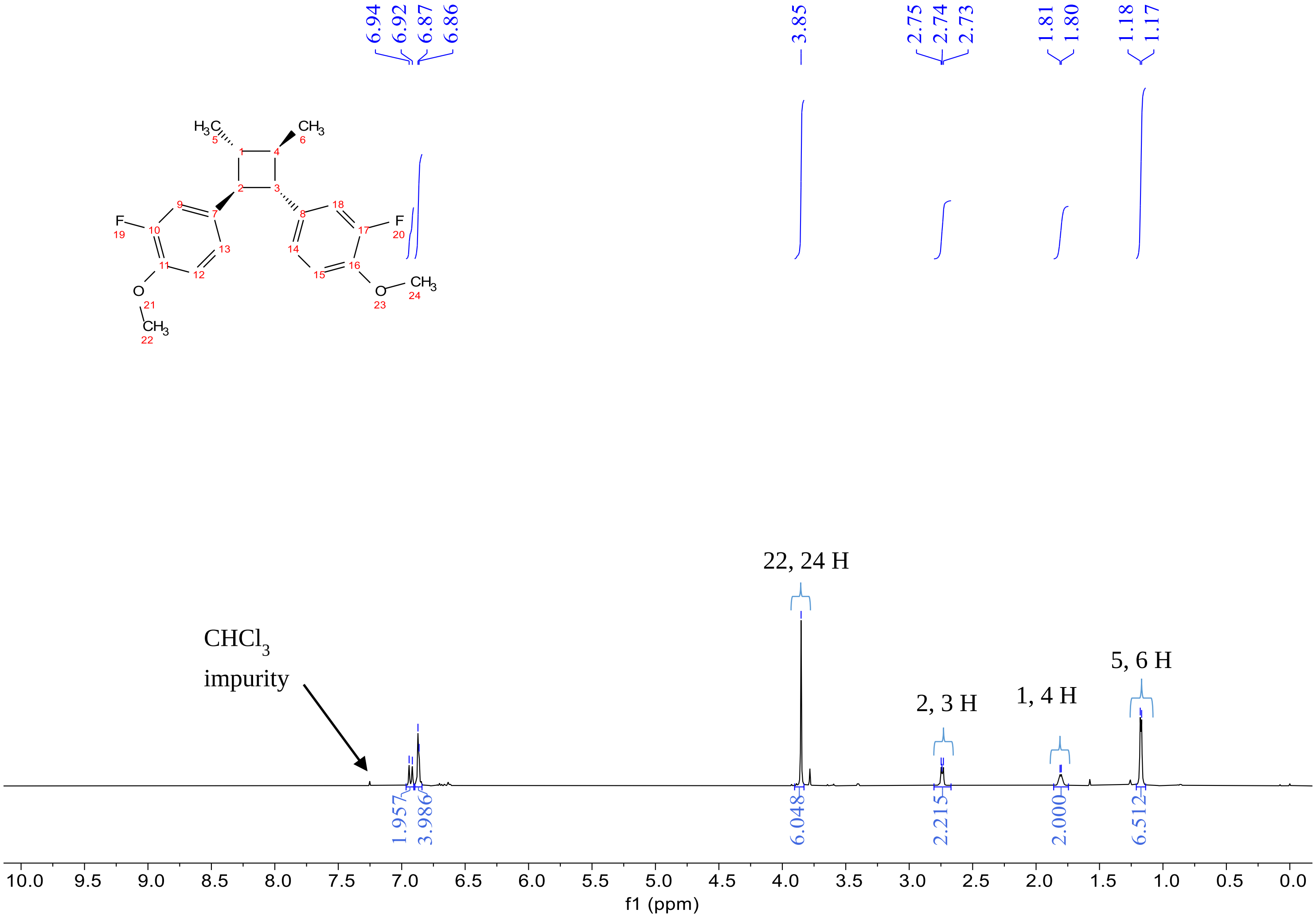

Supplementary Figure 65. ^{1}H NMR of **2d** (500 MHz, Chloroform-*d*)

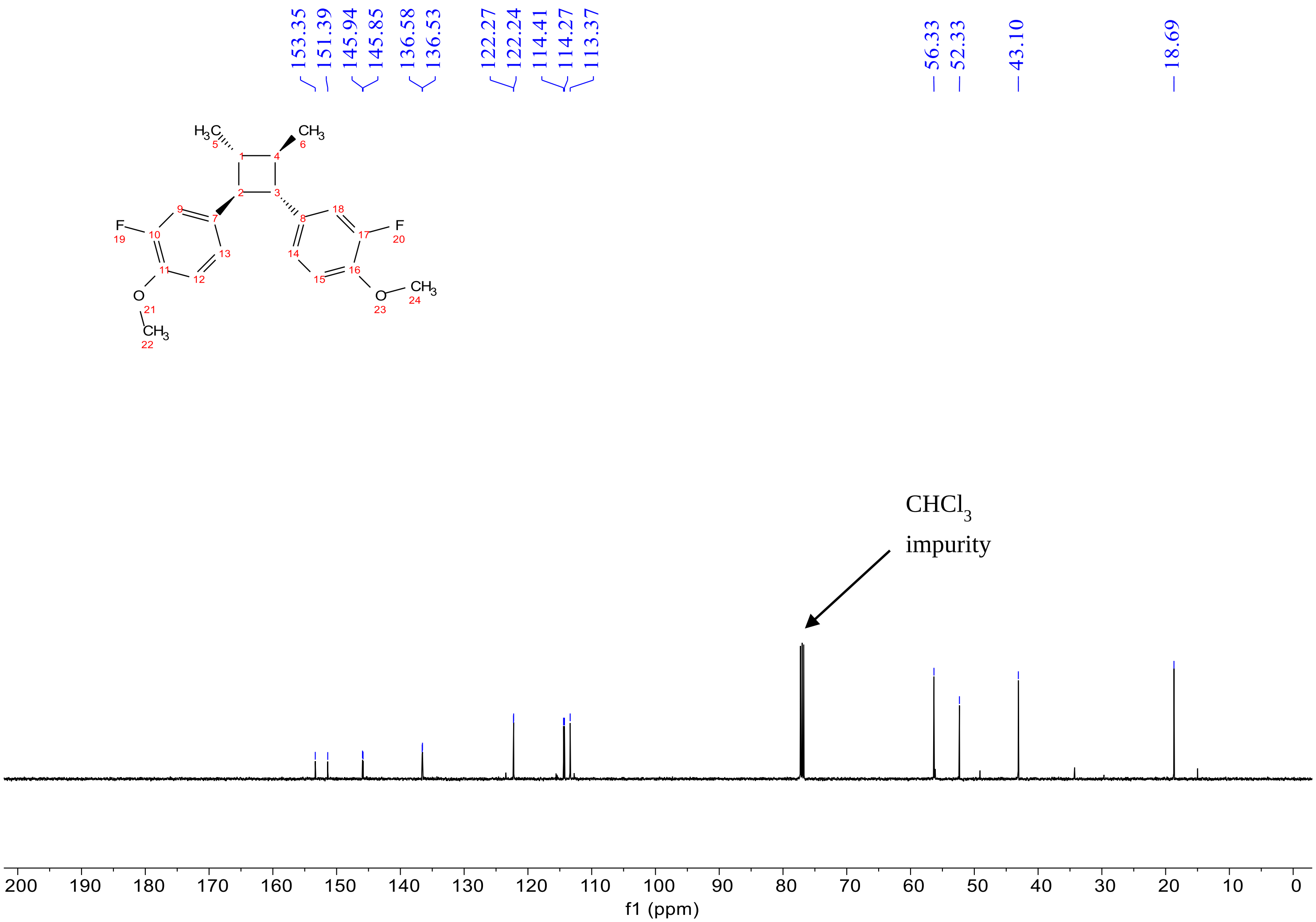

Supplementary Figure 66. ^{13}C NMR of **2d** (126 MHz, Chloroform-*d*)

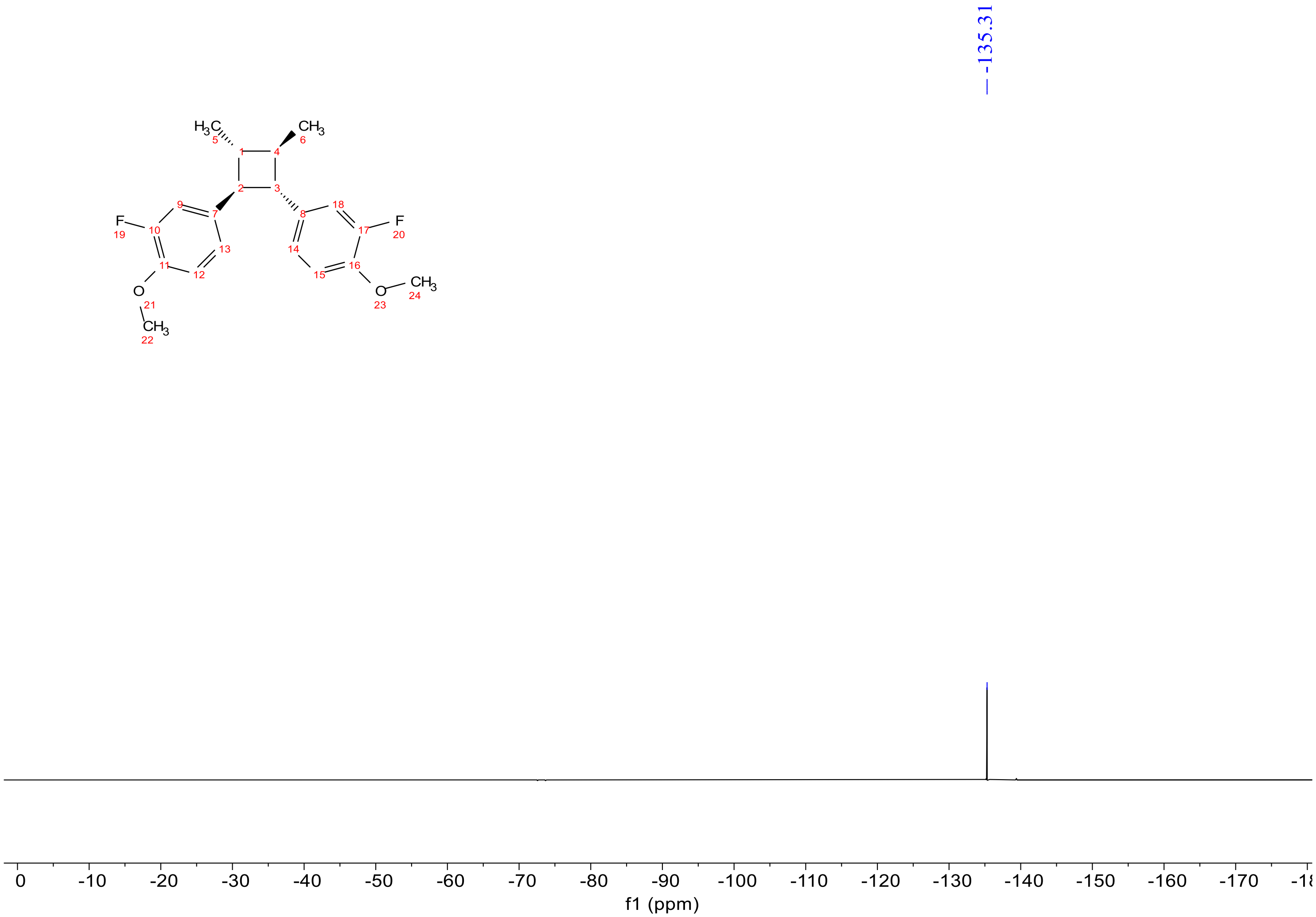

Supplementary Figure 67. ^{19}F NMR of **2d** (471 MHz, Chloroform-*d*)

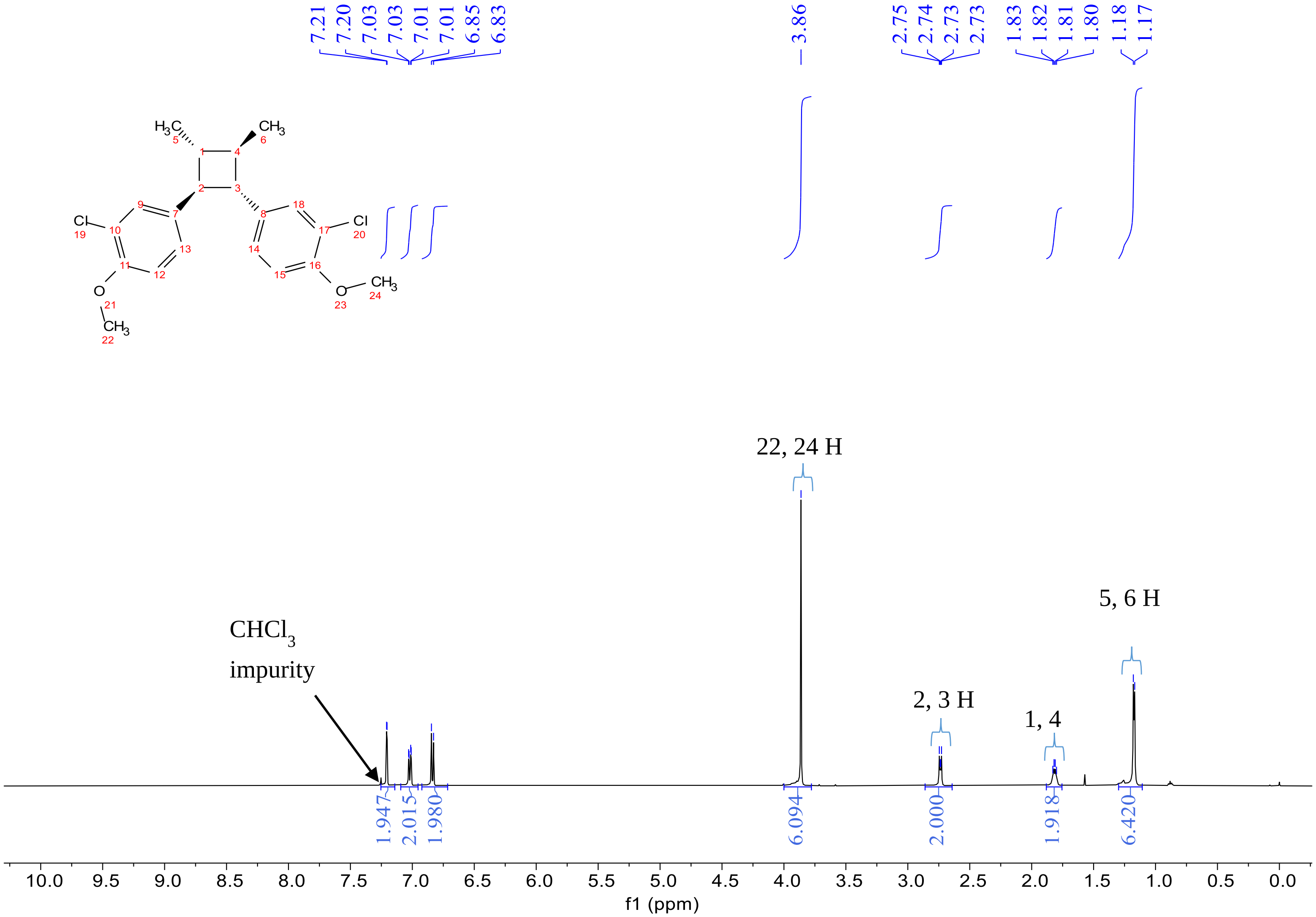

Supplementary Figure 68. ^{1}H NMR of **2e** (500 MHz, Chloroform-*d*)

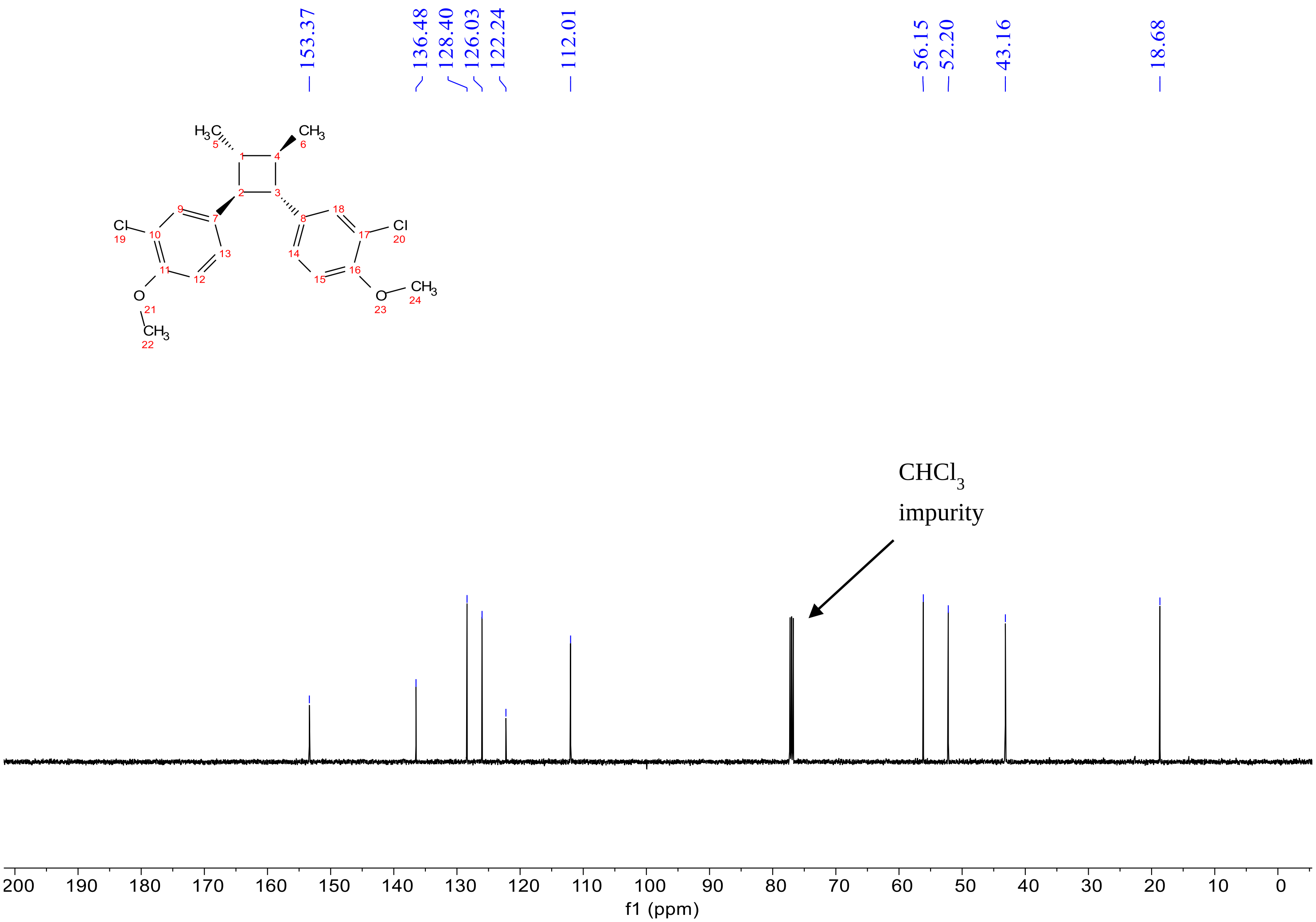

Supplementary Figure 69. ^{13}C NMR of **2e** (126 MHz, Chloroform-*d*)

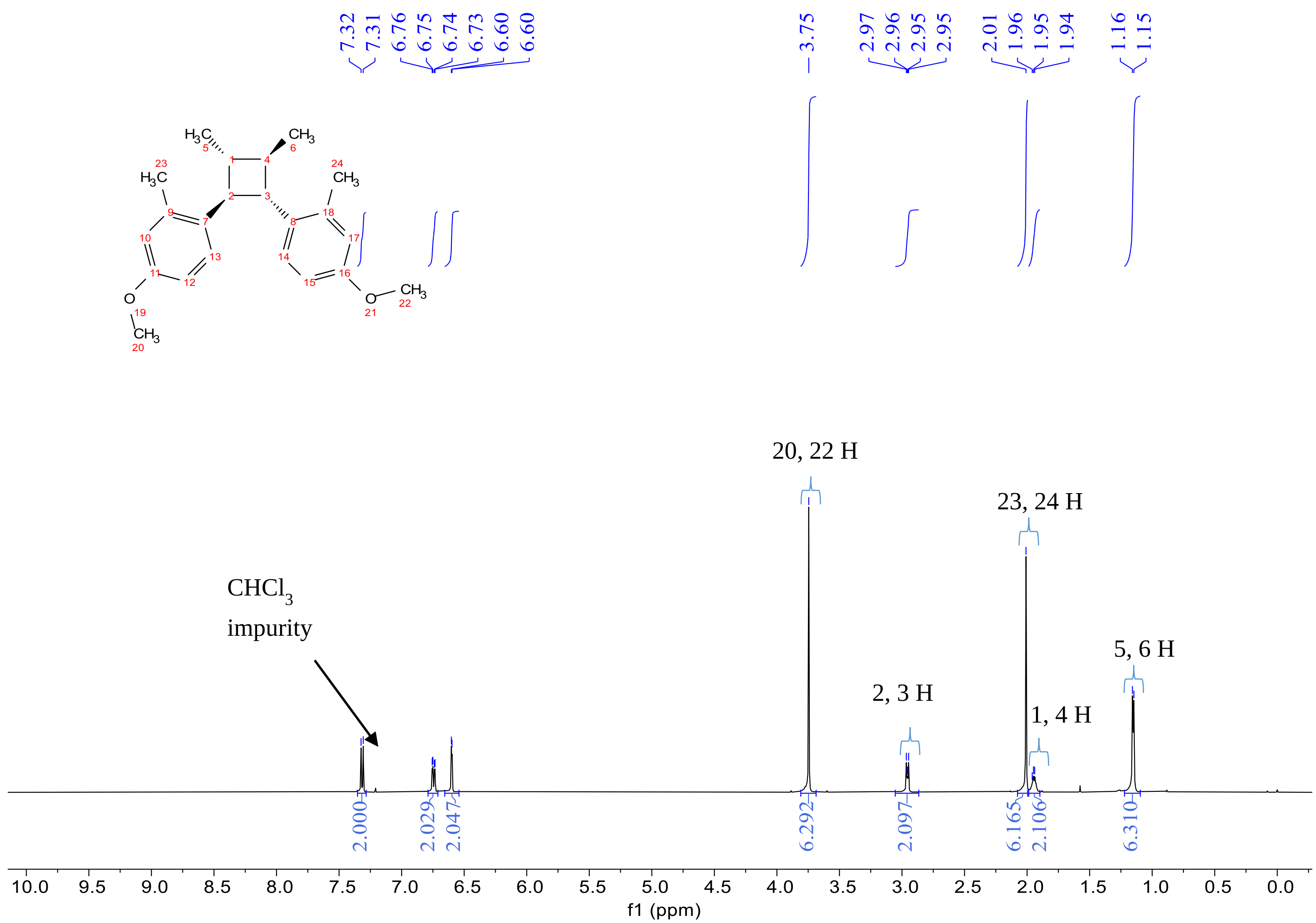

Supplementary Figure 70. ^{1}H NMR of **2f** (500 MHz, Chloroform-*d*)

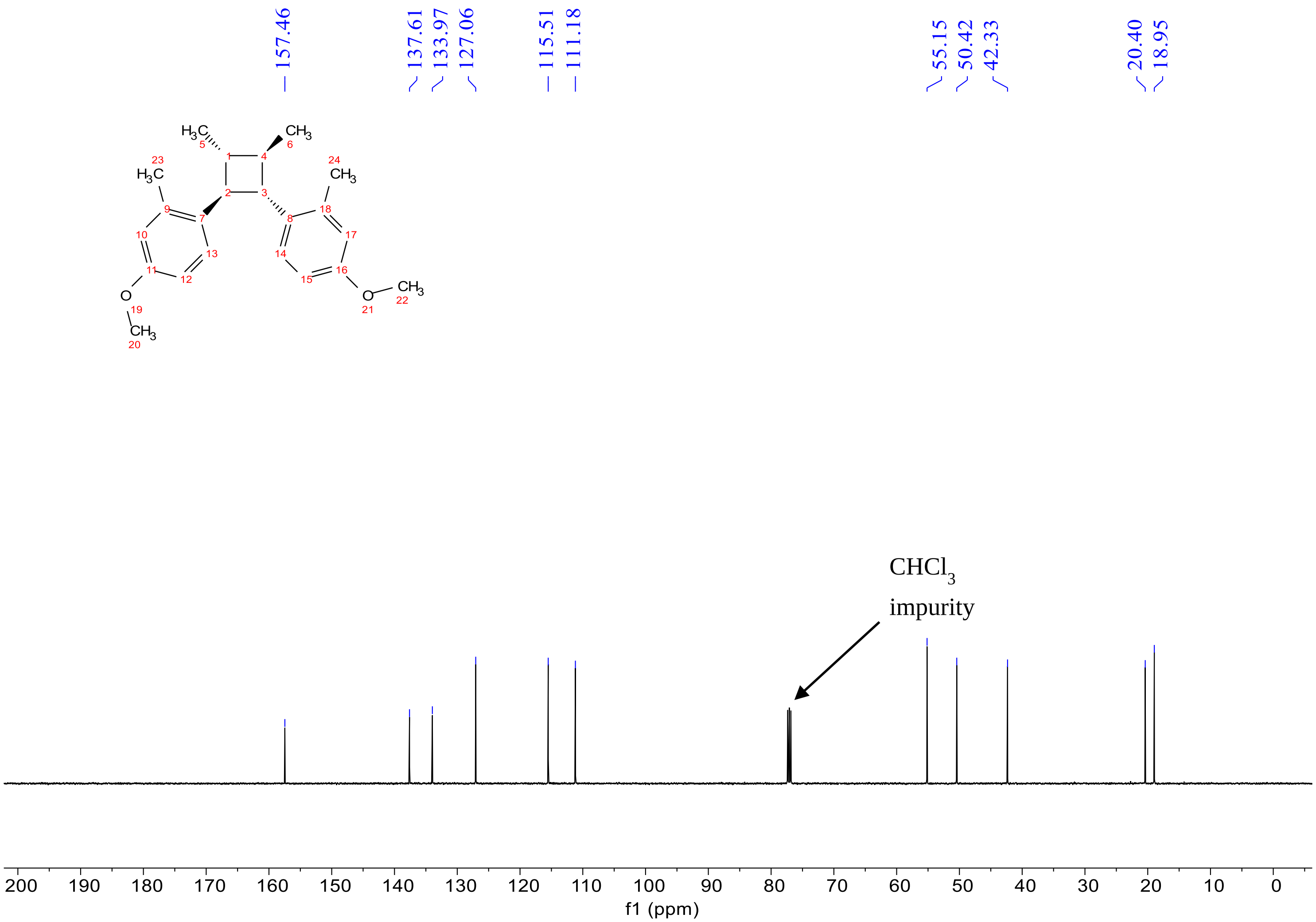

Supplementary Figure 71. ^{13}C NMR of **2f** (126 MHz, Chloroform-*d*)

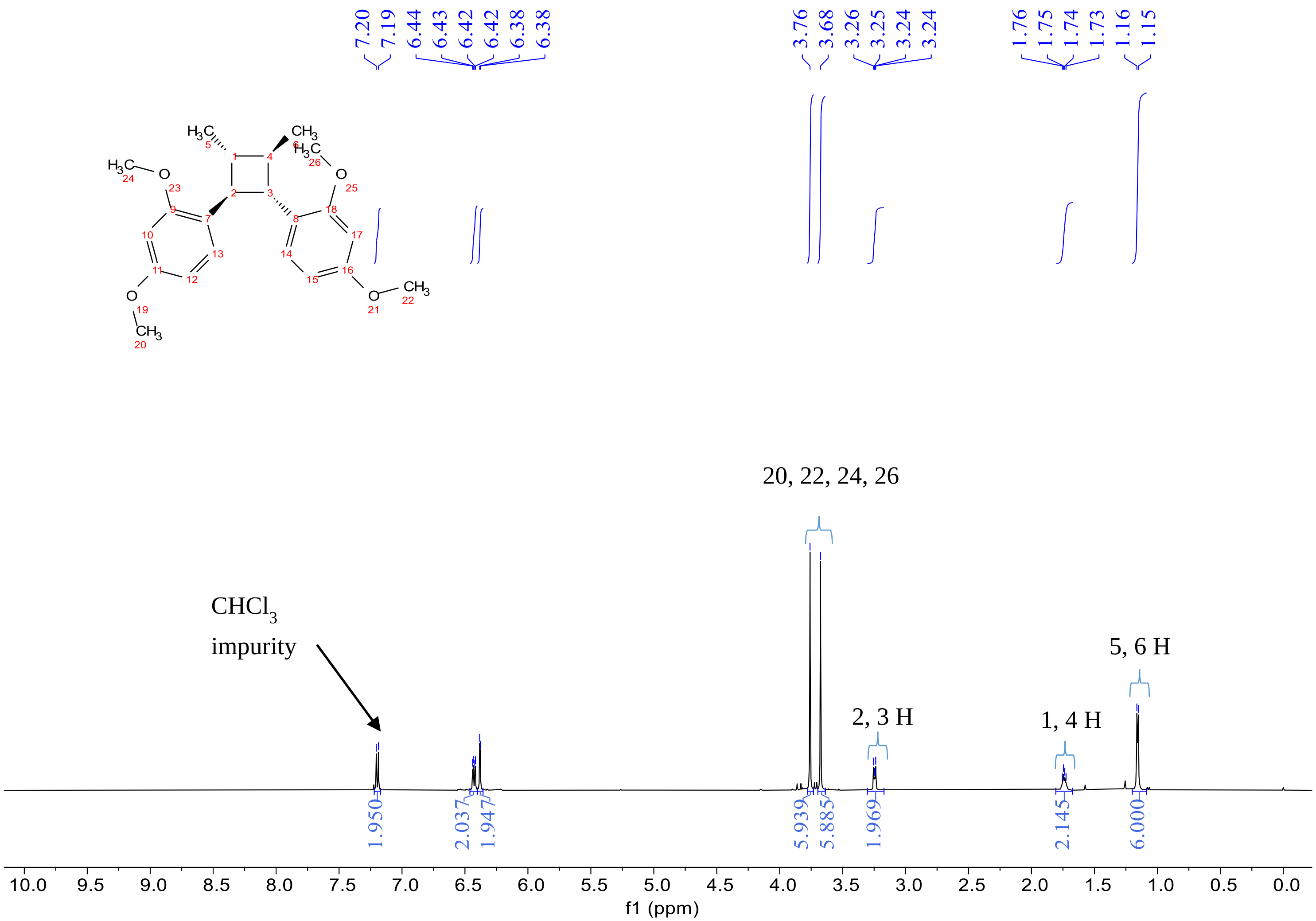

Supplementary Figure 72. ^{1}H NMR of **2g** (500 MHz, Chloroform-*d*)

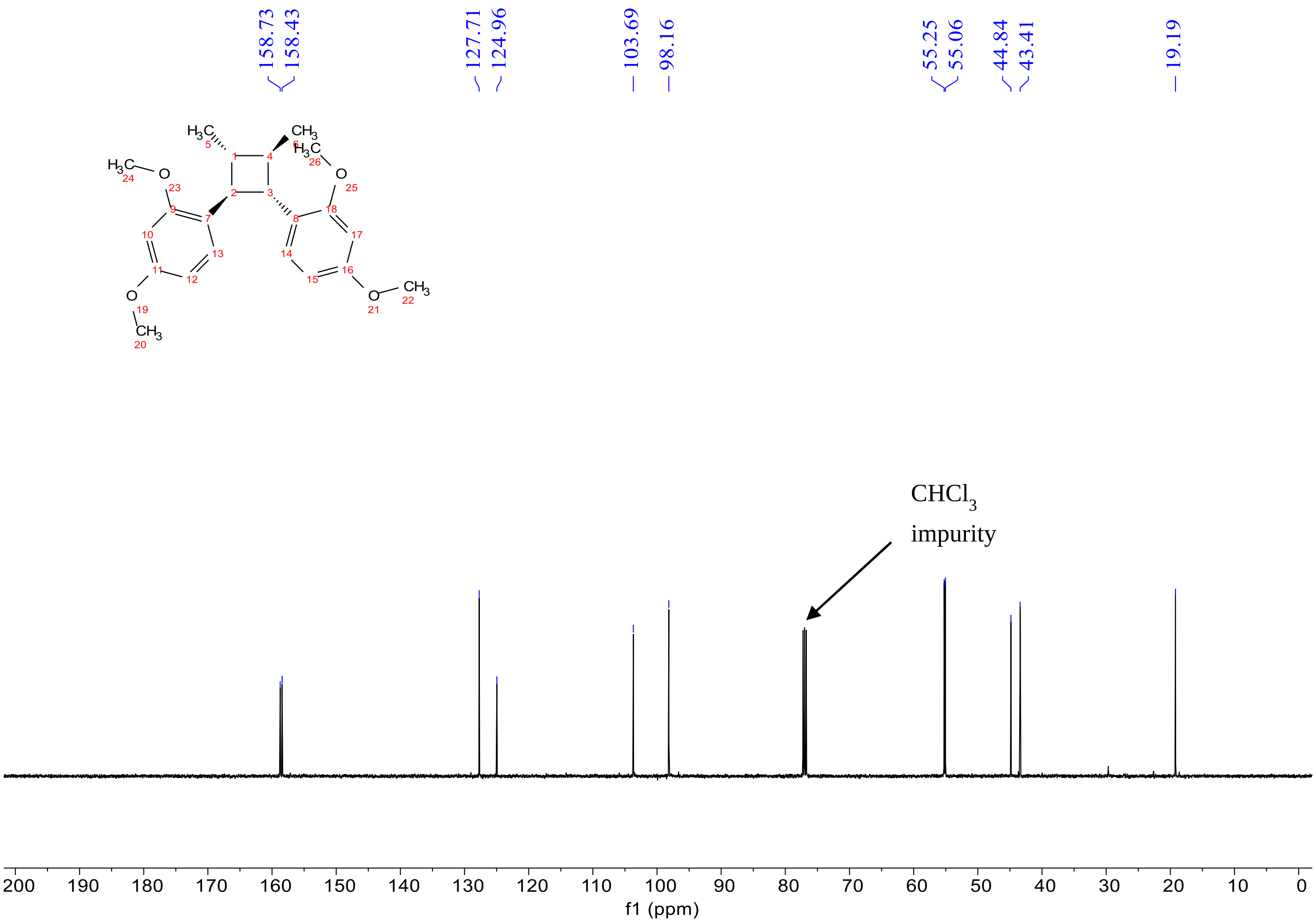

Supplementary Figure 73. ^{13}C NMR of **2g** (126 MHz, Chloroform-*d*)\

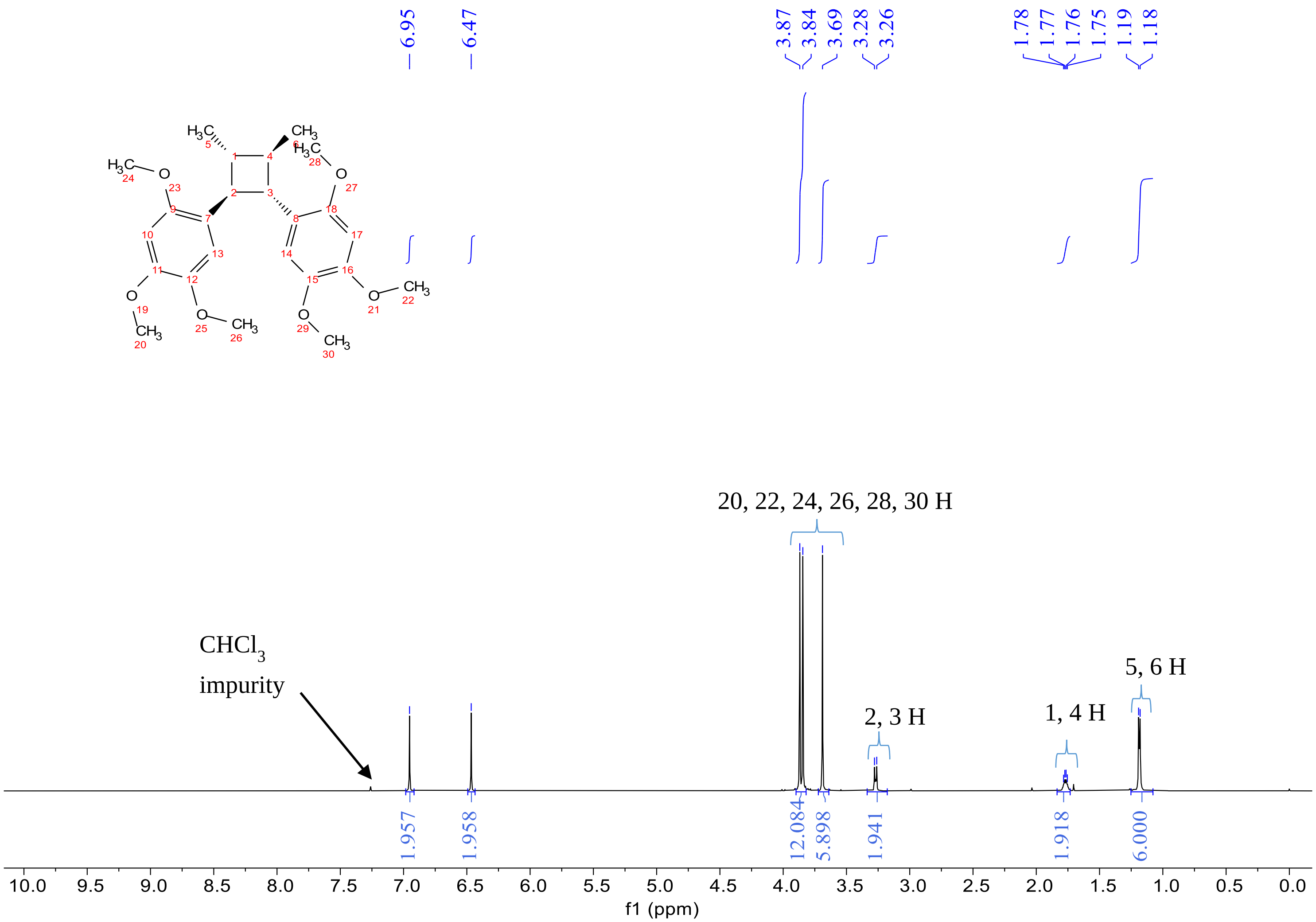

Supplementary Figure 74. ^{1}H NMR of **2h** (500 MHz, Chloroform-*d*)

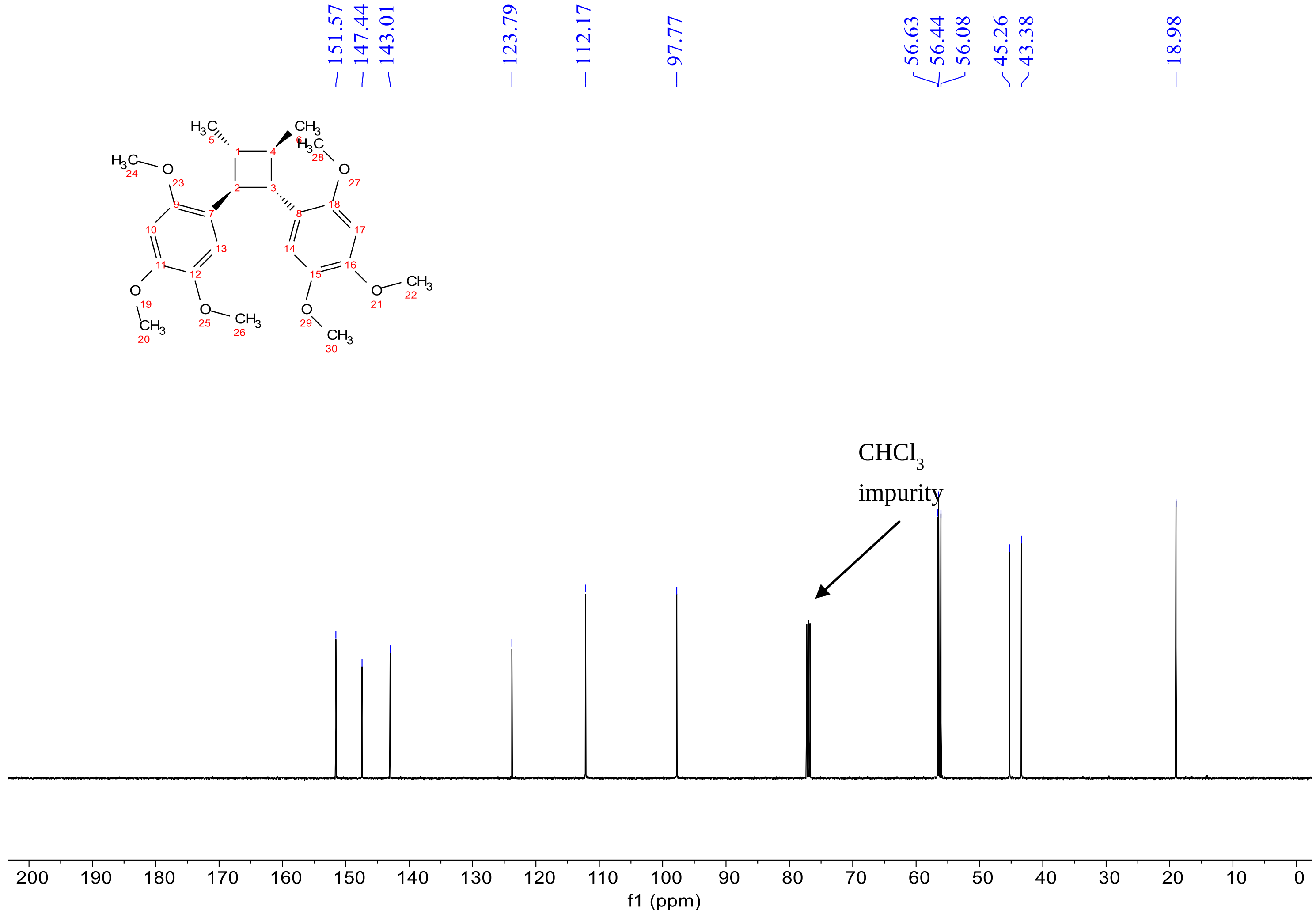

Supplementary Figure 75. ^{13}C NMR of **2h** (126 MHz, Chloroform-*d*)

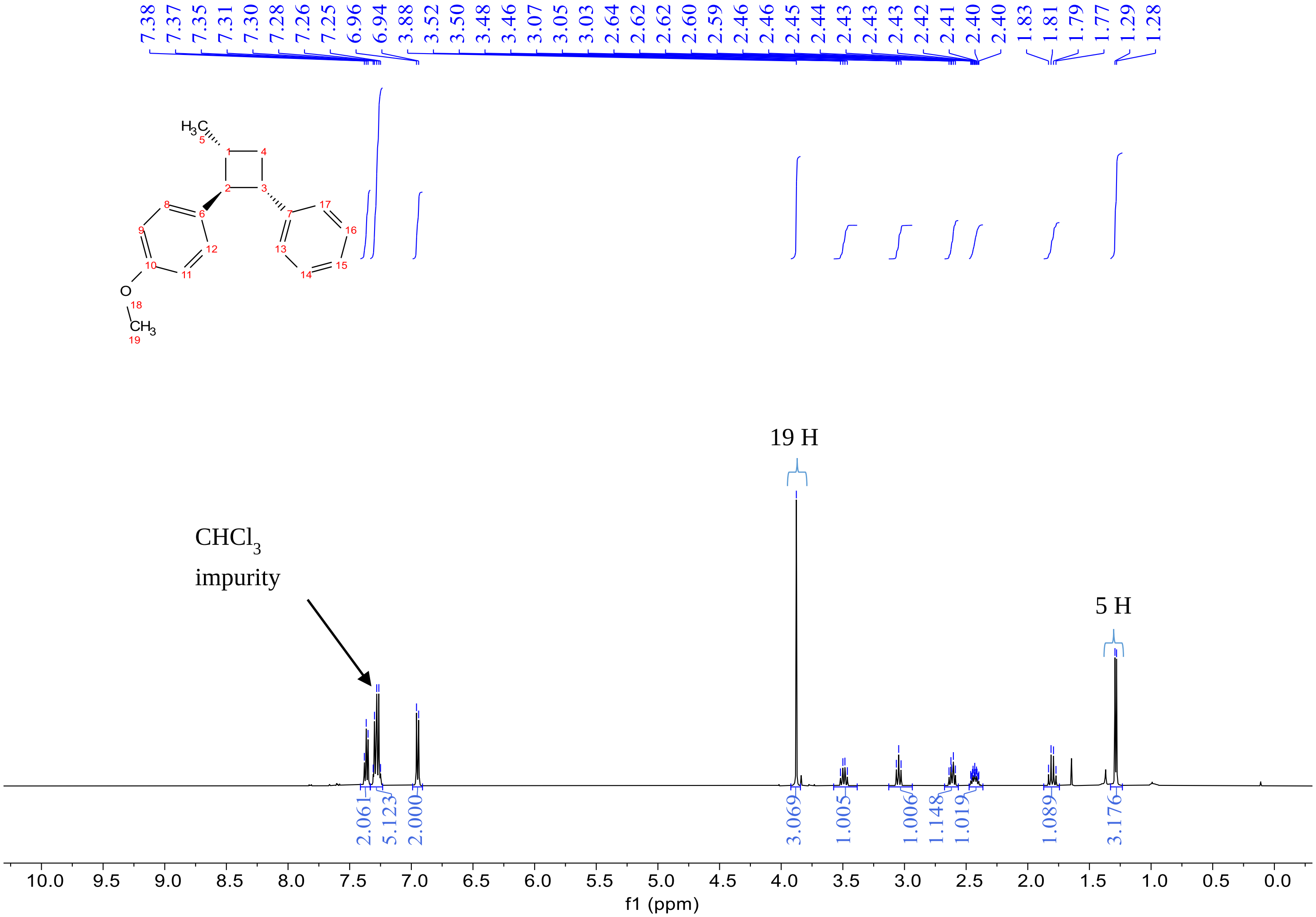

Supplementary Figure 76. ^{1}H NMR of **4a** (500 MHz, Chloroform-*d*)

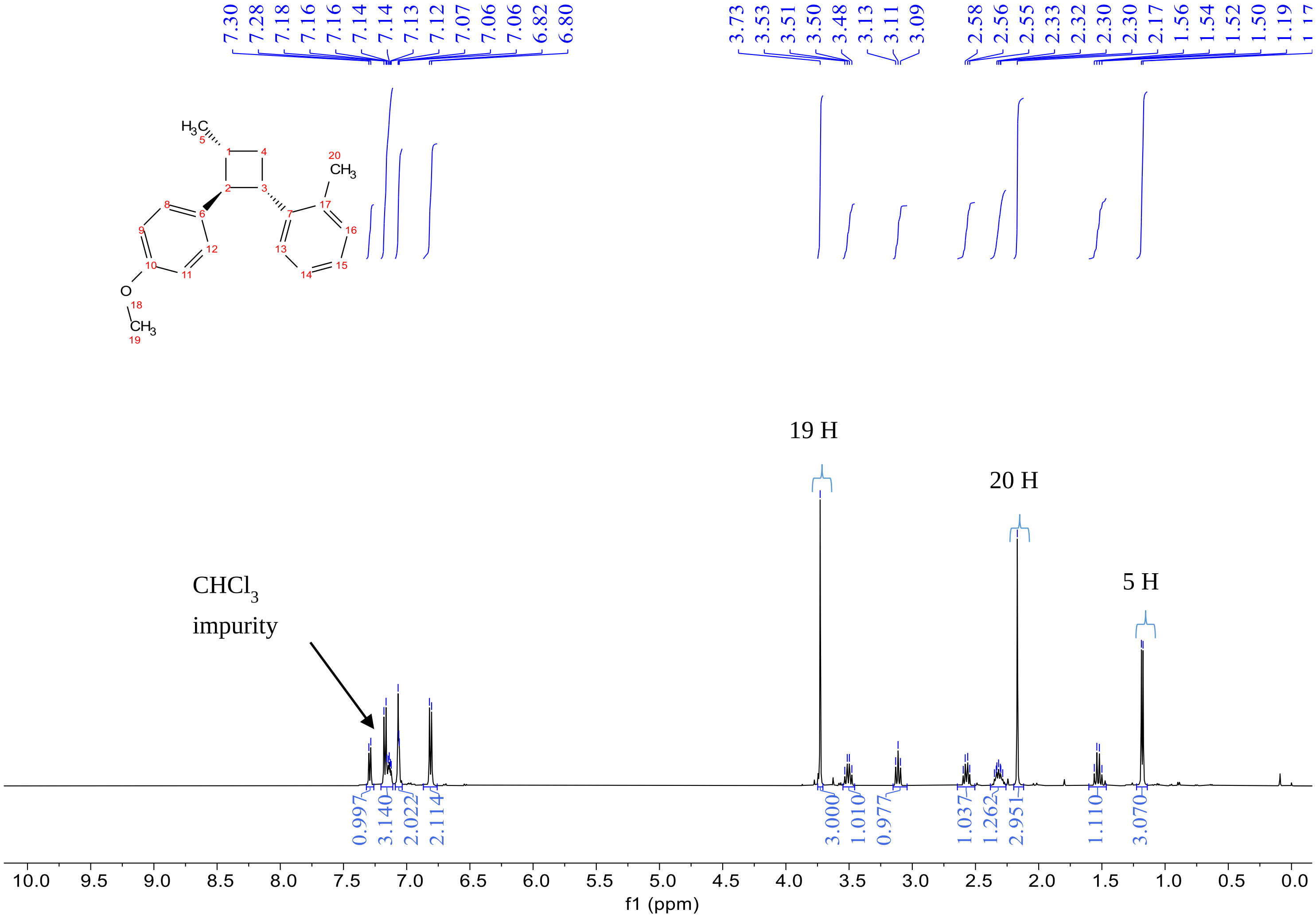

Supplementary Figure 77. ^{1}H NMR of **4b** (500 MHz, Chloroform-*d*)

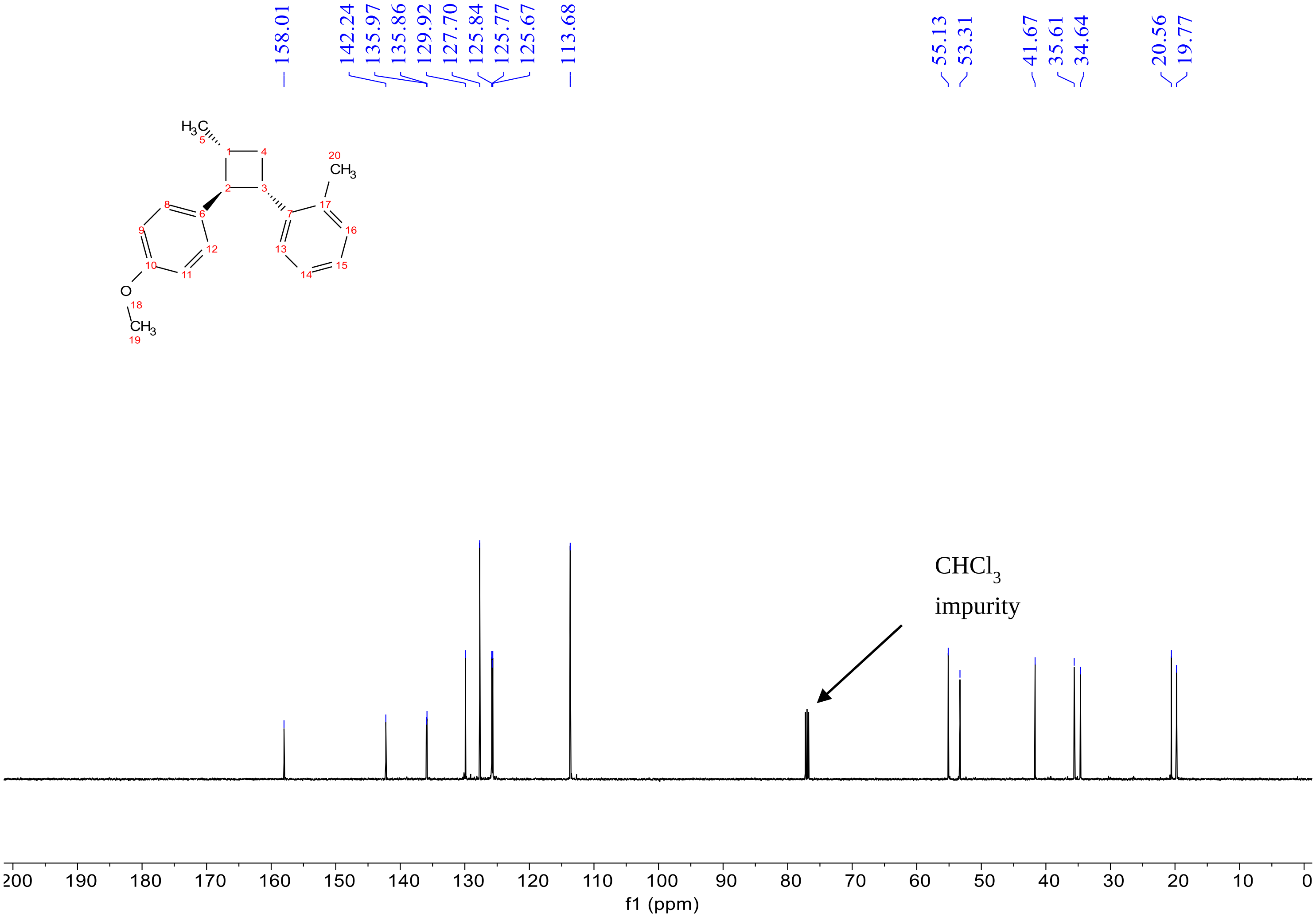

Supplementary Figure 78. ^{13}C NMR of **4b** (126 MHz, Chloroform-*d*)

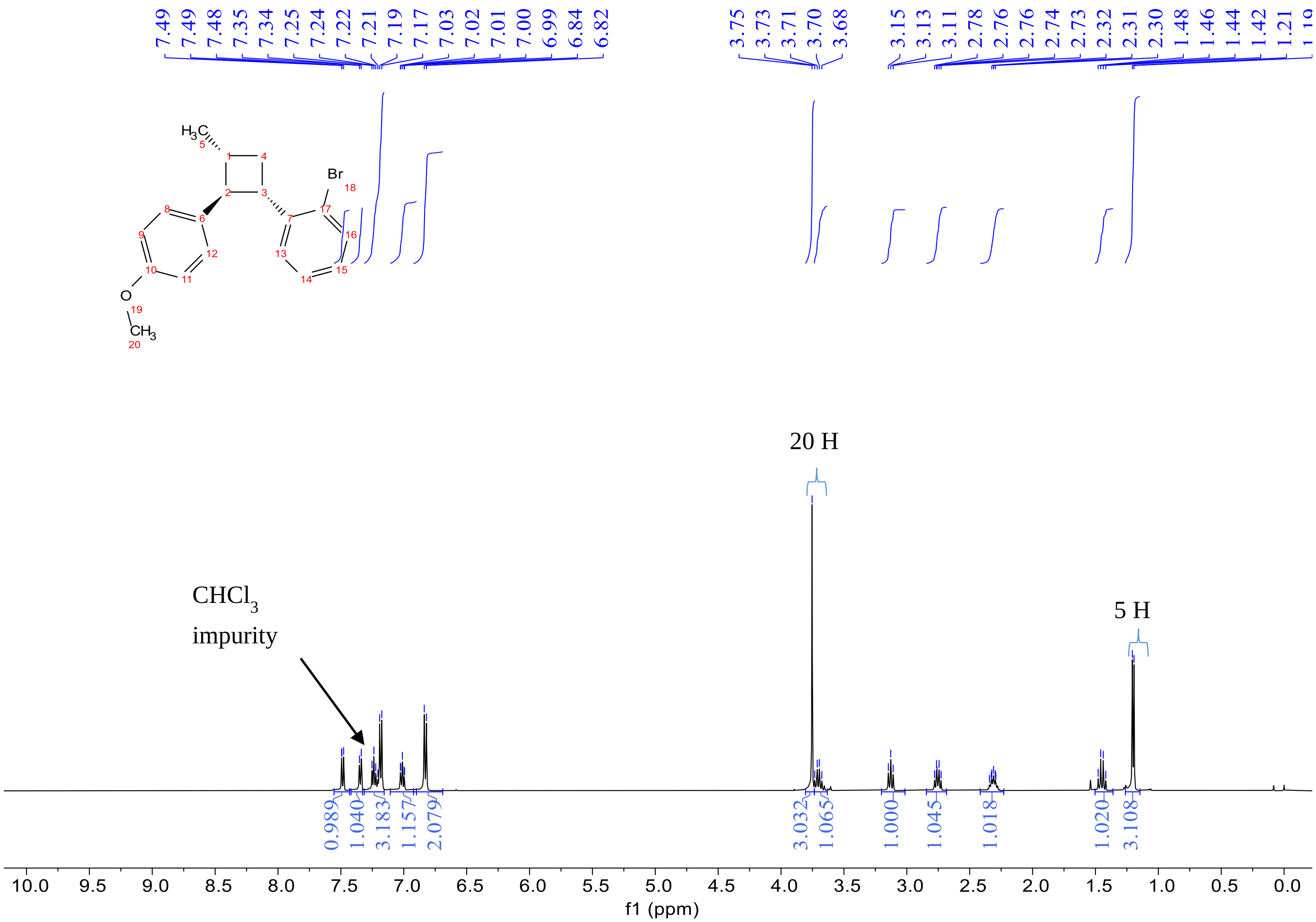

Supplementary Figure 79. ^{1}H NMR of **4c** (500 MHz, Chloroform-*d*)

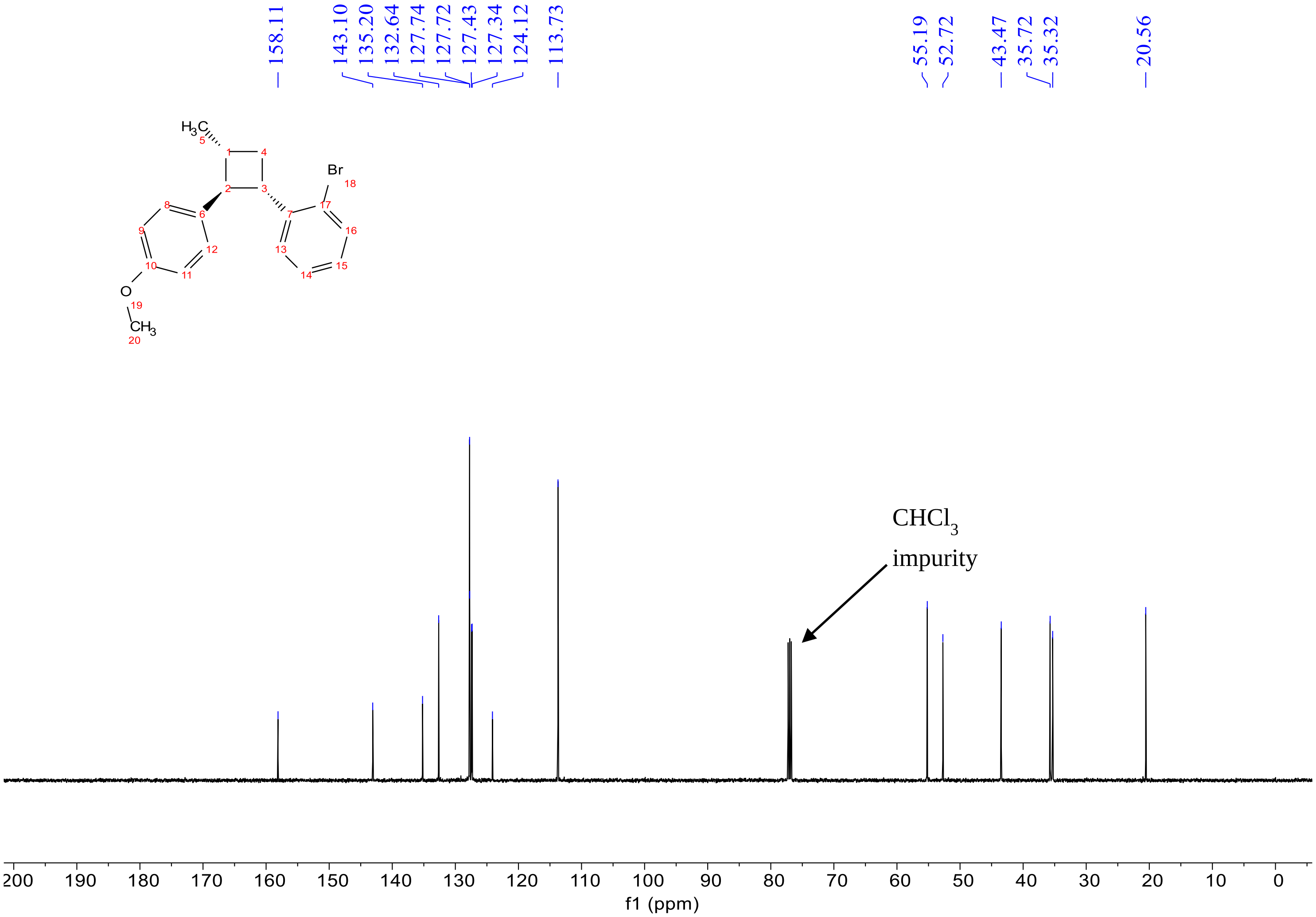

Supplementary Figure 80. ^{13}C NMR of **4c** (126 MHz, Chloroform-*d*)

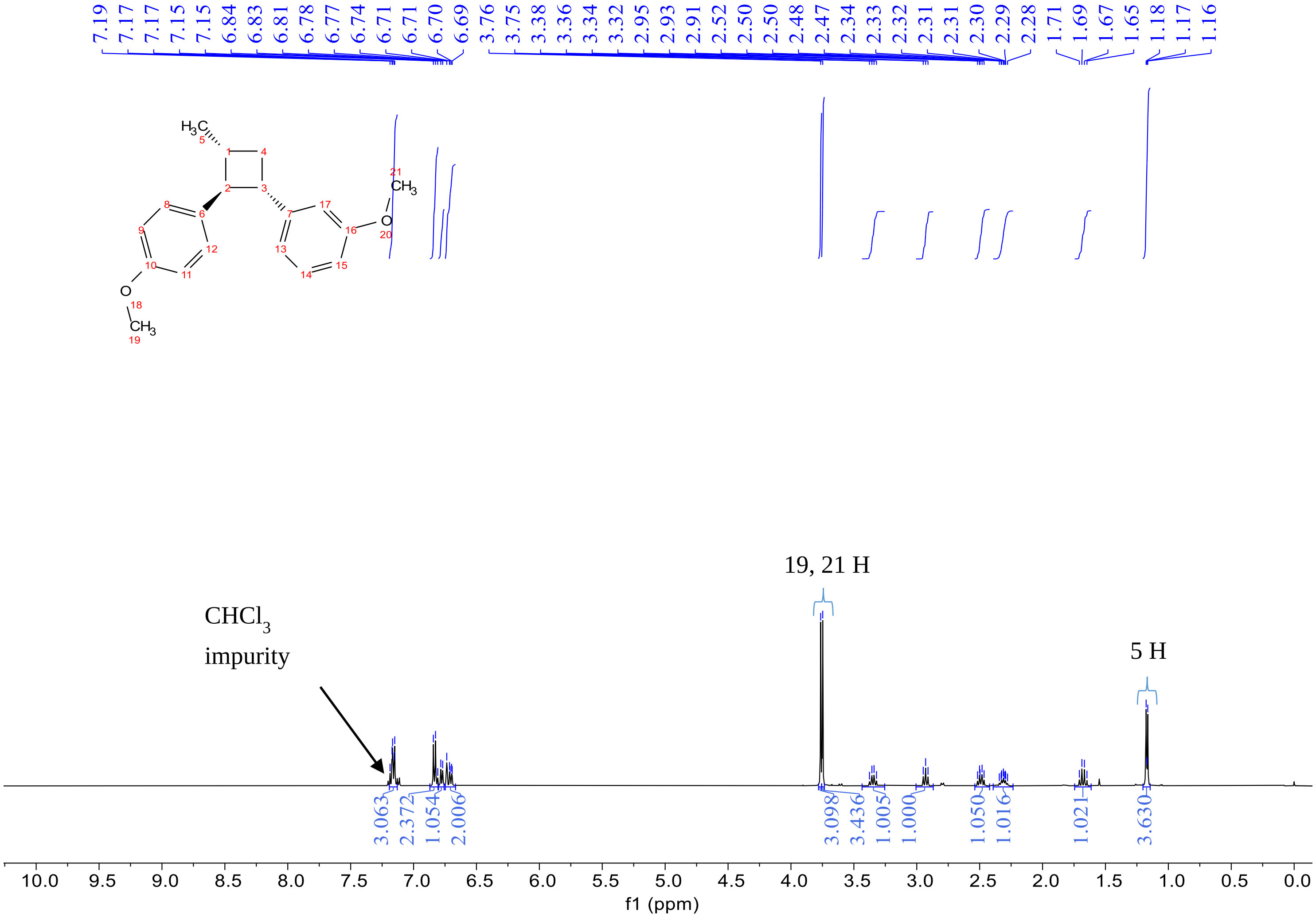

Supplementary Figure 81. ^{1}H NMR of **4d** (500 MHz, Chloroform-*d*)

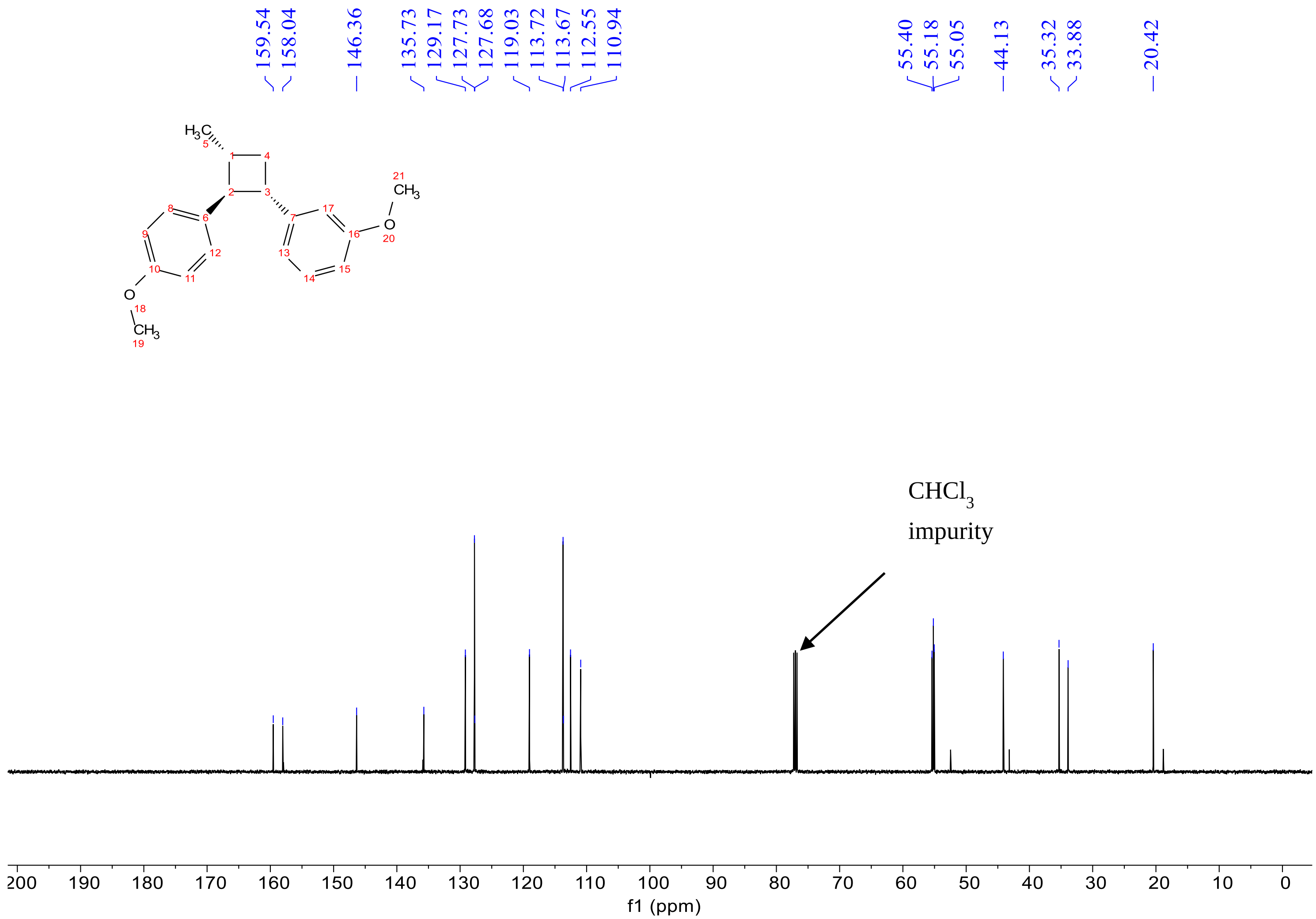

Supplementary Figure 82. ^{13}C NMR of **4d** (126 MHz, Chloroform-*d*)

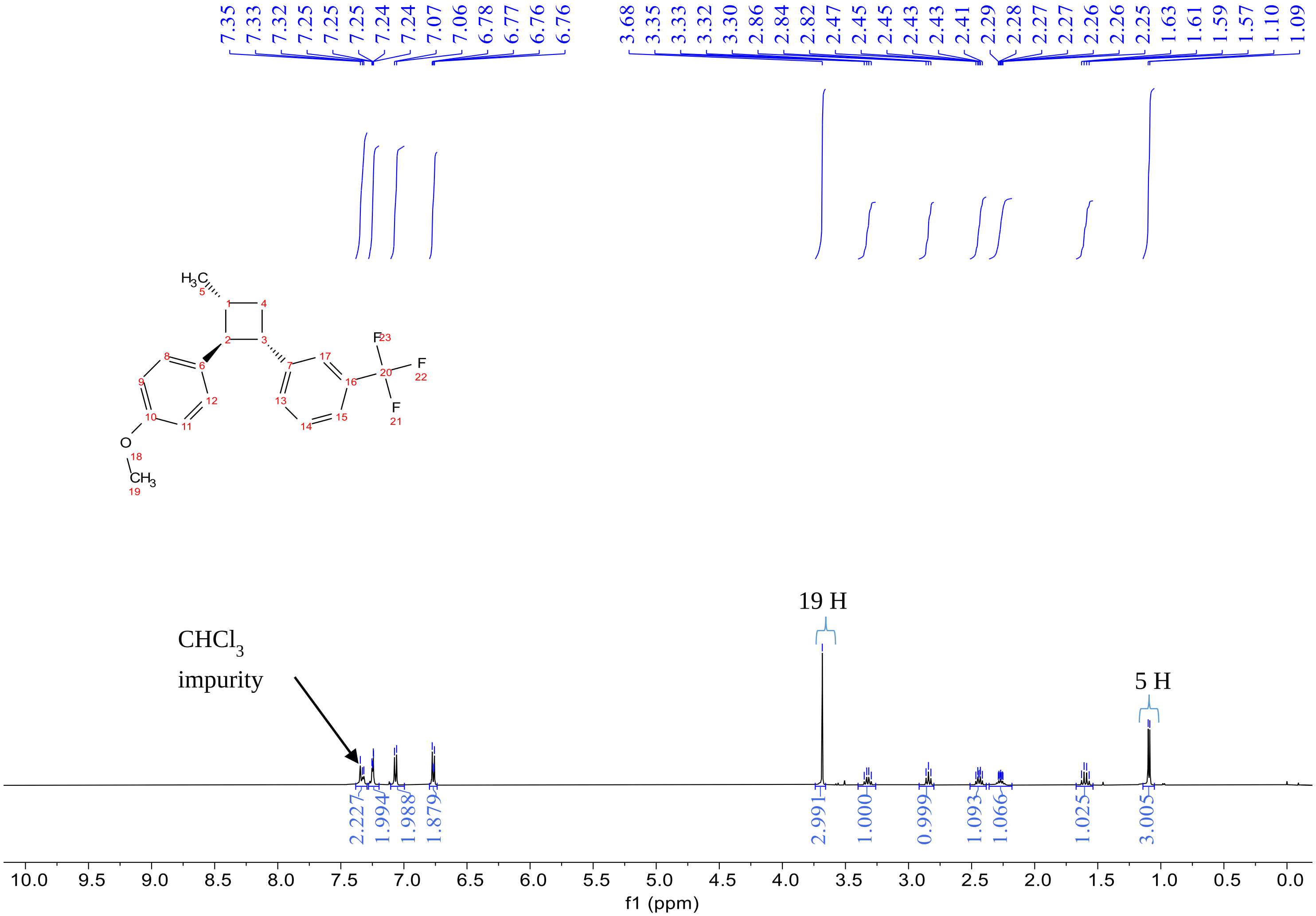

Supplementary Figure 83. ^{1}H NMR of **4e** (500 MHz, Chloroform-*d*)

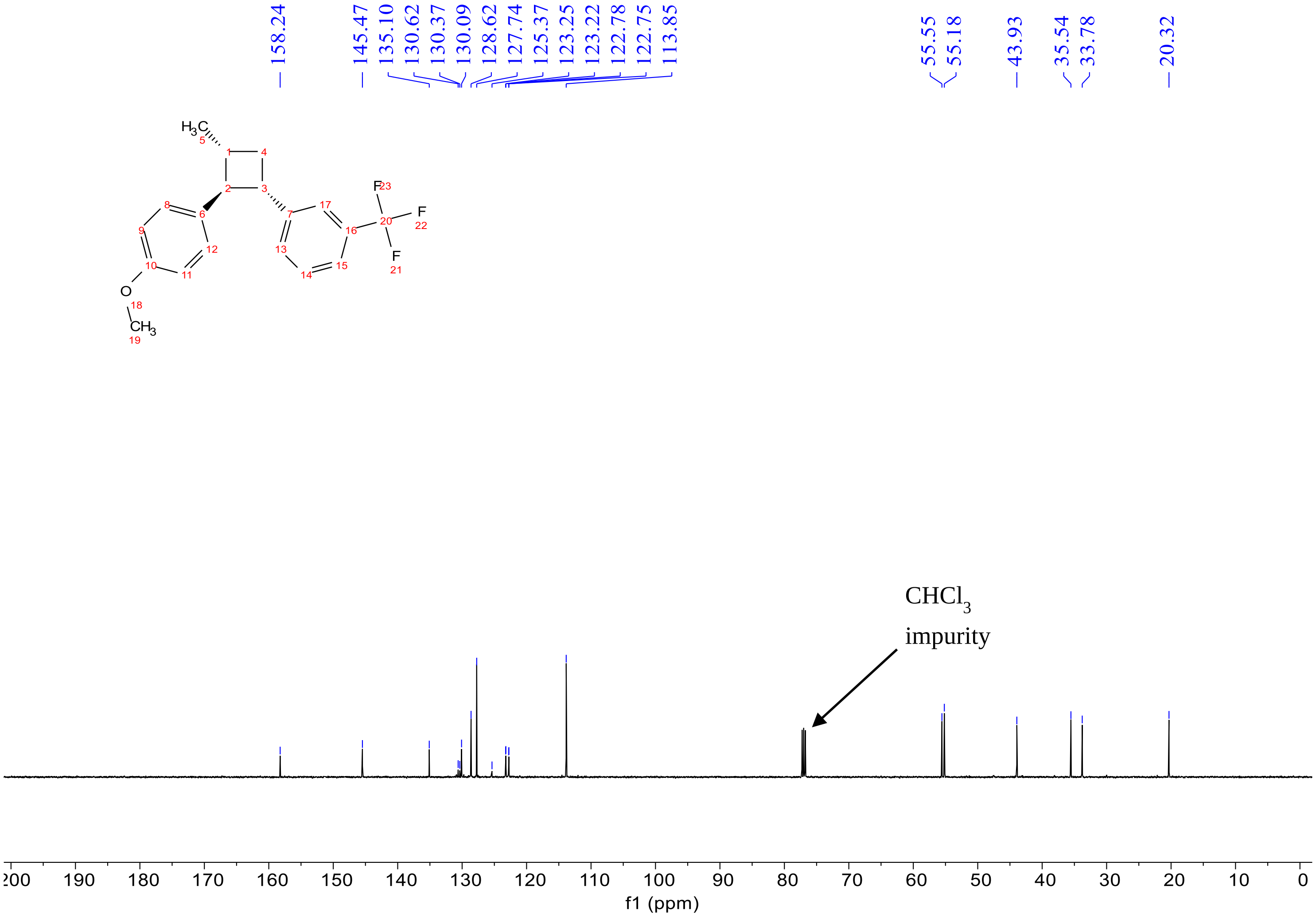

Supplementary Figure 84. ^{13}C NMR of **4e** (126 MHz, Chloroform-*d*)

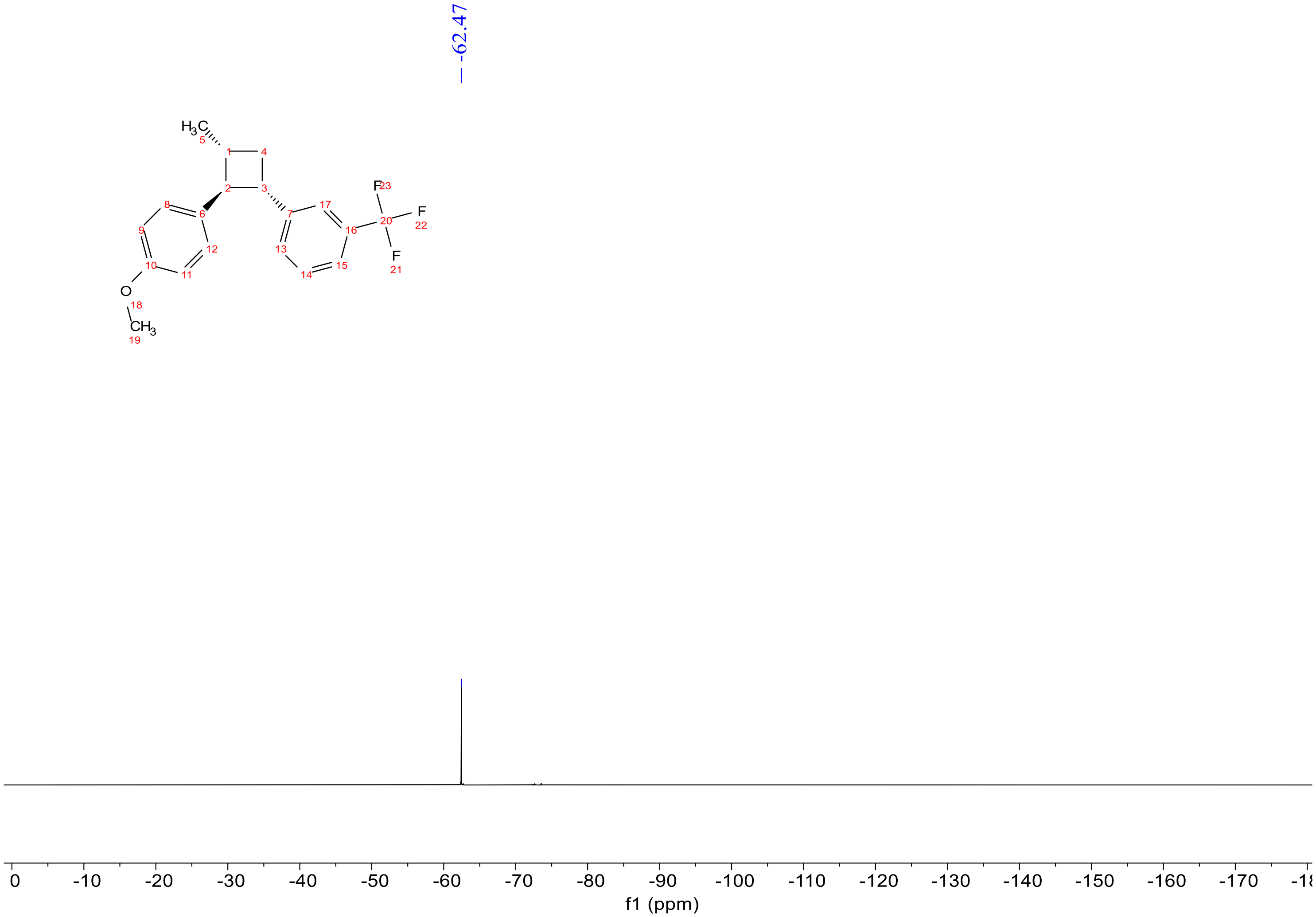

Supplementary Figure 85. ^{19}F NMR of **4e** (471 MHz, Chloroform-*d*)

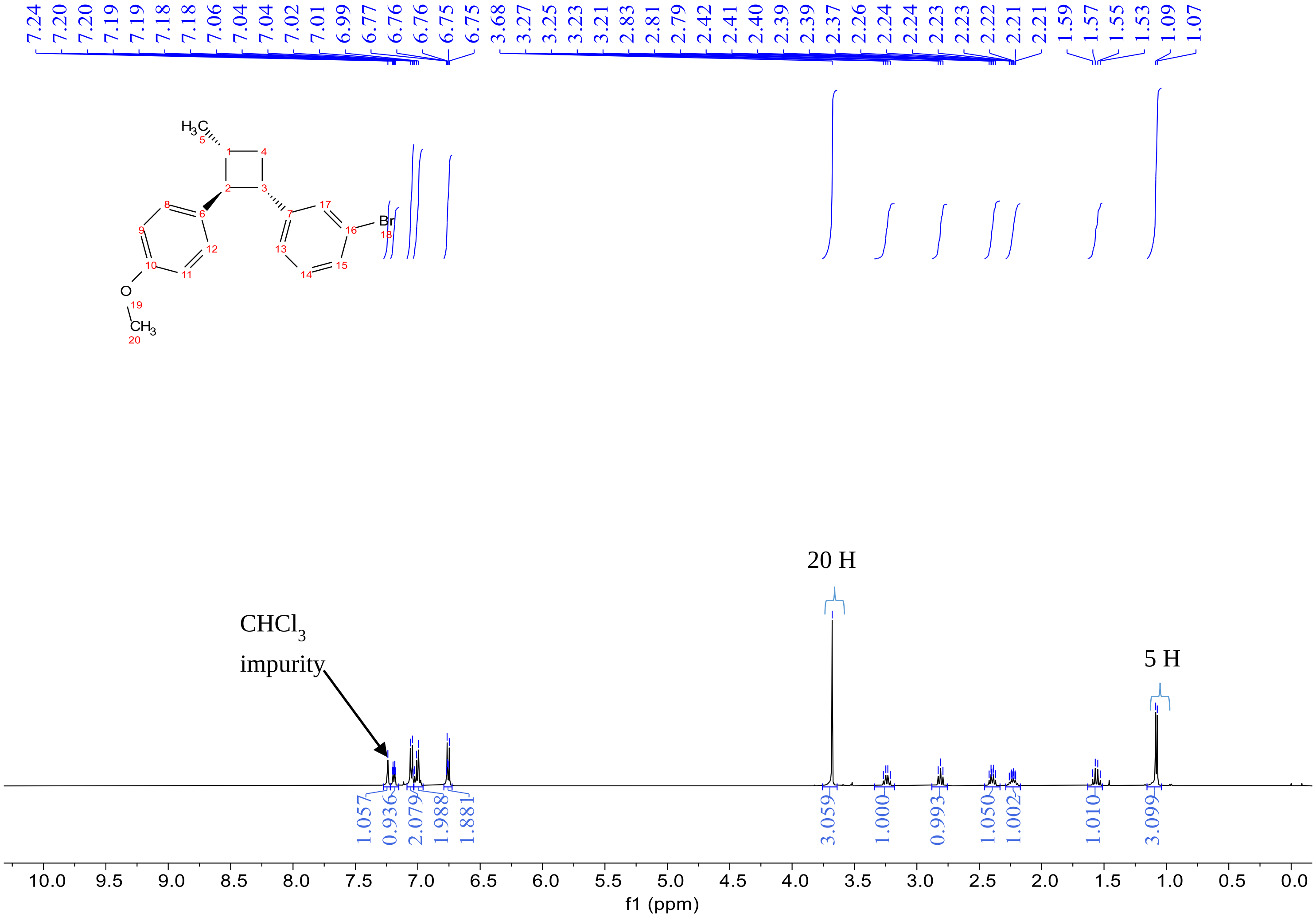

Supplementary Figure 86. ^{1}H NMR of **4f** (500 MHz, Chloroform-*d*)

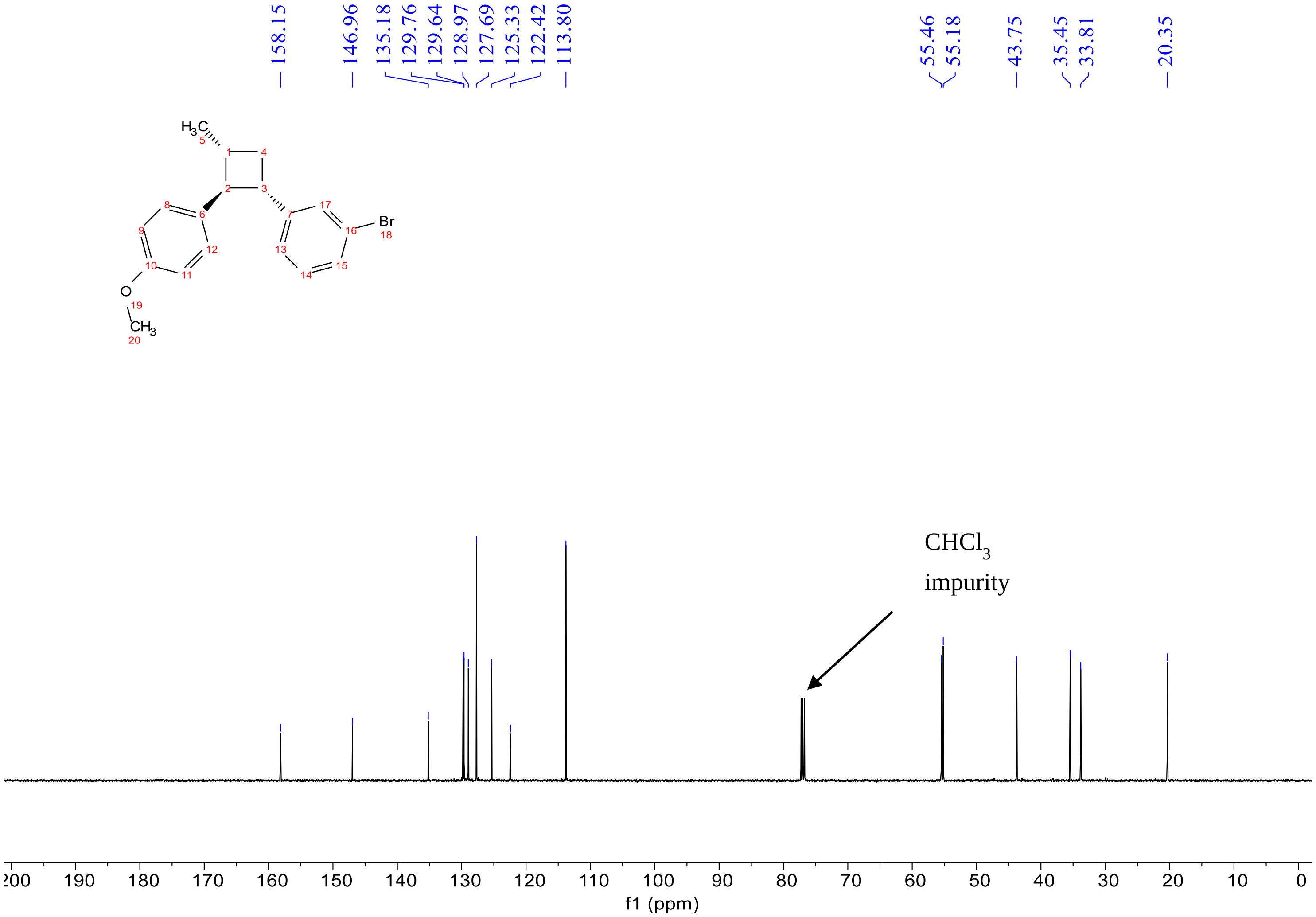

Supplementary Figure 87. ^{13}C NMR of **4f** (126 MHz, Chloroform-*d*)

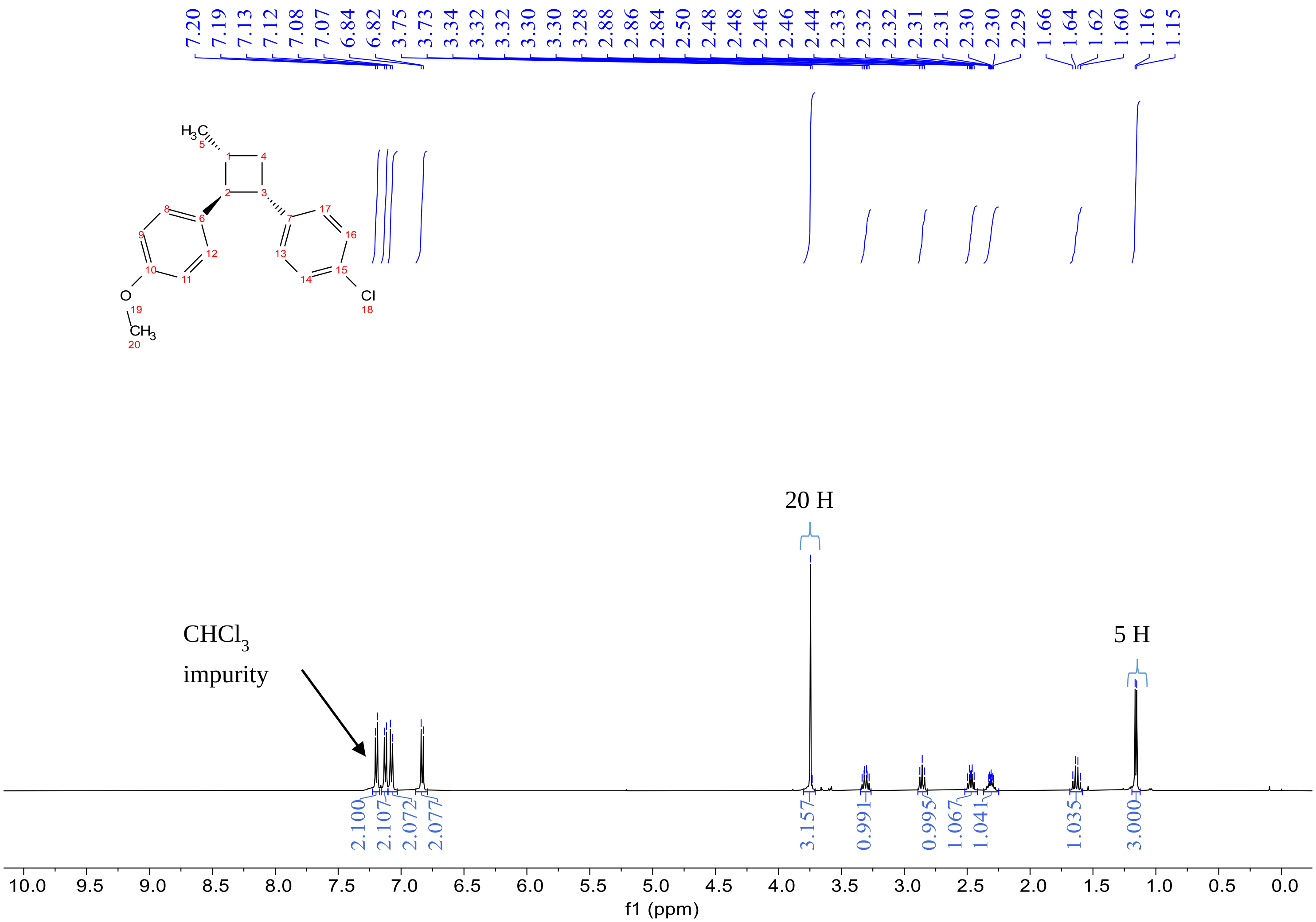

Supplementary Figure 88. ^{1}H NMR of **4g** (500 MHz, Chloroform-*d*)

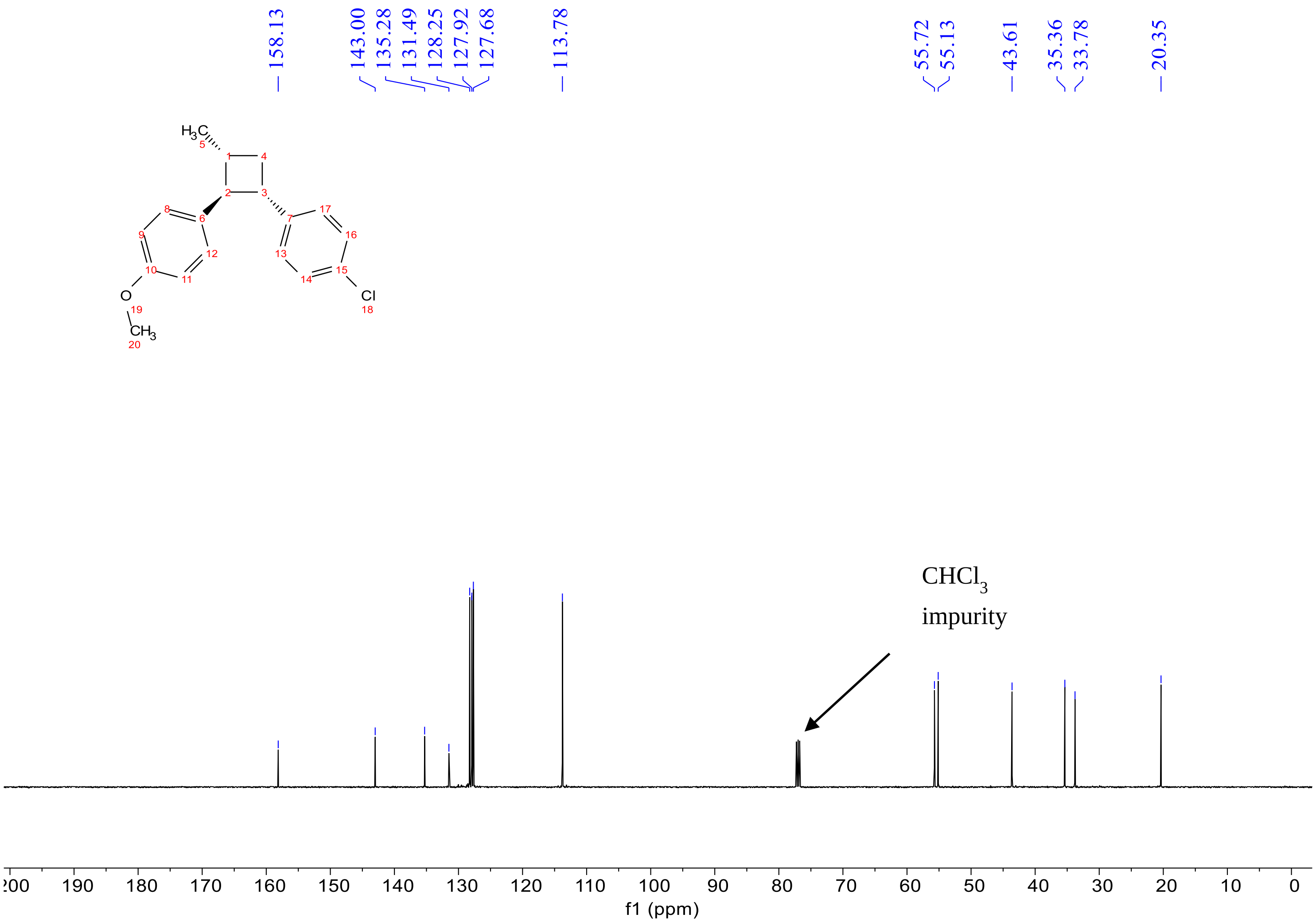

Supplementary Figure 89. ^{13}C NMR of **4g** (126 MHz, Chloroform-*d*)

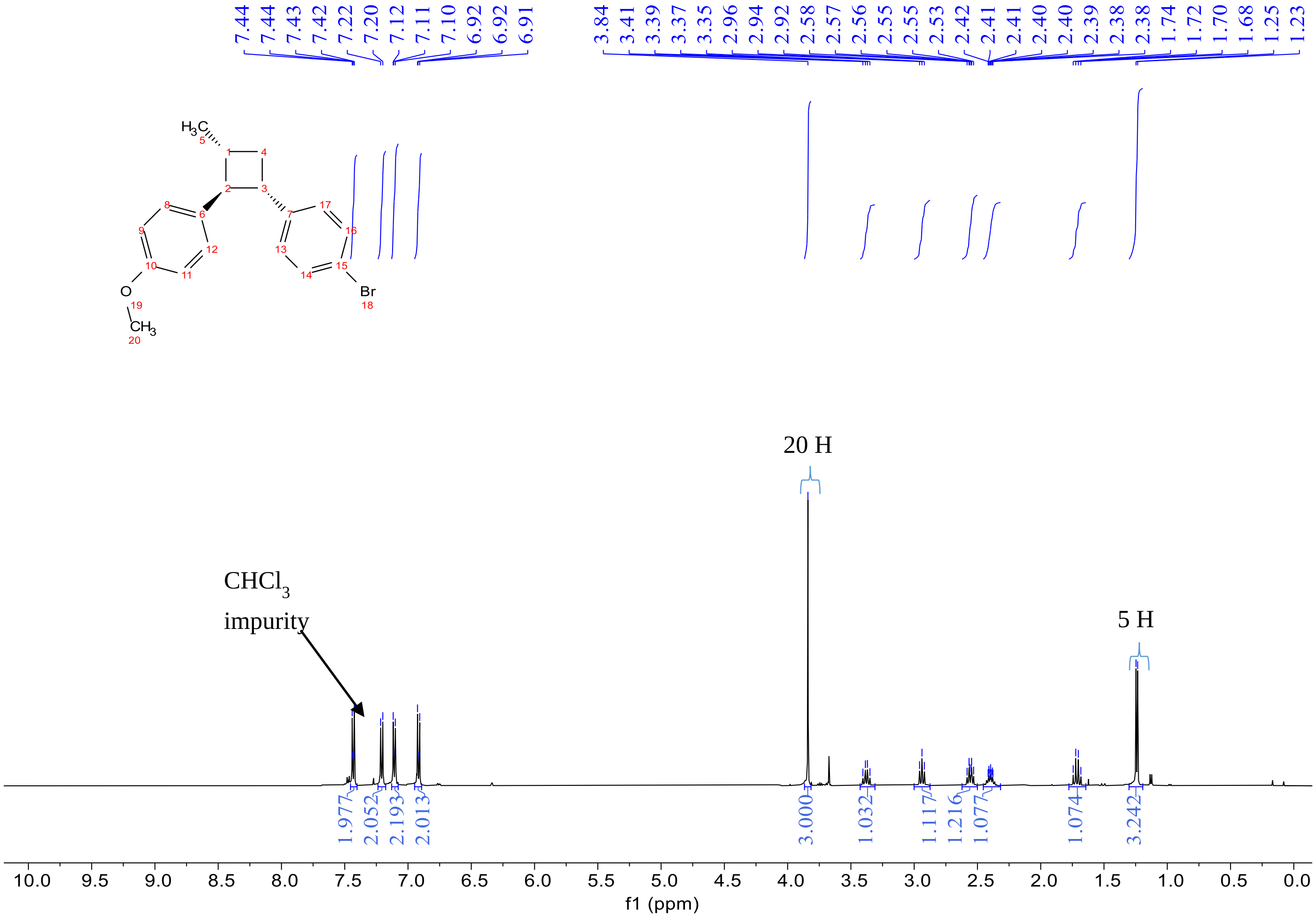

Supplementary Figure 90. ^{1}H NMR of **4h** (500 MHz, Chloroform-*d*)

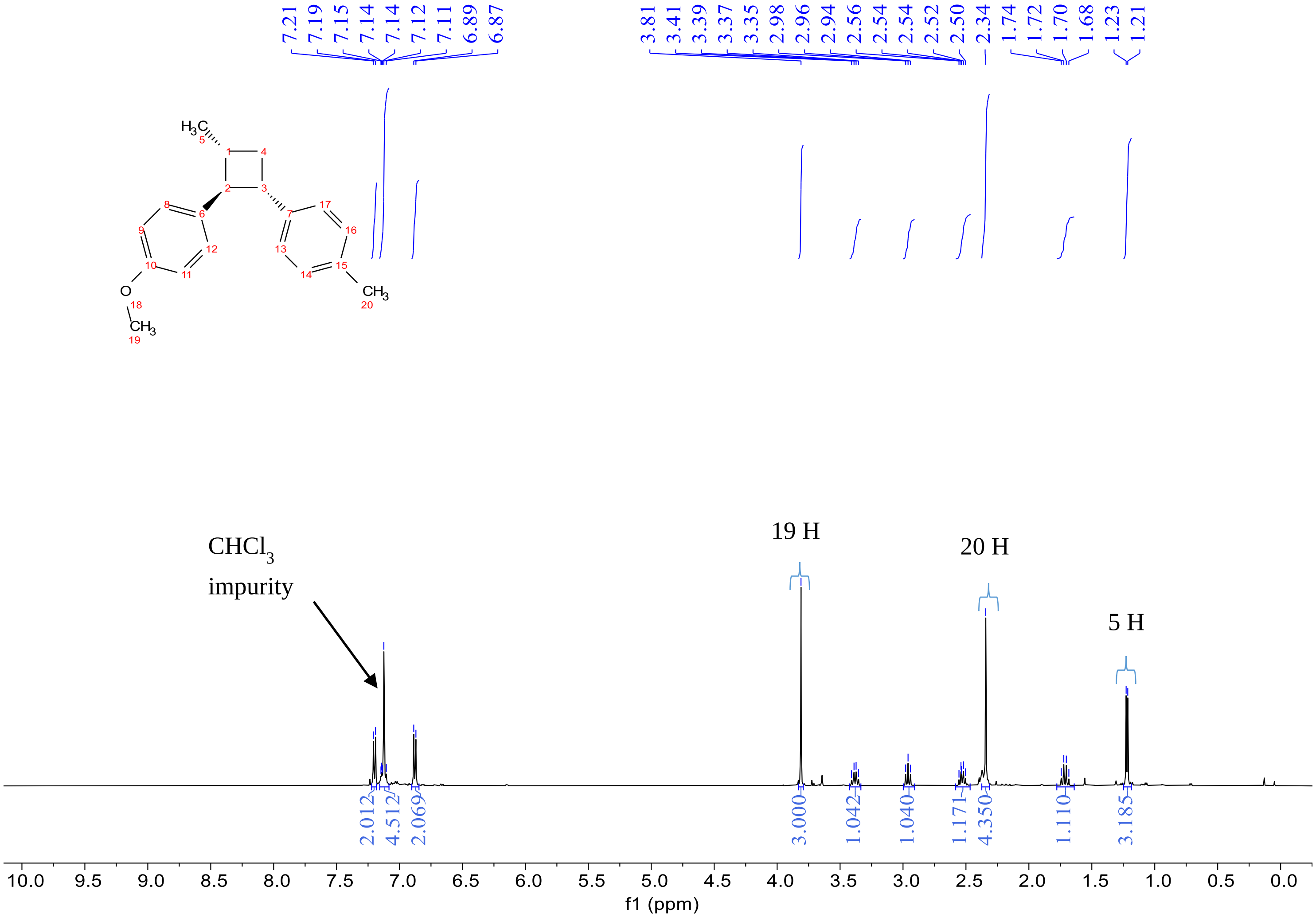

Supplementary Figure 91. ^{1}H NMR of **4i** (500 MHz, Chloroform-*d*)

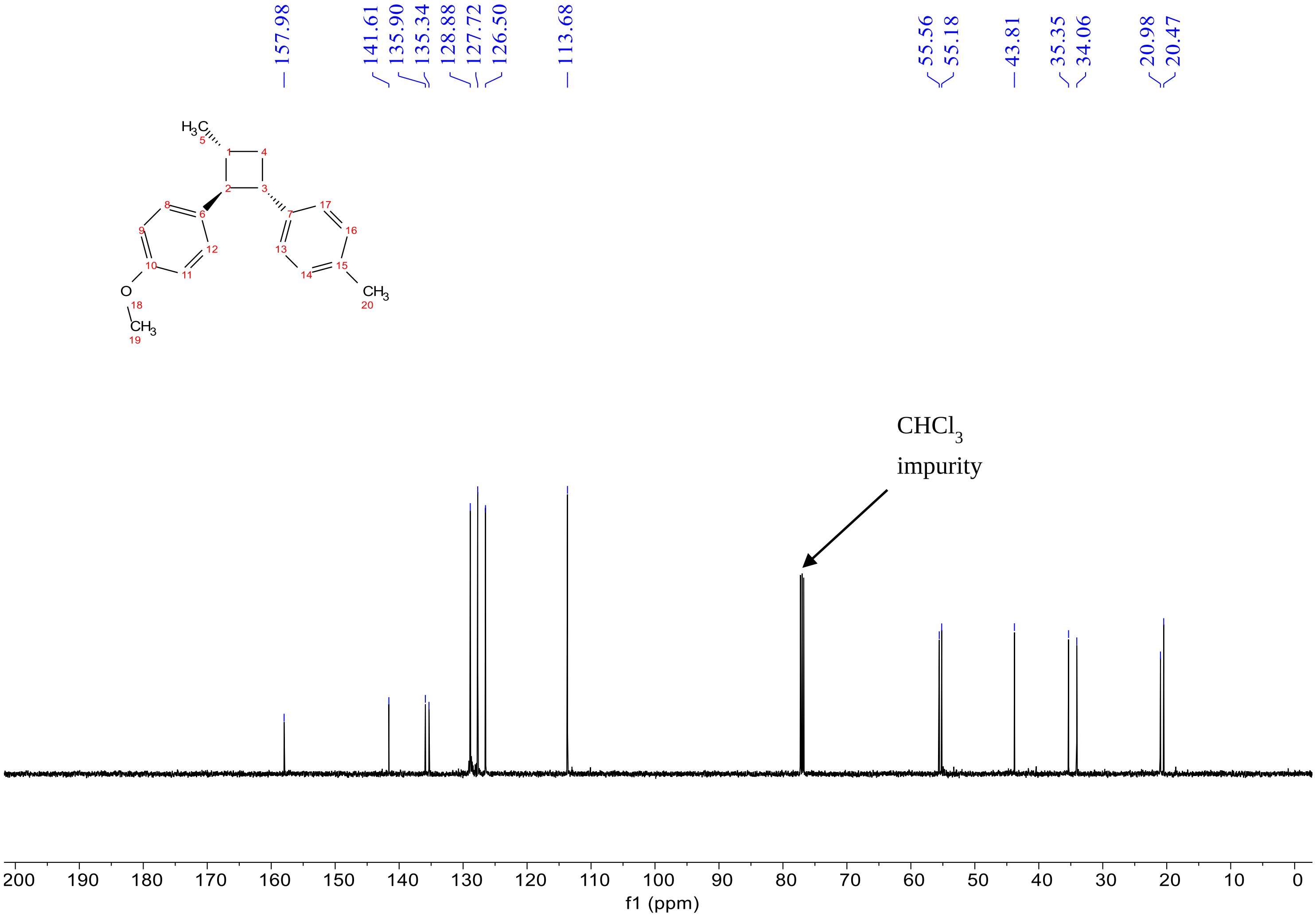

Supplementary Figure 92. ^{13}C NMR of **4i** (126 MHz, Chloroform-*d*)

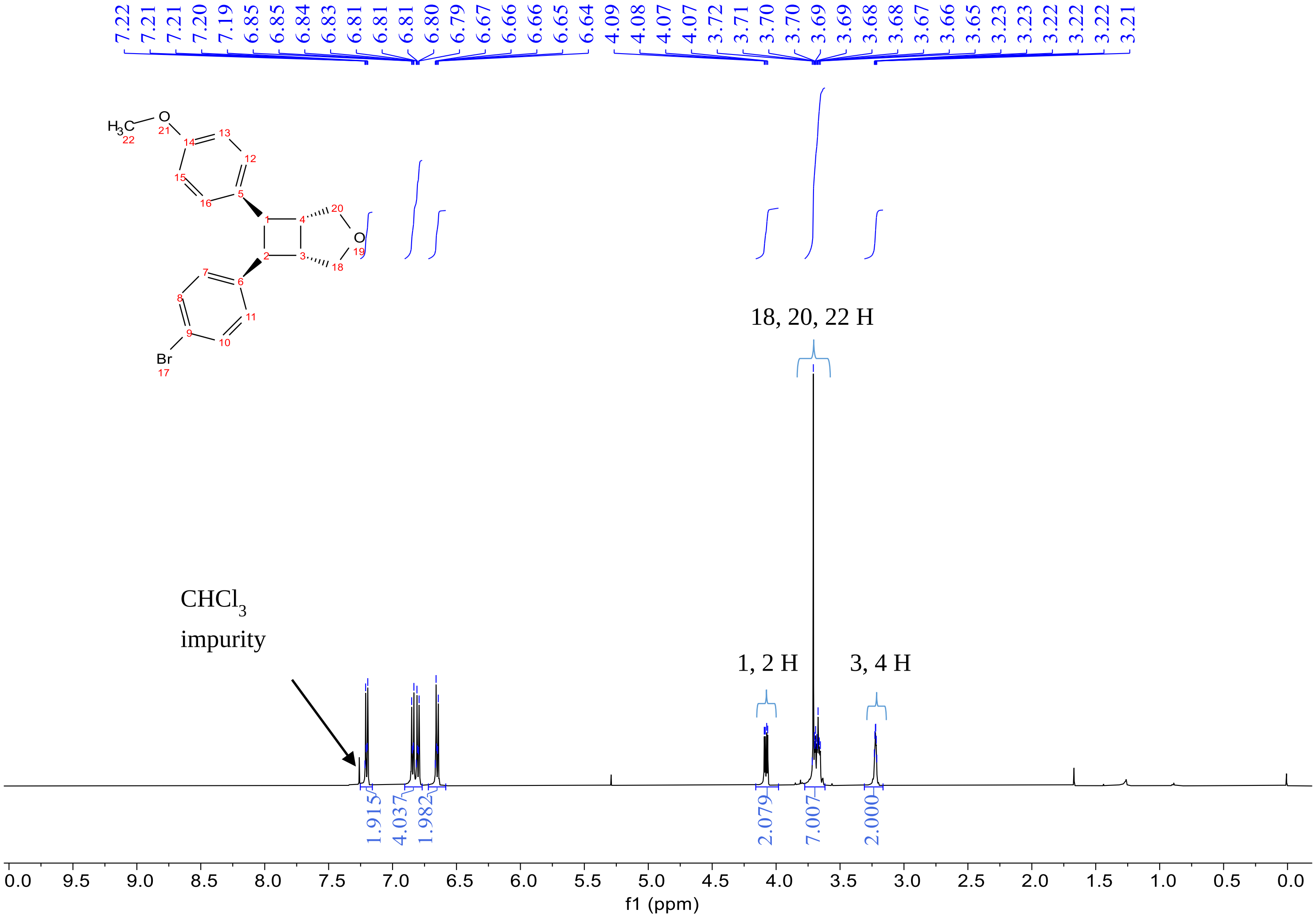

Supplementary Figure 93. ^{1}H NMR of **6a** (500 MHz, Chloroform-*d*)

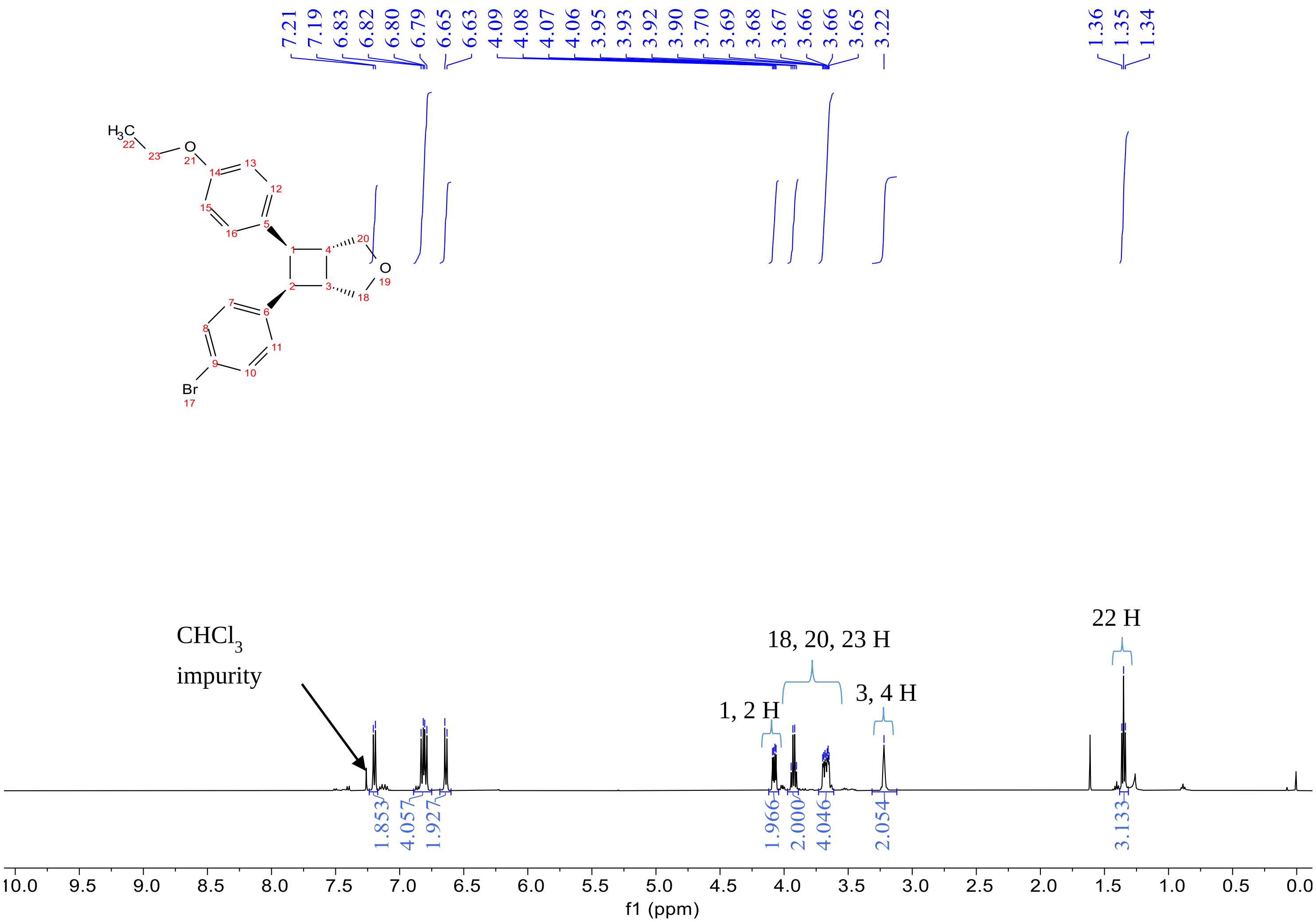

Supplementary Figure 94. ^{1}H NMR of **6b** (500 MHz, Chloroform-*d*)

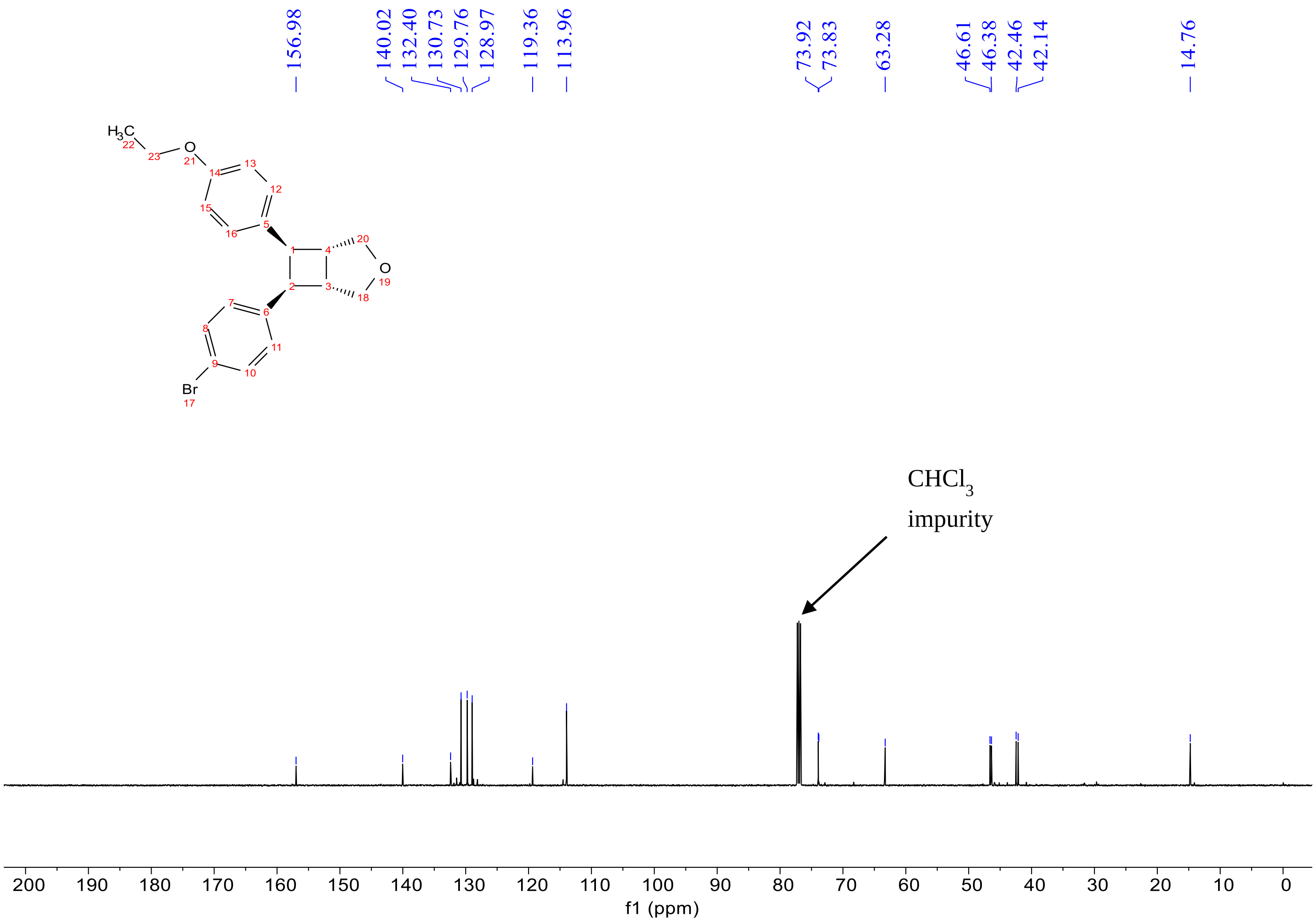

Supplementary Figure 95. ^{13}C NMR of **6b** (126 MHz, Chloroform-*d*)

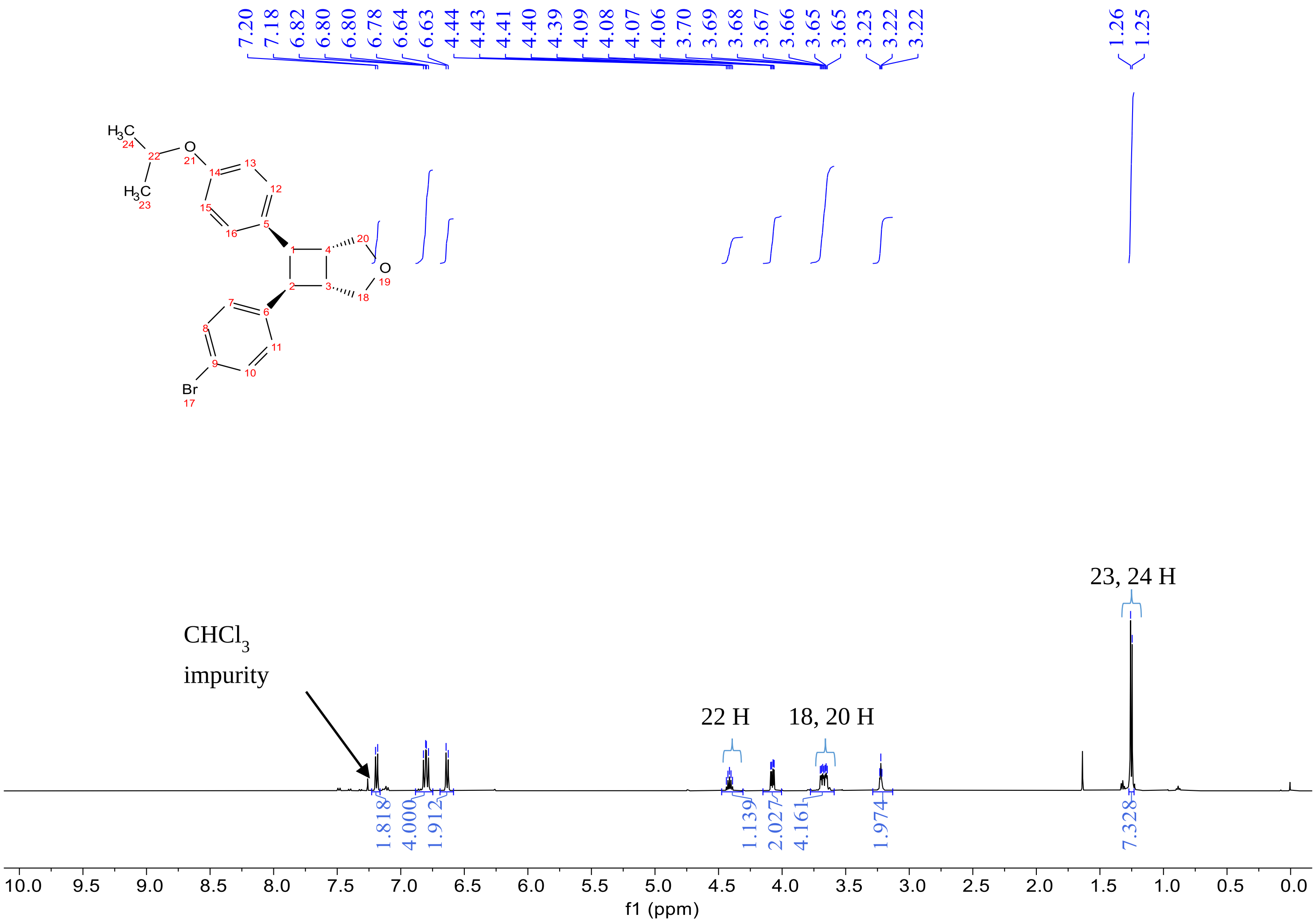

Supplementary Figure 96. ^{1}H NMR of **6c** (500 MHz, Chloroform-*d*)

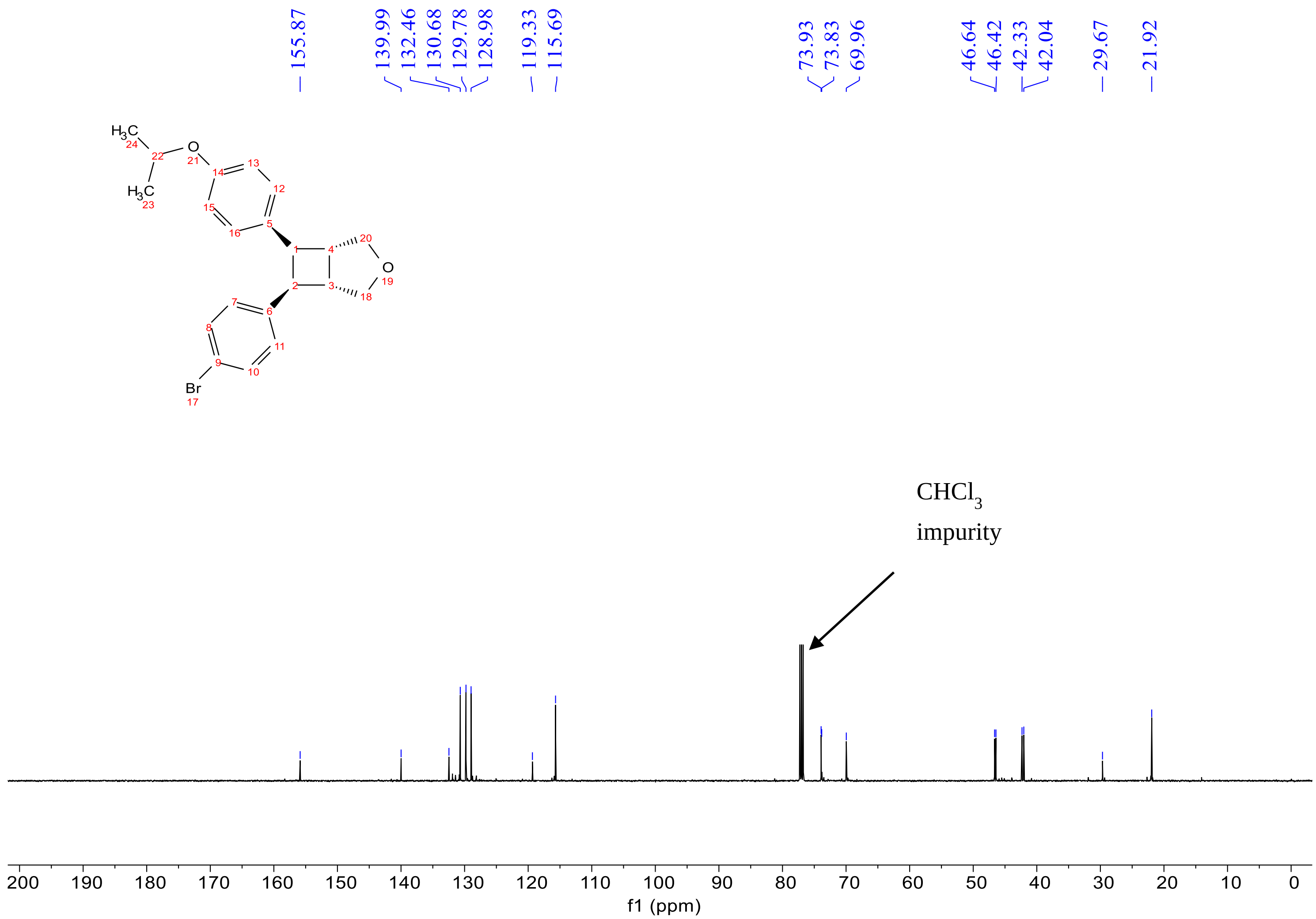

Supplementary Figure 97. ^{13}C NMR of **6c** (126 MHz, Chloroform-*d*)

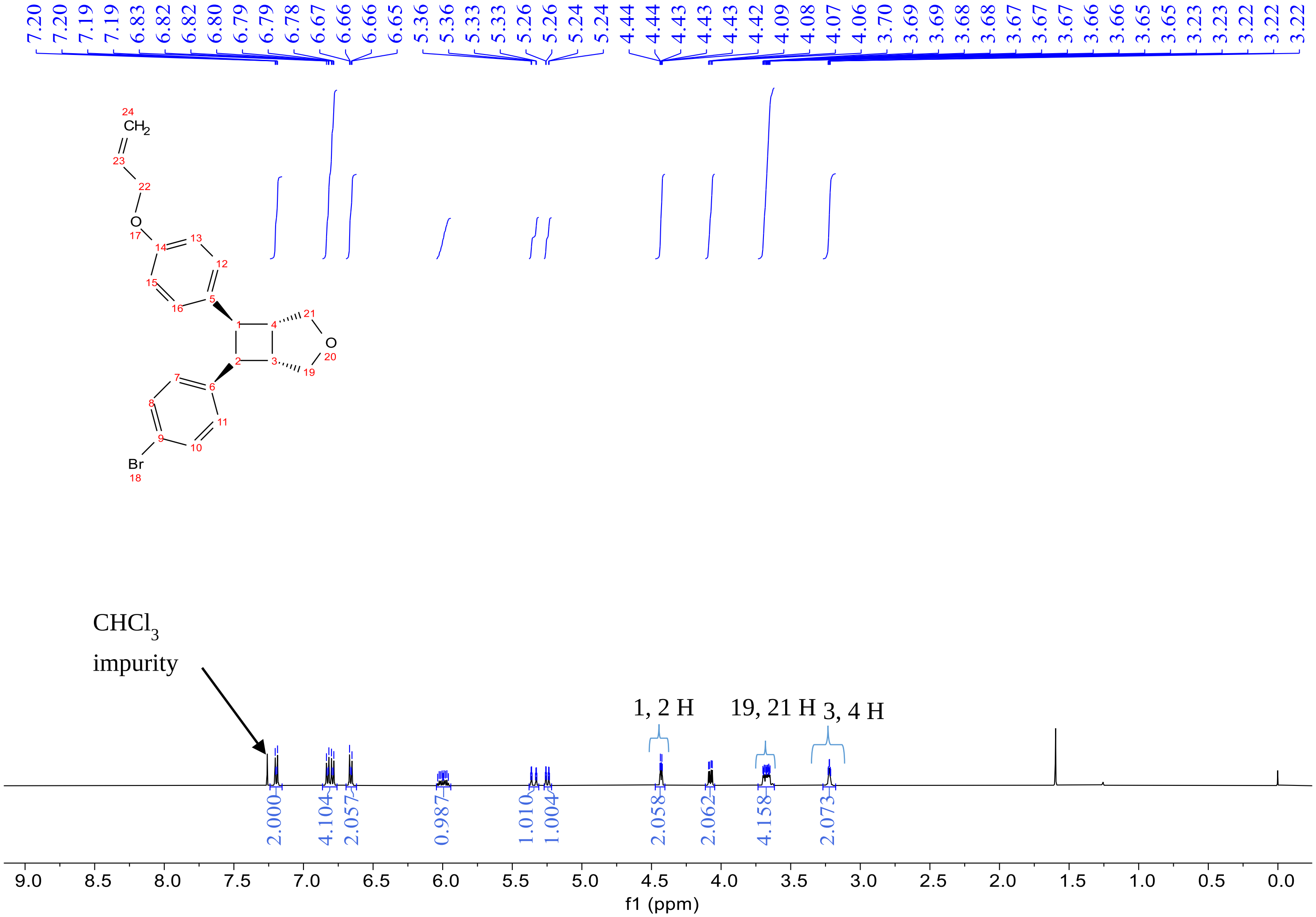

Supplementary Figure 98. ^{1}H NMR of **6d** (500 MHz, Chloroform-*d*)

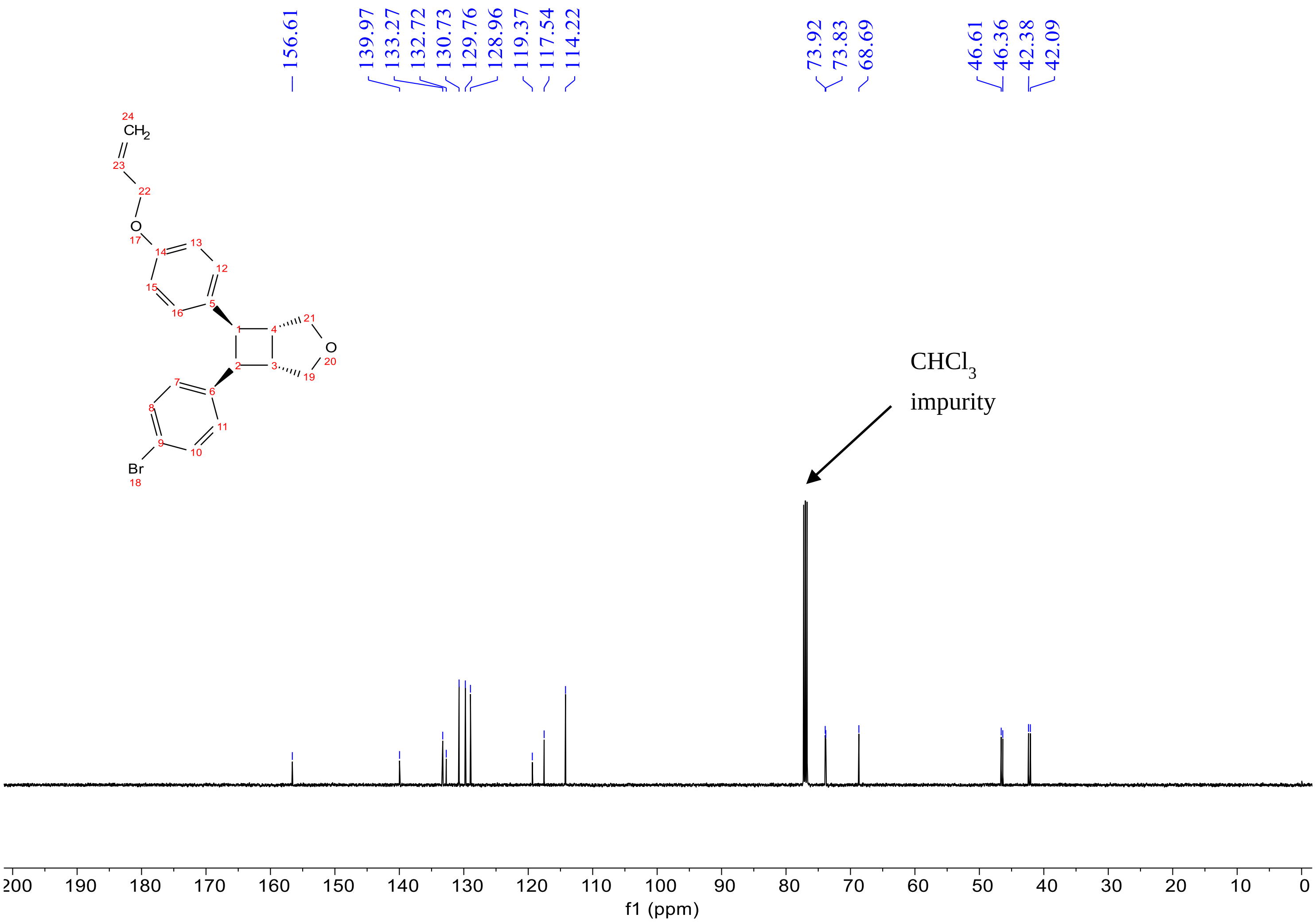

Supplementary Figure 99. ^{13}C NMR of **6d** (126 MHz, Chloroform-*d*)

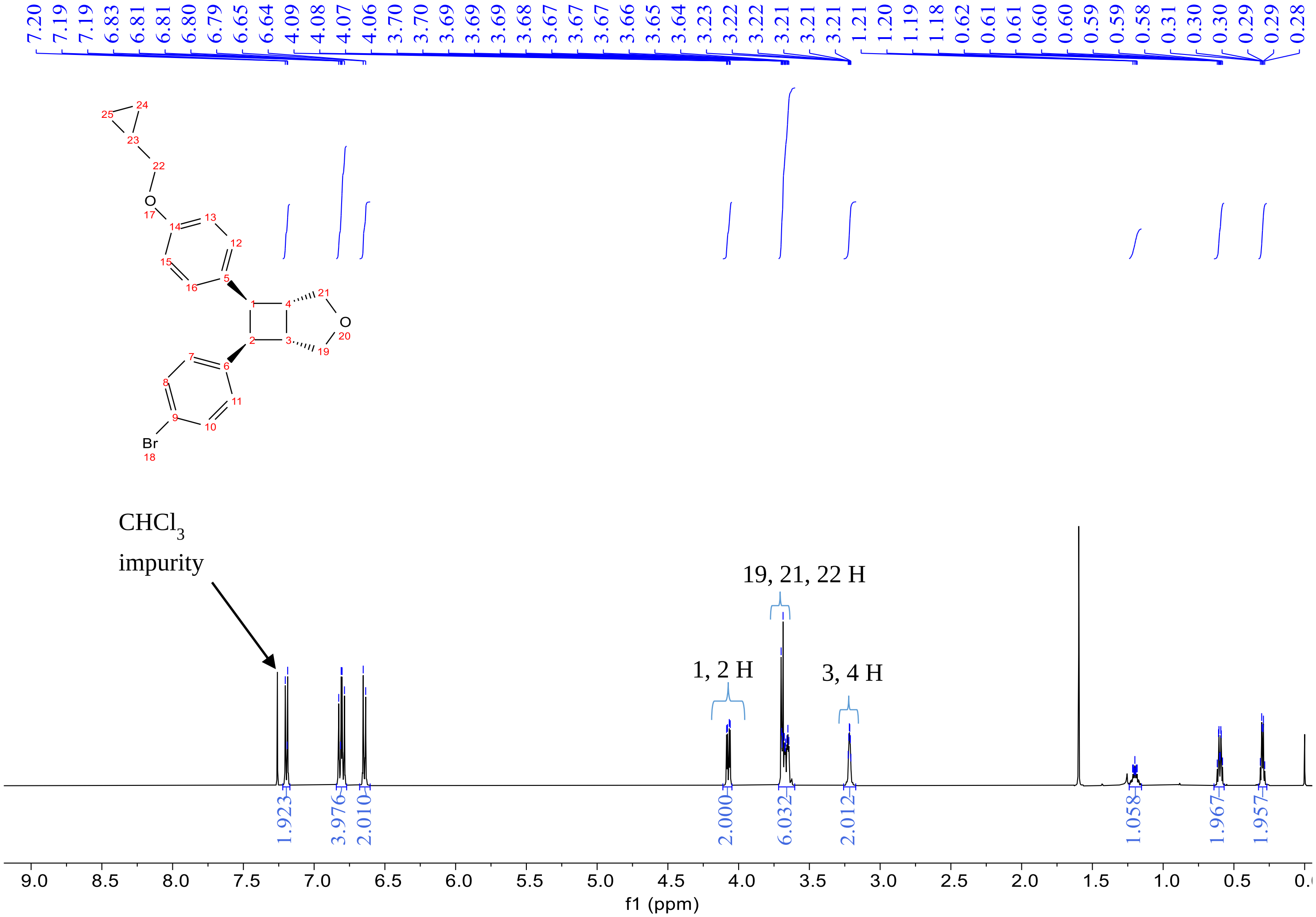

Supplementary Figure 100. ^{1}H NMR of **6e** (500 MHz, Chloroform-*d*)

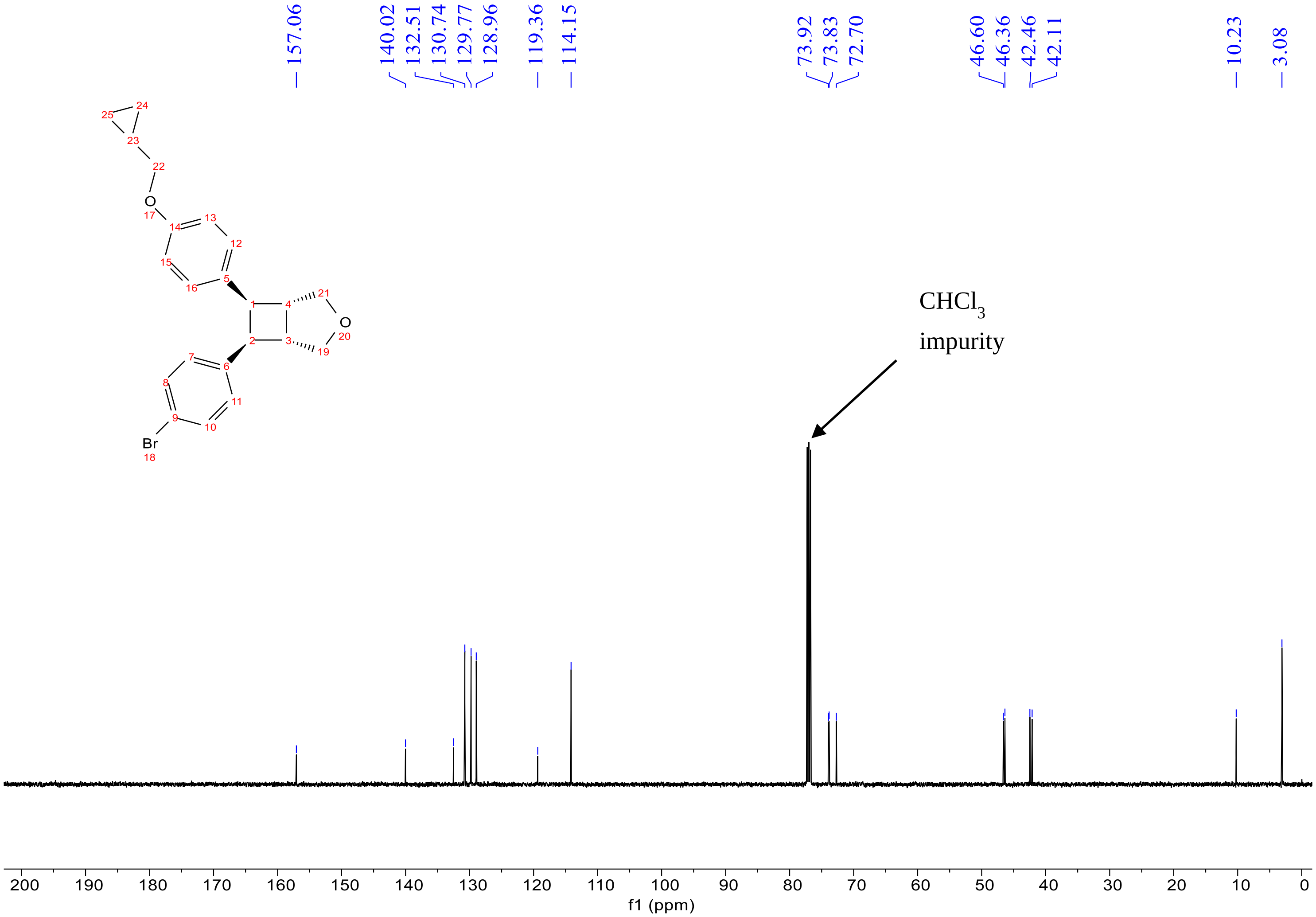

Supplementary Figure 101. ^{13}C NMR of **6e** (126 MHz, Chloroform-*d*)

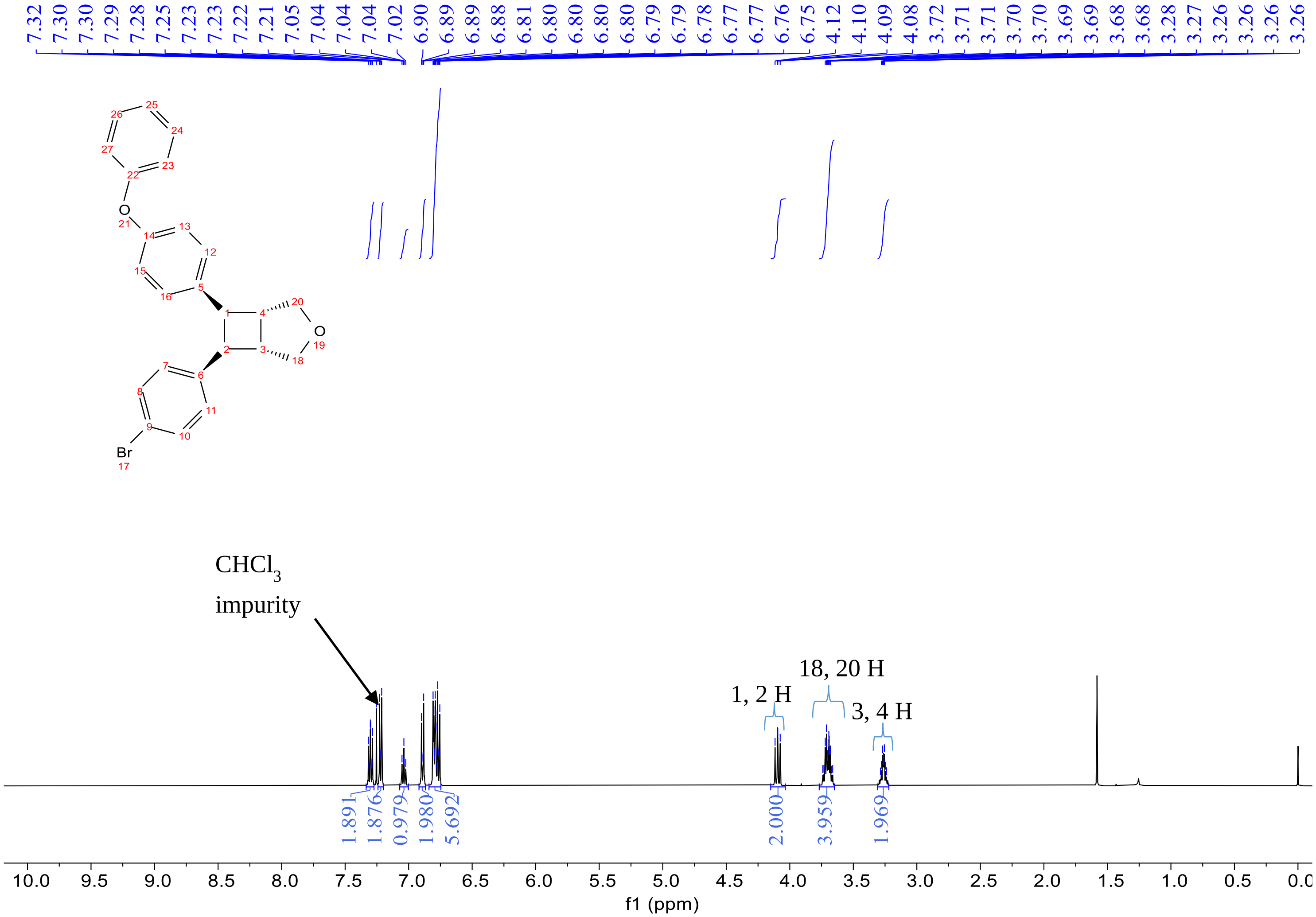

Supplementary Figure 102. ^{1}H NMR of **6f** (500 MHz, Chloroform-*d*)

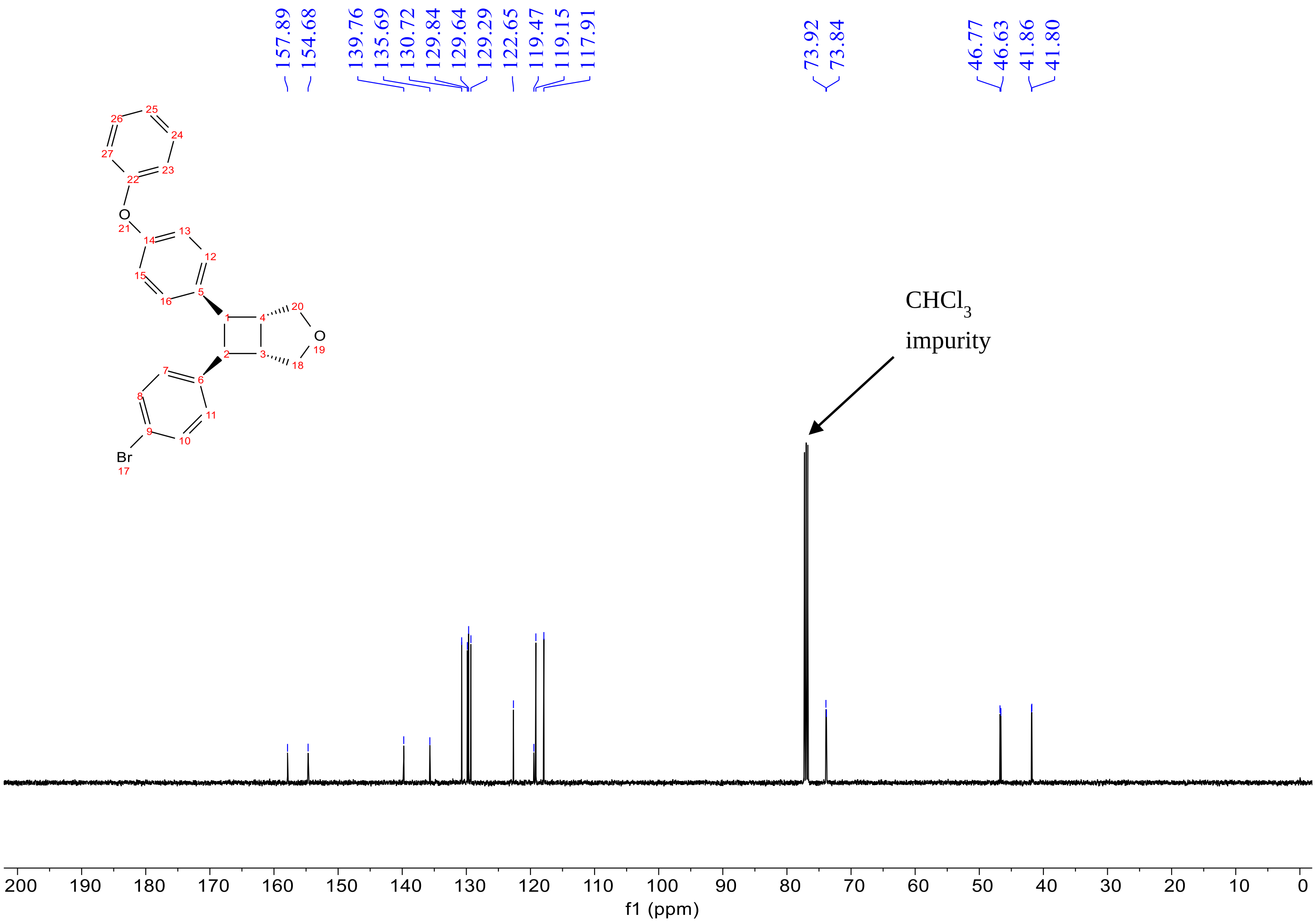

Supplementary Figure 103. ^{13}C NMR of **6f** (126 MHz, Chloroform-*d*)

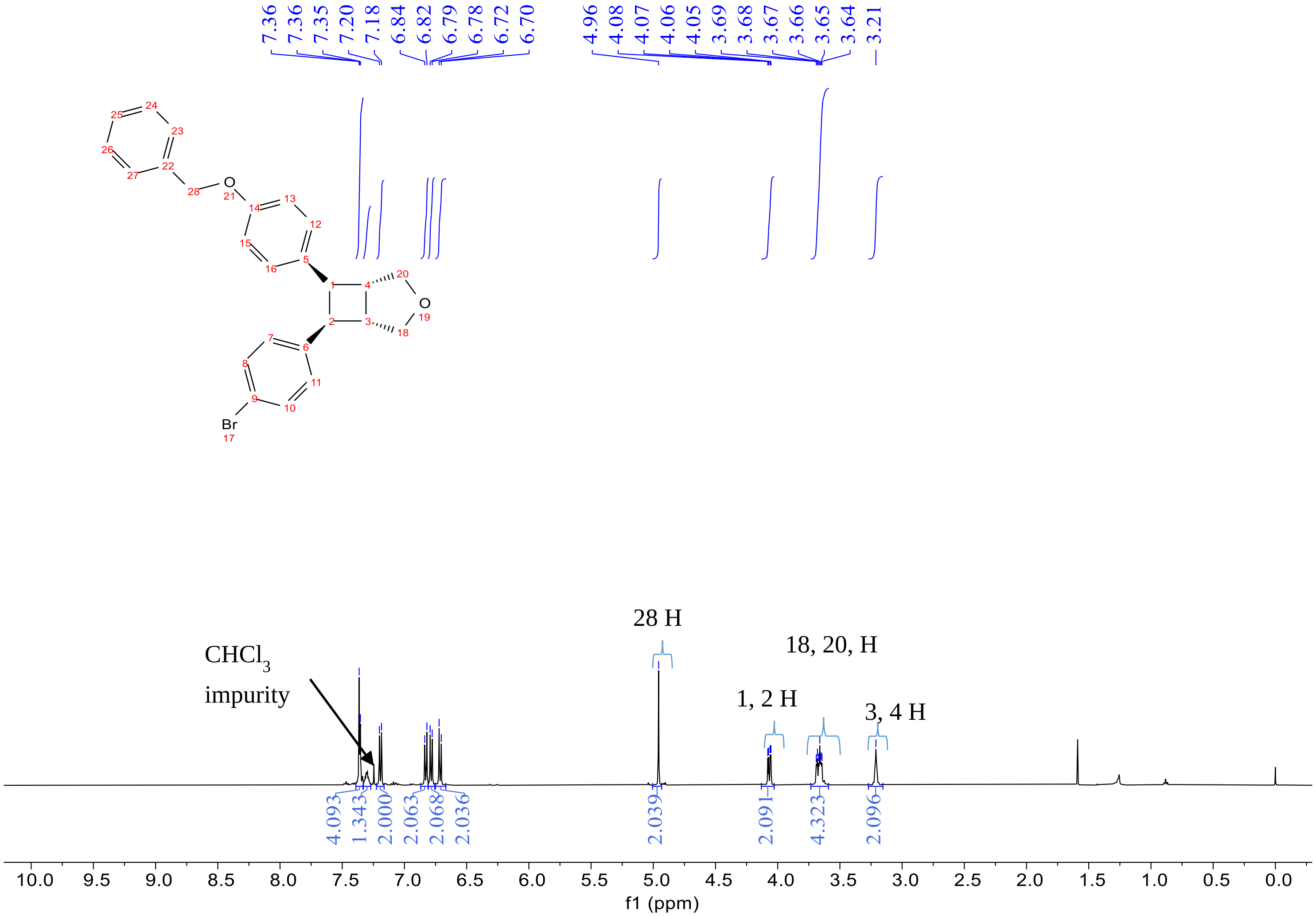

Supplementary Figure 104. ^{1}H NMR of **6g** (500 MHz, Chloroform-*d*)

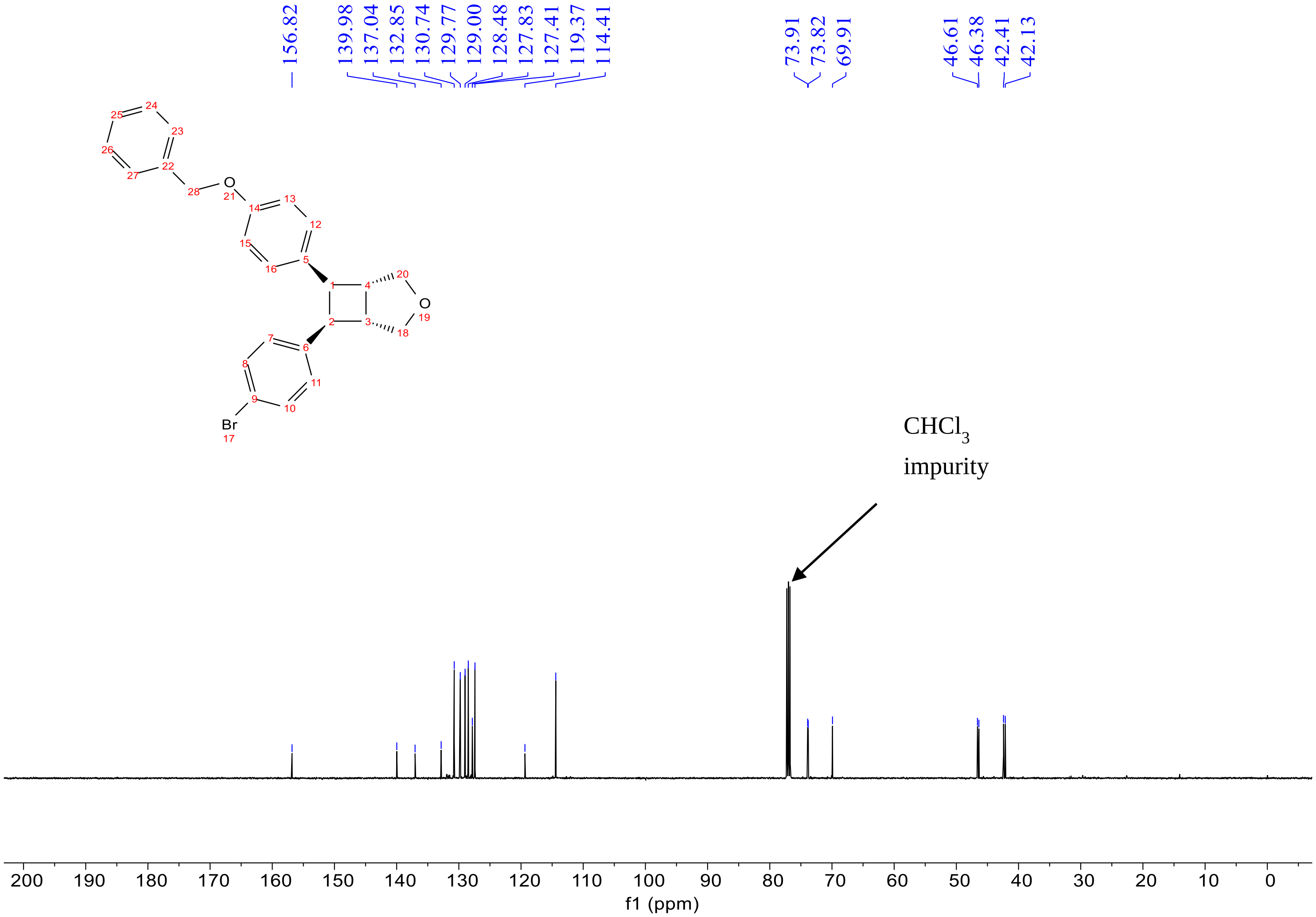

Supplementary Figure 105. ^{13}C NMR of **6g** (126 MHz, Chloroform-*d*)

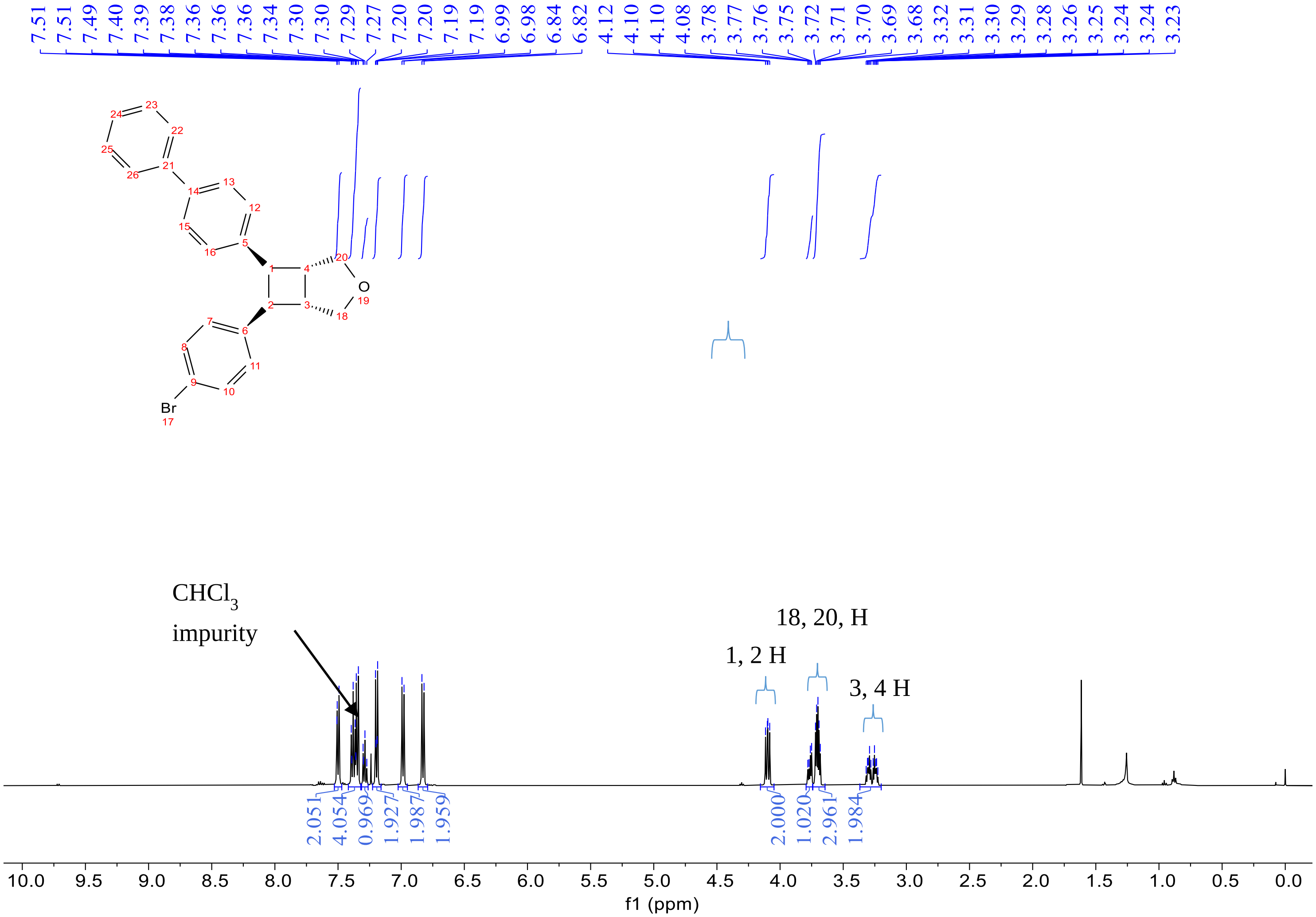

Supplementary Figure 106. ^{1}H NMR of **6h** (500 MHz, Chloroform-*d*)

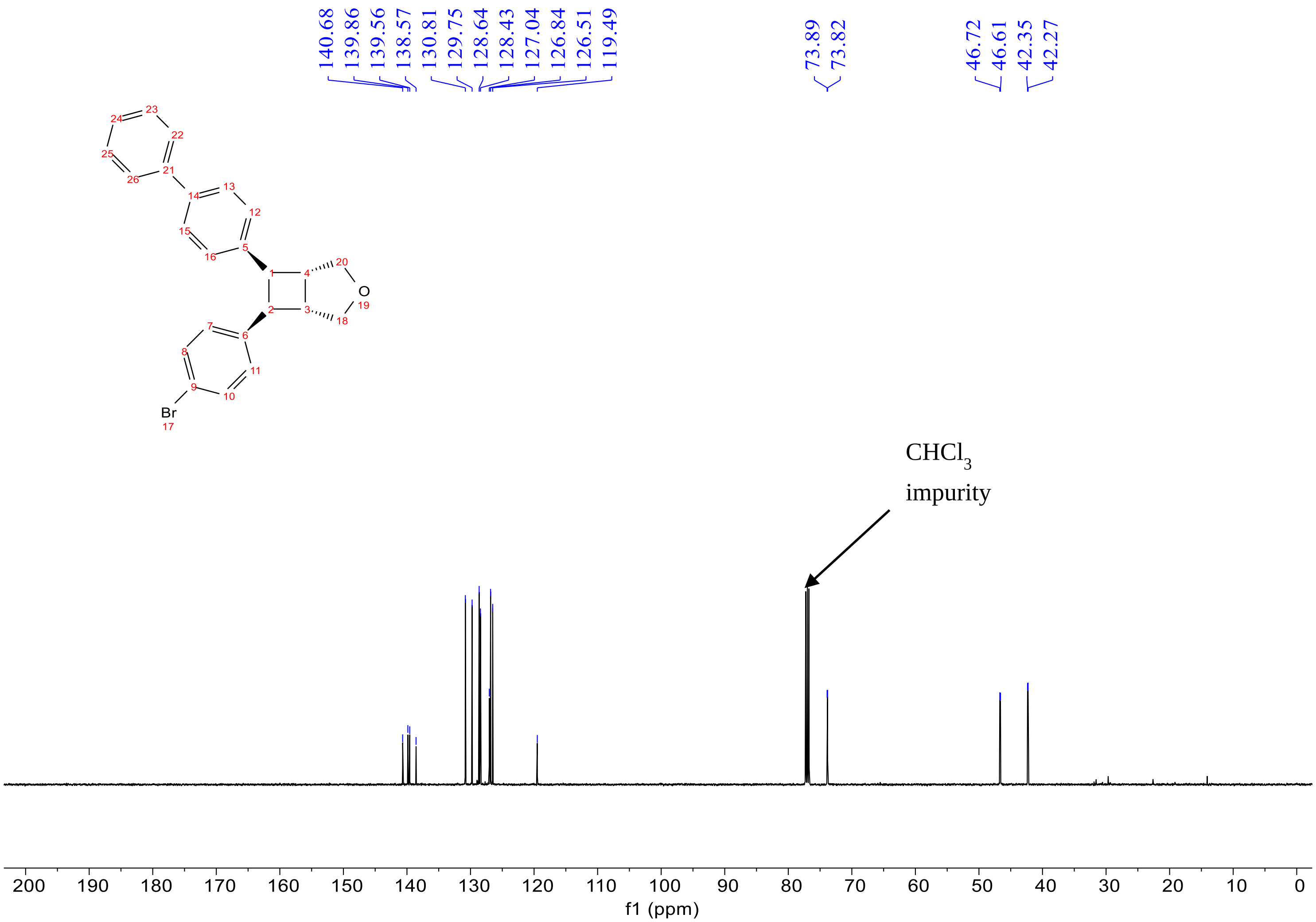

Supplementary Figure 107. ^{13}C NMR of **6h** (126 MHz, Chloroform-*d*)

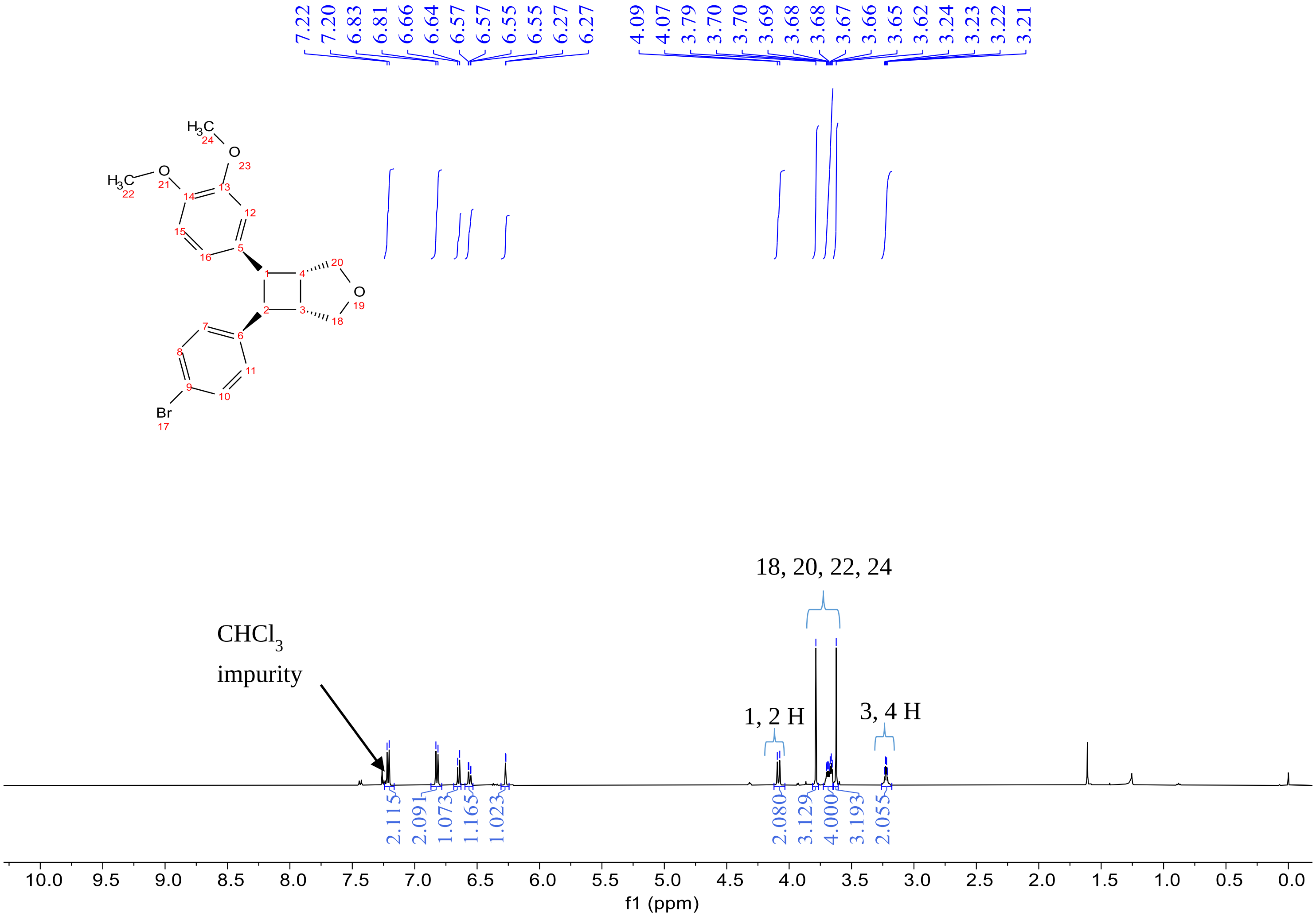

Supplementary Figure 108. ^{1}H NMR of **6i** (500 MHz, Chloroform-*d*)

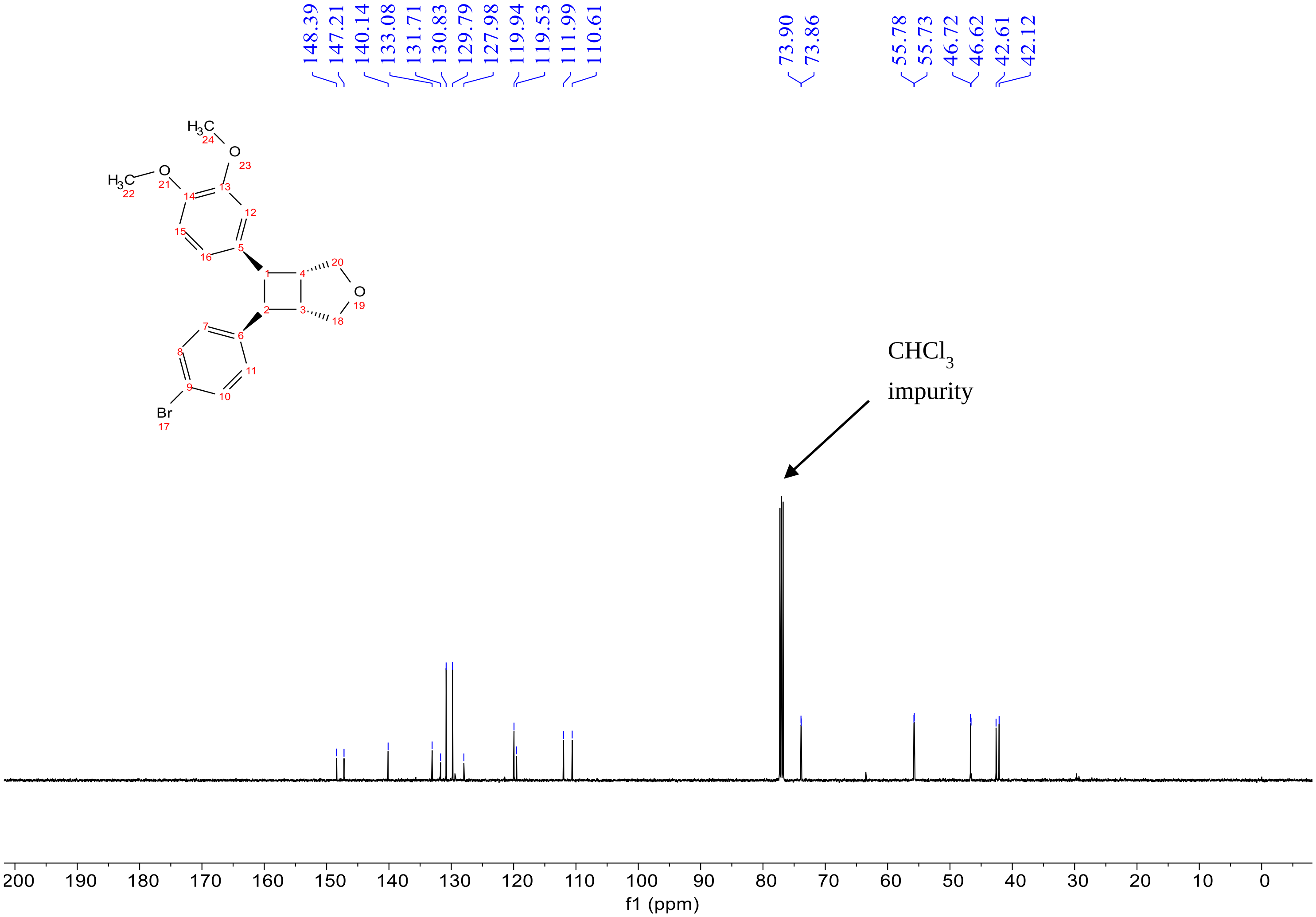

Supplementary Figure 109. ^{13}C NMR of **6i** (126 MHz, Chloroform-*d*)

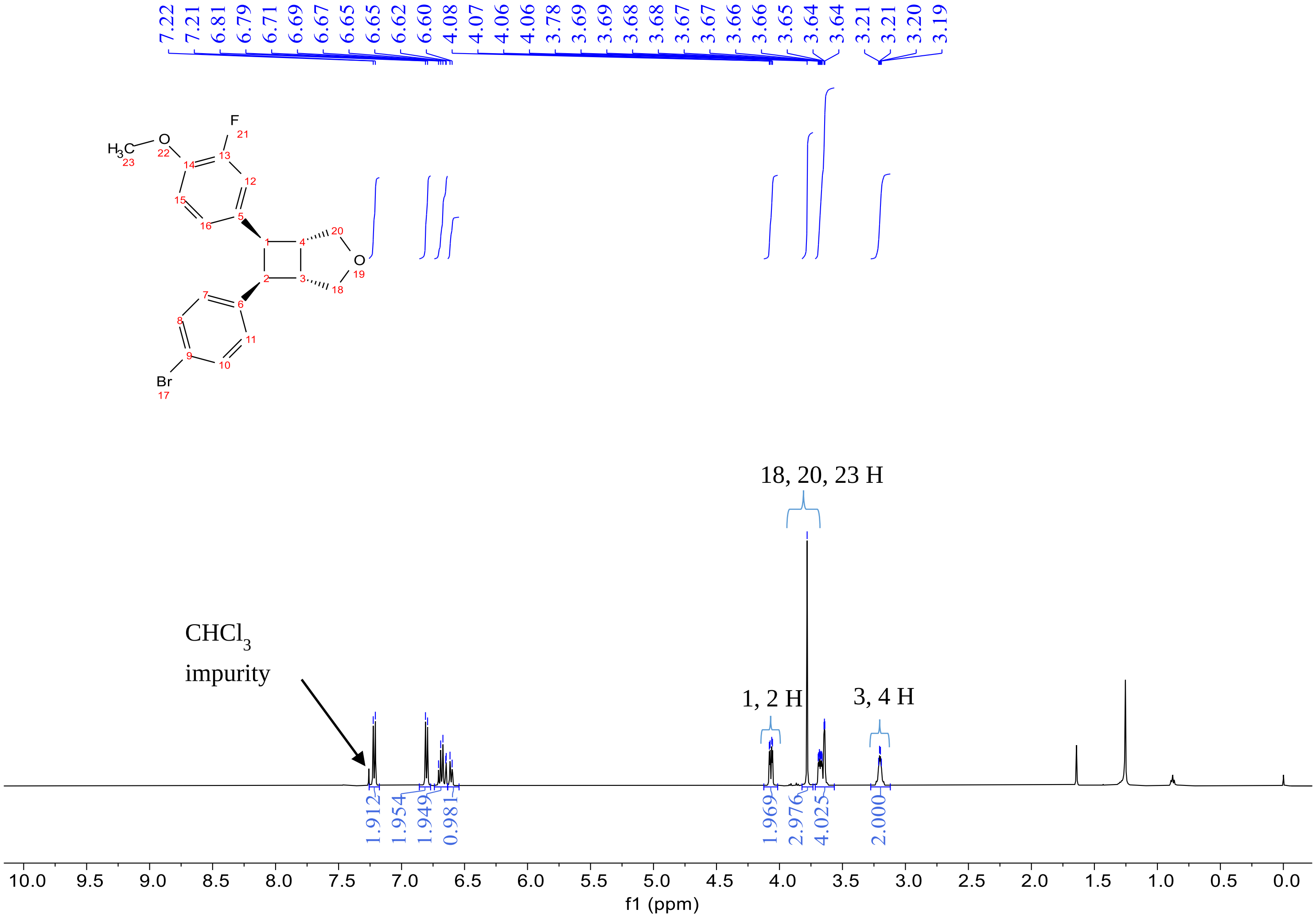

Supplementary Figure 110. ^{1}H NMR of **6j** (500 MHz, Chloroform-*d*)

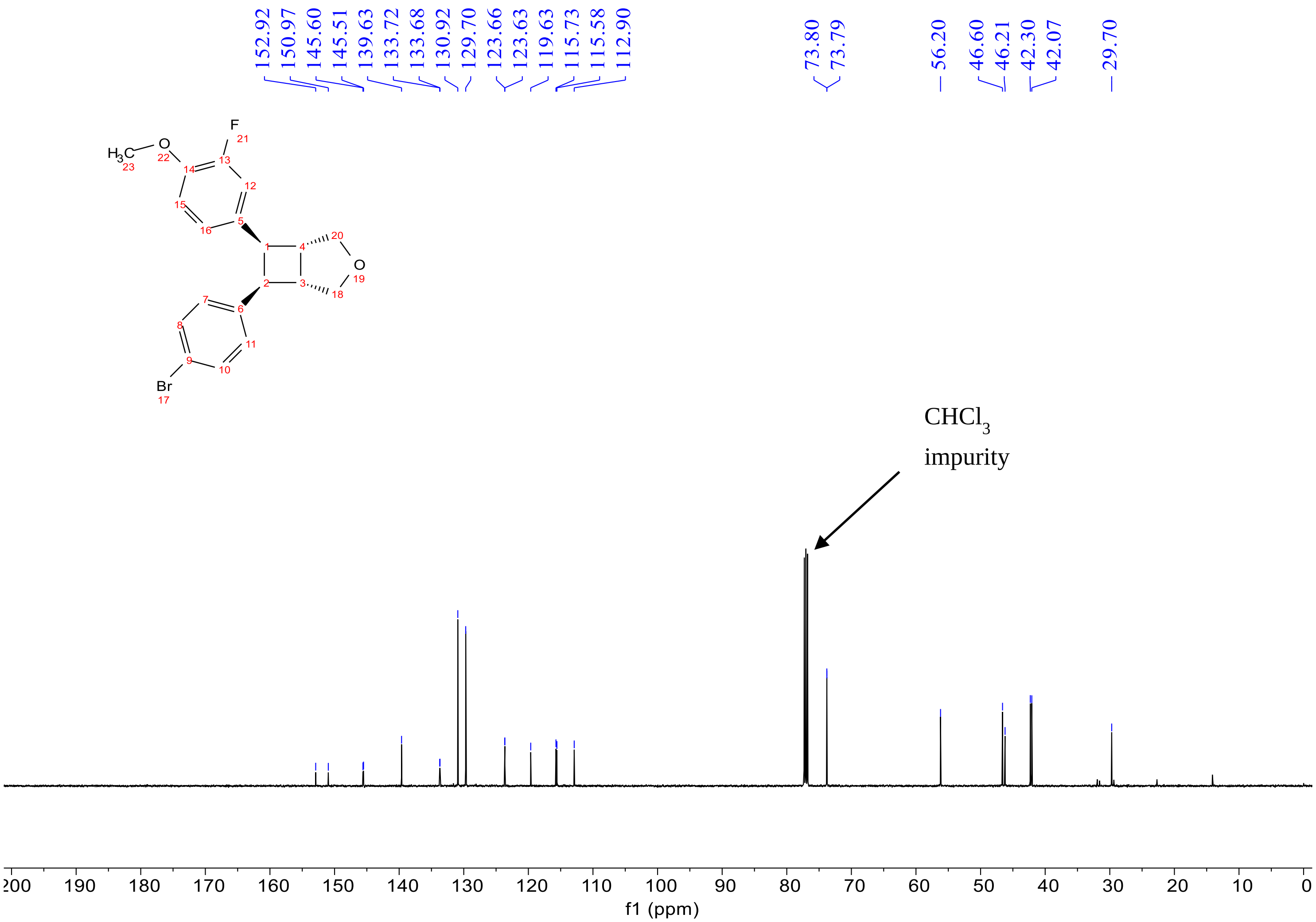

Supplementary Figure 111. ^{13}C NMR of **6j** (126 MHz, Chloroform-*d*)

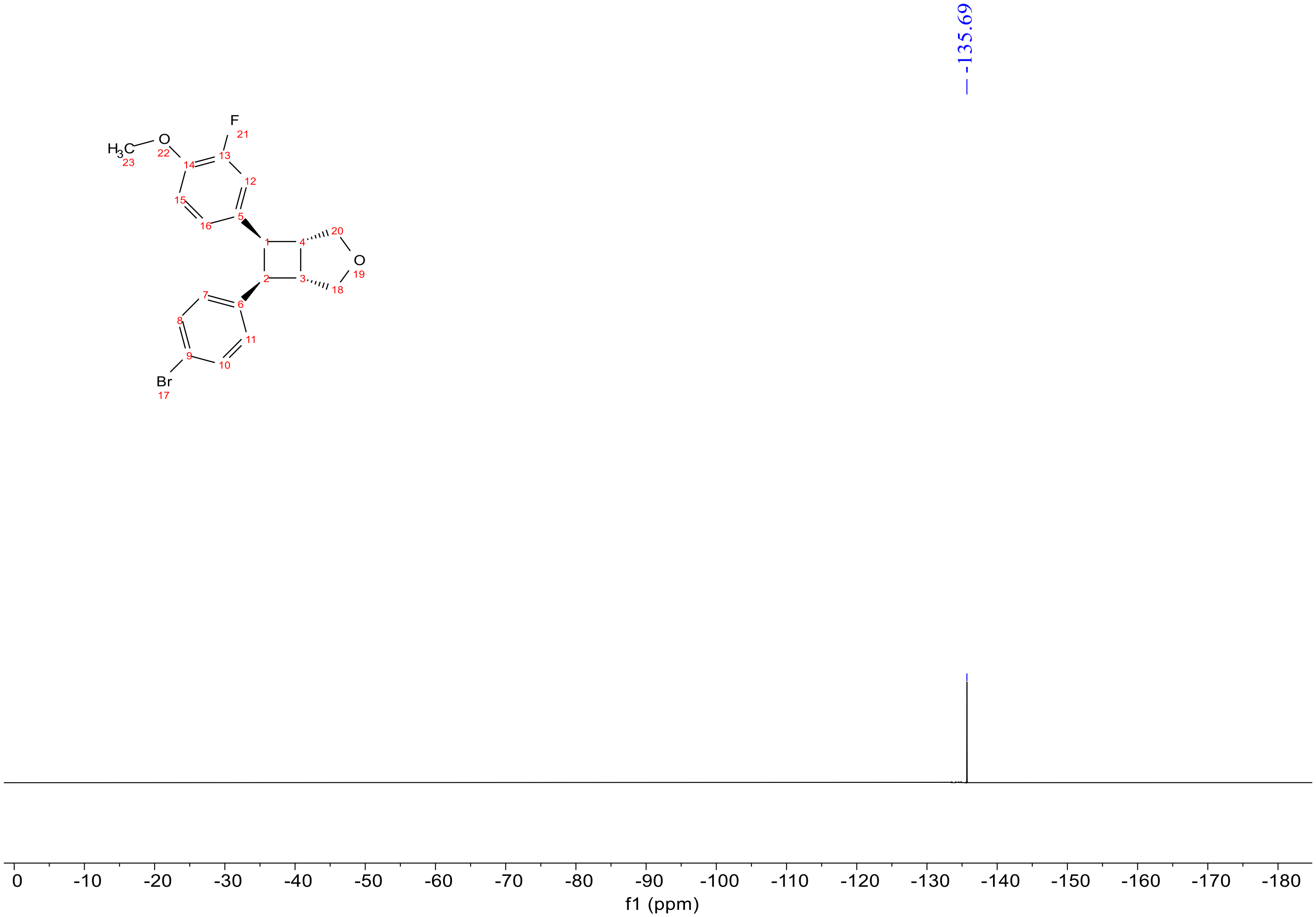

Supplementary Figure 112. ^{19}F NMR of **6j** (471 MHz, Chloroform-*d*)

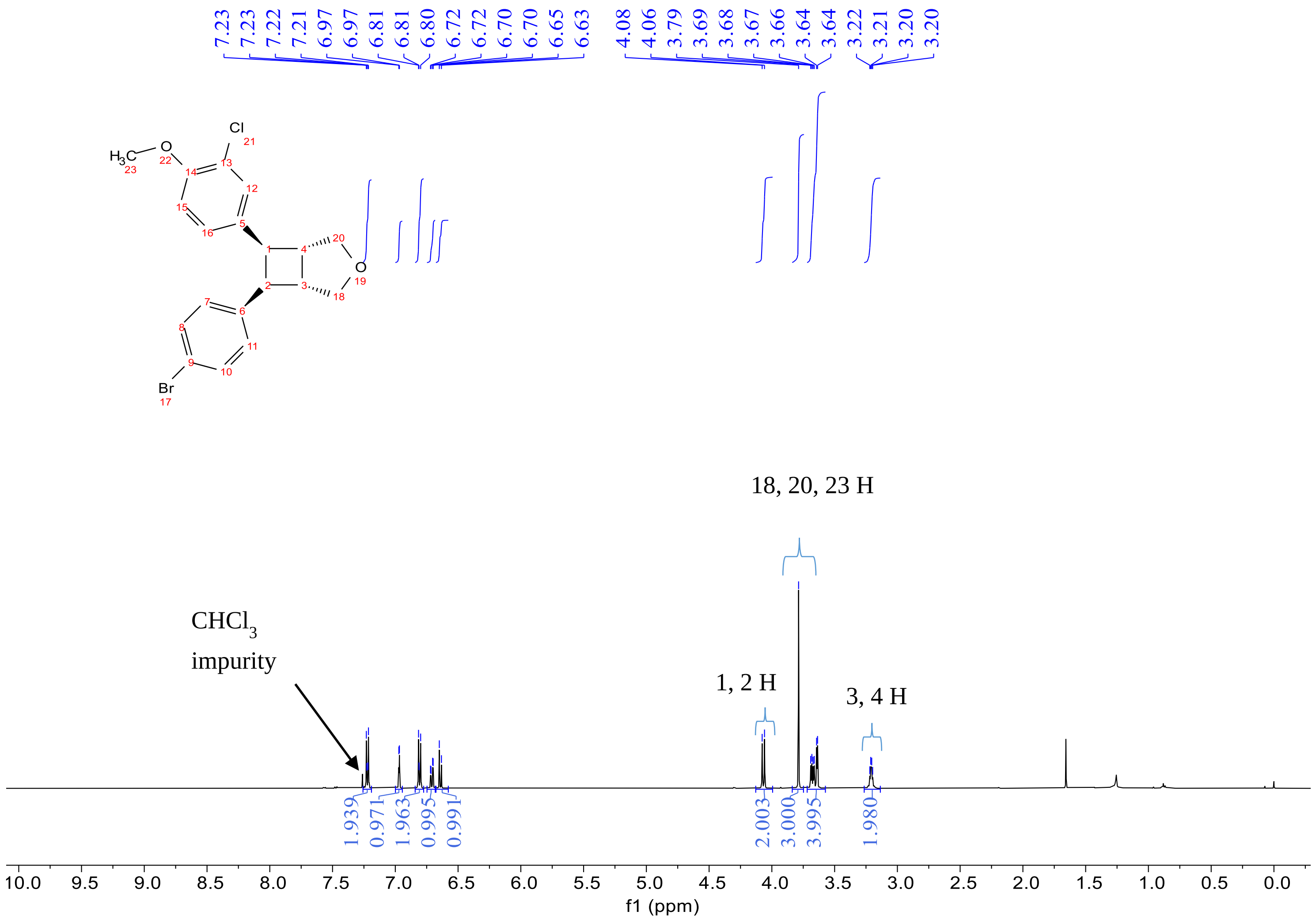

Supplementary Figure 113. ^{1}H NMR of **6k** (500 MHz, Chloroform-*d*)

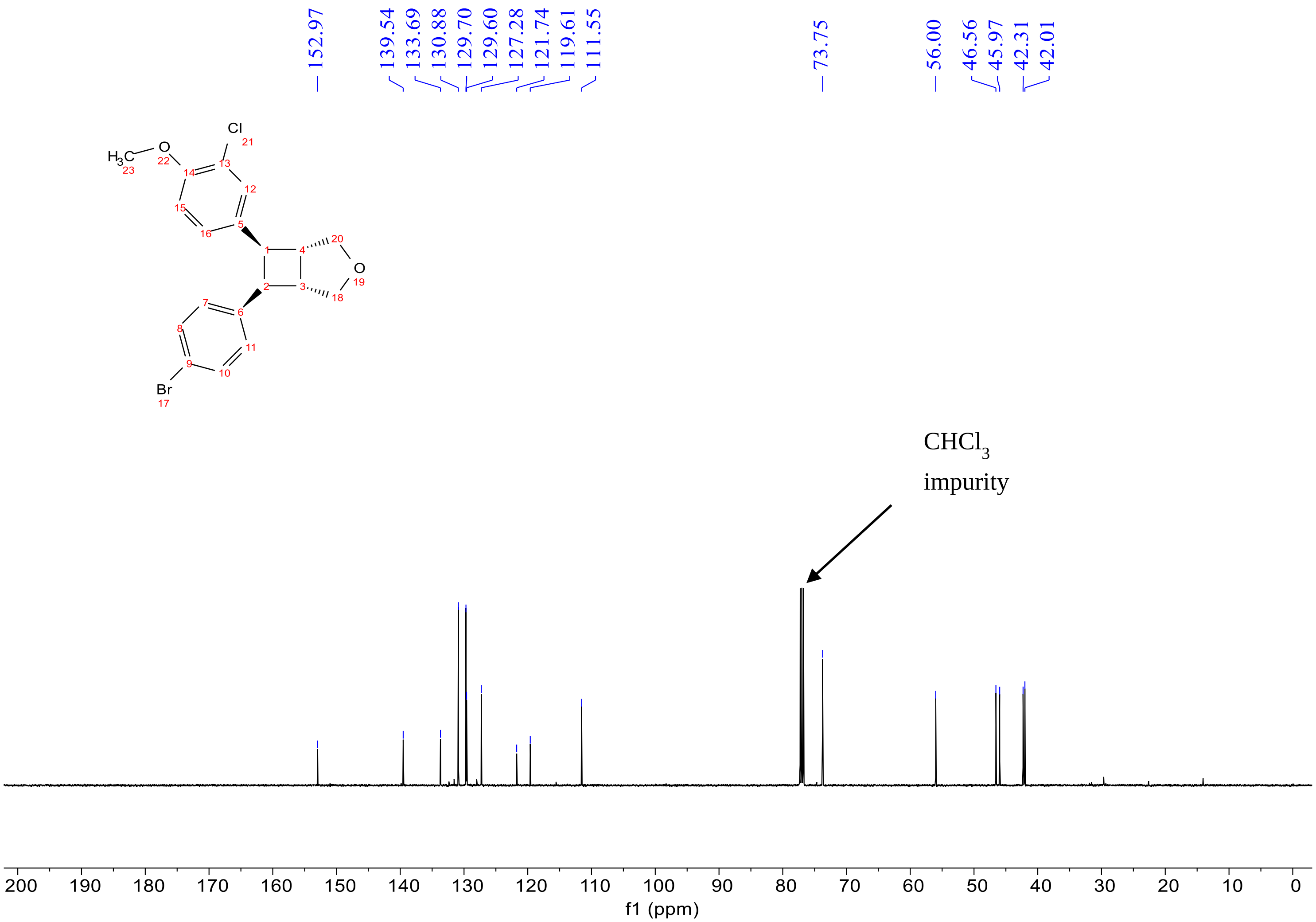

Supplementary Figure 114. ^{13}C NMR of **6k** (126 MHz, Chloroform-*d*)

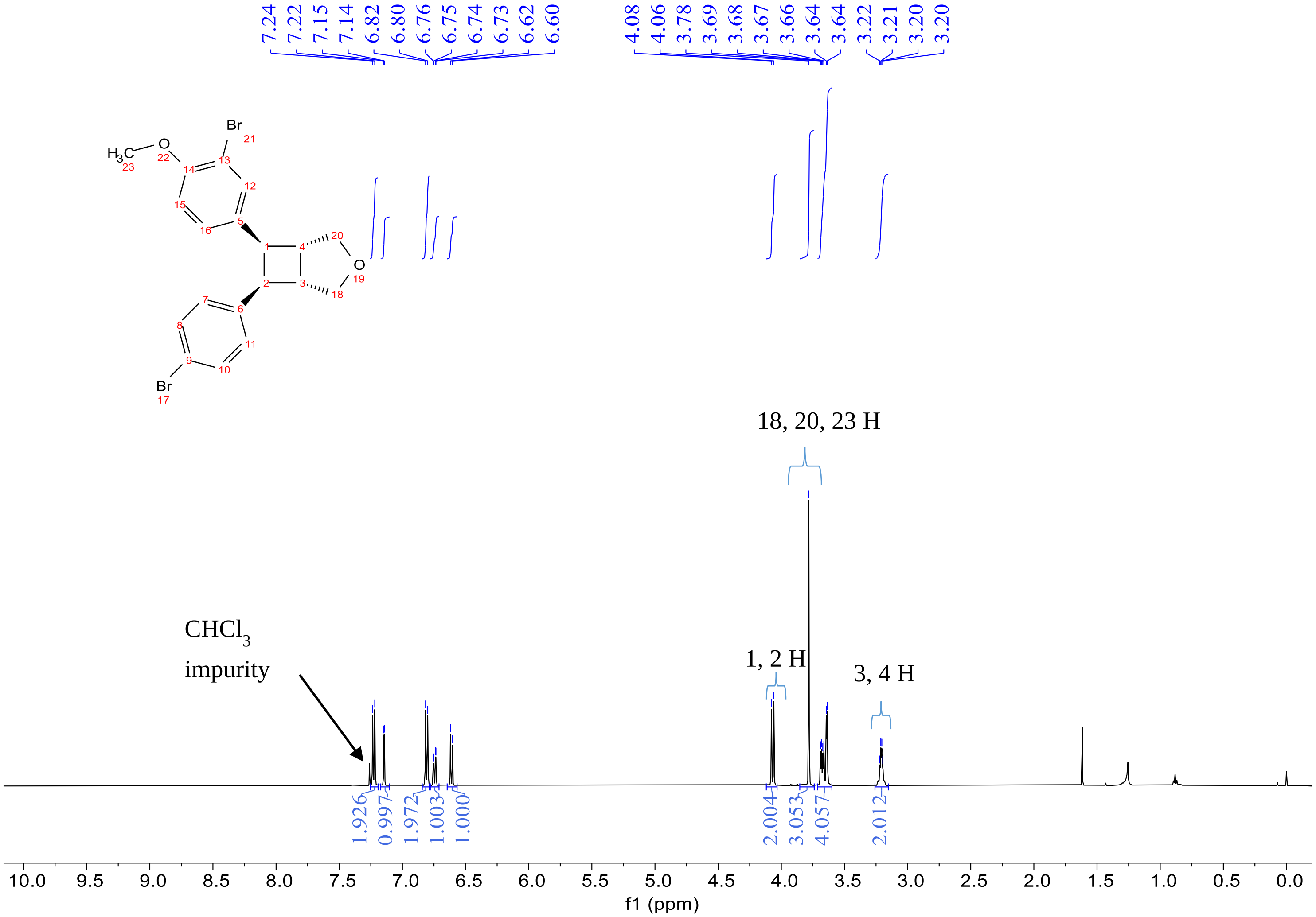

Supplementary Figure 115. ^{1}H NMR of **6l** (500 MHz, Chloroform-*d*)

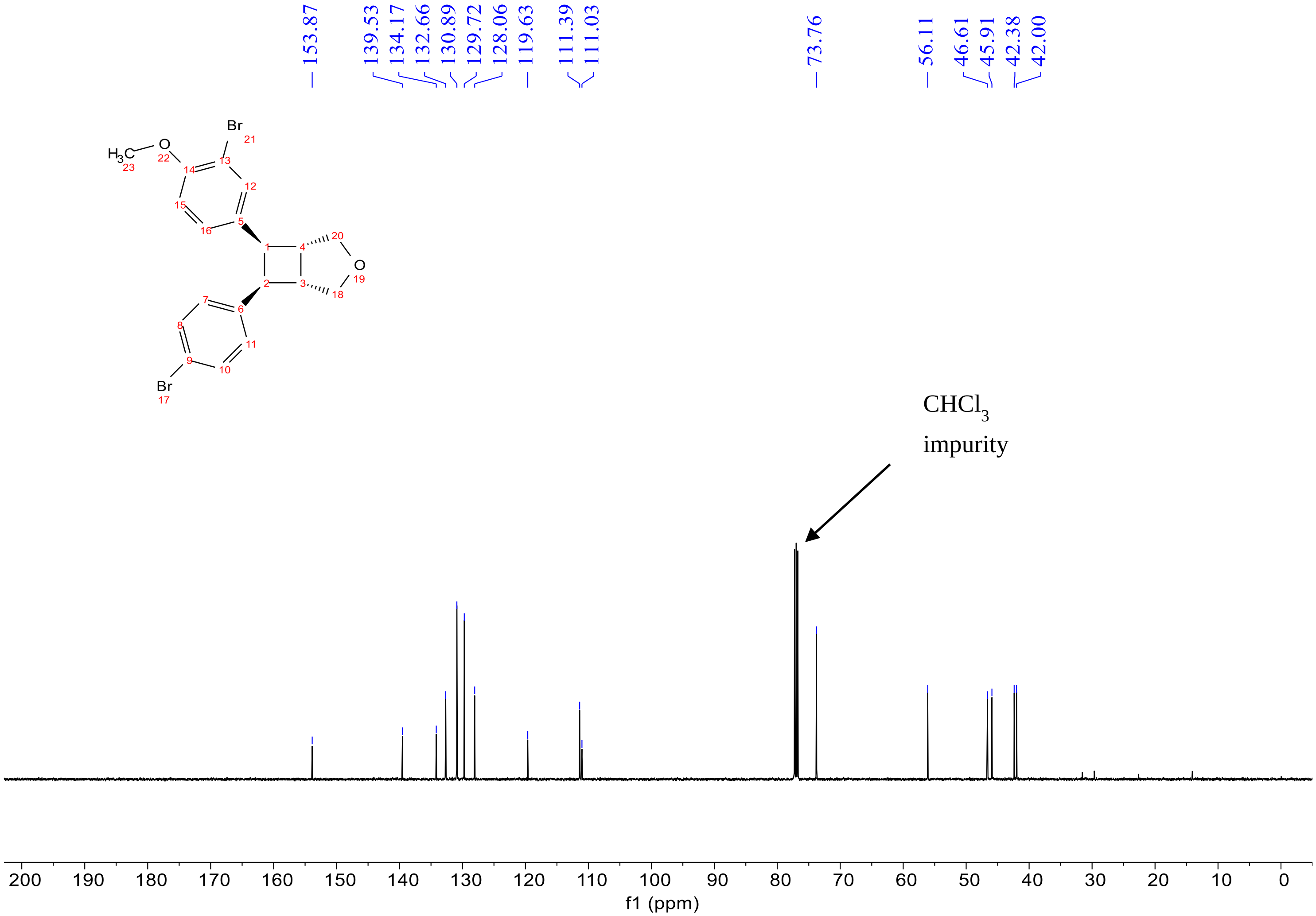

Supplementary Figure 116. ^{13}C NMR of **6l** (126 MHz, Chloroform-*d*)

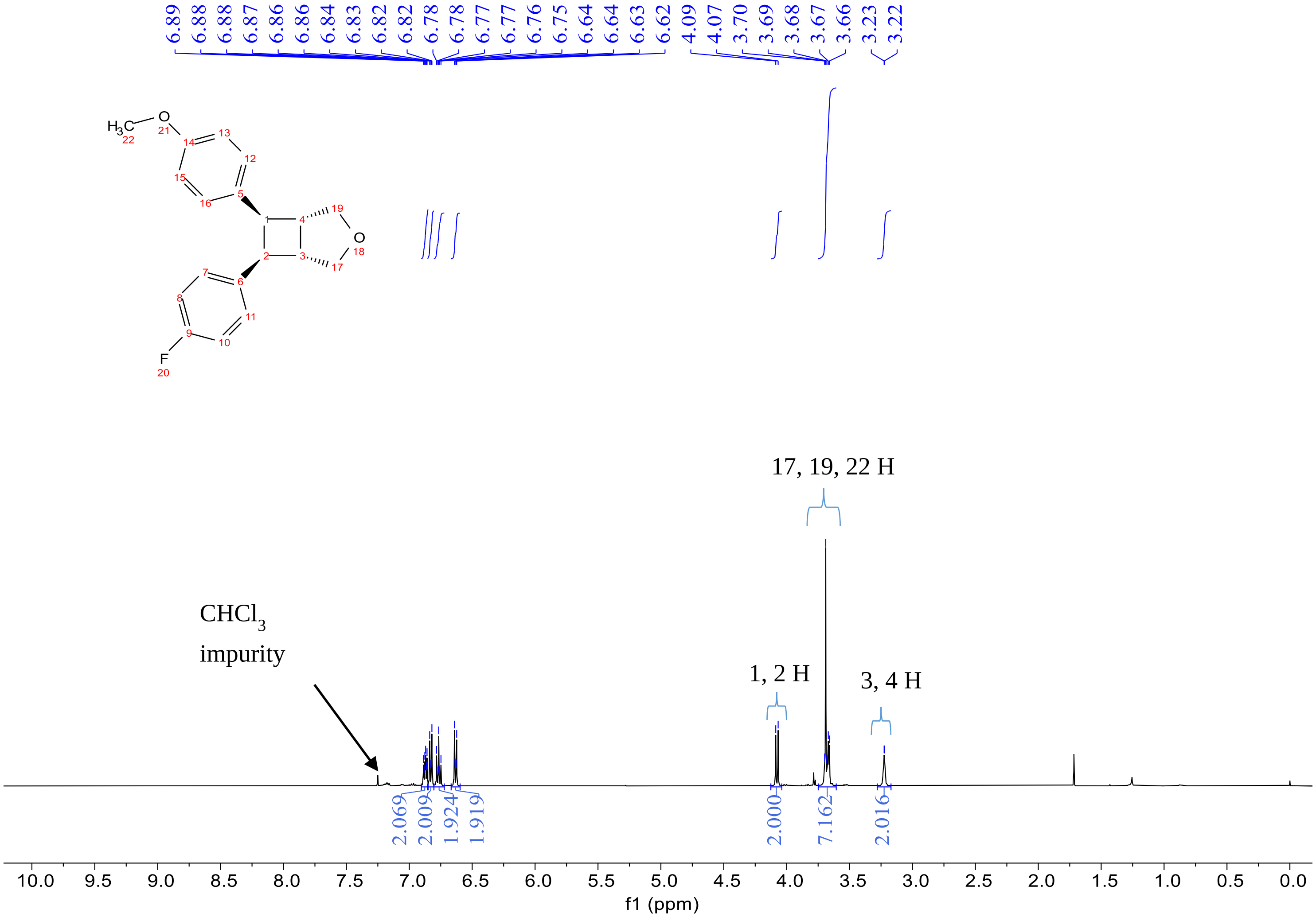

Supplementary Figure 117. ^{1}H NMR of **6m** (500 MHz, Chloroform-*d*)

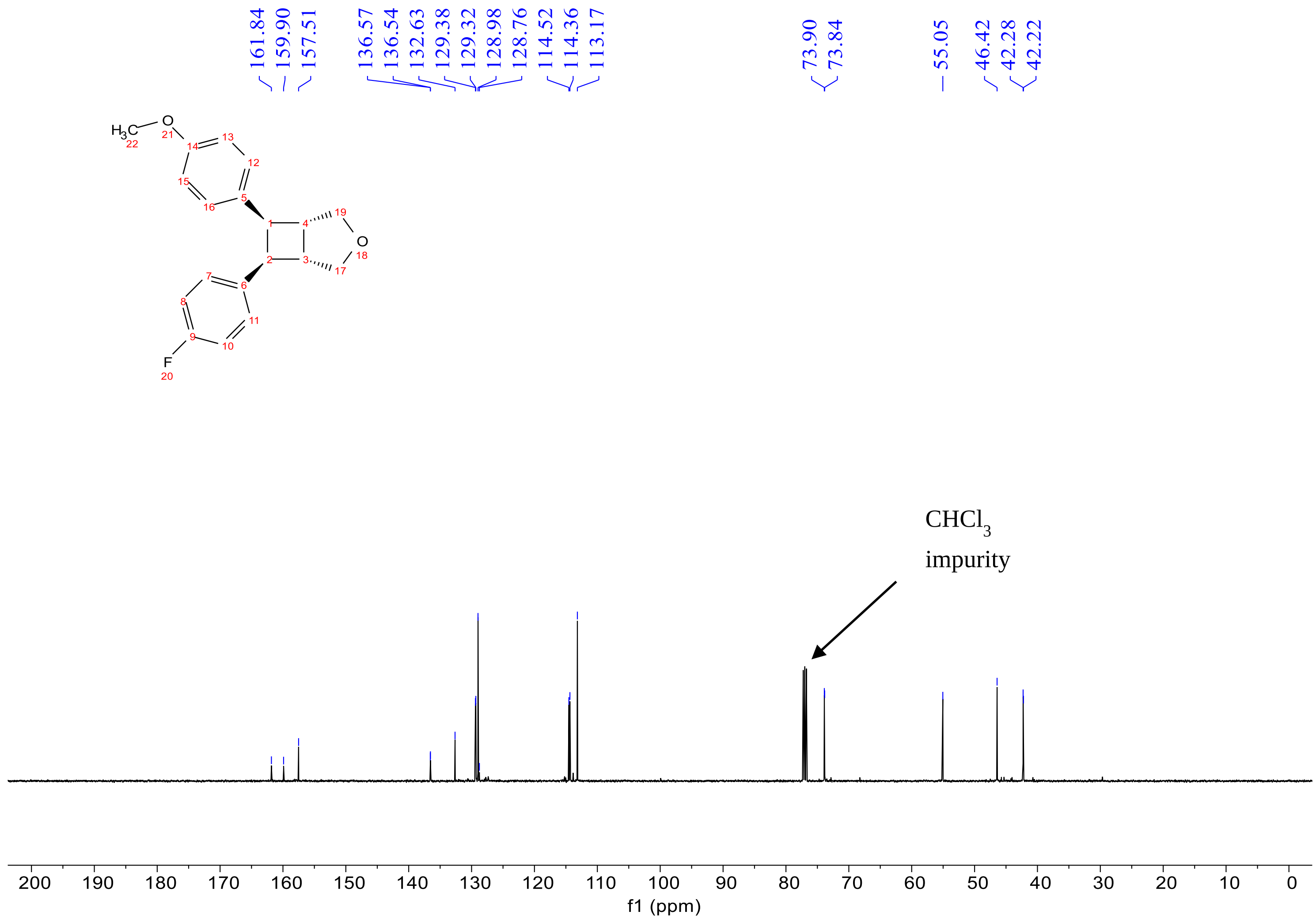

Supplementary Figure 118. ^{13}C NMR of **6m** (126 MHz, Chloroform-*d*)

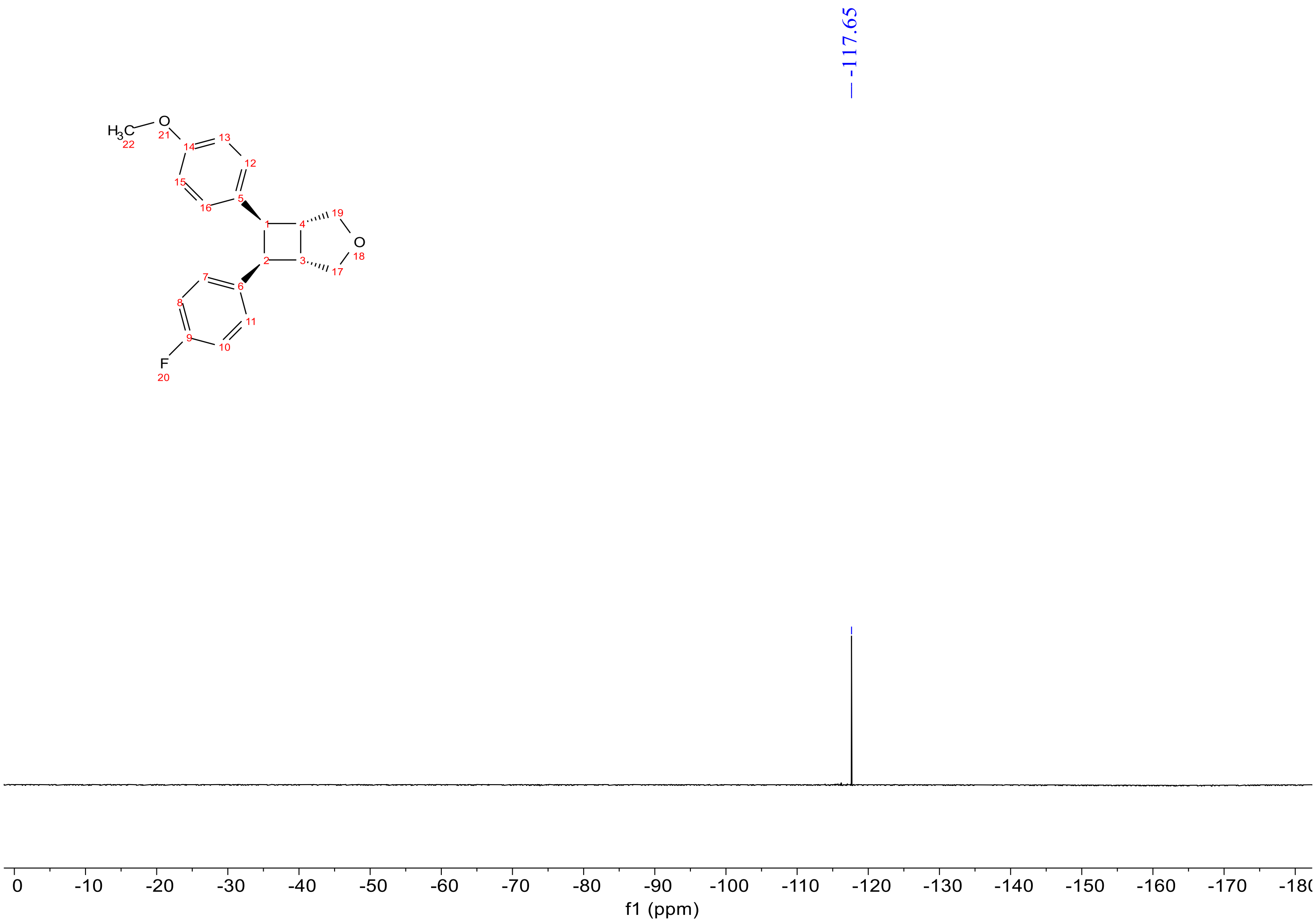

Supplementary Figure 119. ^{19}F NMR of **6m** (471 MHz, Chloroform-*d*)

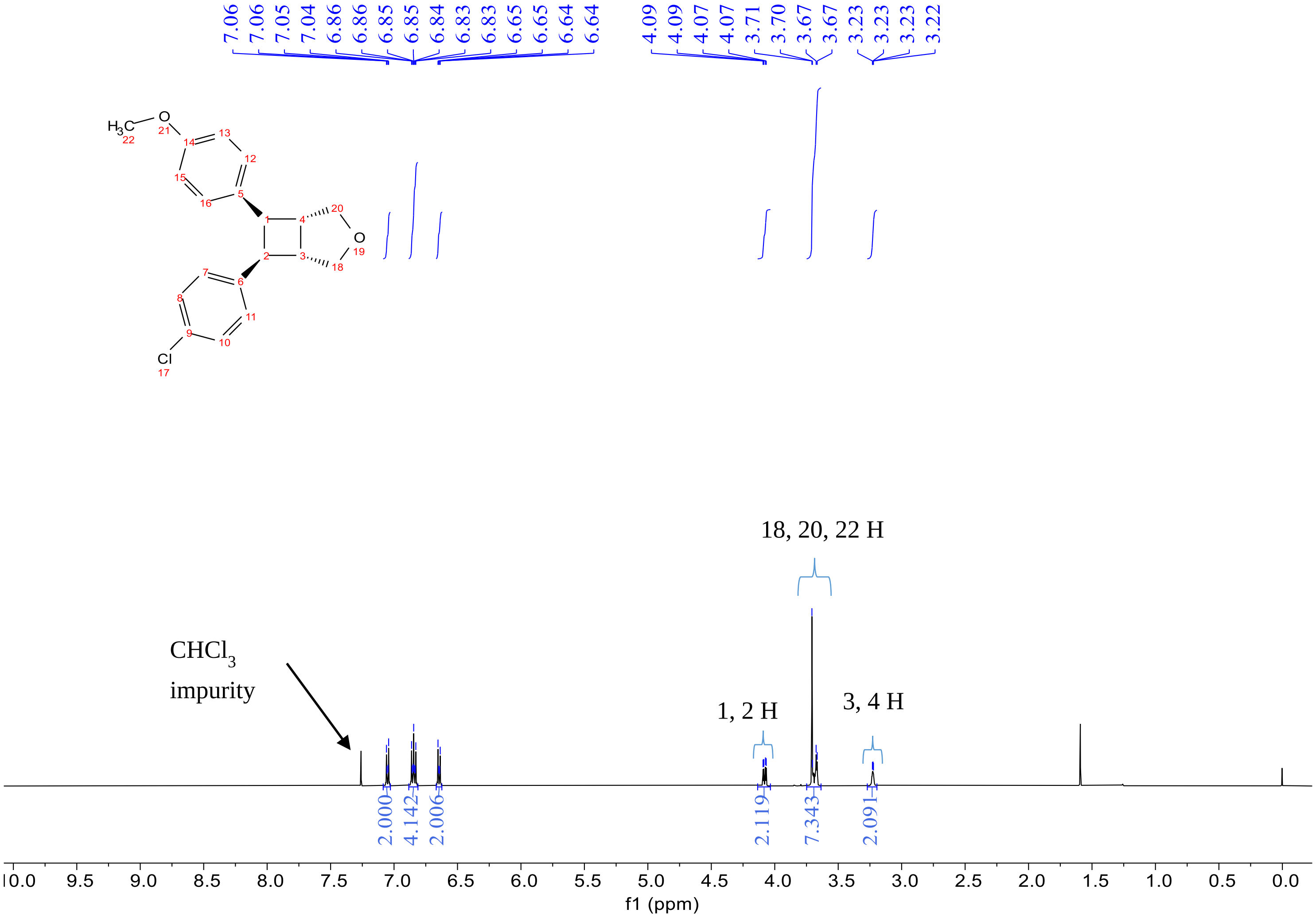

Supplementary Figure 120. ^{1}H NMR of **6n** (500 MHz, Chloroform-*d*)

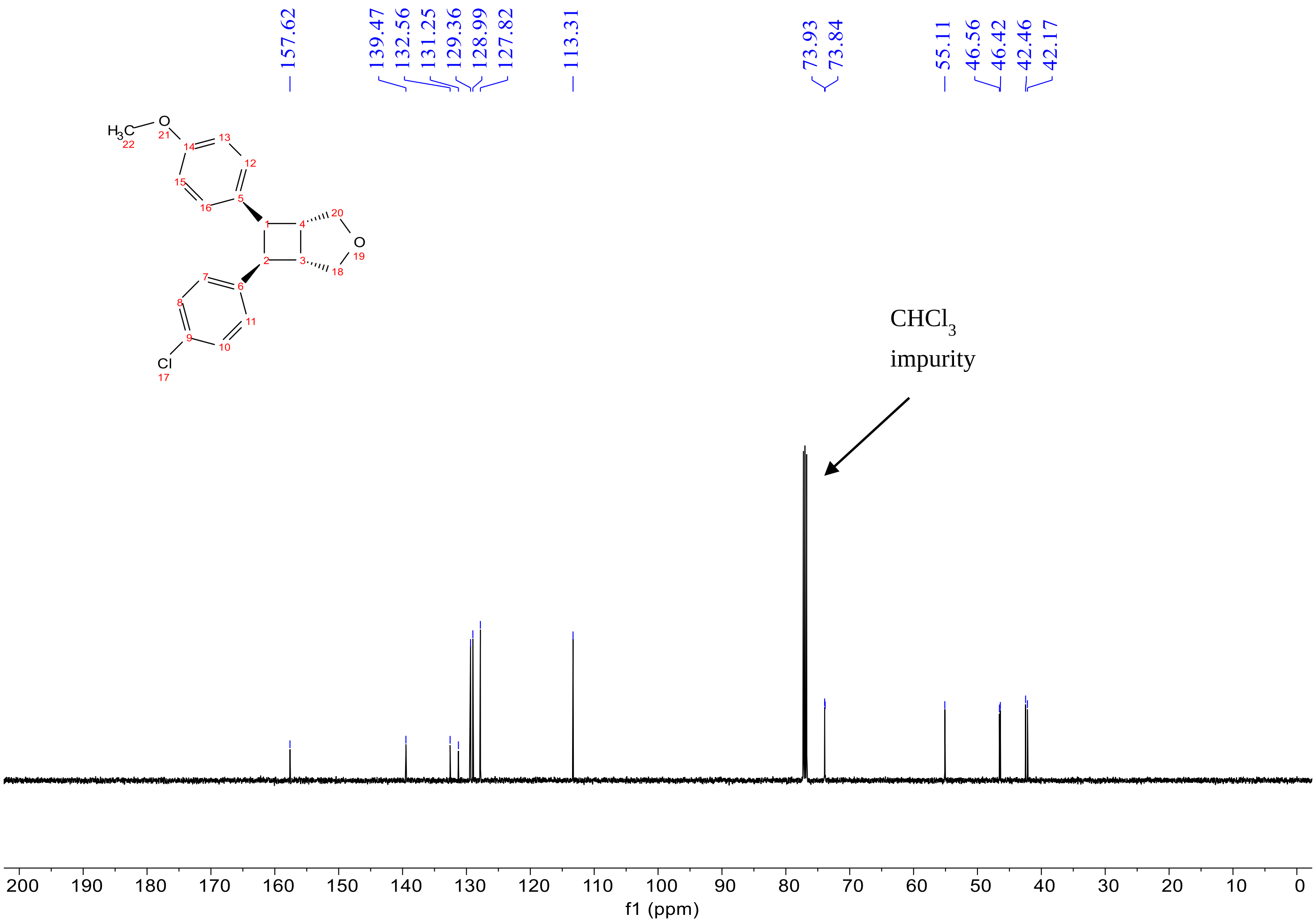

Supplementary Figure 121. ^{13}C NMR of **6n** (126 MHz, Chloroform-*d*)

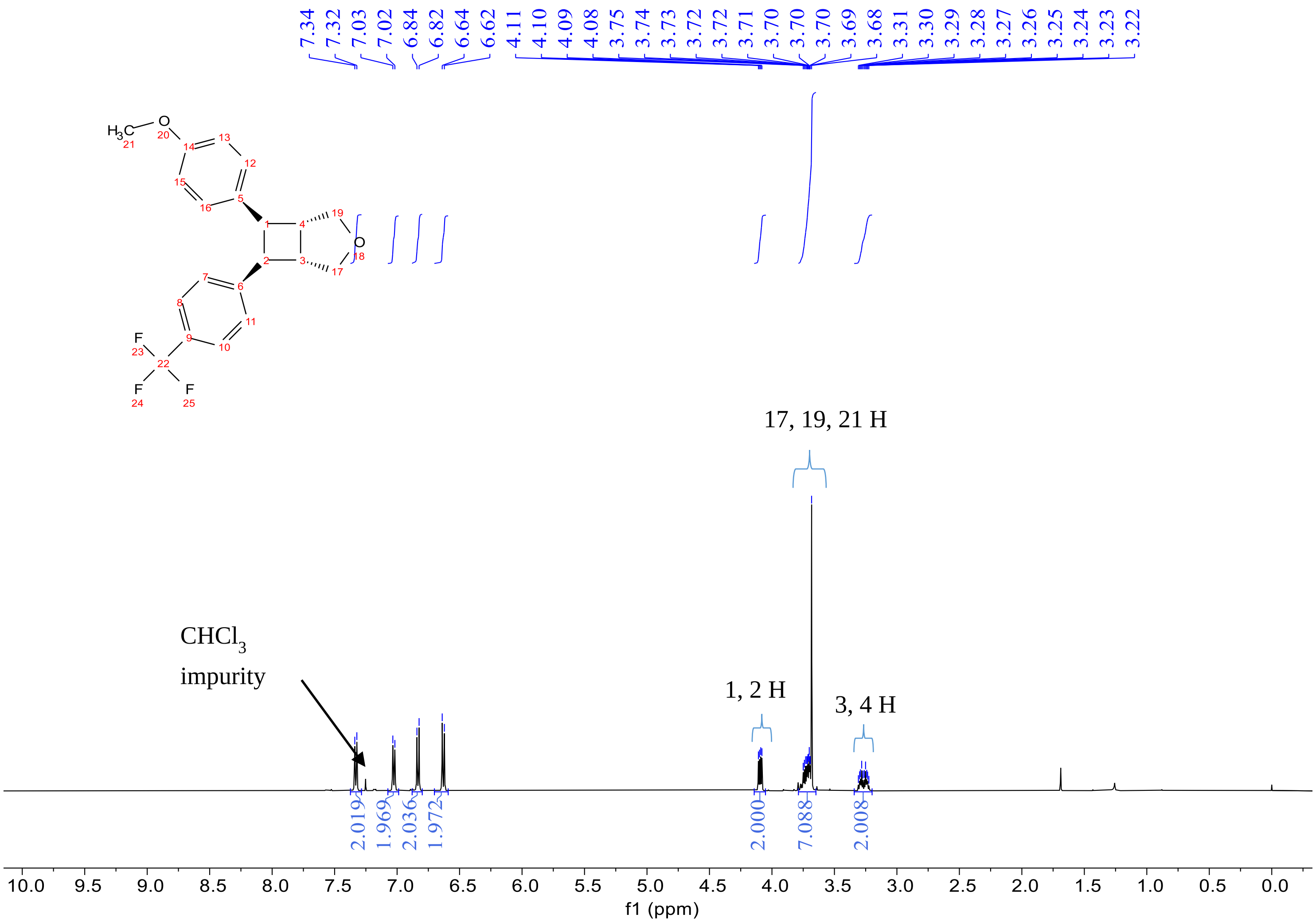

Supplementary Figure 122. ^{1}H NMR of **6o** (500 MHz, Chloroform-*d*)

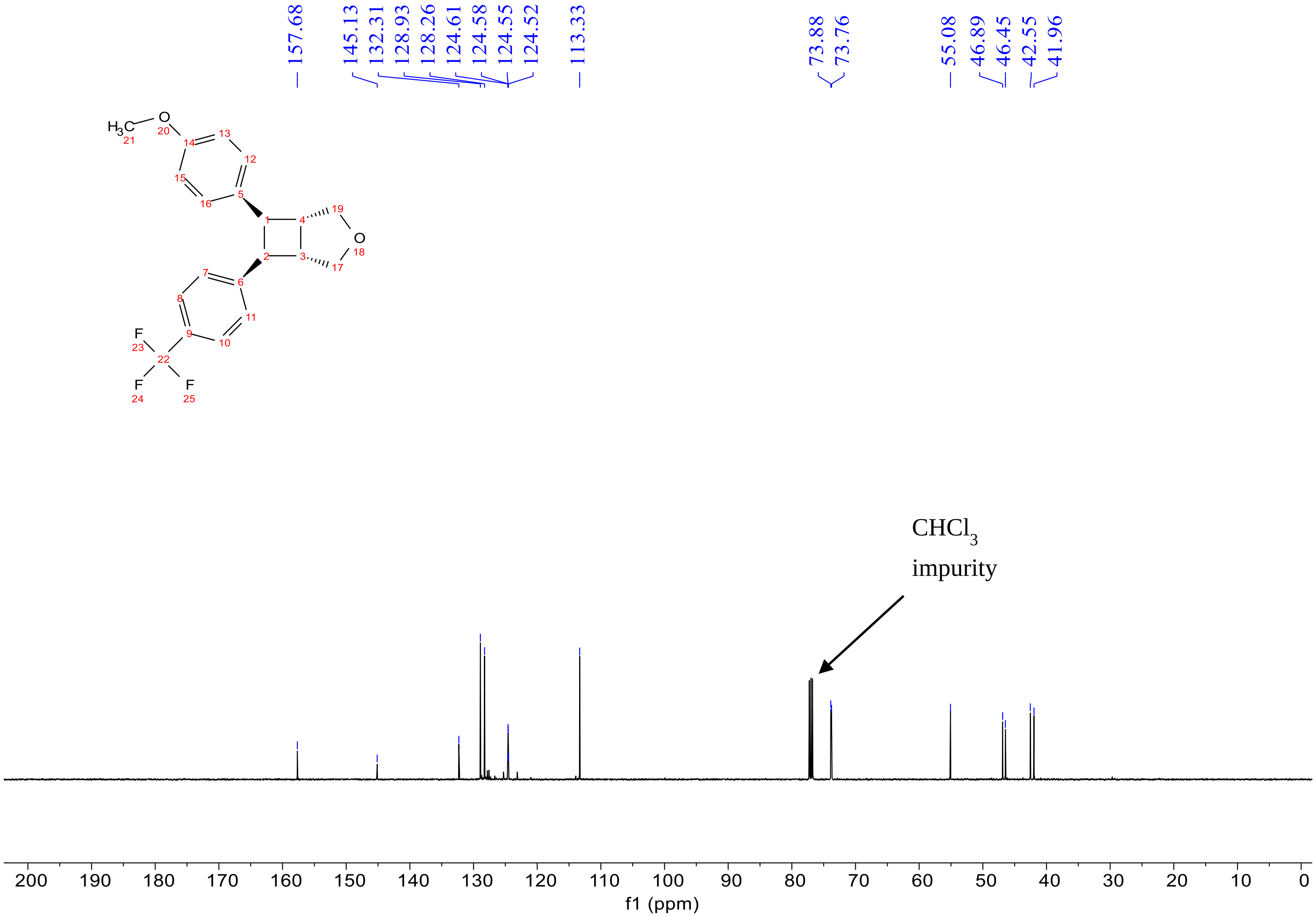

Supplementary Figure 123. ^{13}C NMR of **6o** (126 MHz, Chloroform-*d*)

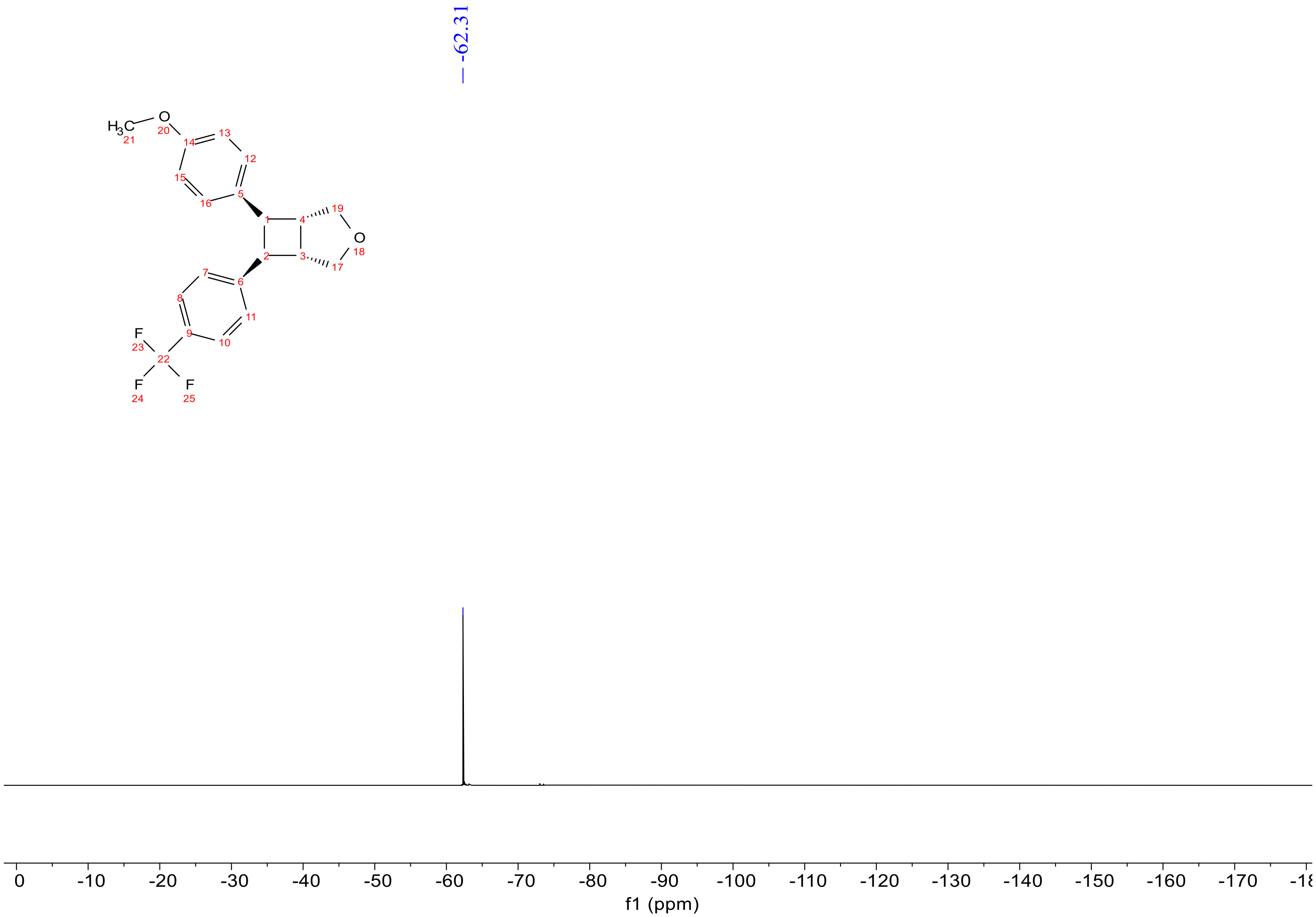

Supplementary Figure 124. ^{19}F NMR of **6o** (471 MHz, Chloroform-*d*)

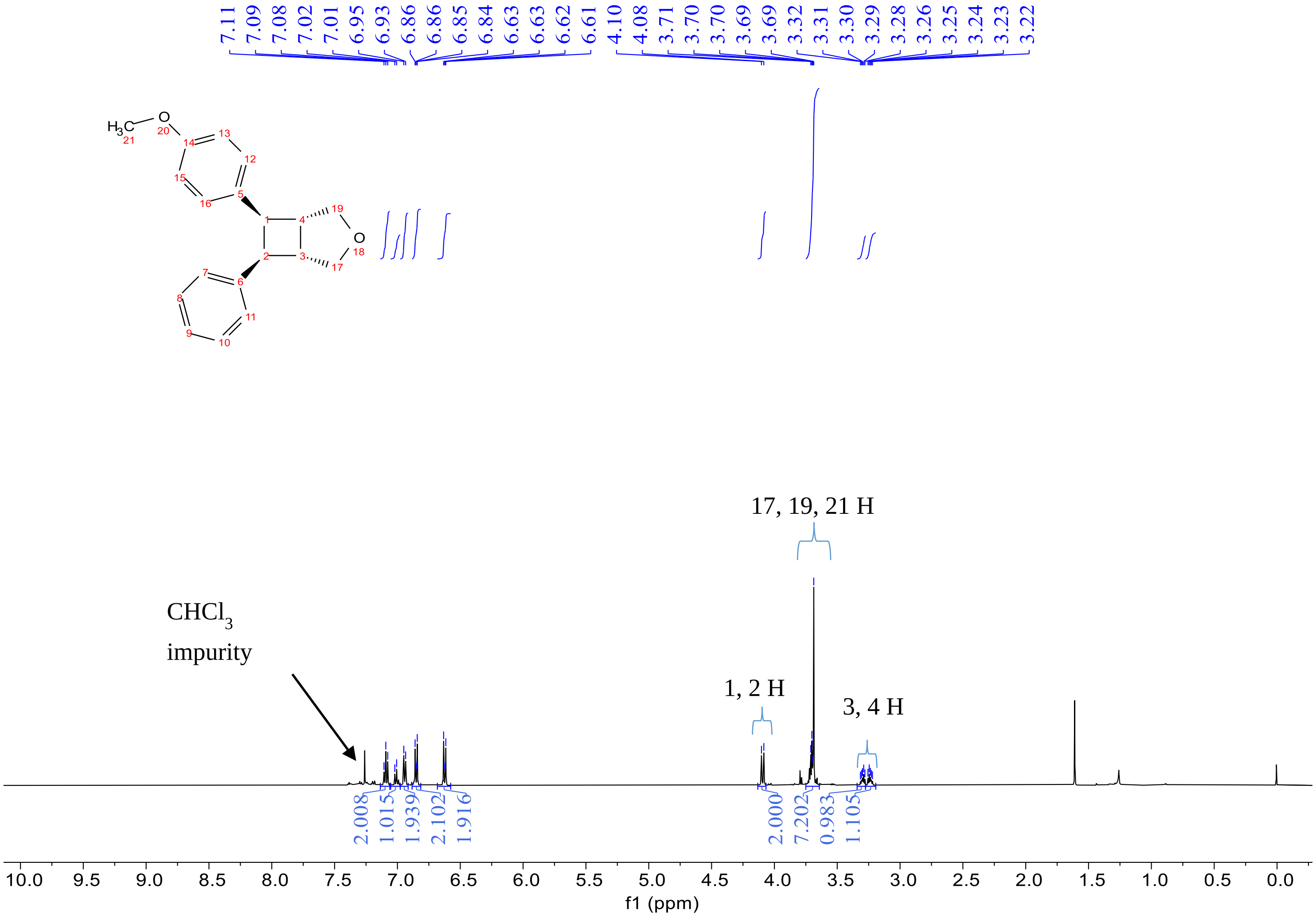

Supplementary Figure 125. ^{1}H NMR of **6p** (500 MHz, Chloroform-*d*)

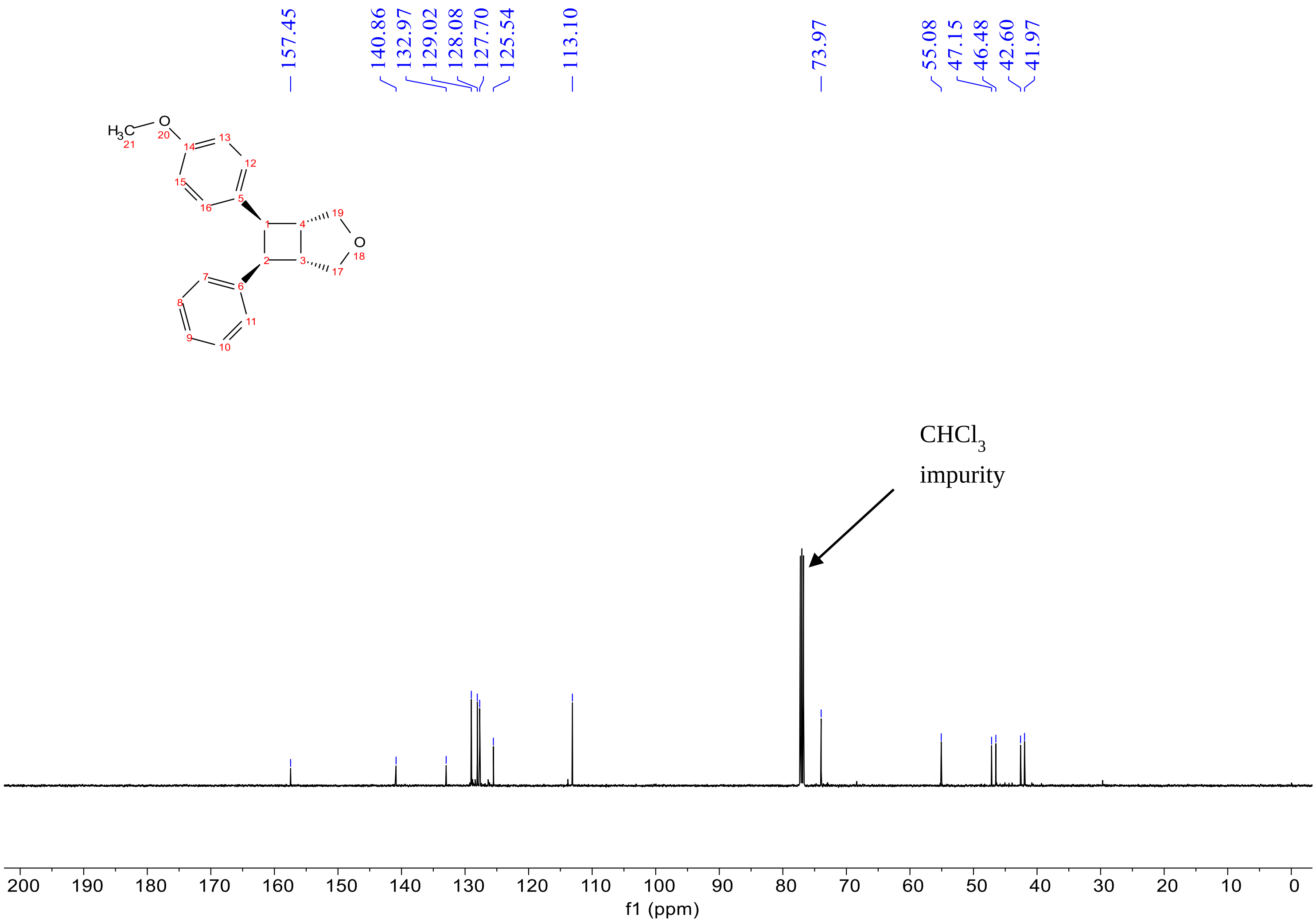

Supplementary Figure 126. ^{13}C NMR of **6p** (126 MHz, Chloroform-*d*)

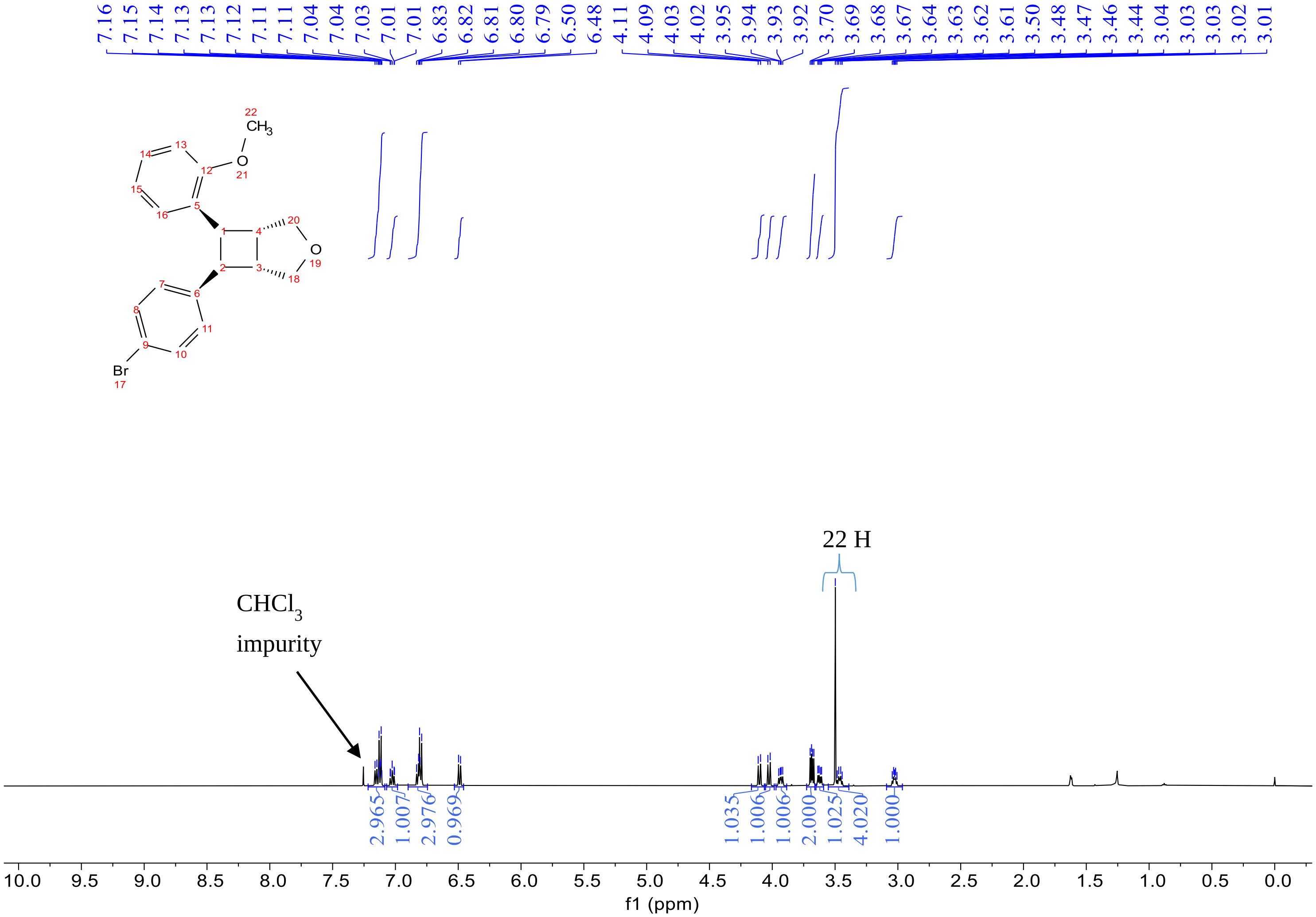

Supplementary Figure 127. ^{1}H NMR of **6q** (500 MHz, Chloroform-*d*)

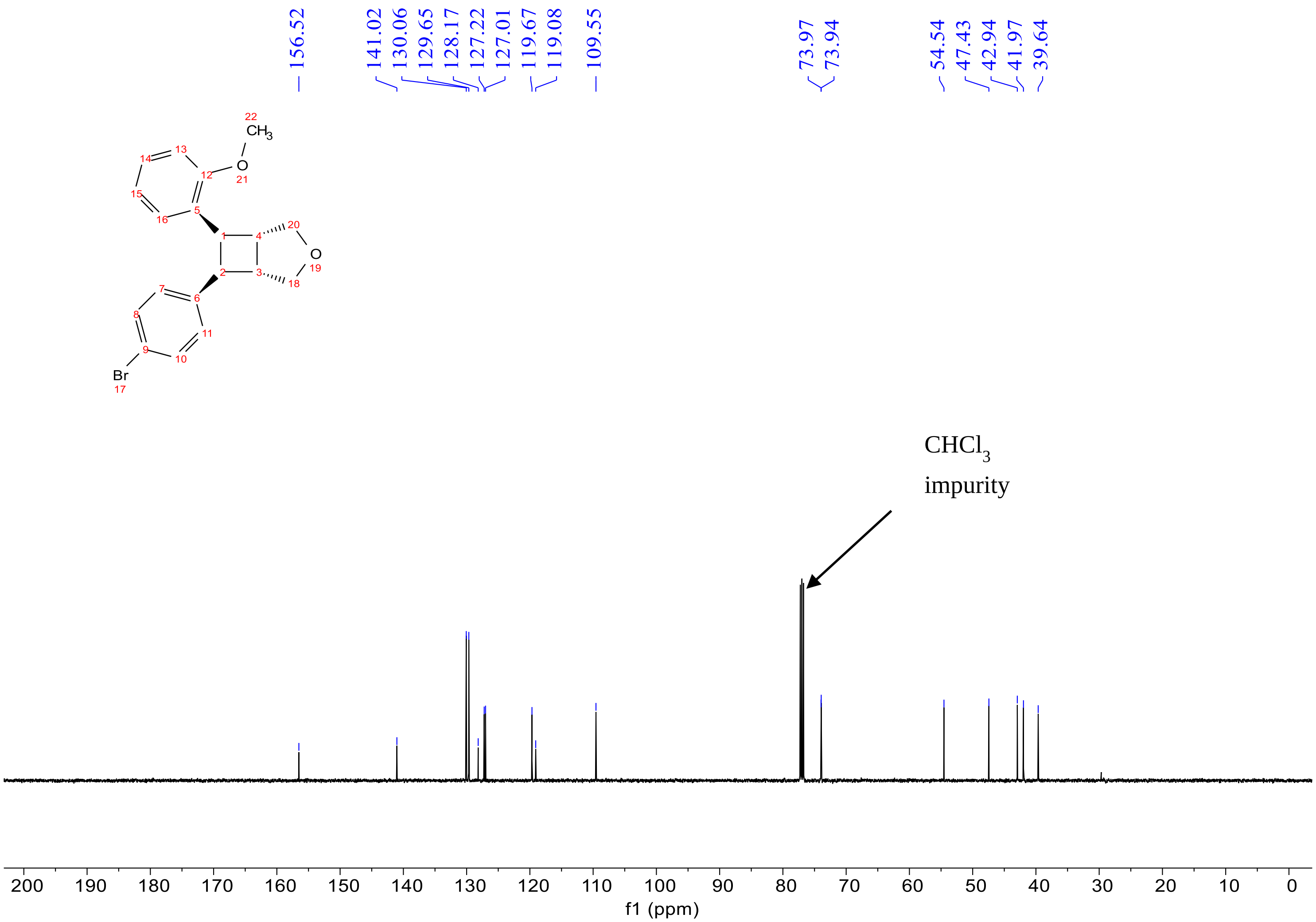

Supplementary Figure 128. ^{13}C NMR of **6q** (126 MHz, Chloroform-*d*)

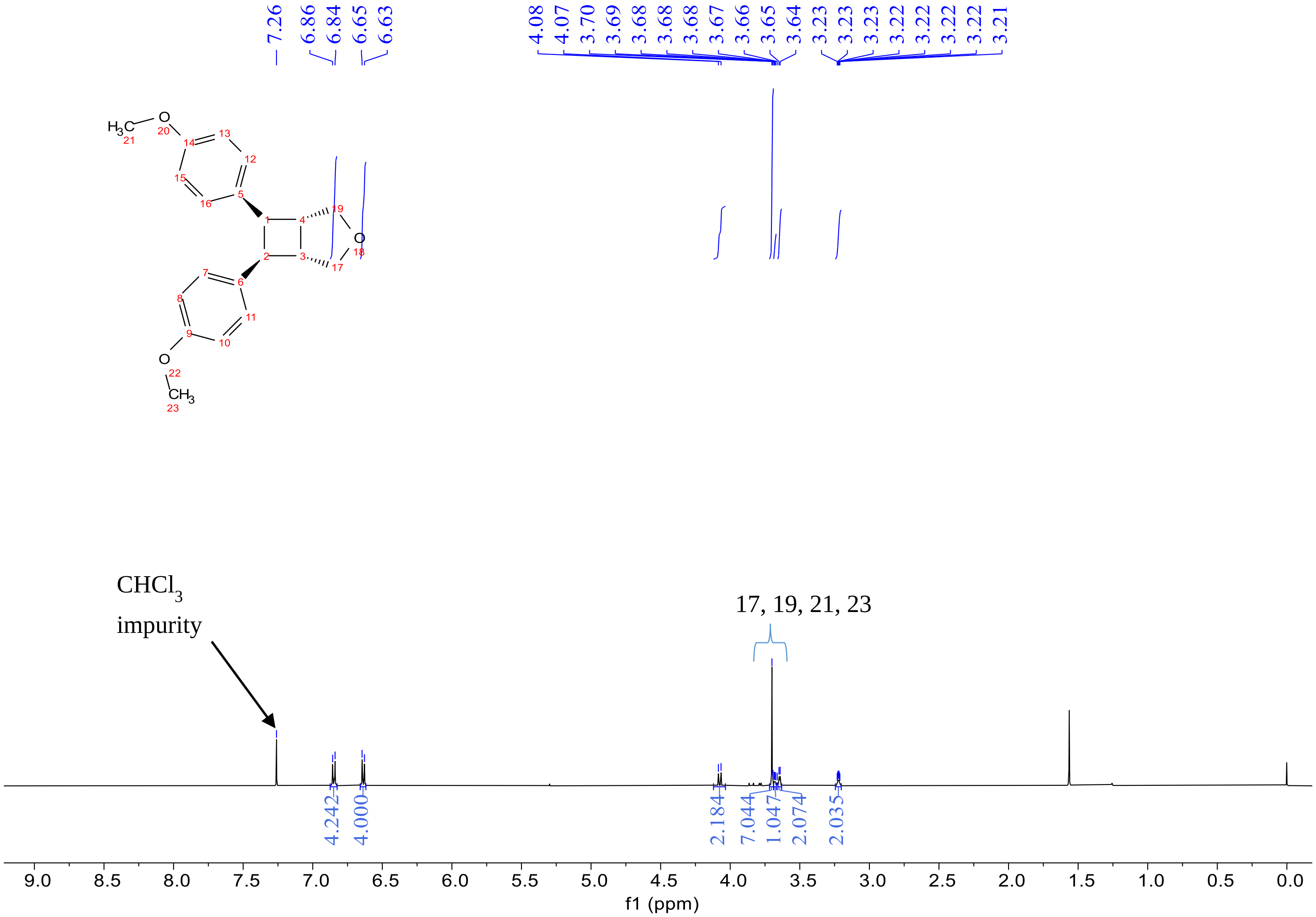

Supplementary Figure 129. ^{1}H NMR of **6r** (500 MHz, Chloroform-*d*)

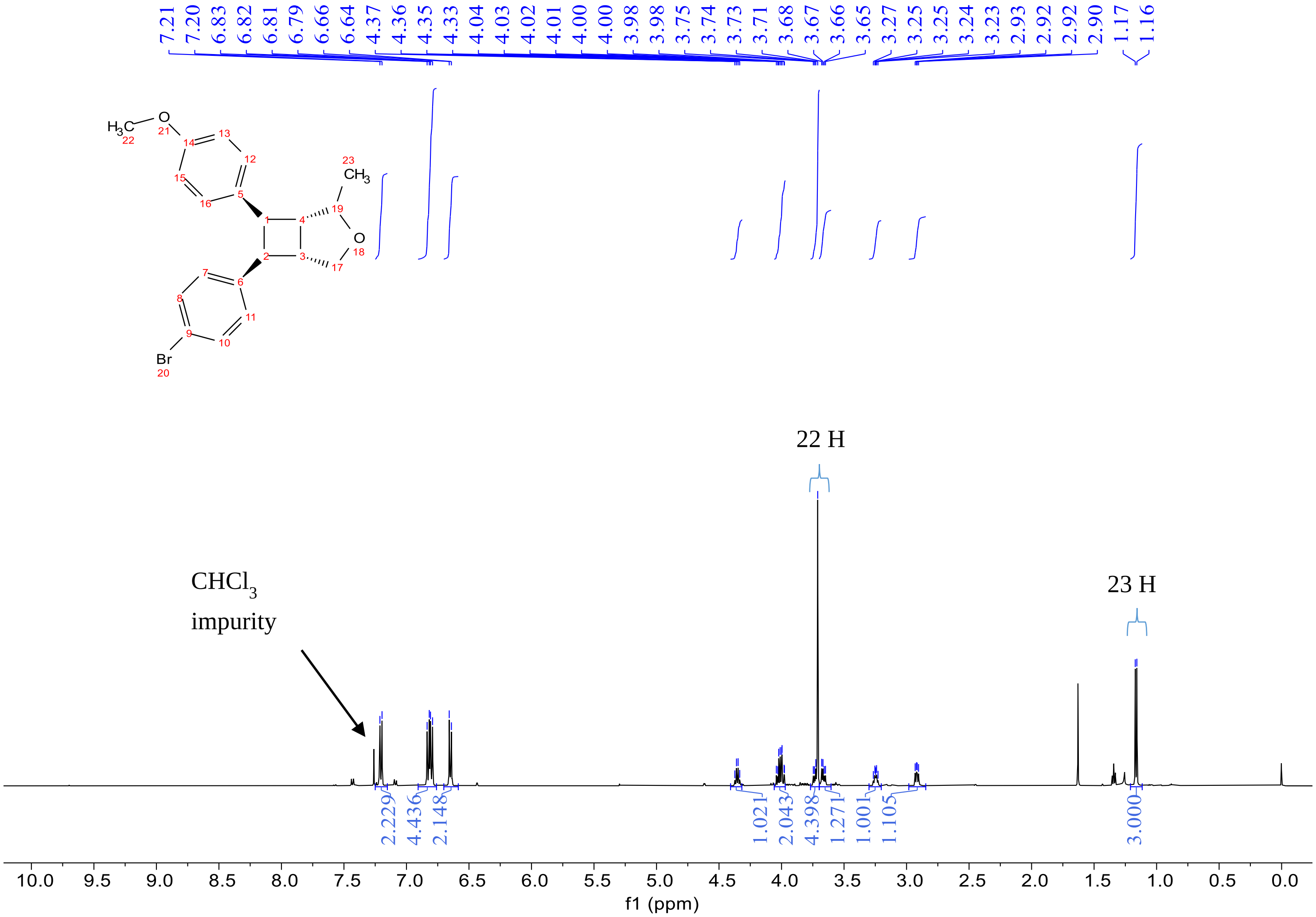

Supplementary Figure 130. ^{1}H NMR of **6s** (500 MHz, Chloroform-*d*)

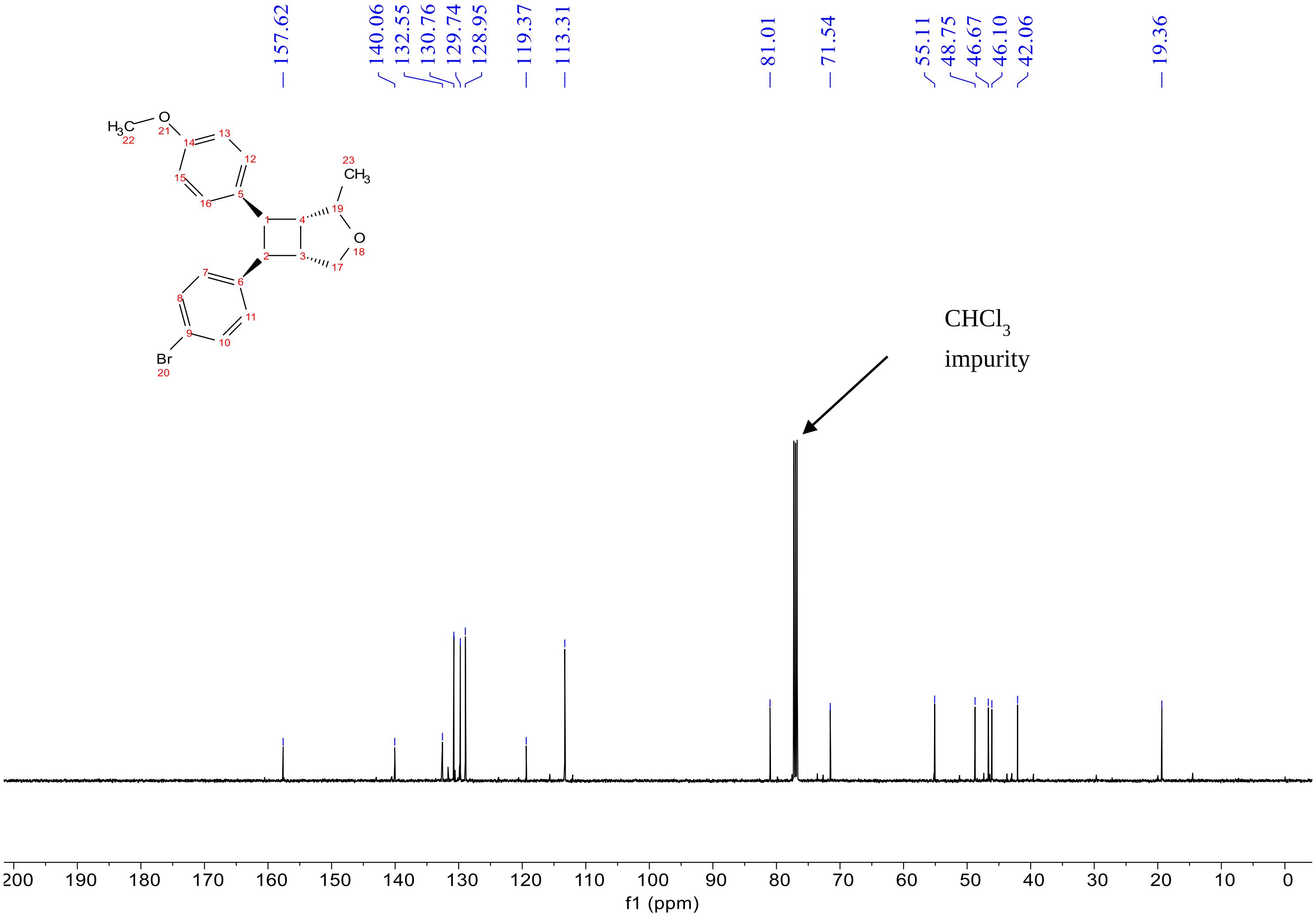

Supplementary Figure 131. ^{13}C NMR of **6s** (126 MHz, Chloroform-*d*)

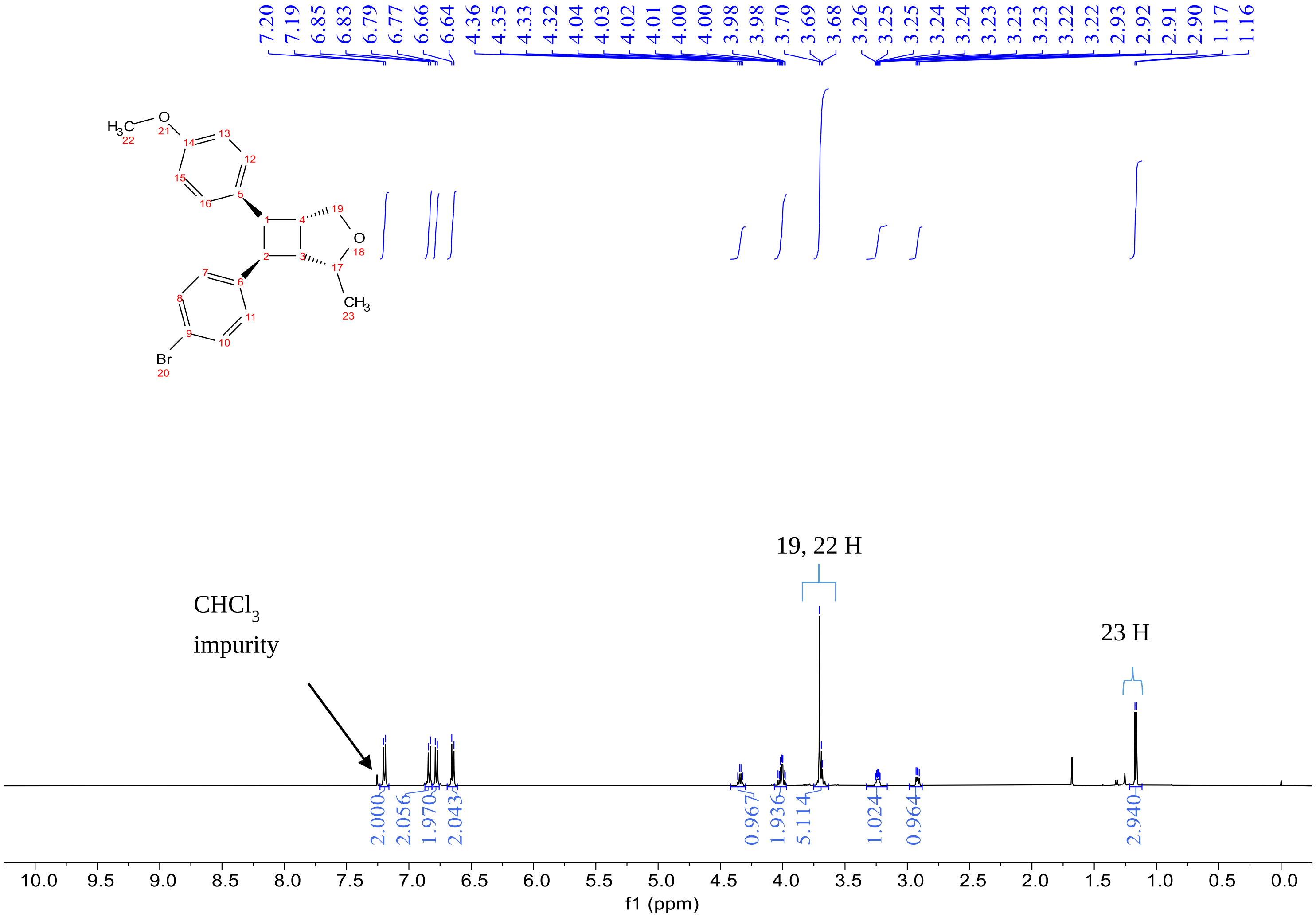

Supplementary Figure 132. ^{1}H NMR of **6t** (500 MHz, Chloroform-*d*)

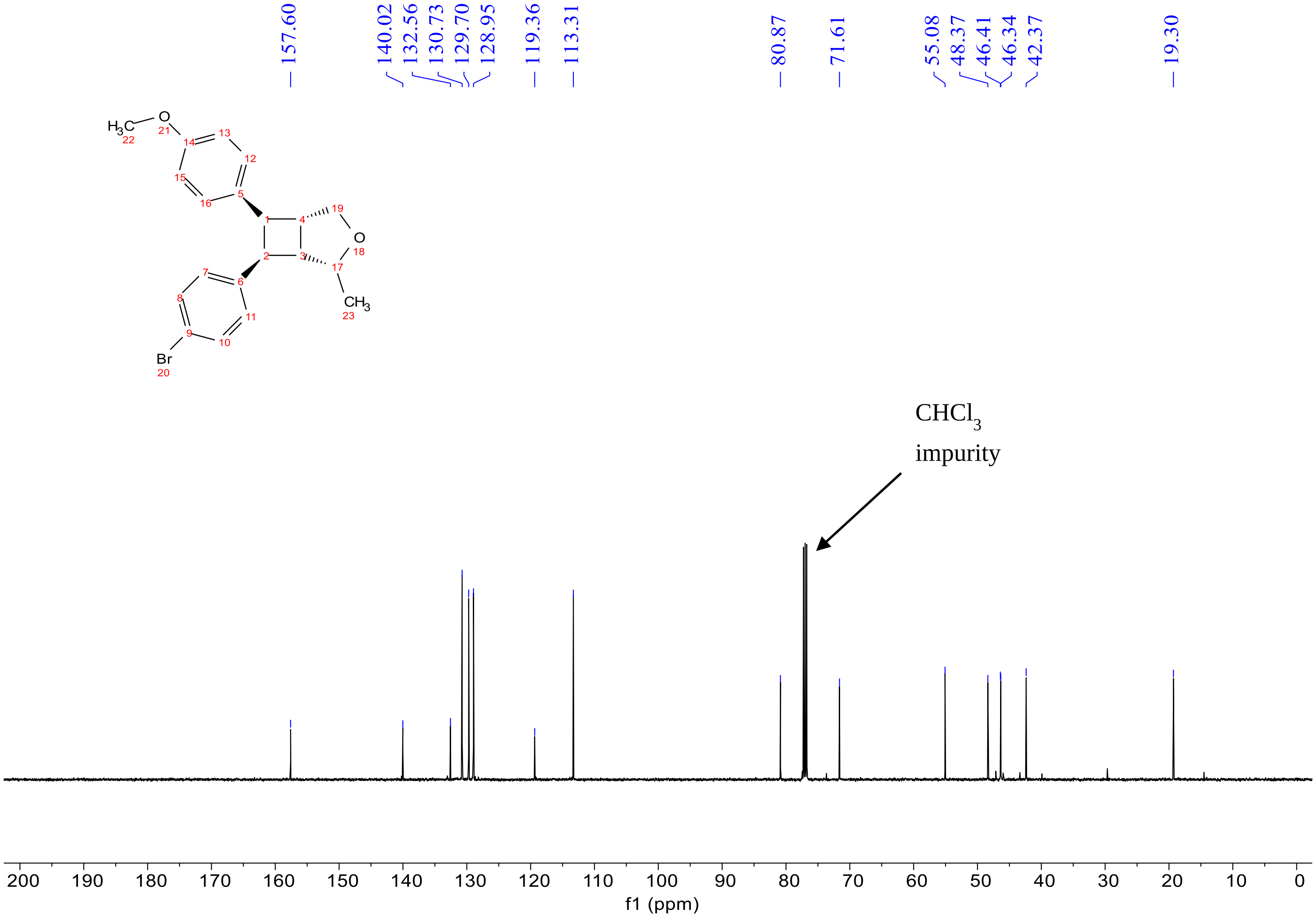

Supplementary Figure 133. ^{13}C NMR of **6t** (126 MHz, Chloroform-*d*)

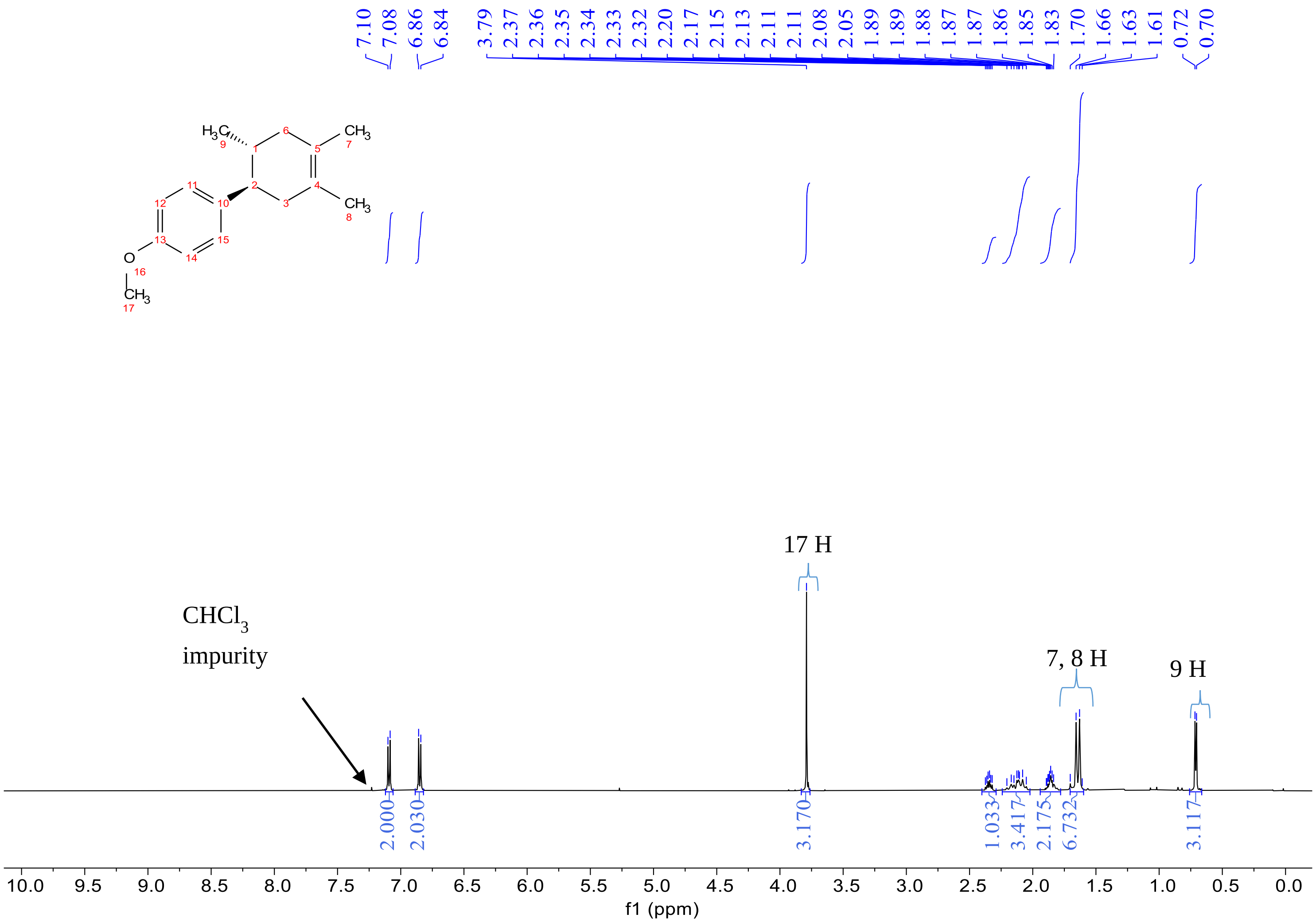

Supplementary Figure 134. ^{1}H NMR of **8a** (500 MHz, Chloroform-*d*)

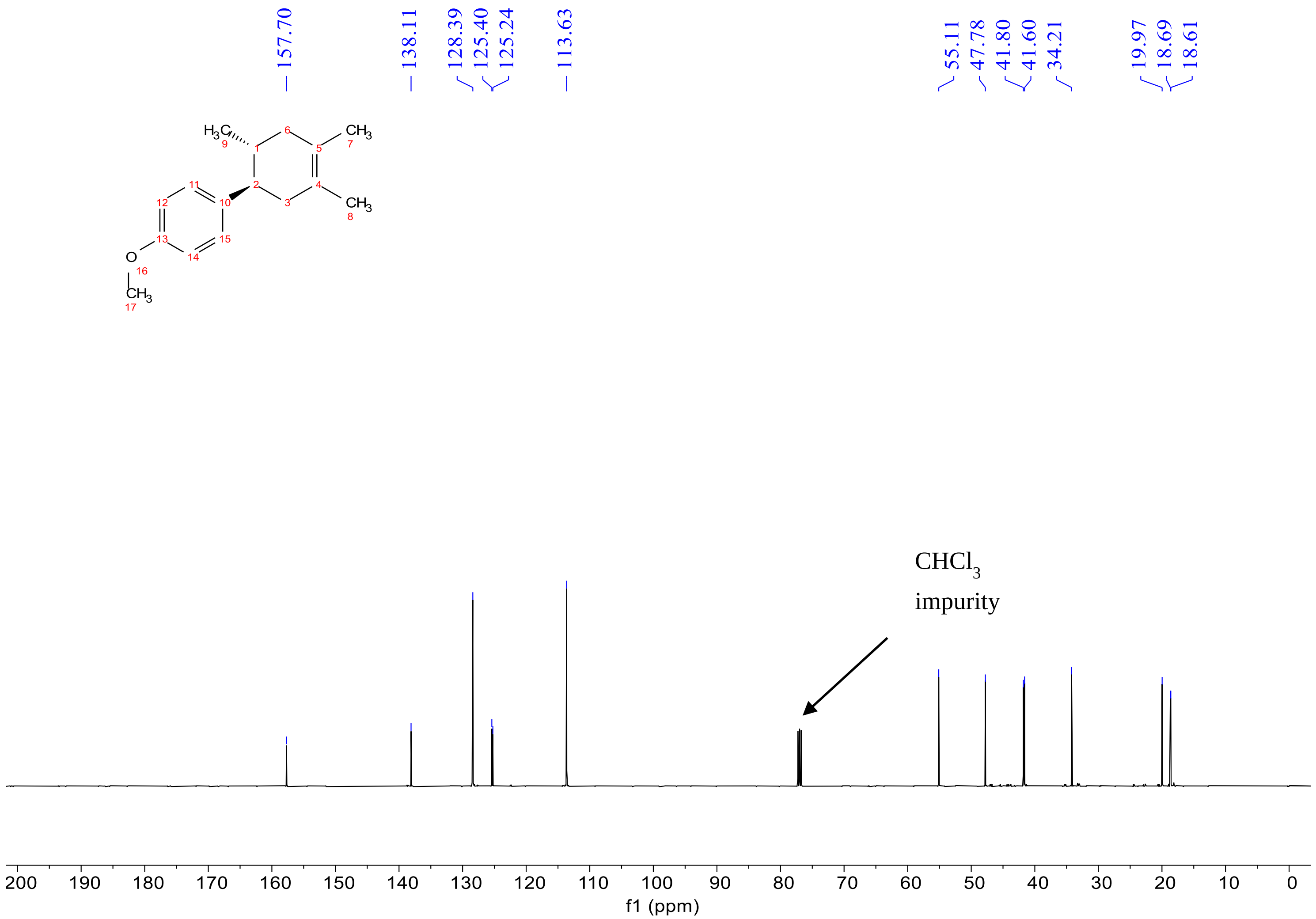

Supplementary Figure 135. ^{13}C NMR of **8a** (126 MHz, Chloroform-*d*)

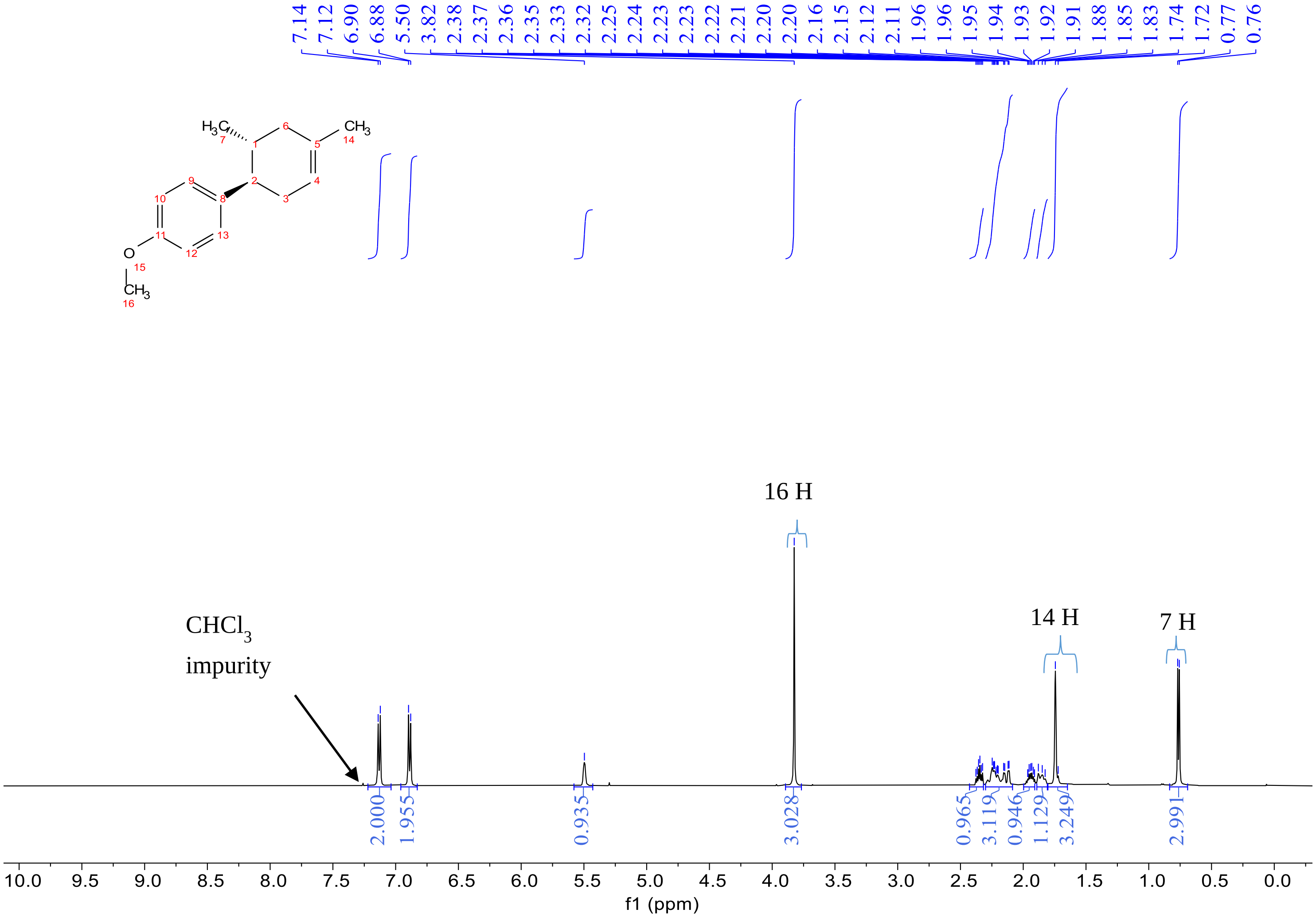

Supplementary Figure 136. ^{1}H NMR of **8b** (500 MHz, Chloroform-*d*)

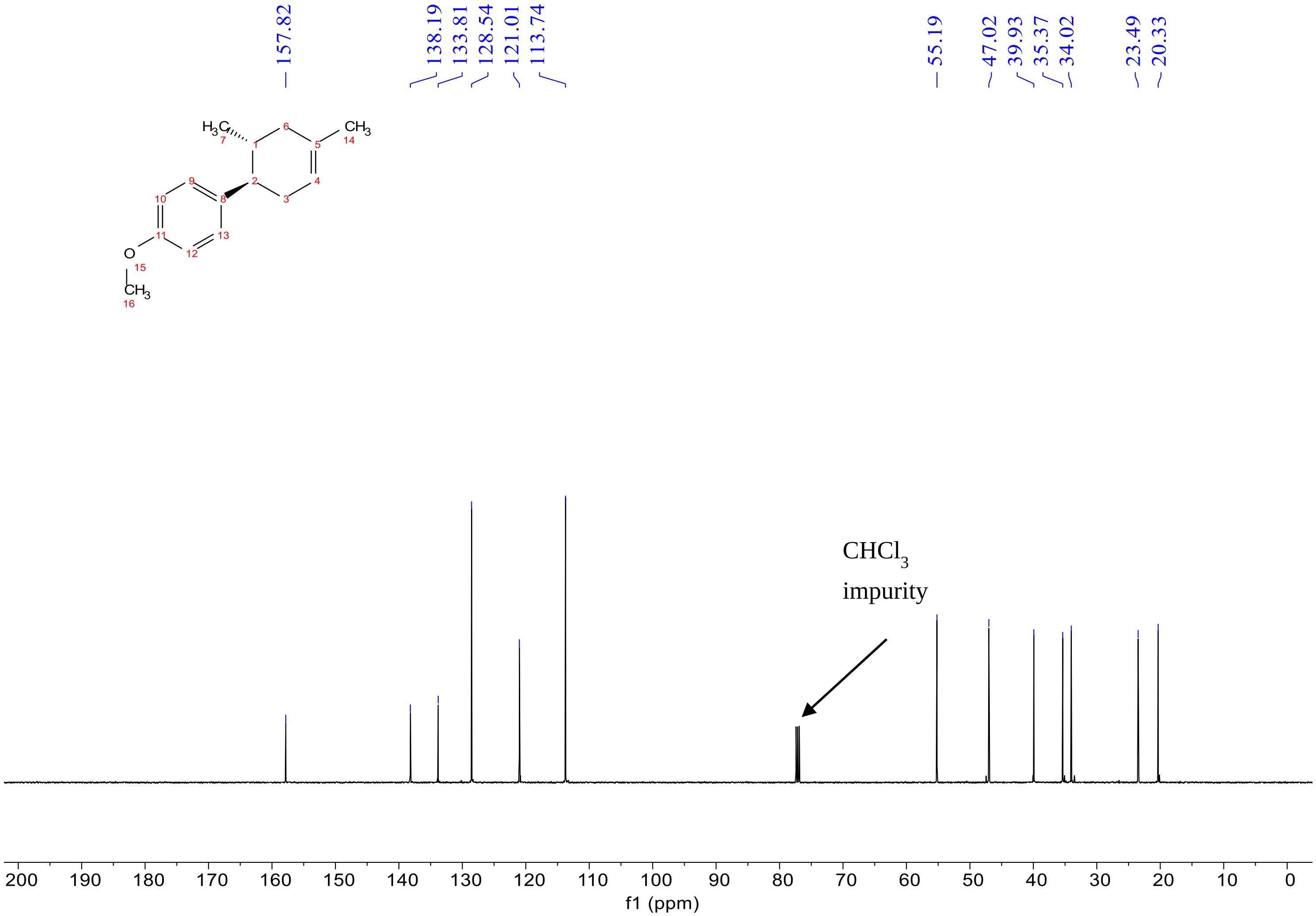

Supplementary Figure 137. ^{13}C NMR of **8b** (126 MHz, Chloroform-*d*)

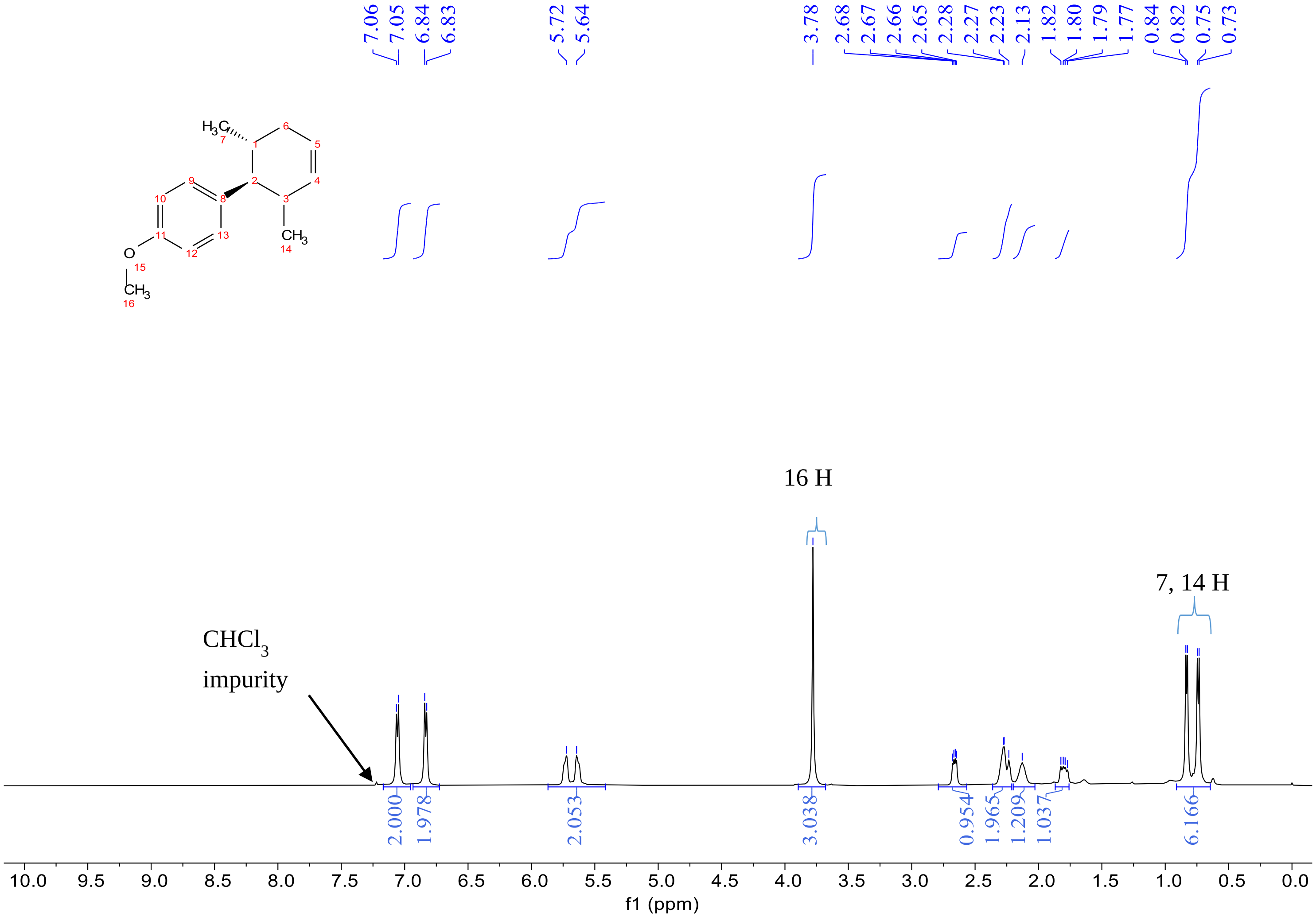

Supplementary Figure 138. ^{1}H NMR of **8c** (500 MHz, Chloroform-*d*)

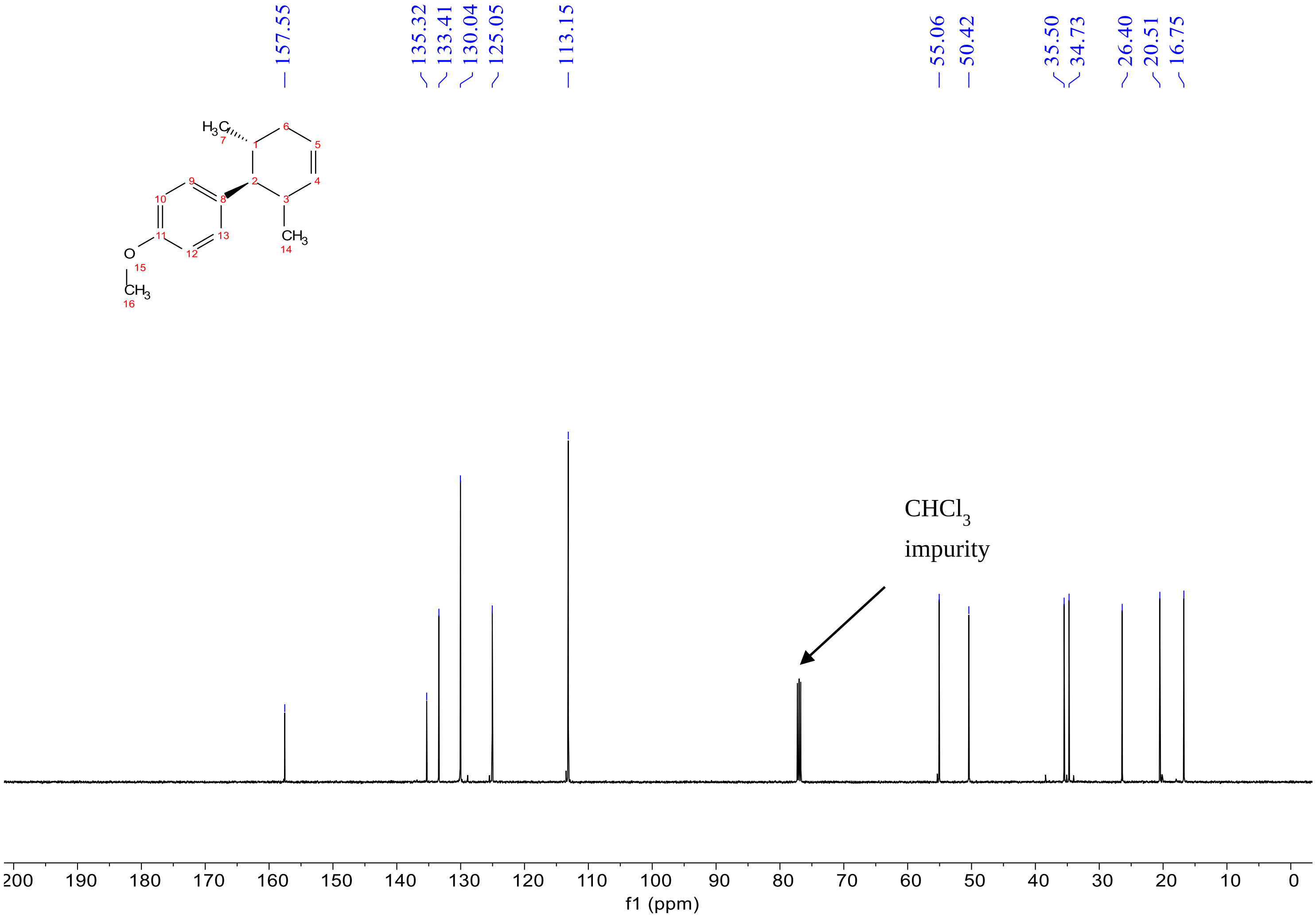

Supplementary Figure 139. ^{13}C NMR of **8c** (126 MHz, Chloroform-*d*)

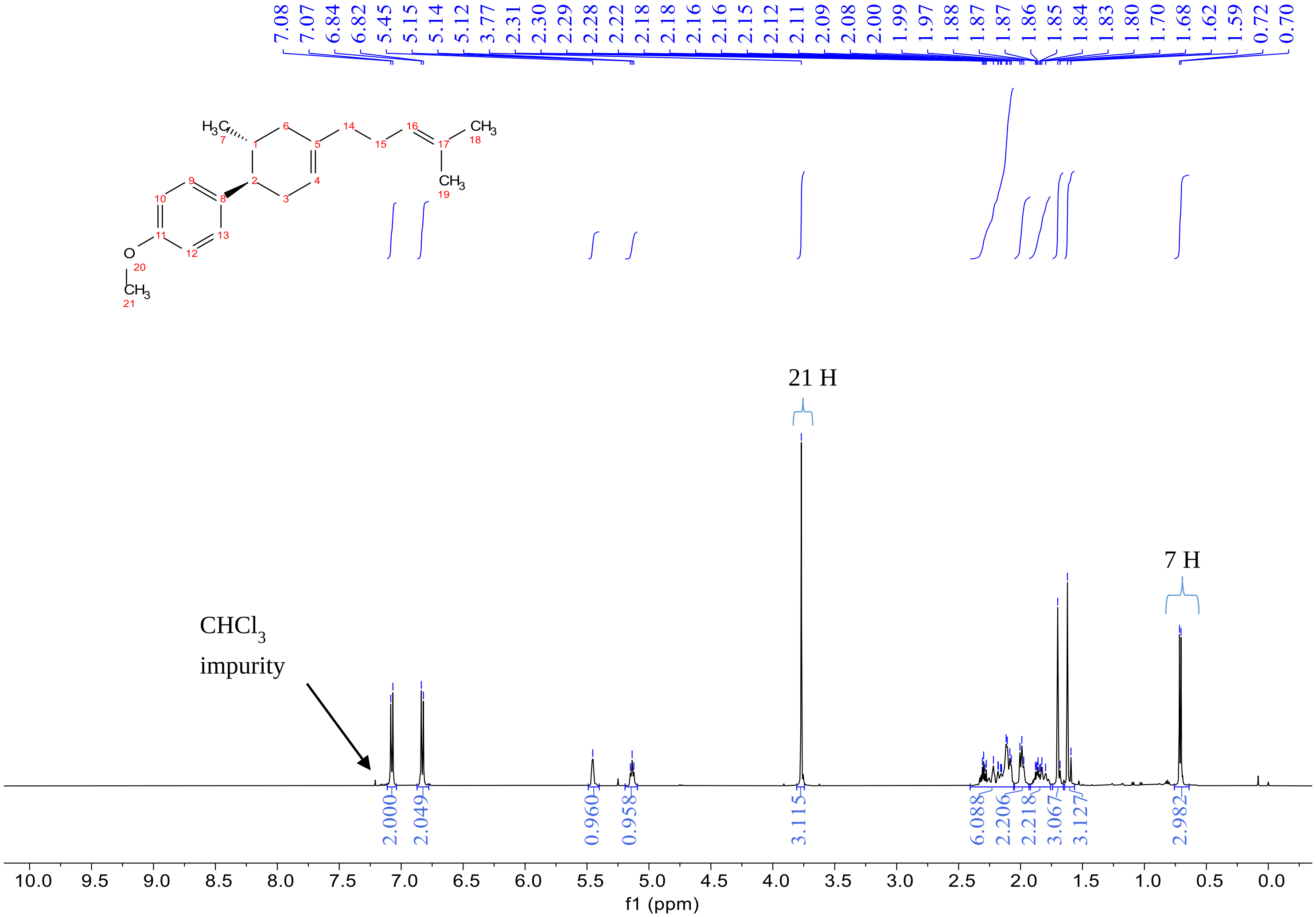

Supplementary Figure 140. ^{1}H NMR of **8d** (500 MHz, Chloroform-*d*)

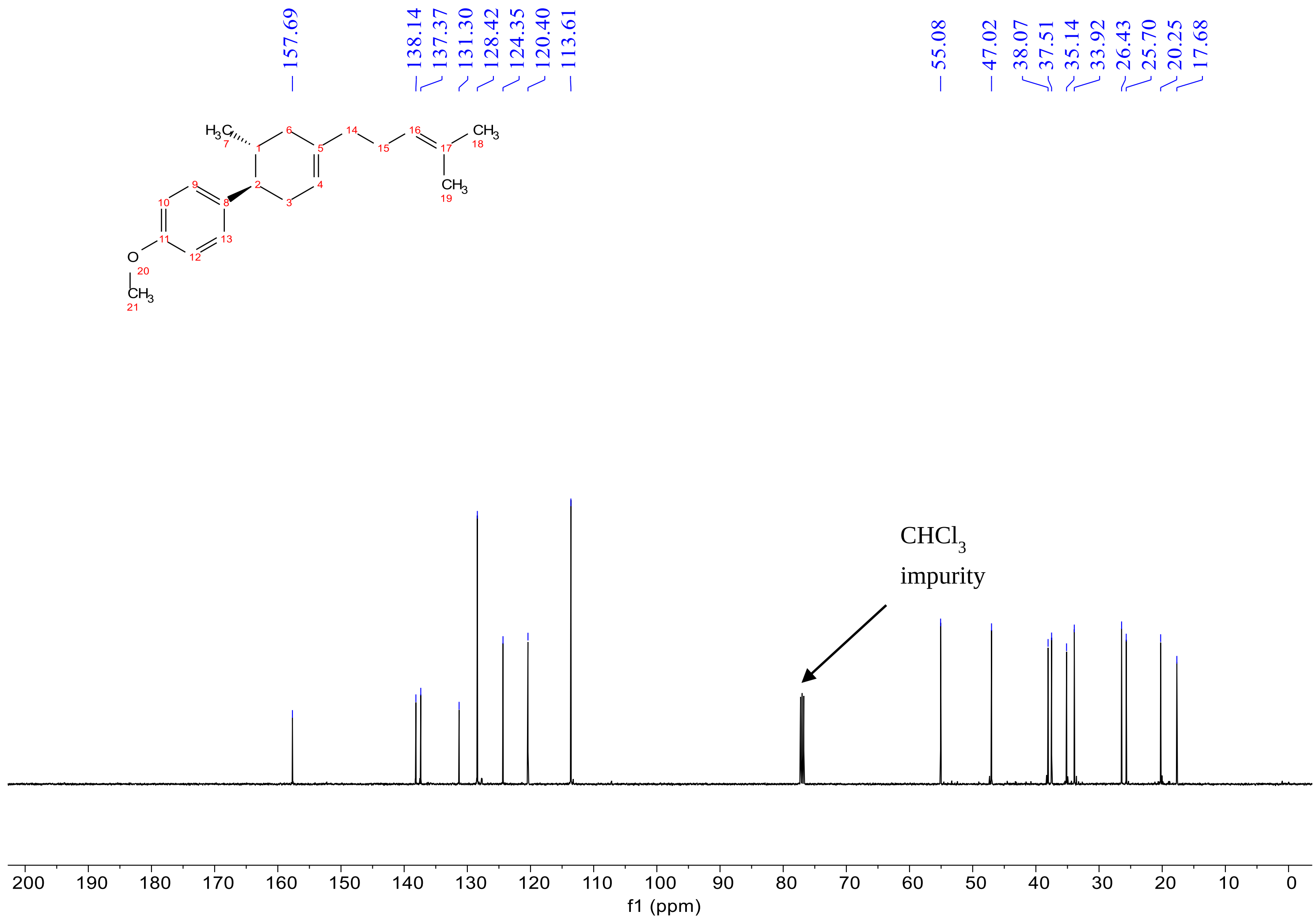

Supplementary Figure 141. ^{13}C NMR of **8d** (126 MHz, Chloroform-*d*)

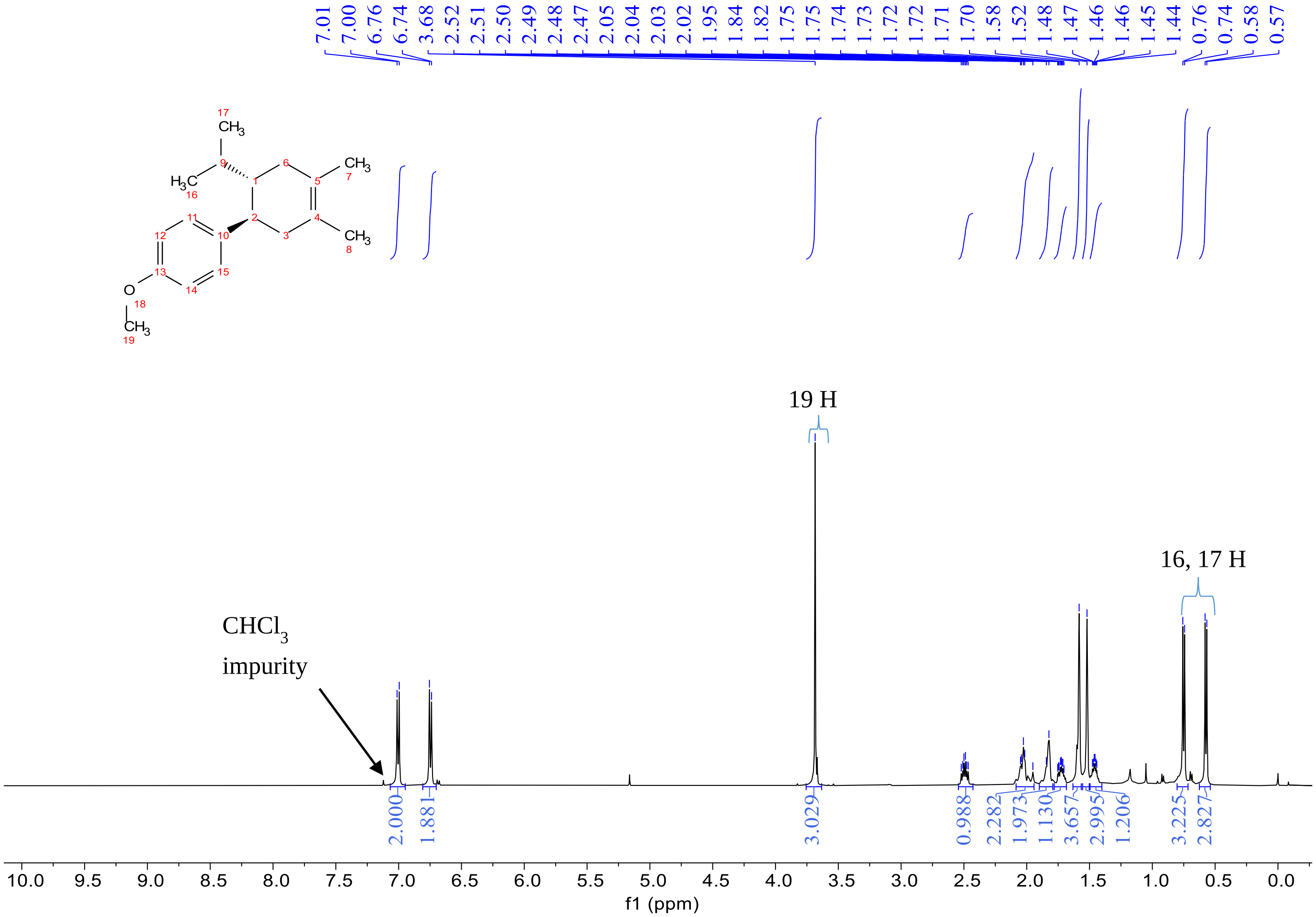

Supplementary Figure 142. ^{1}H NMR of **8e** (500 MHz, Chloroform-*d*)

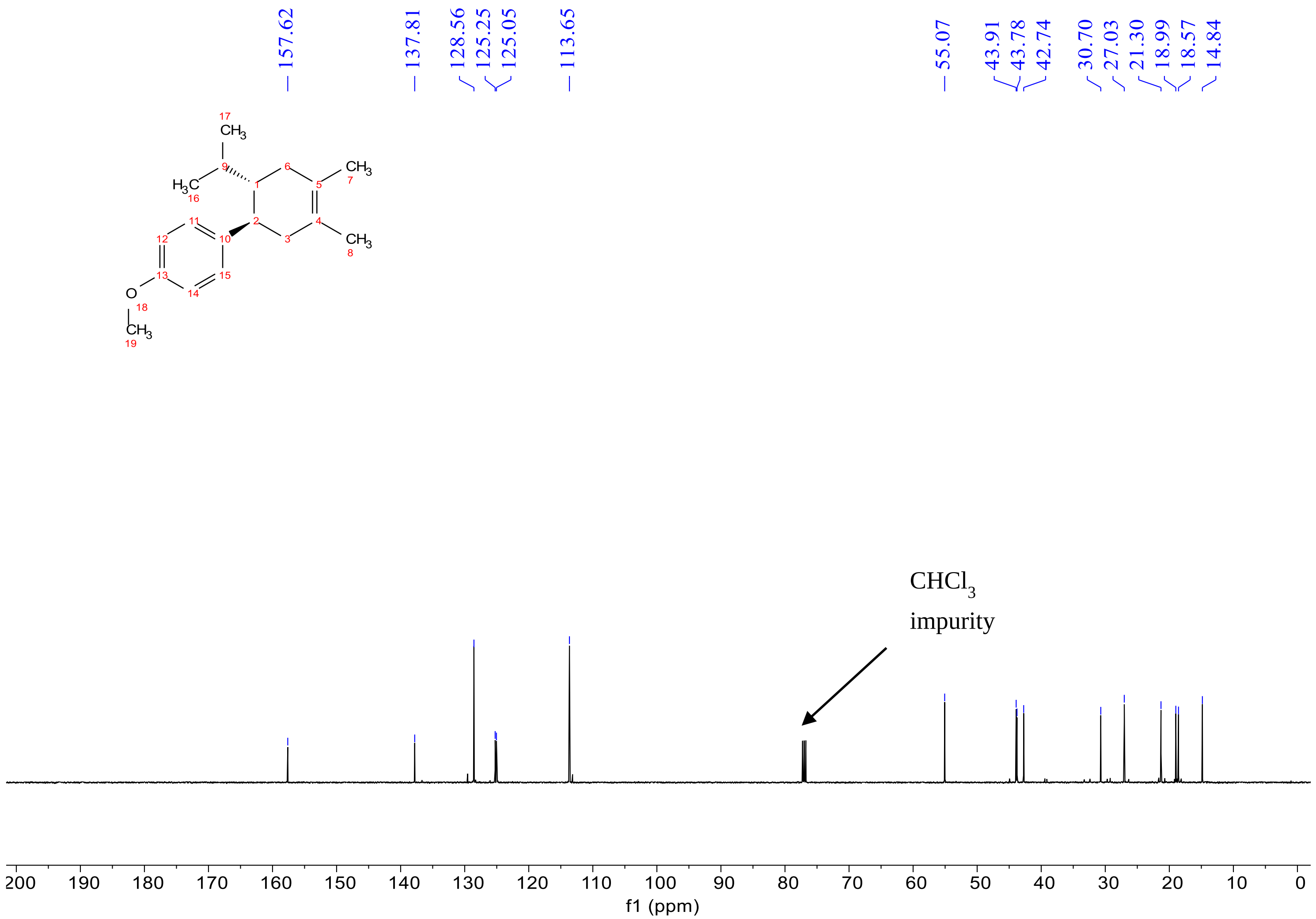

Supplementary Figure 143. ^{13}C NMR of **8e** (126 MHz, Chloroform-*d*)

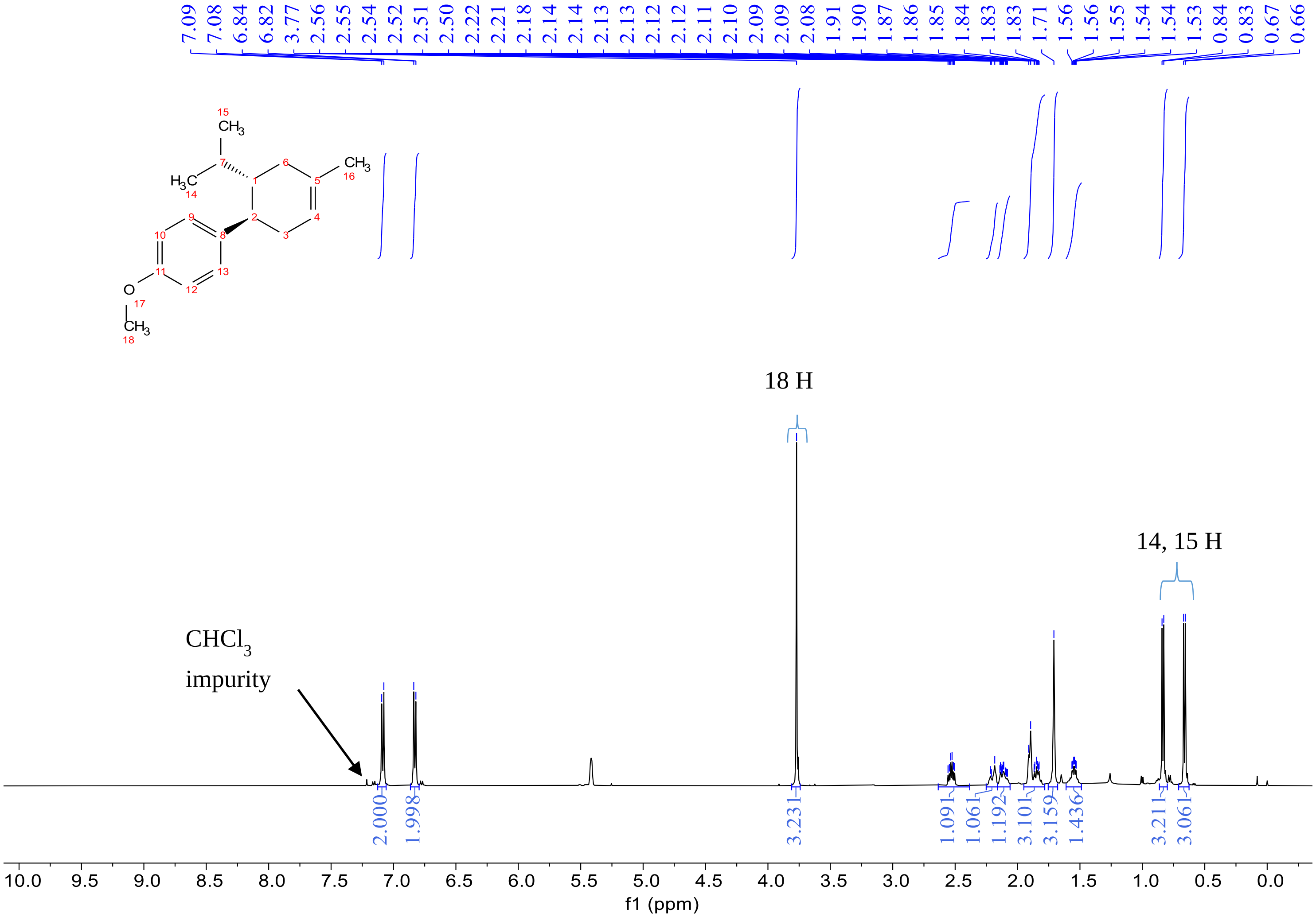

Supplementary Figure 144. ^{1}H NMR of **8f** (500 MHz, Chloroform-*d*)

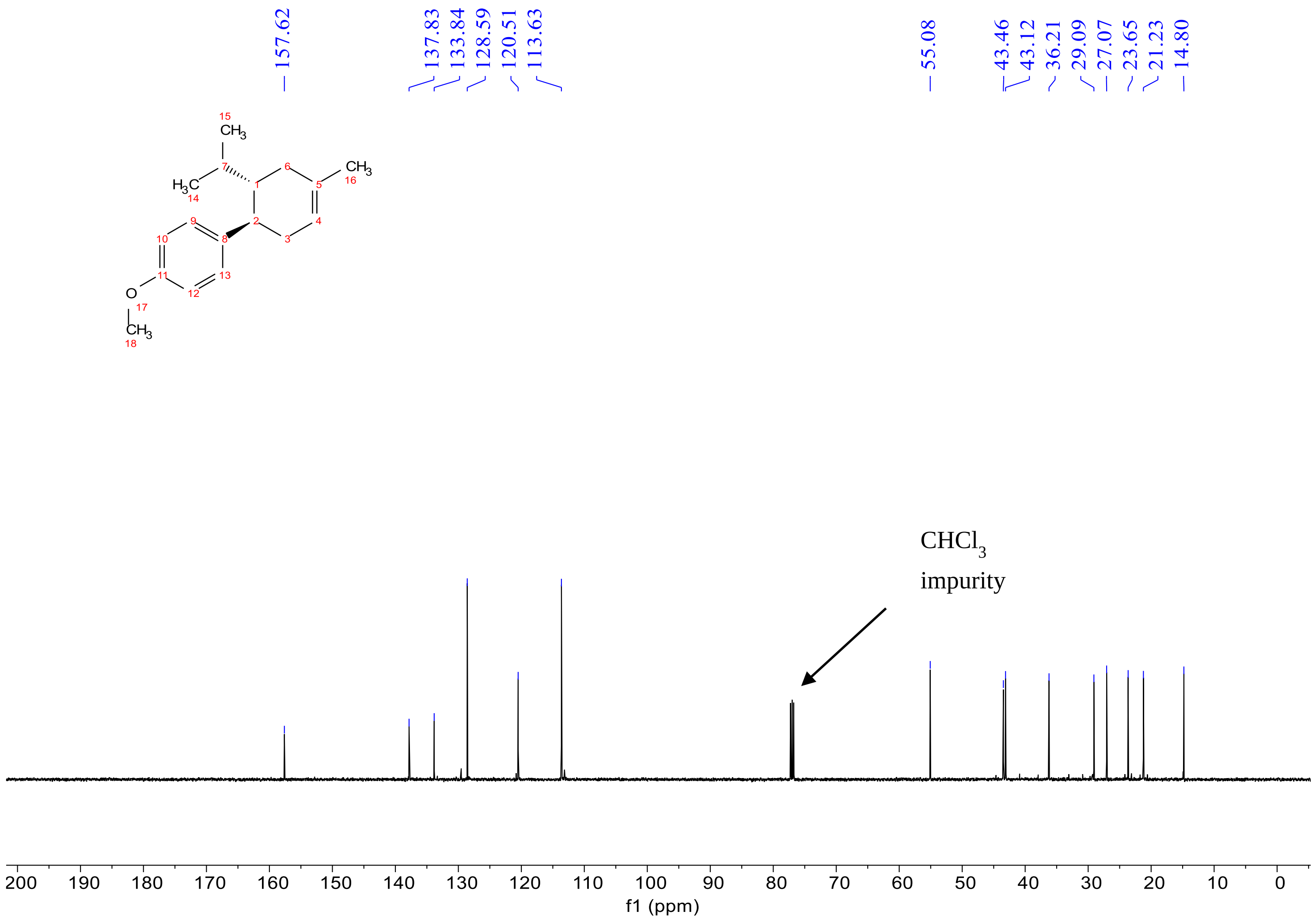

Supplementary Figure 145. ^{13}C NMR of **8f** (126 MHz, Chloroform-*d*)

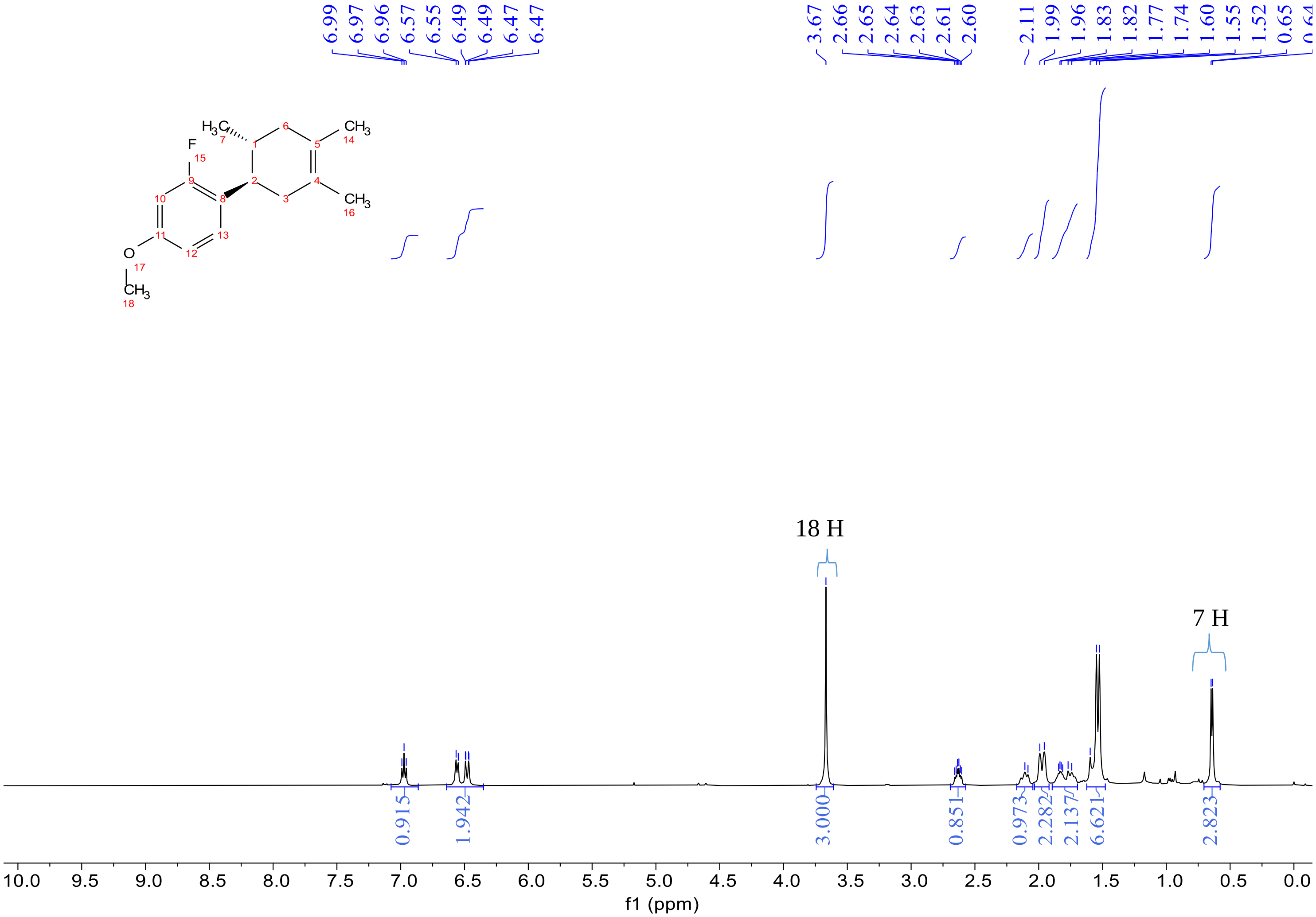

Supplementary Figure 146. ^{1}H NMR of **8g** (500 MHz, Chloroform-*d*)

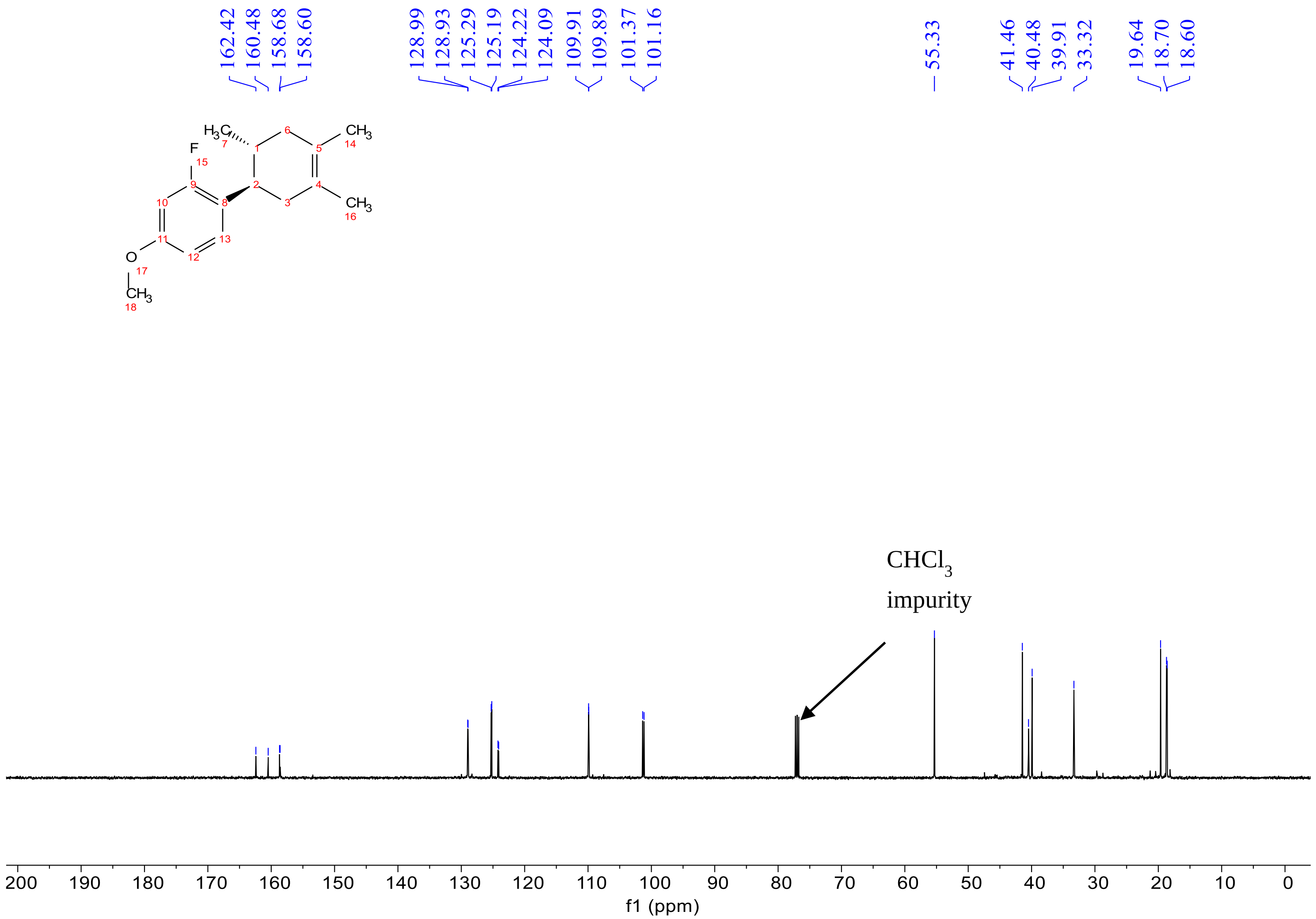

Supplementary Figure 147. ^{13}C NMR of **8g** (126 MHz, Chloroform-*d*)

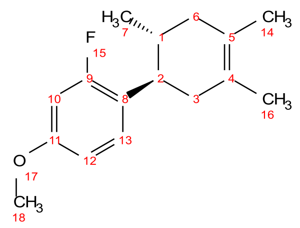

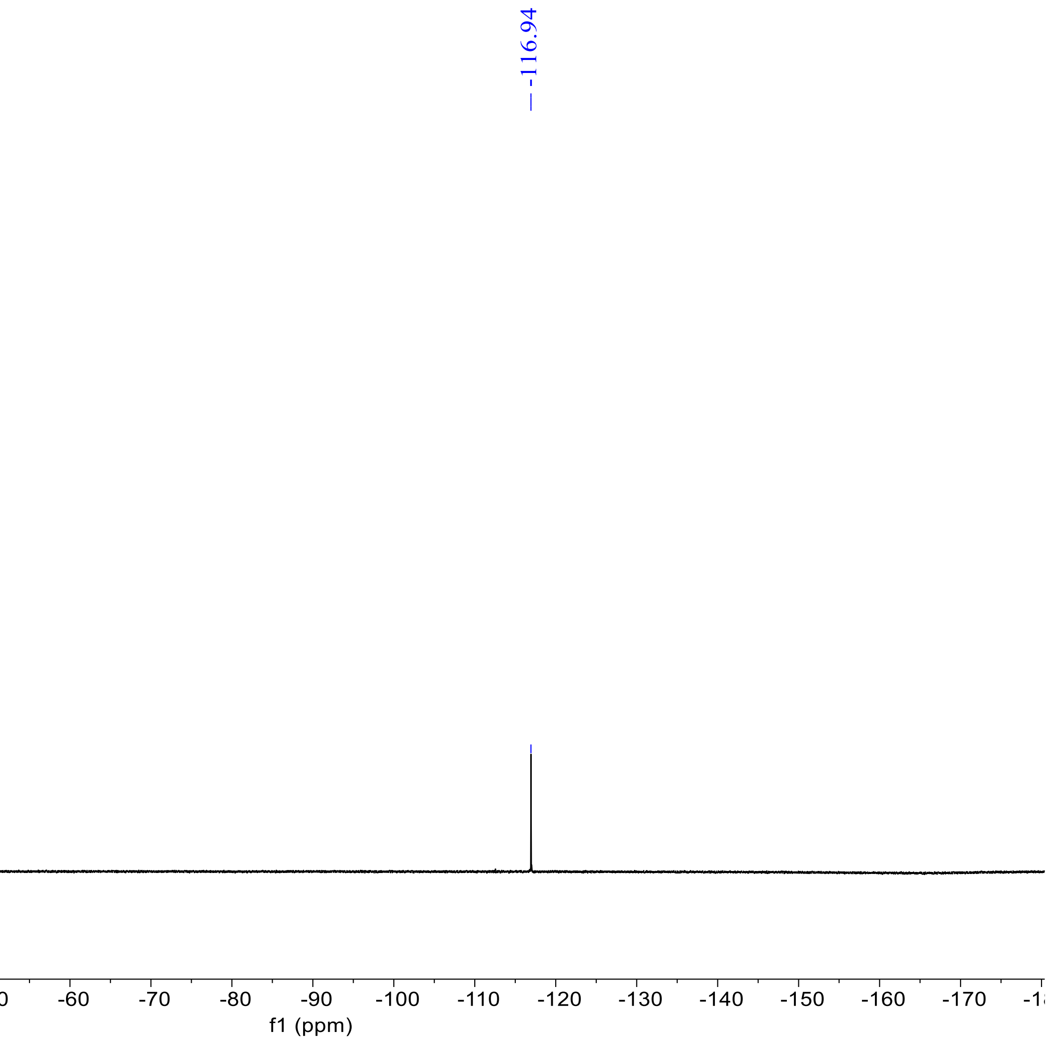

Supplementary Figure 148. ^{19}F NMR of **8g** (471 MHz, Chloroform-*d*)

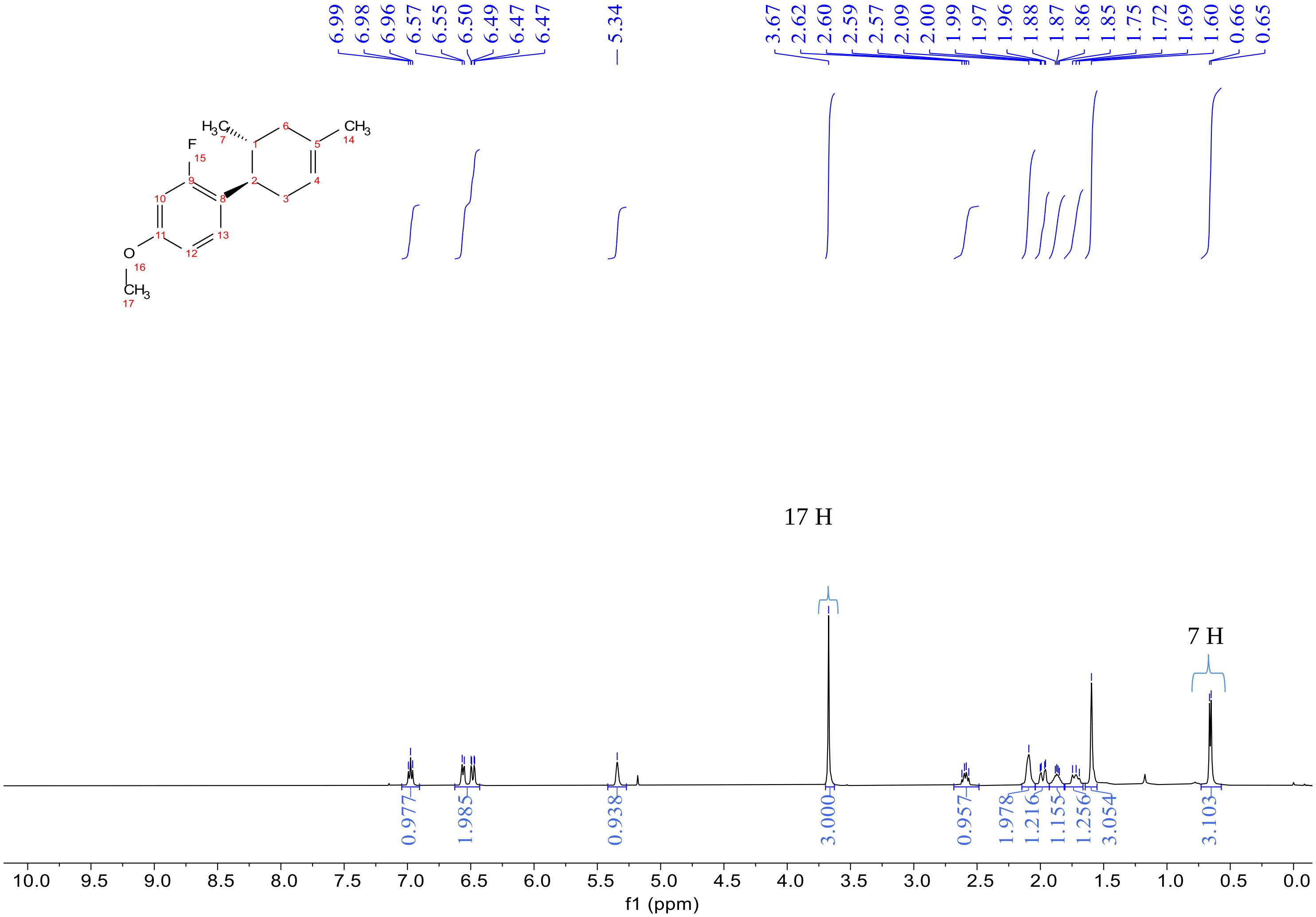

Supplementary Figure 149. ^{1}H NMR of **8h** (500 MHz, Chloroform-*d*)

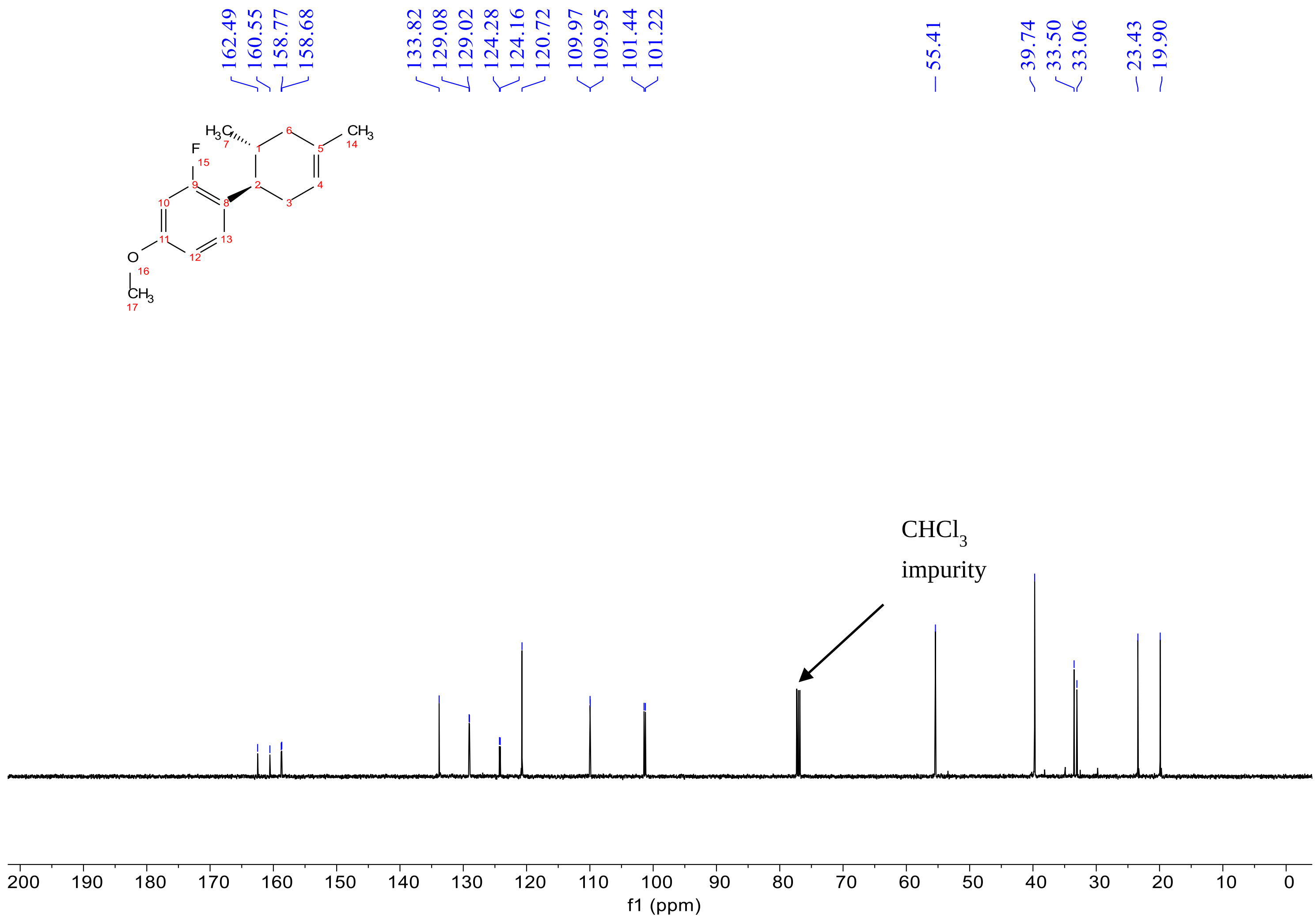

Supplementary Figure 150. ^{13}C NMR of **8h** (126 MHz, Chloroform-*d*)

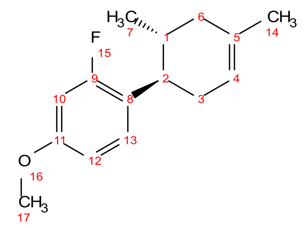

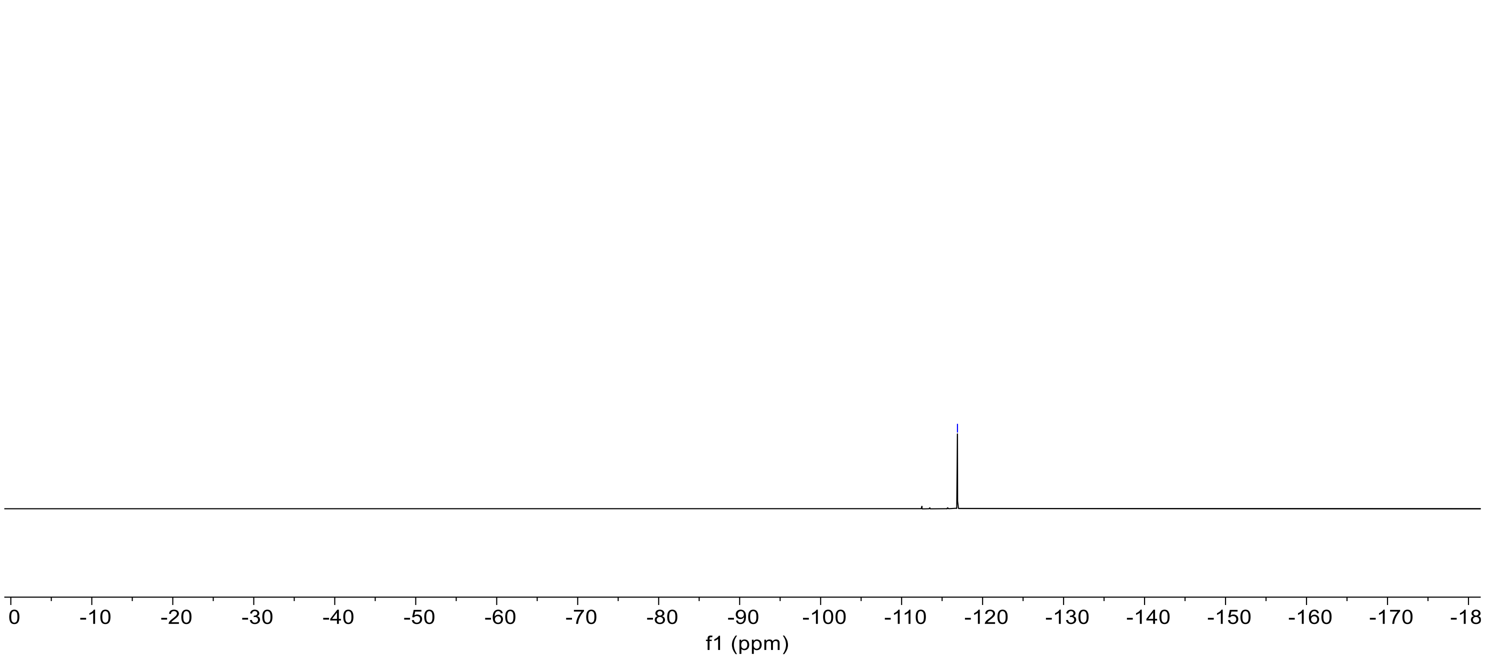

Supplementary Figure 151. ^{19}F NMR of **8h** (471 MHz, Chloroform-*d*)

5. References

1. Wu, W. et al. Involving single-atom silver(0) in selective dehalogenation by AgF under visible-light irradiation. *ACS Catal.* **9**, 6335-6341 (2019).
2. Amornpitoksuk, P.,Intarasuwan, K.,Suwanboon, S. & Baltrusaitis, J. Effect of phosphate salts (Na_3PO_4, Na_2HPO_4, and NaH_2PO_4) on Ag_3PO_4 morphology for photocatalytic dye degradation under visible light and toxicity of the degraded dye products. *Ind. Engin. Chem. Res.* **52**, 17369-17375 (2013).
3. Yi, Z. et al. An orthophosphate semiconductor with photooxidation properties under visible-light irradiation. *Nat. Mater.* **9**, 559-564 (2010).
4. Ke, J. et al. Facet-dependent electrooxidation of propylene into propylene oxide over Ag_3PO_4 crystals. *Nat. Commun.* **13**, 932 (2022).
5. Wang, Y. et al. Construction of six-oxygen-coordinated single Ni sites on g-C_3N_4 with boron-oxo species for photocatalytic water-activation-induced CO_2 reduction. *Adv. Mater.* **33**, 2105482 (2021).
6. Xing, Z. et al. Hollow Bi_2MoO_6 sphere effectively catalyzes the ambient electroreduction of N_2 to NH_3. *ACS Sustainable Chem. Eng.* **7**, 12692-12696 (2019).
7. Colomer, I.,Coura Barcelos, R. & Donohoe, T. J. Catalytic hypervalent iodine promoters lead to styrene dimerization and the formation of tri- and tetrasubstituted cyclobutanes. *Angew. Chem. Int. Ed.* **55**, 4748-4752 (2016).
8. Ischay, M. A.,Lu, Z. & Yoon, T. P. [2 + 2] cycloadditions by oxidative visible light photocatalysis. *J. Am. Chem. Soc.* **132**, 8572-8574 (2010).
9. Johnston, L. J. & Schepp, N. P. Reactivities of radical cations: Characterization of styrene radical cations and measurements of their reactivity toward nucleophiles. *J. Am. Chem. Soc.* **115**, 6564-6571 (1993).
10. Schepp, N. P. & Johnston, L. J. Reactivity of radical cations. Absolute kinetic data for cycloaddition reactions of styrene radical cations to alkenes. *J. Am. Chem. Soc.* **116**, 10330-10331 (1994).

11. Perdew, J. P.,Burke, K. & Ernzerhof, M. Generalized gradient approximation made simple. *Phys. Rev. Lett.* **77**, 3865-3868 (1996).

12. Blöchl, P. E. Projector augmented-wave method. *Phys. Rev. B* **50**, 17953-17979 (1994).

13. Kresse, G. & Joubert, D. From ultrasoft pseudopotentials to the projector augmented-wave method. *Phys. Rev. B* **59**, 1758-1775 (1999).

14. Kresse, G. & Furthmüller, J. Efficiency of ab-initio total energy calculations for metals and semiconductors using a plane-wave basis set. *Comput. Mater. Sci.* **6**, 15-50 (1996).

15. Grimme, S.,Antony, J.,Ehrlich, S. & Krieg, H. A consistent and accurate ab initio parametrization of density functional dispersion correction (DFT-d) for the 94 elements H-Pu. *J. Chem. Phys.* **132**, 154104 (2010).

16. Grimme, S.,Ehrlich, S. & Goerigk, L. Effect of the damping function in dispersion corrected density functional theory. *J. Comput. Chem.* **32**, 1456-1465 (2011).

17. Ischay, M. A.,Ament, M. S. & Yoon, T. P. Crossed intermolecular [2 + 2] cycloaddition of styrenes by visible light photocatalysis. *Chem. Sci.* **3**, 2807-2811 (2012).

18. Yu, Y.,Fu, Y. & Zhong, F. Benigncatalysis with iron:Facile assembly of cyclobutanes and cyclohexenes via intermolecular radical cation cycloadditions. *Green Chem.* **20**, 1743-1747 (2018).
