# Supplementary figures and images for: Ag3PO4 enables the generation of long-lived radical cations for visible light-driven [2 + 2] and [4 + 2] pericyclic reactions
Source: Nat Commun. 2024 Feb 1;15:979. doi: 10.1038/s41467-024-45217-y (PMC10834519; doi:10.1038/s41467-024-45217-y)

## Slide 1
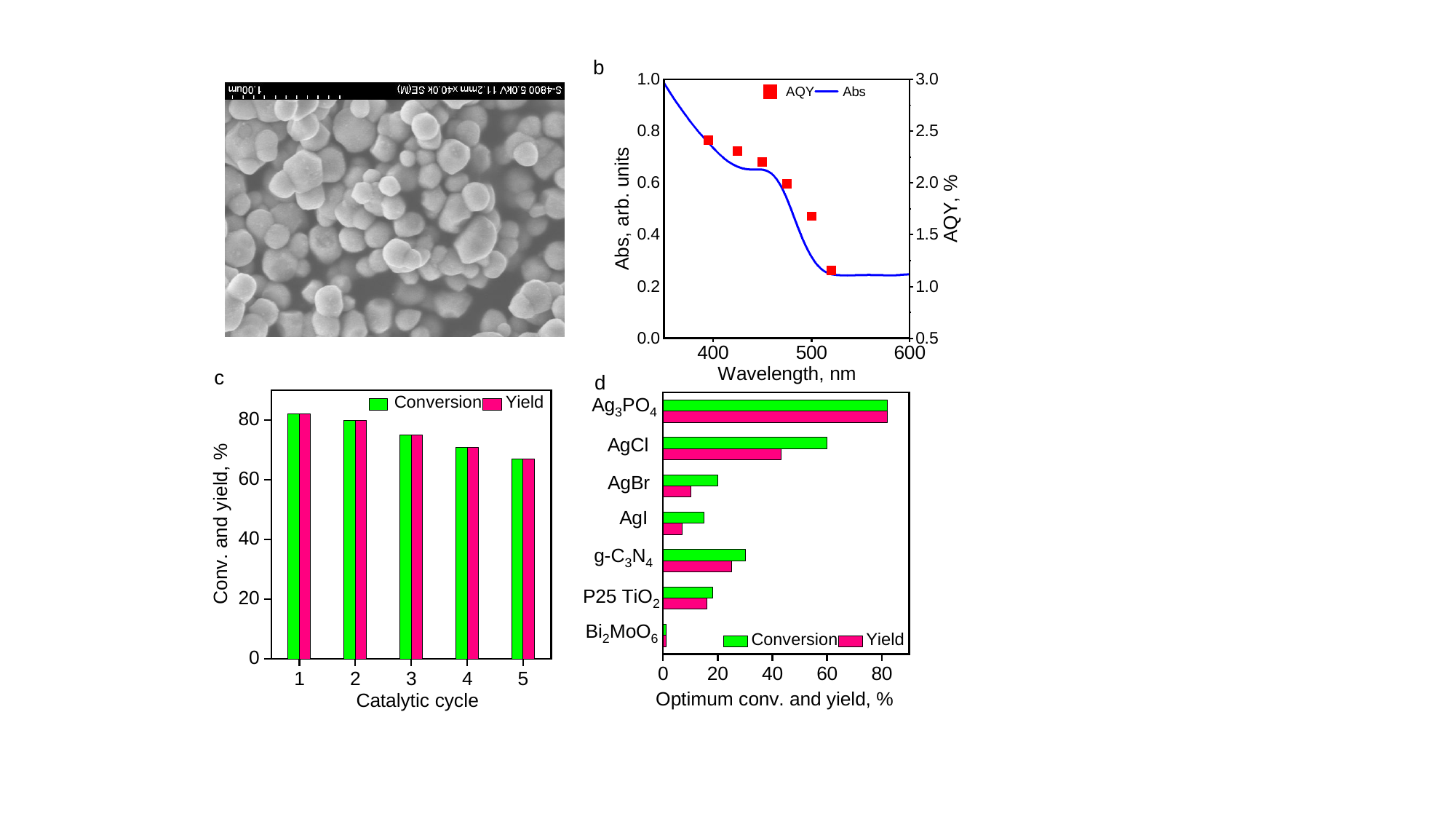

## Slide 2
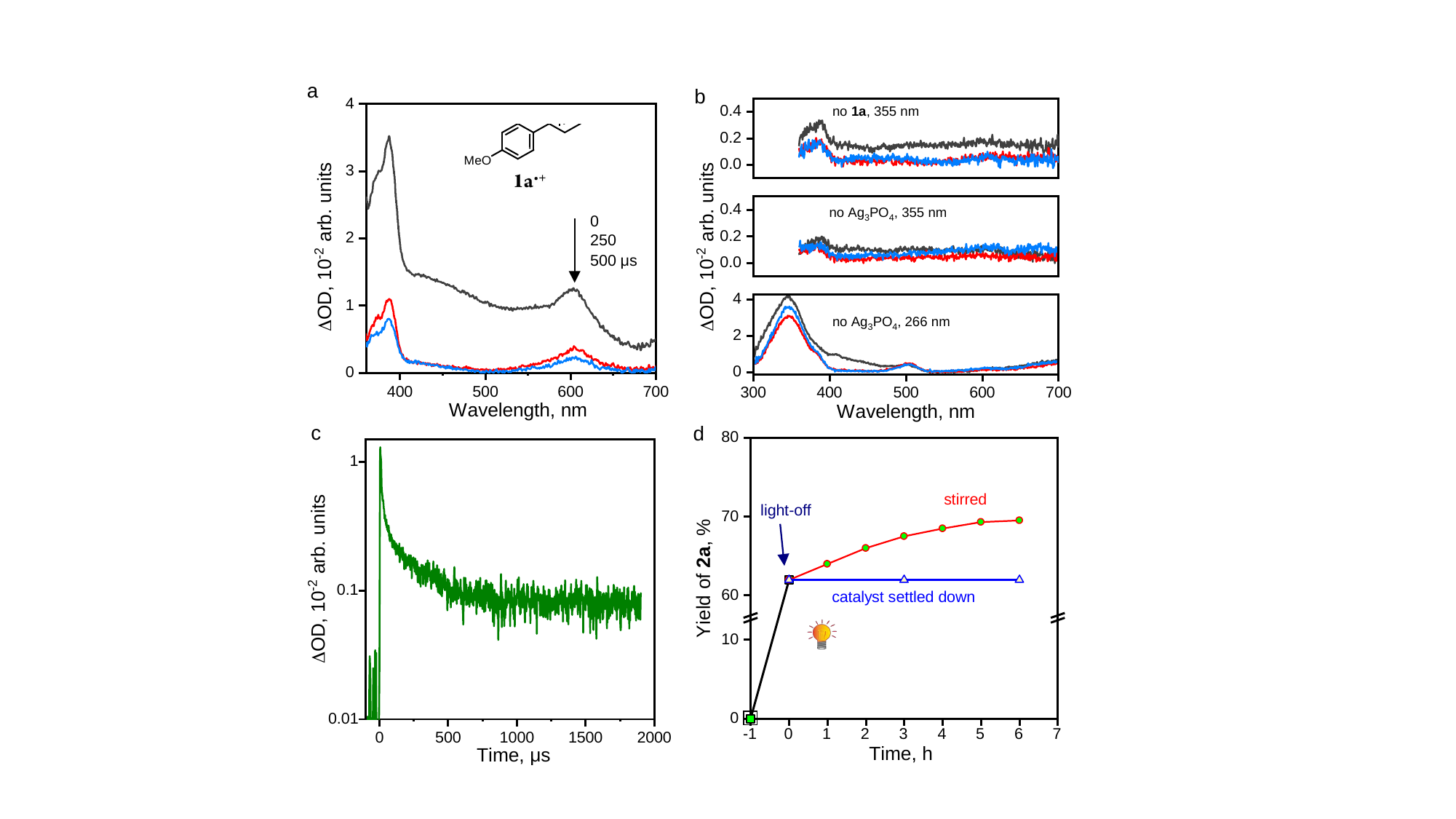

## Slide 3
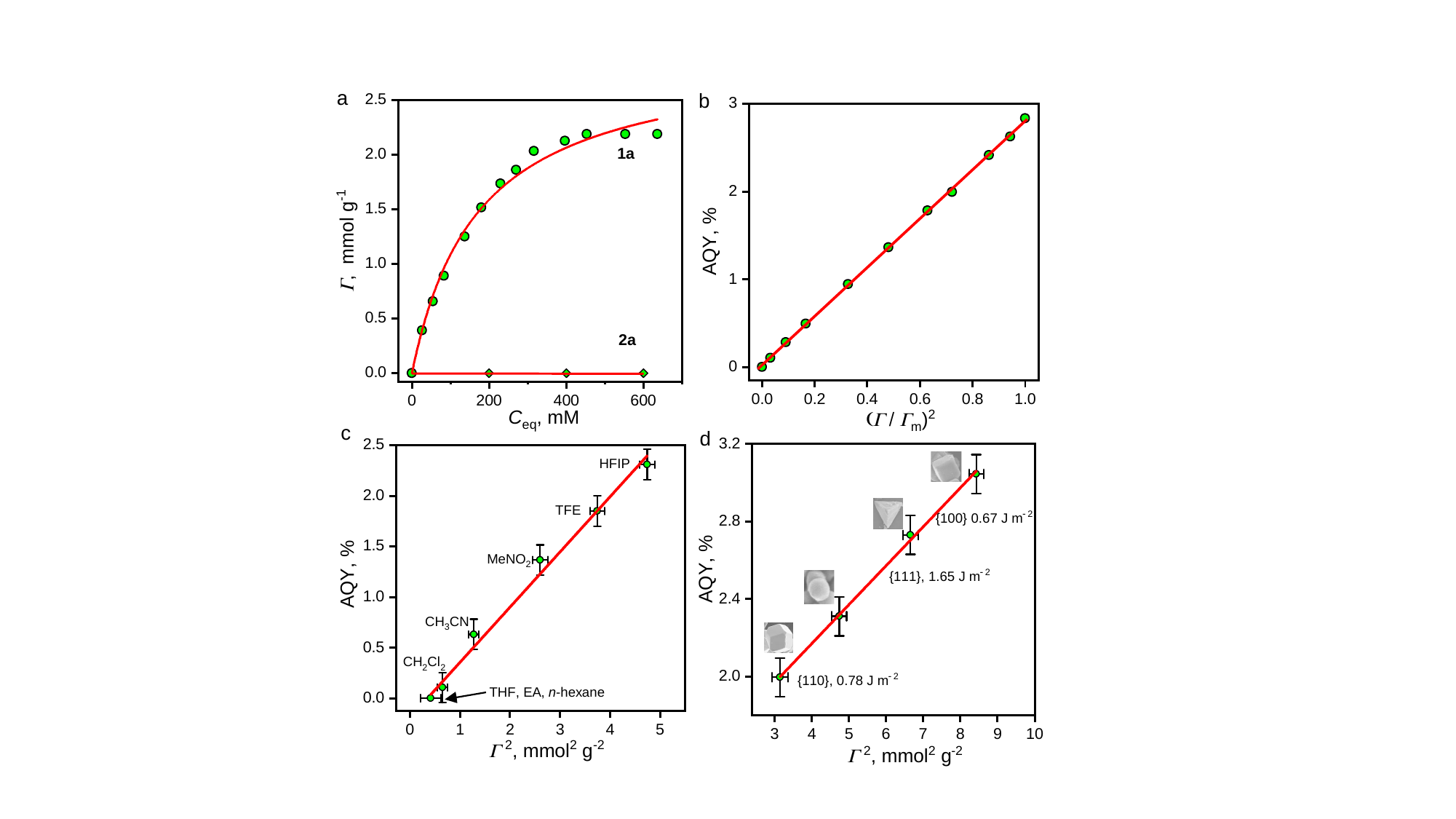

Supplement: Supplementary file 3 — Source Data [file 41467_2024_45217_MOESM3_ESM.pptx]
